# Computational Modeling of complete HOXB13 protein for predicting the functional effect of SNPs and the associated role in hereditary prostate cancer

**DOI:** 10.1038/srep43830

**Published:** 2017-03-08

**Authors:** Gopalakrishnan Chandrasekaran, Eu Chang Hwang, Taek Won Kang, Dong Deuk Kwon, Kwangsung Park, Je-Jung Lee, Vinoth-Kumar Lakshmanan

**Affiliations:** 1 Department of Biomedical Sciences, Chonnam National University Medical School, Gwangju, Republic of Korea; 2 Department of Urology, Chonnam National University Hospital, Gwangju, Republic of Korea; 3 Research Center for Cancer Immunotherapy, Chonnam National University Hospital, Gwangju, Republic of Korea

## Abstract

The human *HOXB13* gene encodes a 284 amino acid transcription factor belonging to the homeobox gene family containing a homeobox and a HoxA13 N-terminal domain. It is highly linked to hereditary prostate cancer, the majority of which is manifested as a result of a Single Nucleotide Polymorphism (SNP). In silico analysis of 95 missense SNP’s corresponding to the non-homeobox region of *HOXB13* predicted 21 nsSNP’s to be potentially deleterious. Among 123 UTR SNPs analysed by UTRScan, rs543028086, rs550968159, rs563065128 were found to affect the UNR_BS, GY-BOX and MBE UTR signals, respectively. Subsequent analysis by PolymiRTS revealed 23 UTR SNPs altering the miRNA binding site. The complete HOXB13_M26 protein structure was modelled using MODELLER v9.17. Computational analysis of the 21 nsSNP’s mapped into the HOXB13_M26 protein revealed seven nsSNP’s (rs761914407, rs8556, rs138213197, rs772962401, rs778843798, rs770620686 and rs587780165) seriously resulting in a damaging and deleterious effect on the protein. G84E, G135E, and A128V resulted in increased, while, R215C, C66R, Y80C and S122R resulted in decreased protein stability, ultimately predicted to result in the altered binding patterns of *HOXB13*. While the genotype-phenotype based effects of nsSNP’s were assessed, the exact biological and biochemical mechanism driven by the above predicted SNPs still needs to be extensively evaluated by *in vivo* and GWAS studies.

Prostate Cancer has been recorded as the leading cause of cancer deaths among men around the globe and the most common cause of cancer-associated deaths among men in the Republic of Korea^1^,^2^,^3^,^4^. Even though there has been a substantial decrease in the morbidity of prostate cancer, the incidence rate has been increasing over the last few decades^2^,^5^. Researchers have identified that several factors contribute to the development of prostate cancer, such as food style, sedentary lifestyle, etc.^6^,^7^,^8^. The hereditary predisposition for prostate cancer seems to be the major cause^1^,^7^,^9^,^10^ and almost 5–10% of the prostate cancer incidences have roots in genetic predisposition^9^,^11^,^12^,^13^.

The prostate cancer susceptibility locus in the human genome was identified as 17q21-22^7^,^14^ with the help of numerous GWAS studies and linkage analysis^12^,^15^. The human *HOXB13* gene consists of two exons and three introns and encodes a 284 amino acid protein (NCBI: NP_006352.2) belonging to the homeobox gene family^16^. It encodes a transcription factor which plays a major role in normal prostate development^17^,^18^. *HOXB13* is encoded by a single gene (NCBI: NM_006361.5) and contains a DNA binding homeobox domain of 60 amino acids (216–275 AA) and an HoxA13 N-terminal domain. The former binds to the DNA and is involved in transcription (see Supplementary Fig. S1). It is also associated with the increased risk of inherited prostate cancer^17^. In early 2012, *HOXB13* was found to be the most important hereditary prostate cancer susceptible gene^15^. Subsequent advances in genetic studies have proved that *HOXB13* has a major role in prostate cancer susceptibility, but the exact mechanism and mode of action remains undiscovered^17^,^19^.

The major genetic cause of Prostate cancer is the occurrence of Single Nucleotide Polymorphisms (SNPs)^9^,^20^,^21^. Among the various types of SNPs, non-synonymous SNPs^22^ (nsSNP’s) resulting in the change of amino acid^23^,^24^ is crucial and is associated with most hereditary prostate cancer^9^,^25^. These nsSNP’s might have deleterious^24^,^26^ and seriously damaging effects on protein structure, stability, activity, and function^27^,^28^,^29^. Peer researchers have confirmed that almost half of the hereditary cancers are associated with at least one form of SNP^7^,^22^,^30^,^31^.

Since the 3D structure for the complete human *HOXB13* is not available in the PDB (Protein Data Bank), the complete *HOXB13* protein structure needs to be modelled using MODELLER v9.17^32^ based on homology modelling. Comparative homology modelling constructs a 3D model by aligning the query sequence (NP_006352.2) known as the target against the closely related template structures obtained from the PDB^33^,^34^,^35^. Comparative modelling involves several computational steps^34^ (see Supplementary Fig. S2), and finally generates the best model for the query sequence which will be further evaluated for its validity^36^.

The complete list of SNPs for human *HOXB13* was retrieved from the dbSNP^37^,^38^ and Ensembl database^39^. Those SNPs were analyzed with SIFT, PolyPhen, PANTHER, PROVEAN, nsSNPAnalyzer, PhD-SNP, etc., to screen the most deleterious nsSNP’s affecting the protein structure and function. Further, the screened nsSNP’s were mapped onto the protein structure for structure-based functional analysis. The mapping of nsSNP’s to the given protein structure was done with the help of “mutation” tool in the SWISSPDB Viewer. Since the complete protein structure of the *HOXB13* protein was not available in the PDB, a complete *HOXB13* protein model was developed by comparative homology modelling^32^,^33^ by comparing and aligning the query sequence (*HOXB13* - NP_006352.2) against similar template 3D structures obtained from the PDB. The modelling was done with the help of MODELLER v9.17. The best *HOXB13* protein model constructed was selected and was then validated by Ramachandran Plot and PDBsum. The best protein model generated by MODELLER was considered as the native protein structure of *HOXB13* and was then subjected to single point mutation with the help of the “mutation tool” in SwissPDBViewer^40^,^41^. Energy minimization was done for both the native and the mutated proteins using NomadRefServer^42^. The RMSD values were then calculated, and the stability of the mutant protein structure was analyzed by the I-Mutant server^22^. Finally, all the energy minimized native and mutant protein structures were subjected to dihedral angle analysis of the atoms present in the amino acid residues by Ramachandran Plot^36^,^43^,^44^ to ascertain the conclusive effect of the encompassed nsSNP’s in the mutated protein structure (Figs 1 and 2).

## Results and Discussion

### Retrieval of functional *HOXB13* SNPs (Dataset)

**Figure 3:**
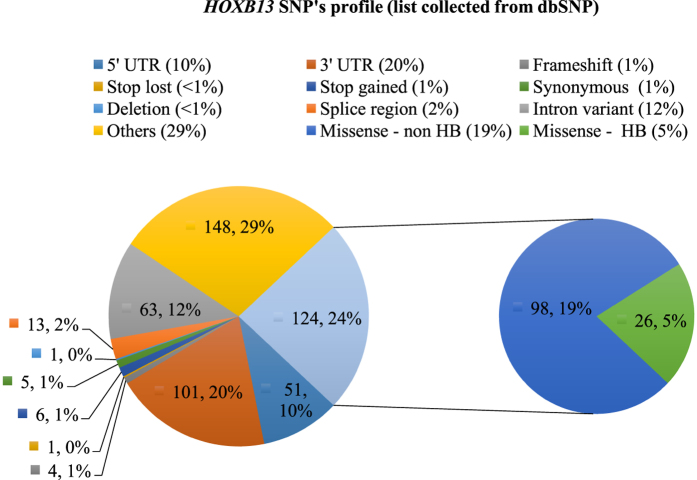
A statistical representation of the distribution of SNPs in the *HOXB13* gene. Distribution of SNPs in the *HOXB13* gene with insight into the homeobox (HB) and non-homeobox (non-HB) region missense SNPs (info collected from dbSNP database). The numerical figures (ex. 148, 63, 101) denote the no. of SNPs as recorded in the dbSNP database, whereas, the % (ex. 29%, 20%, 12%) represents the corresponding percent of the SNP variation among the overall SNPs.

The human *HOXB13* gene contained a total of 517 SNPs retrieved from the NCBI dbSNP database^37^ and were validated using Ensembl and HGVBase. Among the retrieved SNPs, missense, UTR, synonymous, and intronic SNPs accounted for about 123, 158, 57, and 63 (Fig. 3), respectively. The *HOXB13* protein had a functional homeobox binding domain (216–275 AA) accounting for about 4% of the missense SNPs (23 SNPs), whereas the remaining 95 missense SNPs fell in the non-homeobox region. In this study, the missense SNPs of the non-homeobox region of *HOXB13* were subjected to subsequent analysis for predicting their effects on the protein structure, stability, and function.

### Functionally deleterious nsSNP’s predicted by SIFT program (Sequence-based homology)

**Figure 4:**
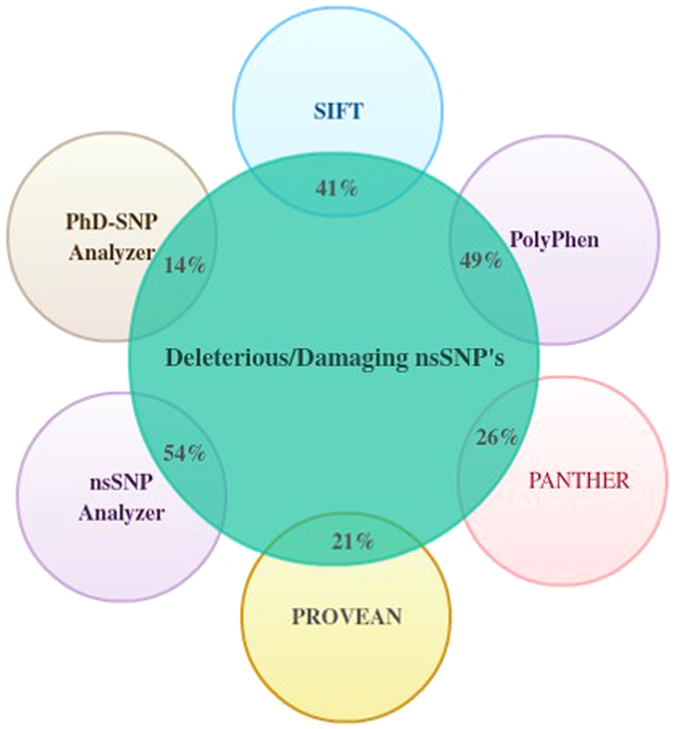
A statistical representation of the deleterious/damaging nsSNP predicted by various insilico tools. Percent deleterious nsSNP’s predicted by SIFT, PolyPhen, PANTHER, PROVEAN, nsSNPAnalyzer, PhD-SNP Analyzer. Out of 95 *HOXB13* non-homeobox nsSNP’s SIFT predicted 41%, PolyPhen predicted 49%, PANTHER predicted 26%, PROVEAN 21%, nsSNPAnalyzer 54% and PhD-SNPAnalyzer 14% of the nsSNP’s to be potentially deleterious/damaging.

From a total of 95 non-homeobox region nsSNP’s, 39 nsSNP’s were predicted to be functionally deleterious and were marked as “Affects protein structure” by the SIFT server (Table 1). Among those, 18 non-homeobox region nsSNP’s were marked as deleterious with a tolerance index of 0.00. The remaining 56 variants were predicted as “Tolerated” by the SIFT program. The detailed analysis of the effect of nsSNP’s on the entire non-homeobox region (1–215 and 276–284 AA) of *HOXB13* by the SIFT program can be found as Supplementary Fig. S3. The statistical representation of the results is given in Fig. 4. The complete SIFT prediction results can be found as Supplementary Table S1.

### The functionally damaging nsSNP’s predicted by PolyPhen version 2 server (Structure-based homology)

The PolyPhen server predicted 47 non-homeobox region nsSNP’s to be functionally deleterious to the protein structure. Out of those 47 nsSNP’s, 34 nsSNP’s were predicted to be “probably damaging” with the score ranging from 0.845 to 1.00 and the remaining 13 nsSNP’s were predicted to be “possibly damaging” with the score ranging from 0.537 to 0.851 (Table 2). Interestingly, 22 nsSNP’s predicted as deleterious by SIFT were also predicted to be functionally damaging by the PolyPhen server. Therefore, the 22nsSNP’s predicted commonly by SIFT and PolyPhen were of functional importance. The statistical representation of the results are given in Fig. 4. The complete PolyPhen prediction results are available as Supplementary Table S2.

### The functional validation of deleterious nsSNP’s by the PANTHER server

The PANTHER server predicted 25 non-homeobox region nsSNP’s to be damaging and the remaining nsSNP’s were predicted to be benign. Interestingly, 19 nsSNP’s were predicted as deleterious in common among SIFT, PolyPhen, and PANTHER server (Table 3). Additionally, two nsSNP’s (G153S, L152M) predicted by PANTHER and PolyPhen, and one nsSNP (H30Q) predicted by PANTHER and SIFT, were found to be common. The graphical representation of the results are given in Fig. 4. The complete PANTHER prediction results are available as Supplementary Table S3.

### The functional impact of deleterious nsSNP’s by the PROVEAN server

The PROVEAN server predicted 20 non-homeobox region nsSNP’s to be functionally damaging out of the 95 nsSNP’s submitted for analysis (Table 4). Among those, 16 nsSNP’s were found to be in common as predicted by SIFT, PolyPhen, and PANTHER servers. One nsSNP (D167N) was found to be in common to both predicted by SIFT and PolyPhen. Two nsSNP’s (G117E and G117R) were found to be in common with the nsSNP’s predicted by SIFT program. The graphical representation of the results are given Fig. 4. The complete PROVEAN prediction results are available as Supplementary Table S4.

### The functional impact of deleterious nsSNP’s by the nsSNPAnalyzer server

Out of the 95 nsSNP’s submitted for analysis, 51 nsSNP’s were predicted to be associated with a diseased phenotype. Among those, 20 nsSNP’s were common to those predicted by the above four servers (SIFT, PolyPhen, PANTHER, and PROVEAN) (Table 5). The graphical representation of the results are given in Fig. 4. The complete nsSNPAnalyzer prediction results are available as Supplementary Table S5.

### The functional impact of deleterious nsSNP’s by the PhD-SNP server

We used the SVM based method utilizing sequence and profile information algorithm for the analysis of 95 non-homeobox region nsSNP’s of the human *HOXB13* gene. The server predicted 13 nsSNP’s (Table 6) (Fig. 4) to be functionally associated with the disease and the remaining were considered benign. Among those, ten nsSNP’s were common to those predicted by the above-described servers (SIFT, PolyPhen, PANTHER, PROVEAN and nsSNPAnalyzer). The graphical representation of the results are given Fig. 4. The complete PhD-SNP prediction results are available as Supplementary Table S6.

Among the 95 *HOXB13* non-homeobox region nsSNP’s subjected to analysis by SIFT, PolyPhen, PANTHER, PROVEAN, nsSNPAnalyzer and PhD-SNP servers, 21 nsSNP’s were found to be functionally significant and causing damaging effects to the *HOXB13* protein structure, stability, and function by the servers mentioned above. The list of those 21 nsSNP’s are as follows: rs761914407 (R215C), rs779330626 (Q188R), rs570681642 (Q181R), rs777986934 (G177V), rs587780164 (D167N), rs766929278 (G153V), rs575899185 (Q138H), rs770891609 (Y137S), rs769634543 (G135E), rs775273363 (A128V), rs201428095 (R123H), rs8556 (S122R), rs138213197 (G84E), rs772962401 (Y80C), rs778843798 (C66R), rs199813155 (C63Y), rs758166293 (C63G), rs568967699 (K61M), rs770620686 (P59L), rs561048036 (H30Q), rs587780165 (R25Q). For subsequent analysis, these 21 nsSNP’s were taken into consideration. The *HOXB13* protein structure was available only for the homeobox binding domain (216–275 AA) of the complete protein and since there was no complete structure of *HOXB13* in the PDB, the Homology Modelling approach was adopted for simulating the complete protein structure of *HOXB13* *in silico*, so that we could map the above screened 21 nsSNP’s into the protein structure and could predict their effects on the protein function, stability, and bioactivity.

### Functionally significant *HOXB13* UTR SNPs predicted by the UTRscan server

Mutations in the untranslated region of the gene were reported very often to be linked with hereditary diseases such as cancer and various immune deficiency syndromes and also plays a key role in mRNA localization, stability, and translational efficiency^45^. Both the 5′UTR and the 3′UTR have important functions concerning the stability and expression of the mature mRNA. Mutations in those regions are linked with severe effects on the expression patterns of the gene at the level of mNA processing and translation^46^. The polymorphisms in the 5′ UTR are increasingly related to the altered patterns of ribosomal binding capacity, stability and translational regulation of mRNA, thereby influencing the RNA half-life. Whereas the polymorphisms corresponding to the 3′ UTR are highly involved in altered patterns of polyadenylation, localization, stability, translational efficiency and microRNA (miRNA) binding specificity, thereby rendering a tremendous effect on the gene expression patterns.

The UTRscan server predicts both the 5′UTR and 3′UTR SNPs. Among a total of 101 valid 3′UTR SNPs taken for evaluation, the UTRscan server predicted three SNPs (rs543028086, rs550968159, rs563065128) to be functionally significant to cause a pattern change (Table 7). However, the UTRscan server did not predict any harmful 5′ UTR SNPs.

UNR (Upstream of N-ras) is a transcription factor containing five cold shock domains (CSD) that bind to single-stranded DNA and RNA^47^. It controls and plays a major role in transcriptional and post-transcriptional gene expression. UNR is a cytoplasmic protein known to function as an RNA chaperone and is found to be crucial in the control of cell proliferation and death^48^,^49^. The protein mainly destabilizes the c-fos mRNA and helps in the initiation and activation of cap-independent translation via the IRES for various transcripts, especially the proto-oncogene c-myc, rhinovirus, poliovirus, the cell cycle PISTLRE kinase, and pro-apoptotic factor (Apaf-1)^50^,^51^. The SNP rs543028086 was predicted to result in the disruption of the UNR Binding Site (UNR_BS) motif in the 3′UTR of human *HOXB13* gene, which results in the deregulation of pro-apoptotic factor (Apaf-1), which might have a negative effect on the control of cell death.

The GY-Box is a conserved motif present in the Notch pathway target genes in Drosophila^52^. It is highly conserved in 3′ UTR regions that have sequence complementarity to the 5′ regions of the miRNA seed region. The result is the formation of RNA duplexes by the interaction of the 3′ UTR end of mRNA and the 5′ end of the miRNA, leading to translational repression^52^. The SNP rs550968159 is present in this region of the 3′UTR of human *HOXB13* gene and leads to the loss of the specific GY-Box pattern, hence voiding the chance of translational repression of *HOXB13* mediated by the GY-Box in the 3′UT region. Thus, an imbalance in the feedback regulation of *HOXB13* expression has been predicted to result in the diseased state.

The Mushashi Binding Element (MBE) is an mRNA binding protein, which plays a very important role in the regulation of stem cell renewal process^53^,^54^ by suppressing the translation of all the mRNA coding for the proteins involved in inhibiting cell cycle progression^55^,^56^. The 3′ UTR SNP rs563065128 results in the loss of the MBE UTR motif in the human *HOXB13* gene and is thereby found to lose its natural role of regulating the stem cell renewal process by suppressing the expression of cell cycle progression inhibitors, thereby predicted to result in the loss of stem cell niche. Loss of stem cell niche, in turn, leads to the unavailability of the local stem cell source to replenish the damaged cells of the tissue, thus, leading to the disease state.

These three 3′UTR SNPs were predicted to have important deleterious effects and functional significance on the expression of human *HOXB13* gene.

### Functionally significant *HOXB13* 3′ UTR SNPs predicted by the PolymiRTS Database

Out of the 95 nsSNP’s under consideration, only 23 nsSNP’s were found to have a crucial role in the 3′ UTR region (see Supplementary Table S7). Among those, five nsSNP’s (rs8064432, rs79812861, rs148791210, rs184053751 and rs183620920) were found to disrupt only the conserved (ancestral allele with support >=2) miRNA sites. Two nsSNP’s (rs116931900 and rs1056656) were exclusively found to create a new miRNA site. Whereas, the remaining 16 nsSNP’s were predicted to be involved in the disruption and creation of a new miRNA site, out of which rs61123825 (disrupting – 2 and creating – 7) and rs192244427 (disrupting – 4 and creating 5) were found to have a maximum of 9 pattern changes.

### Modelling of the complete *HOXB13* protein using MODELLER v9.17 (Comparative Homology Modelling)

The PDB contains only the 3D structure of the Homeobox binding domain of the human *HOXB13* protein and not the complete 3D structure. In order to further analyze the effect of the above shortlisted 21 non-homeobox region missense SNPs on the *HOXB13* protein structure and function, the complete protein structure was mandatory, since further analysis of those nsSNP’s demands mapping the nsSNP’s into the protein structure and thereby validating their subsequent effects on structural and functional aspects of the protein *in silico*. Thus, the complete *HOXB13* protein structured was modelled by a technique called comparative homology modelling using the MODELLER v9.17 tool from the Andrej Sali laboratory. The complete modelling procedure and the steps performed were mentioned in the Supplementary Material.

A suitable template structure for developing the model was obtained using psiBLAST by setting PDB as the source database^57^ for finding the 3D structure templates. The resulting sequences of at least >30% similarity and identity were picked for comparative homology modelling. The Supplementary Table S8 shows the results of psiBLAST. PDB ID 2CRA was found to have 100% identity with the query sequence and PDB ID 2LD5 and 2L7Z showed 78% identity with the query sequence and were chosen as the template for modelling the *HOXB13* protein. The respective “.pdb” files of the above-mentioned proteins were downloaded and kept in the same folder where the python script files were located. These three PDB ID structures – 2CRA, 2LD5 and 2L7Z were used as the template for modelling the complete *HOXB13* protein using comparative homology modelling by MODELLER v9.17. The distance tree of the query sequence and the protein structures from the PDB computed by psiBLAST are available as Supplementary Fig. S4.

From the results, we found that 2LD5 and 2L7Z were both structurally and sequentially identical with the same crystallographic resolution of 1.0 Å (as Supplementary Fig. S5). Conversely, the structure 2CRA was found to be diversified from both 2LD5 and 2L7Z with a distance score of 63.5. Hence, the structure 2L7Z was finally selected for modelling of complete *HOXB13* because of its high sequence and structural similarity to the query sequence. The alignment of the query sequence (NP_006352) with the template structure 2L7Z was done (Supplementary Fig. S6). MODELLER v9.17 was instructed to generate 30 similar models of complete *HOXB13* protein based on the 2L7Z template structure and “hox13-2l7z.ali” file (Supplementary Fig. S7). There are several criteria to select the best model among the various models generated by MODELLER v9.17. The most important and widely practised criteria includes selecting the model with the lowest DOPE score^58^ and the highest GA341 score^59^. Accordingly, from the summary of the models generated (Supplementary Fig. S7.), we found that the 26^th^ model “hoxb13.B99990026.pdb” had the lowest DOPE score of −10661.23047 and the highest GA341 score of 1.00000. Thus, the selected model “hoxb13.B99990026.pdb” was subjected to further validation of protein structure and folding properties with the help of the Ramachandran Plot and PDBsum.

### Model validation by Ramachandran Plot

**Figure 5:**
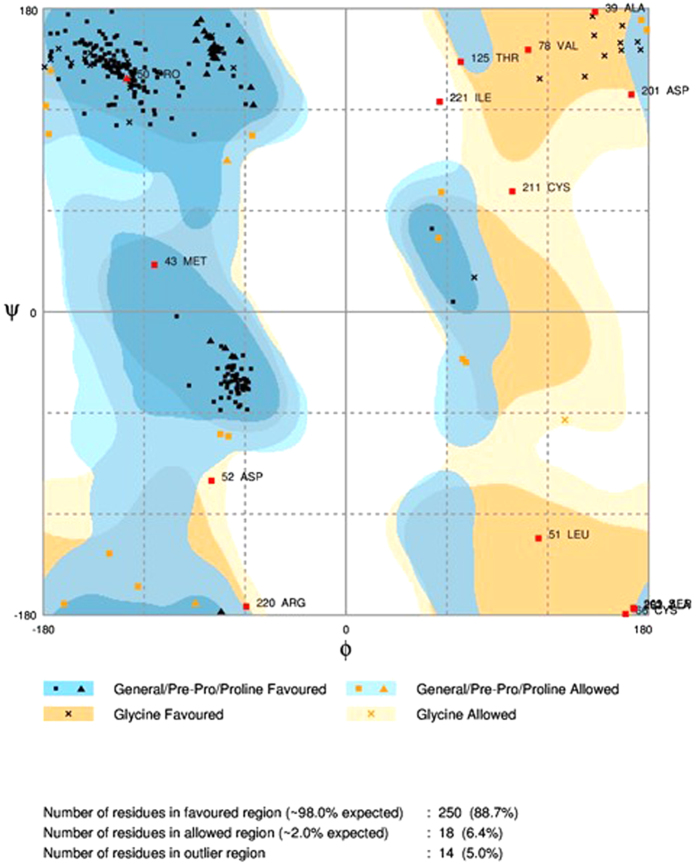
Ramachandran Plot for the generated protein model “hoxb13.B99990026.pdb”. Almost 89% of the amino acid residues in the modelled protein “hoxb13.B99990026.pdb” occupied the favored region, 6% of the residues occupied the allowed region, and the remaining 5% of the residues occupied the outlier region, respectively.

The selected *HOXB13* protein model “hoxb13.B99990026.pdb” was validated and authenticated as the best-generated model and was subjected to analysis for the backbone dihedral angles (phi and psi) of the amino acid residues in the protein structure^44^. For a good protein structure, it is expected that there should be more than 90% of the residues in the core or favoured region of the protein^36^,^43^. The generated model “hoxb13.B99990026.pdb” was analyzed by RAMPAGE and was found to have 250 residues (88.7%) in the favored region, 18 residues (6.4%) in the allowed region and 14 residues (5.0%) in the outlier region, respectively (Fig. 5.) The model was found to be good and reliable since approximately 89% of the residues fell in the favored region and also because of the low DOPE and high GA341 score.

### Model validation by PDBsum

**Figure 6:**
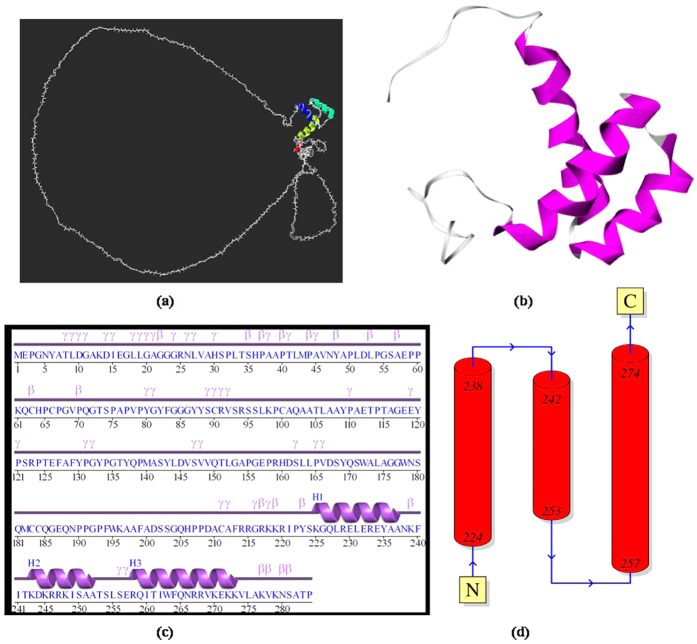
Validation results of the hoxb13.B99990026.pdb modelled protein by PDBsum. (**a**) 3D protein structure of the complete *HOXB13* protein (hoxb13.B99990026.pdb) modelled using MODELLER v9.17 (Figure generated using SWISSPDBViewer). (**b**) *HOXB13* (2CRA) protein 3D structure (Figure generated using PDBsum). (**c**) The amino acid residues contributing to the secondary structure (alpha helix and beta turns) of the complete *HOXB13* protein are depicted in the topology diagram (Figure simulated using PDBsum). (**d**) Linear view of the modelled complete *HOXB13* protein structure with alpha helices, beta and gamma turn and corresponding amino acid residues (Figure simulated using PDBsum).

The simulated *HOXB13* protein model was further validated with the help of PDBsum for information regarding the motifs, helices, strands, domains, tunnels, angles, positions, error, etc., present in the 3D structure of the proteins^60^. The “hoxb13.B99990026.pdb” was subjected to analysis by PDBsum and was found to have three alpha helices, three helix-helix interactions, 18 beta, and 40 gamma turns, respectively (Fig. 6). The results were in accordance with the features of the homeobox domain of the *HOXB13* protein (2CRA). The complete 3D structure of the protein is given in Fig. 6(a). The complete *HOXB13* protein, which was modelled using MODELLER v9.17, contained the same features and folding patterns of the homeobox domain of the *HOXB13* protein (2CRA), which was clearly evident from Fig. 6(a,b). The detailed protein 3D structure features can be found as Supplementary Fig. S8.

Thus, the protein model “hoxb13.B99990026.pdb” generated by MODELLER v9.17 was found to be the best model based upon the DOPE and GA341 scores and was further validated to be good with the help of the Ramachandra Plot and PDBsum analysis. Hence, this model was taken as the complete human *HOXB13* protein structure for further analysis of the corresponding deleterious nsSNP’s. The model “hoxb13.B99990026.pdb” was denoted as “HOXB13_M26” protein structure in the subsequent analysis.

### Mapping the missense amino acid variation into the protein

#### Protein template for performing mutation and subsequent analysis

The HOXB13_M26 protein structure was taken as the complete native protein structure for mapping the previously predicted 21 deleterious nsSNP’s and also, for further studying their effect on the protein.

#### Protein mutation and Energy minimization of the native and mutated protein

The 21 nsSNP’s screened to be potentially deleterious by various servers were mapped into the HOXB13_M26 protein using the “mutation” tool in SwissPDBViewer^40^,^61^. The resulting 21 mutated proteins were denoted as “HOXB13_M26” Mutant.

In order to mimic the *in vivo* folding conditions and parameters of the protein, energy minimization of both the native (HOXB13_M26) and all the mutant proteins (HOXB13_M26 Mutant) was done with the help of NomadRef Gromacs Server using conjugant gradient force fields^42^. The resulting energy values of all of the native and the mutant structures are given in Table 8.

**Figure 7:**
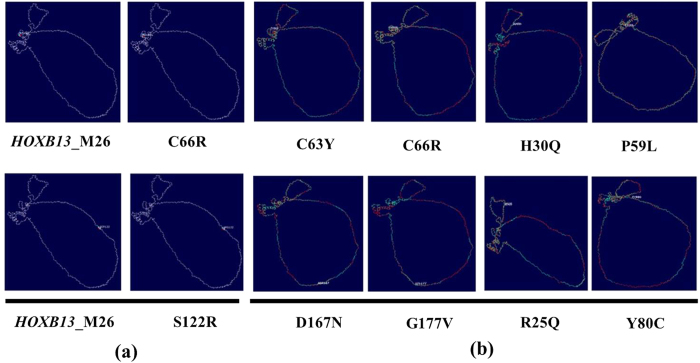
Electron Cloud densities and RMSD value calculation of the mutant proteins. (**a**) Figure depicting the electron cloud density difference between the native protein structure and the mutants C66R and S122R. The images were modelled using SwissPDBViewer. (**b**) Superimposed native (HOXB13_M26) and mutant protein structures C63Y (1.80 Å), C66R (1.68 Å), D167N (1.69 Å), G177V (1.56 Å), H30Q (2.02 Å), P59L (1.80 Å), R25Q (1.52 Å) and Y80C (1.23 Å). The RMSD values and the image were modelled using SwissPDBViewer.

The total energy of the native protein structure HOXB13_M26 was determined to be −4505.484 KJ/mol. Among all the 21 mutants, the mutant C66R and S122R were found to have the highest energy of −4763.567 KJ/mol and −4745.173 KJ/mol, respectively even after energy minimization, when compared with the native structure. The mutants D167N (−477.684 KJ/mol), H30Q (−4675.440 KJ/mol), C63G (−4663.925 KJ/mol), Q181R (−4601.128 KJ/mol), C63Y (−596.328 KJ/mol) and Q188R (−4588.476 KJ/mol) were found to have considerably higher energy values, whereas the mutants R215C, R123H and Q138H showed very small energy values of −4273.583 KJ/mol, −4274.708 KJ/mol, −4376.856 KJ/mol, respectively, after energy minimization. These nsSNP’s with very high and low energies when compared to the native protein structure implied a possible underlying damaging effect on the protein structure, thereby affecting the protein stability and function. The other nsSNP’s were found to have near equal energy values as compared with the native structure. The electron cloud density maps of the variants C66R and S122 that had the highest energy are given in Fig. 7(a).

#### RMSD value calculation of the modelled protein

Among the 21 mutants analyzed, the variant H30Q was found to have the highest RMSD value of 2.02 Å, followed by C63Y and P59L, having 1.80 Å each, respectively (Fig. 7(b)). The mutants D167N, C66R, G177V, R25Q and Y80C, were found to have RMSD values of 1.69 Å, 1.68 Å, 1.56 Å, 1.52 Å and 1.2 Å, respectively (Table 8). The remaining mutants were found to have RMSD values of less than 1 Å. Among the mutants with high RMSD values, the mutants D167N, C66R, C63Y, P59L and H30Q were found to have both increased energies after energy minimization and RMSD values, which was of critical importance and was taken into further consideration in the subsequent analysis.

#### Predicting the change in stability of the mutant proteins by I-Mutant Server

Among the 21 nsSNP’s submitted, I-Mutant predicted an increase in the stability of 4 mutants, namely rs769634543 (G135E), rs775273363 (A128V), rs8556 (S122R) and rs138213197 (G84E) (Table 8). The remaining nsSNP’s were predicted to be associated with decreased stability. Also, the four variants that were predicted to have increased stability were also found to have low RMSD values. The RMSD values and the I-Mutant results were found to be in conjunction, but the authenticity was yet to be verified by Ramachandran Plot.

#### Validation of the native and the mutant model using Ramachandran Plot

**Figure 8:**
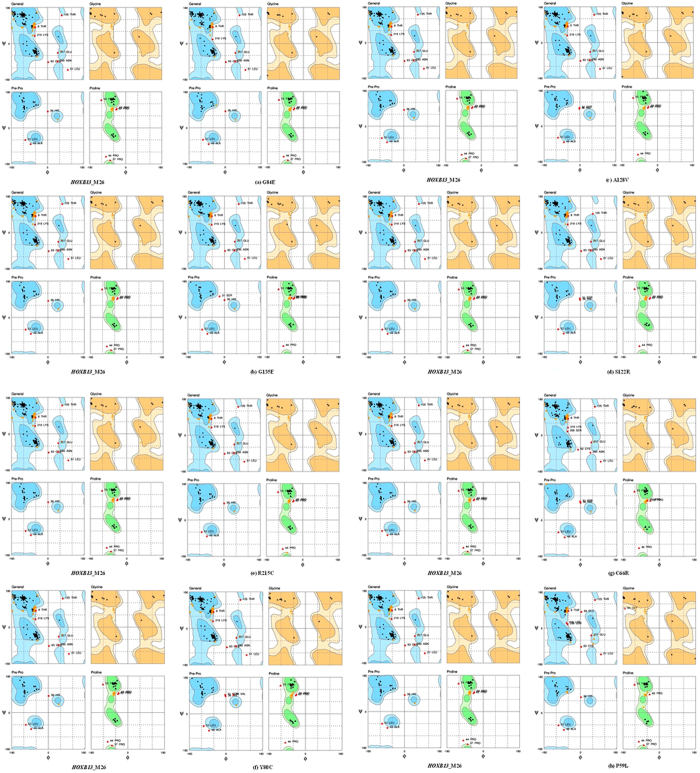
The Ramachandran plot for the native (HOXB13_M26) & mutant (HOXB13_M26 Mutant) protein. Each panel contains the general plot and the plots for Glycine and Proline residues. The native protein (HOXB13_M26) showed 217 (77.2%), 49 (17.4%) and 15 (5.3%) residues in the favored, allowed and outlier region, respectively (**a**–**c**) Ramachandran Plot for the mutants G84E (220, 46, 15 residues), G135E (222, 43, 16 residues), A128V (219, 47, 1 residues) respectively, showed an increased and stable amino acid residue pattern compared to the native structure. (**d**) The mutant S122R (217,49, 15 residues) showed the same pattern as that of the native structure. (**e**–**h**) The mutants R215C (216, 51, 14 residue), Y80C (220, 45, 16 residues), C66R (222, 43, 16 residues) and P59L (217, 48, 16 residues) showed a highly destabilizing and damaging pattern of amino acid residue distribution, respectively.

The energy minimized native (HOXB13_M26) and mutant (HOXB13_M26 Mutant) protein structures in.pdb format were submitted to RAMPAGE for assessment. The native (HOXB13_M26) contained 217 residues (77.2%) in the favored region, 49 residues (17.4%) in the allowed region and 15 residues (5.3%) in the outlier region, respectively (Fig. 8). Interestingly, the mutants G84E, G135E and A128V showed increased positive pattern when compared with the native protein. G84E had 220 (78.3%), 46 (16.4%), and 15 (5.3%) residues in the favored, allowed and outlier regions, respectively (Fig. 8). Three residues from the allowed regions were shifted to the favored region in the G84E mutant and resulted in a better pattern than the native protein. The mutant G135E also showed an increased and stable amino acid residue pattern with five residues shifting from the allowed region to the favored region with a total of 222 (79%), 43(15.3%) and 16 (5.7%) residues in the favored, allowed, and outlier regions, respectively (Fig. 8) (Table 8). The mutant A128V also showed a similar increased stabilizing pattern, where two residues from the allowed region became shifted to the favored region. The mutant A128V contained 219 (77.9%), 47 (16.7%) and 15 (5.3%) residues in the three regions, respectively (Fig. 8). The variant S122R, which was predicted to have increased stability by I-Mutant, showed no trace of increased pattern in the Ramachandran plot. It exactly resembled the native protein structure. The mutants Y80C, C66R and P59L, were found to have a destabilizing pattern of amino acid residues. Y80C had (220, 45, 16), C66R had (222, 43, 16), and P59L had (217, 48, 16) residues in the favored, allowed, and outlier regions, respectively. Interestingly, the mutants C66R and P59L were also found to have higher energy and RMSD values, whereas Y80C was reported to have a higher RMSD value. Among all the mutants, the mutant R215C were predicted to have the most destabilizing and damaging combination of amino acid residues with 216 (76.9%), 51 (18.1%), 14 (5.0%) residues in the favored, allowed, and outlier regions respectively. Four residues were shifted from the favored region to the allowed region. The mutant C63G was also found to have a similar destabilizing and damaging pattern to R215C. The remaining mutant models showed near similar or acceptable dihedral angles, which were predicted to confer less damaging effect to the protein when compared to the above-mentioned mutants.

## Discussion and Conclusion

**Figure 9:**
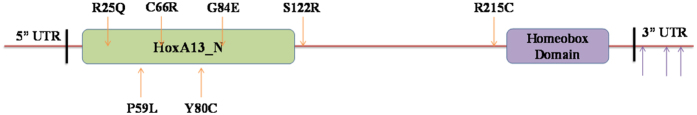
Mapping of the predicted SNPs to the *HOXB13* protein. Schematic representation depicting the location of seven missense and three 3′ UTR SNPs predicted as deleterious as a result of this study.

The thorough computational analysis of the 21nsSNP’s mapped into the HOXB13_M26 protein model, it was predicted that 7 nsSNP’s rs761914407 (R215C), rs8556 (S122R), rs138213197 (G84E), rs772962401 (Y80C), rs778843798 (C66R), rs770620686 (P59L) and rs587780165 (R25Q) were found to have seriously damaging and deleterious effects on the *HOXB13* with respect to DNA binding and function. Interestingly, the variants G84E, G135E, A128V were found to result in the increased stability of the protein structure. G84E variation was widely reported to be present in large cases of hereditary prostate cancer as epidemiologically reported elsewhere^8^,^12^,^17^,^25^,^62^,^63^,^64^,^65^ and was in agreement with the results of this study. G135E was also widely reported to be present highly among the Chinese population^66^ as reported elsewhere. Thus, the G84E, G135E and A128V variations were predicted to cause some severe structural changes in the protein, which renders it more stable with an increased half-life. It has also been reported that the gene *RFX6* transcribed and regulated by a HOXB13 transcription factor is activated and expressed over a longer period, which results in an imbalance in the feedback mechanism^5^,^67^ that is under the vigilance of *HOXB13*. It has been scientifically proven that the overexpression of *RFX6* helps in the prostate cancer cell migration and disease progression^5^,^6^,^12^,^67^. The variants that cause an increase in the stability of the *HOXB13* were found to constitutively express the downstream genes under the influence of *HOXB13* and thus it is predicted to be associated with an increase in the risk of prostate cancer, like in the case of *RFX6*^5^. The mutants R215C, Y80C, C66R and P59L, were found to have highly damaging and deleterious structural and functional properties. This in turn might disturb the role of *HOXB13* as a transcriptional factor in activating the genes responsible for cell cycle control and proliferation, eventually leading to the malignancy of the prostate. While G84E, G135E, and A128V were found to increase the stability of the protein structure, the other four nsSNP’s, R215C, C66R, Y80C, and S122R, were found to have decreased protein stability. The exact mechanism and the role of those predicted nsSNP’s with increased or decreased energy levels and protein stability should further be validated in vitro, since practically either the increased state or the decreased state might possibly involve in the altered patterns of protein structure, function and disease progression. Figure 9 shows the map of the predicted nsSNP’s. In addition, three 3′UTR variations rs543028086, rs550968159, and rs563065128, were found to affect the UNR_BS, GY-BOX and MBE UTR signal, respectively present in the 3′UTR of the *HOXB13* gene, which was predicted to result in the deregulation of the pro-apoptotic factor (Apaf-1). This altered the pattern and regulation of the translational repression of *HOXB13* via a feedback mechanism. Thus, it results in the loss of regulating the process of stem cell renewal by blocking or deregulating the cell cycle promotion inhibitors respectively, thereby causing severe damage to the *HOXB13* mediated gene expression and function. Out of the 95 nsSNP’s subjected to analysis for the variation in the miRNA patterns by the PolymiRTS database five nsSNP’s (rs8064432, rs79812861, rs148791210, rs184053751 and rs183620920) were found to disrupt only the conserved (ancestral allele with support >=2) miRNA sites. Two nsSNP’s (rs116931900 and rs1056656) were exclusively found to create a new miRNA site. Conversely, the remaining 16 nsSNP’s were predicted to be involved in the disruption and creation of a new miRNA site, out of which rs61123825 (disrupting – 2 and creating – 7) and rs192244427 (disrupting – 4 and creating 5) were found to have a maximum of 9 pattern changes. Thus, the above-mentioned nsSNP’s showed up in the progression of prostate tissue malignancy either due to the increase in the stability and half-life of the *HOXB13* encoded transcription factor or due to the damaging effects on the protein structure, which resulted in altered binding patterns of the transcription factor, thereby eventually leading to prostate tissue malignancy. The exact mechanism underlying the onset of hereditary prostate cancer by *HOXB13* nsSNP’s needs to be evaluated and studied extensively with the help of *in vivo* models and GWAS studies. This study, thus, paves the gateway for future GWAS and clinical studies related to the role of SNPs in hereditary prostate cancer and also has the potential in developing a mechanism for drug targeting and biomarkers for PCa theranostic applications.

## Materials and Methods

### Datasets

The complete list of HOXB13 SNPs, gene and protein sequences in the FASTA format were retrieved from the dbSNP database^37^,^38^ (http://www.ncbi.nlm.nih.gov/SNP/) and Ensembl genome browser (http://asia.ensembl.org/index.html).

### Prediction and Screening of deleterious nsSNP’s

The highly deleterious missense SNPs associated with the non-homeobox region of *HOXB13* gene were predicted using the following *in silico* servers: The SIFT (Sorting Intolerant From Tolerant) program (http://sift.bii.a-star.edu.sg/www/SIFT_BLink_submit.html) predicts the deleterious or damaging nature of the missense SNPs based upon sequence homology based prediction, physical properties of amino acids and also by calculating the degree of evolutionary conservation of the sequence among various species^68^. The SIFT results were reliable and the scores generated by SIFT program were classified as affecting protein structure (0.00–0.05) and as tolerated (>0.05). The PolyPhen (Polymorphism and Phenotyping) server (http://genetics.bwh.harvard.edu/pph2), screens and predicts the deleterious nsSNP’s based on the observable structural changes induced by the nsSNP’s with the help of various proven algorithms^69^,^70^ (THMM, Colis2 program, SignalP program, etc.). These structural changes are in turn known to affect the protein function and stability deleteriously. The relative solvent accessibility and secondary structure details were predicted using DSSP database. The PANTHER (Protein Analysis through Evolutionary Relationships) server (http://pantherdb.org/tools/cSNPscoreForm.jsp?), calculates the duration of a given amino acid that has been evolutionary preserved among various species and predicts the effect of that specific amino acid change on the structural and functional effect on the protein^71^. The longer the amino acid is conserved during the course of evolution, the greater the likelihood of having functional importance in protein structure and function. The PROVEAN (Protein Variation Effect Analyzer) server (http://pantherdb.org/tools/cSNPscoreForm.jsp?) relies upon the data corresponding to the standard properties of the amino acids and protein structure, thereby predicting the effect of the amino acid variations in the protein structure, stability, and function^72^. The nsSNPAnalyzer (http://snpanalyzer.uthsc.edu/) is a tool to predict the phenotypic effect of the missense SNPs based on the data from MSA and three-dimensional protein structure^73^. The PhD-SNP (Predictor of human Deleterious Single Nucleotide Polymorphisms) server (http://SNPs.biofold.org/phd-snp/phd-snp.html) functions with the help of support vector machines based (SVM-based) and evolutionary information of the sequences^20^. The nsSNP’s, which are commonly predicted by more than five servers, were taken into further consideration and analysis. The UTRscan server (http://itbtools.ba.itb.cnr.it/utrscan) is a pattern match identifier that finds the UTR pattern motif match from the protein or nucleotide sequences from the UTRsite databases using UTRblast function^45^. The PolymiRTS (Polymorphism in microRNA and their Target Sites) database (http://compbio.uthsc.edu/miRSNP/) contains comprehensive data of all the nucleotide variations occurring in the miRNA seed regions and miRNA target sites^74^. The amino acid sequence of the *HOXB13* protein (NP_006352) in FASTA format was fed to the server along with its corresponding amino acid variations (ex. G135E).

### Modelling the complete *HOXB13* protein using MODELLER v9.17

The complete *HOXB13* protein was modelled using MODELLER v9.17, which is a computer program used for comparative homology modelling of protein structures. The MODELLER v9.17 was downloaded from the Andrej Sali laboratory website (https://salilab.org/modeller/). Since MODELLER v9.17 runs on Python scripts, Python was also installed along with MODELLER v9.17^32^,^34^,^75^. The Python scripts in MODELLER v9.17 can be executed by the command “mod*9.17 script1.py*”. The basic steps involved in homology modelling using MODELLER are the initial template selection using BLAST, final template selection and alignment of the query sequence with the template structure, building the model based on the final template selected, followed by model evaluation and validation using Ramachandran Plot and PDBsum. The finally validated model waas saved as the HOXB13_M26.

### Mapping the missense amino acid variation on the protein structure

#### Template

The best model was chosen from the various models generated by MODELLER v9.17 and it was used as the template for incorporating the mutations into the protein structure and for subsequent evaluation. This template was taken as the native protein.

#### Mapping the nsSNP’s and Energy minimization of the modelled protein

Each mutant model (21 models) was generated using the “mutation” tool in SwissPDBViewer. Energy minimization of the native and the mutated protein was carried out using NOMAD-Ref Gromacs server (http://lorentz.immstr.pasteur.fr/gromacs/minimization_submission.php). The NOMAD-Ref Server utilizes Gromacs using conjugant gradient force fields for energy minimization according to the steepest descent, conjugate gradient, or L-BFGS methods. The conjugate gradient method was utilized in this study.

#### RMSD value calculation of the modelled protein

The RMSD of the atoms upon superimposing the native and the mutant protein structure was calculated using SwissPDBViewer by the “Calculate RMS” function. The extent of structural deviation between the native and the mutant protein structures was found to have an associated functional effect on the protein, which was predicted by calculating the RMSD by superimposing the native and protein structures. The higher the RMSD value, the higher the structural deviation and associated function of the protein^76^,^77^.

#### Predicting the change in stability of the mutant proteins

The stability study of the native and the mutant protein structure was crucial and was carried out with the help of the I-Mutant Server (http://folding.biofold.org/cgi-bin/i-mutant2.0.cgi). The stability of the protein and its structural changes were predicted by I-Mutant server based on calculating the relative solvent accessibility area, amino acid properties, evolutionary, and structural information of the protein^22^. The server uses the FOLD-X prediction algorithm. The input to the server was the *HOXB13* protein sequence (NP_006352), and the amino acid variations were provided manually for each variation.

#### Validation of the native and the mutant model using Ramachandran Plot and PDBsum

The Ramachandran Plot was used to calculate the dihedral angles of the amino acid residues and to predict the energetically allowed residues based upon their phi and psi dihedral angles, thereby ascertaining the structural and functional properties of the protein structure^36^,^43^,^44^. The energy minimized native and the mutant protein models were validated with the online tool RAMPAGE (http://mordred.bioc.cam.ac.uk/~rapper/rampage.php). A percentage of more than 90% residues in the favored region is required for a good protein structure. PDBsum provides the 3D protein structure information regarding the motifs, domains, helices, beta sheets and strands, angles, etc. PDBsum can be accessed online at (http://www.ebi.ac.uk/thornton-srv/databases/cgi-bin/pdbsum/GetPage.pl?pdbcode=index.html).

## Additional Information

**How to cite this article**: Chandrasekaran, G. *et al*. Computational Modeling of complete HOXB13 protein for predicting the functional effect of SNPs and the associated role in hereditary prostate cancer. *Sci. Rep.* **7**, 43830; doi: 10.1038/srep43830 (2017).

**Publisher's note:** Springer Nature remains neutral with regard to jurisdictional claims in published maps and institutional affiliations.

## Supplementary Material

Supplementary Dataset

Supplementary Data

## Figures and Tables

**Figure 1:**
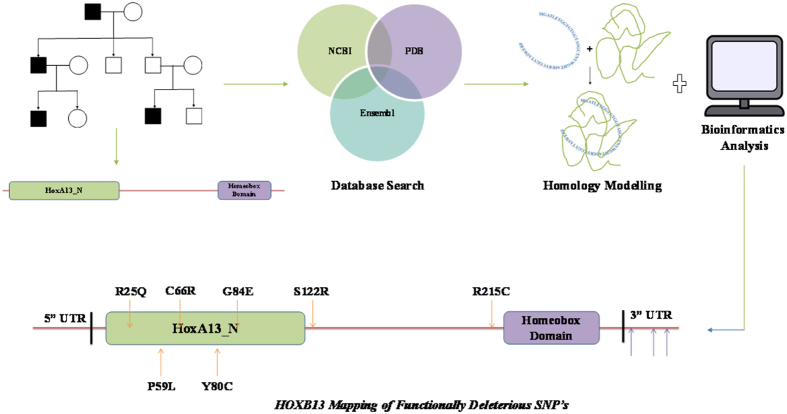
Overall scheme. Schematic representation of the overall work and the outcome of *in silico* analysis of nsSNP’s.

**Figure 2:**
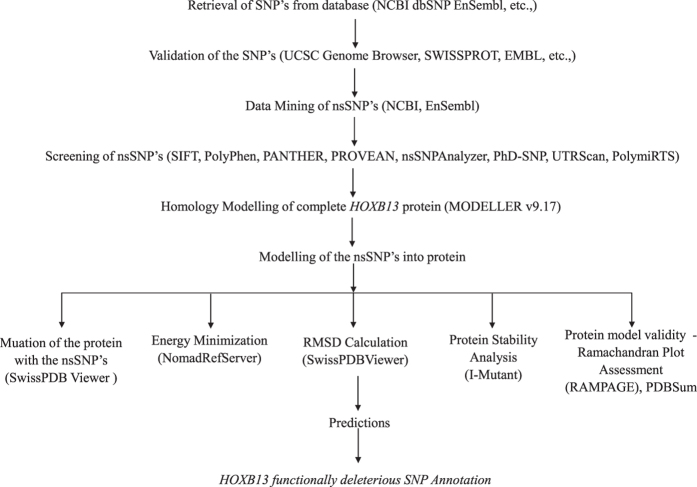
Overall workflow. Entire workflow for the *in silico* analysis of *HOXB13* non-homeobox region nsSNP’s.

**Table 1:** List of nsSNP’s predicted by SIFT as deleterious.

Variant ID	NCBI AC.No	Nucleotide Variation	AA Variation	SIFT Score	SIFT Tolerance Index
rs761914407	NP_006352	C-T	R215C	0	Affects Protein Function
rs779330626	NP_006352	A-G	Q188R	0	Affects Protein Function
rs772349818	NP_006352	T-C	M182T	0.05	Affects Protein Function
rs570681642	NP_006352	A-G	Q181R	0	Affects Protein Function
rs777986934	NP_006352	G-T	G177V	0	Affects Protein Function
rs539086211	NP_006352	G-A	G176D	0.03	Affects Protein Function
rs747003841	NP_006352	G-C	G176R	0.01	Affects Protein Function
rs587780164	NP_006352	G-A	D167N	0.01	Affects Protein Function
rs751081605	NP_006352	C-G	S162C	0.01	Affects Protein Function
rs587780163	NP_006352	C-T	A154V	0.01	Affects Protein Function
rs766929278	NP_006352	G-T	G153V	0	Affects Protein Function
rs556045007	NP_006352	C-T	A141V	0	Affects Protein Function
rs575899185	NP_006352	G-C	Q138H	0.01	Affects Protein Function
rs770891609	NP_006352	A-C	Y137S	0.01	Affects Protein Function
rs769634543	NP_006352	G-A	G135E	0.01	Affects Protein Function
rs775273363	NP_006352	C-T	A128V	0	Affects Protein Function
rs201428095	NP_006352	G-A	R123H	0.01	Affects Protein Function
rs8556	NP_006352	C-A	S122R	0	Affects Protein Function
rs760111060	NP_006352	G-A	G117E	0	Affects Protein Function
rs533641489	NP_006352	G-A	G117R	0	Affects Protein Function
rs763448911	NP_006352	C-T	A101V	0.04	Affects Protein Function
rs757433384	NP_006352	C-A	L97M	0.03	Affects Protein Function
rs138213197	NP_006352	G-A	G84E	0	Affects Protein Function
rs772962401	NP_006352	A-G	Y80C	0	Affects Protein Function
rs763353615	NP_006352	C-T	T73M	0	Affects Protein Function
rs778843798	NP_006352	T-C	C66R	0	Affects Protein Function
rs199813155	NP_006352	G-A	C63Y	0	Affects Protein Function
rs758166293	NP_006352	T-G	C63G	0.03	Affects Protein Function
rs568967699	NP_006352	A-T	K61M	0	Affects Protein Function
rs770620686	NP_006352	C-T	P59L	0.02	Affects Protein Function
rs199799743	NP_006352	C-T	T41M	0.03	Affects Protein Function
rs773491778	NP_006352	C-A	A39E	0.04	Affects Protein Function
rs561048036	NP_006352	C-G	H30Q	0	Affects Protein Function
rs587780165	NP_006352	G-A	R25Q	0.01	Affects Protein Function
rs780947625	NP_006352	G-A	G24R	0.01	Affects Protein Function
rs539706443	NP_006352	G-A	G22R	0.05	Affects Protein Function
rs772484566	NP_006352	C-T	A21V	0.01	Affects Protein Function
rs776869015	NP_006352	G-T	G4V	0	Affects Protein Function
rs546307661	NP_006352	C-A	P3T	0.02	Affects Protein Function

**Table 2:** List of nsSNP’s predicted by PolyPhen as deleterious.

Variant ID	Nucleotide Variation	AA Variation	PolyPhen-2			
			Score	Predictions	Sensitivity	Specificity
rs191886930	G-A	A276T	0.998	PD	0.27	0.99
rs761914407	C-T	R215C	1	PD	0	1
rs766169510	G-C	Q205H	0.968	PD	0.77	0.95
rs761530565	A-G	Q205R	0.538	PSD	0.88	0.91
rs750945370	G-T	A200S	0.996	PD	0.55	0.98
rs779330626	A-G	Q188R	0.985	PD	0.74	0.96
rs570681642	A-G	Q181R	0.993	PD	0.7	0.97
rs777986934	G-T	G177V	0.997	PD	0.41	0.98
rs587780164	G-A	D167N	0.958	PD	0.78	0.95
rs751081605	C-G	S162C	0.845	PD	0.83	0.93
rs766929278	G-T	G153V	1	PD	0	1
rs754280897	G-A	G153S	1	PD	0	1
rs140373548	C-A	L152M	0.998	PD	0.27	0.99
rs587780162	G-A	V146M	0.999	PD	0.14	0.99
rs556045007	C-T	A141V	0.958	PD	0.78	0.95
rs575899185	G-C	Q138H	0.998	PD	0.27	0.99
rs770891609	A-C	Y137S	0.999	PD	0.14	0.99
rs769634543	G-A	G135E	0.976	PD	0.76	0.96
rs775273363	C-T	A128V	0.999	PD	0.14	0.99
rs762666370	C-G	P124R	0.993	PD	0.7	0.97
rs201428095	G-A	R123H	1	PD	0	1
rs533641489	G-A	G117R	0.537	PSD	0.88	0.9
rs370361482	C-G	P110A	0.906	PSD	0.82	0.94
rs771674803	T-G	L106R	0.999	PD	0.14	0.99
rs764359688	C-A	P99T	0.704	PSD	0.86	0.92
rs751865027	T-A	L97Q	1	PD	0	1
rs757433384	C-A	L97M	0.999	PD	0.14	0.99
rs756135357	G-A	R94Q	1	PD	0	1
rs778563157	G-A	G85D	0.827	PSD	0.84	0.93
rs138213197	G-A	G84E	1	PD	0	1
rs772962401	A-G	Y80C	1	PD	0	1
rs763353615	C-T	T73M	0.905	PSD	0.82	0.94
rs774579054	G-T	G72V	0.905	PSD	0.82	0.94
rs750621041	C-T	P70L	0.996	PD	0.55	0.98
rs370934116	C-A	P70T	0.996	PD	0.55	0.98
rs199813155	G-A	C63Y	0.997	PD	0.41	0.98
rs758166293	T-G	C63G	0.997	PD	0.41	0.98
rs568967699	A-T	K61M	1	PD	0	1
rs770620686	C-T	P59L	0.964	PD	0.78	0.95
rs199799743	C-T	T41M	0.768	PSD	0.85	0.92
rs550726919	C-A	H36N	0.925	PSD	0.81	0.94
rs758169931	C-G	S31C	0.808	PSD	0.84	0.93
rs561048036	C-G	H30Q	0.57	PSD	0.88	0.91
rs587780165	G-A	R25Q	0.998	PD	0.27	0.99
rs539706443	G-A	G22R	0.997	PD	0.41	0.98
rs747307642	C-T	T8I	0.652	PSD	0.87	0.91
rs771173385	A-T	Y6F	0.851	PSD	0.83	0.93
**PD**	**Probably Damaging**		**PSD**		**Possibly Damaging**	

**Table 3:** List of nsSNP’s predicted by PANTHER as deleterious.

Variant ID	NCBI AC.No	Nucleotide Variation	AA Variation	PANTHER Prediction
rs761914407	NP_006352	C-T	R215C	probably damaging
rs779330626	NP_006352	A-G	Q188R	probably damaging
rs570681642	NP_006352	A-G	Q181R	probably damaging
rs777986934	NP_006352	G-T	G177V	probably damaging
rs766929278	NP_006352	G-T	G153V	possibly damaging
rs754280897	NP_006352	G-A	G153S	possibly damaging
rs140373548	NP_006352	C-A	L152M	possibly damaging
rs575899185	NP_006352	G-C	Q138H	probably damaging
rs770891609	NP_006352	A-C	Y137S	probably damaging
rs769634543	NP_006352	G-A	G135E	possibly damaging
rs775273363	NP_006352	C-T	A128V	probably damaging
rs201428095	NP_006352	G-A	R123H	probably damaging
rs8556	NP_006352	C-A	S122R	probably damaging
rs138213197	NP_006352	G-A	G84E	probably damaging
rs772962401	NP_006352	A-G	Y80C	probably damaging
rs778843798	NP_006352	T-C	C66R	probably damaging
rs199813155	NP_006352	G-A	C63Y	probably damaging
rs758166293	NP_006352	T-G	C63G	probably damaging
rs568967699	NP_006352	A-T	K61M	probably damaging
rs770620686	NP_006352	C-T	P59L	probably damaging
rs773491778	NP_006352	C-A	A39E	possibly damaging
rs760874697	NP_006352	G-T	A39S	possibly damaging
rs561048036	NP_006352	C-G	H30Q	probably damaging
rs751338230	NP_006352	A-G	H30R	probably damaging
rs587780165	NP_006352	G-A	R25Q	probably damaging

**Table 4:** List of nsSNP’s predicted by PROVEAN as deleterious.

Variant ID	NCBI AC.No	Nucleotide Variation	AA Variation	PROVEAN SCORE	PROVEAN Prediction
rs761914407	NP_006352	C-T	R215C	−6.781	Deleterious
rs779330626	NP_006352	A-G	Q188R	−3.397	Deleterious
rs772349818	NP_006352	T-C	M182T	−2.836	Deleterious
rs570681642	NP_006352	A-G	Q181R	−3.355	Deleterious
rs777986934	NP_006352	G-T	G177V	−7.026	Deleterious
rs587780164	NP_006352	G-A	D167N	−2.914	Deleterious
rs766929278	NP_006352	G-T	G153V	−4.476	Deleterious
rs575899185	NP_006352	G-C	Q138H	−2.605	Deleterious
rs770891609	NP_006352	A-C	Y137S	−7.227	Deleterious
rs769634543	NP_006352	G-A	G135E	−2.745	Deleterious
rs775273363	NP_006352	C-T	A128V	−3.159	Deleterious
rs201428095	NP_006352	G-A	R123H	−4.202	Deleterious
rs8556	NP_006352	C-A	S122R	−3.662	Deleterious
rs760111060	NP_006352	G-A	G117E	−3.229	Deleterious
rs533641489	NP_006352	G-A	G117R	−3.205	Deleterious
rs138213197	NP_006352	G-A	G84E	−6.485	Deleterious
rs772962401	NP_006352	A-G	Y80C	−6.313	Deleterious
rs778843798	NP_006352	T-C	C66R	−4.527	Deleterious
rs199813155	NP_006352	G-A	C63Y	−4.179	Deleterious
rs758166293	NP_006352	T-G	C63G	−4.117	Deleterious

**Table 5:** List of nsSNP’s predicted by nsSNPAnalyzer as deleterious.

Variant ID	Nucleotide Variation	SNP	nsSNPAnalyzer Predictions					
			Phenotype	Environ.	Area Buried	Frac. Polar	Second Str.	Scop-Link
rs761914407	C-T	R215C	Disease	P2C	0.177	0.812	C	d1ahdp_
rs779330626	A-G	Q188R	Disease	—	—	—	—	—
rs748353425	G-A	M182I	Disease	—	—	—	—	—
rs570681642	A-G	Q181R	Disease	—	—	—	—	—
rs777986934	G-T	G177V	Disease	—	—	—	—	—
rs539086211	G-A	G176D	Disease	—	—	—	—	—
rs747003841	G-C	G176R	Disease	—	—	—	—	—
rs587780164	G-A	D167N	Disease	—	—	—	—	—
rs751081605	C-G	S162C	Disease	—	—	—	—	—
rs766929278	G-T	G153V	Disease	—	—	—	—	—
rs140373548	C-A	L152M	Disease	—	—	—	—	—
rs556045007	C-T	A141V	Disease	—	—	—	—	—
rs575899185	G-C	Q138H	Disease	—	—	—	—	—
rs770891609	A-C	Y137S	Disease	—	—	—	—	—
rs769634543	G-A	G135E	Disease	—	—	—	—	—
rs775273363	C-T	A128V	Disease	—	—	—	—	—
rs762666370	C-G	P124R	Disease	—	—	—	—	—
rs201428095	G-A	R123H	Disease	—	—	—	—	—
rs8556	C-A	S122R	Disease	—	—	—	—	—
rs760111060	G-A	G117E	Disease	—	—	—	—	—
rs533641489	G-A	G117R	Disease	—	—	—	—	—
rs764401781	C-T	T115M	Disease	—	—	—	—	—
rs140492479	C-T	T105I	Disease	—	—	—	—	—
rs763448911	C-T	A101V	Disease	—	—	—	—	—
rs778563157	G-A	G85D	Disease	—	—	—	—	—
rs138213197	G-A	G84E	Disease	—	—	—	—	—
rs772962401	A-G	Y80C	Disease	—	—	—	—	—
rs763353615	C-T	T73M	Disease	—	—	—	—	—
rs774579054	G-T	G72V	Disease	—	—	—	—	—
rs750621041	C-T	P70L	Disease	—	—	—	—	—
rs370934116	C-A	P70T	Disease	—	—	—	—	—
rs766443552	G-T	V69L	Disease	—	—	—	—	—
rs778843798	T-C	C66R	Disease	—	—	—	—	—
rs199813155	G-A	C63Y	Disease	—	—	—	—	—
rs568967699	A-T	K61M	Disease	—	—	—	—	—
rs770620686	C-T	P59L	Disease	—	—	—	—	—
rs199799743	C-T	T41M	Disease	—	—	—	—	—
rs773491778	C-A	A39E	Disease	—	—	—	—	—
rs587780160	C-A	A38E	Disease	—	—	—	—	—
rs79344505	C-A	L33M	Disease	—	—	—	—	—
rs758169931	C-G	S31C	Disease	—	—	—	—	—
rs561048036	C-G	H30Q	Disease	—	—	—	—	—
rs751338230	A-G	H30R	Disease	—	—	—	—	—
rs587780165	G-A	R25Q	Disease	—	—	—	—	—
rs780947625	G-A	G24R	Disease	—	—	—	—	—
rs769323553	G-A	G23R	Disease	—	—	—	—	—
rs539706443	G-A	G22R	Disease	—	—	—	—	—
rs772484566	C-T	A21V	Disease	—	—	—	—	—
rs747307642	C-T	T8I	Disease	—	—	—	—	—
rs776869015	G-T	G4V	Disease	—	—	—	—	—
rs546307661	C-A	P3T	Disease	—	—	—	—	—

**Table 6:** List of nsSNP’s predicted by PhD-SNP server as deleterious.

Variant ID	Nucleotide Variation	AA Variation	PhD-SNP Sequence & Profile based Prediction	RI
rs761914407	C-T	R215C	Disease	7
rs779330626	A-G	Q188R	Disease	0
rs772349818	T-C	M182T	Disease	0
rs777986934	G-T	G177V	Disease	8
rs747003841	G-C	G176R	Disease	1
rs766929278	G-T	G153V	Disease	1
rs575899185	G-C	Q138H	Disease	6
rs770891609	A-C	Y137S	Disease	8
rs201428095	G-A	R123H	Disease	4
rs8556	C-A	S122R	Disease	1
rs751865027	T-A	L97Q	Disease	3
rs138213197	G-A	G84E	Disease	4
rs772962401	A-G	Y80C	Disease	7

**Table 7:** List of *HOXB13* UTR SNPs predicted to be functionally significant by UTRscan server.

Variant ID	Position	HGVS	Nucleotide Change	UTR region	UTR Signal	Functional Element Change
rs543028086	1484	c.*473 G > T	G-T	3′UTR	UNR_BS	UNR_BS - no pattern
rs550968159	1776	c.*765 C > G	C-G	3′UTR	GY-BOX	GY-BOX - no pattern
rs563065128	2241	c.*1230 A > C	A-C	3′ UTR	MBE	MBE - no pattern

**Table 8:** RMSD, Energy Minimisation, Protein Stability, Quality and Structure Assessment of HOXB13_M26 Native and Mutant Protein Structures.

Molecule	Variant ID	Nucleotide Variation	AA Variation	Total Energy after EM(KJ/mol)	RMSD (Å)	I-Mutant Results			RAMPAGE (No. of residues)		
						I-Mutant Score	RI	DDG Value (Kcal/mol)	Favored	Allowed	Outlier
HOXB13_M26	Nil	Nil	Nil	−4505.484	0	Nil	Nil	Nil	217 (77.2%)	49 (17.4%)	15 (5.3%)
HOXB13_M26 (Mutant)	rs761914407	C-T	R215C	−4273.583	0.92	Decrease	5	−0.63	216 (76.9%)	51 (18.1%)	14 (5%)
HOXB13_M26 (Mutant)	rs779330626	A-G	Q188R	−4588.476	0.83	Decrease	3	0.51	220 (78.3%)	46 (16.4%)	15 (5.3%)
HOXB13_M26 (Mutant)	rs570681642	A-G	Q181R	−4601.128	0.7	Decrease	3	0.67	219 (77.9%)	47 (16.7%)	15 (5.3%)
HOXB13_M26 (Mutant)	rs777986934	G-T	G177V	−4564.104	1.56	Decrease	3	0.31	218 (77.6%)	49 (17.4%)	14 (5%)
HOXB13_M26 (Mutant)	rs587780164	G-A	D167N	−4677.684	1.69	Decrease	7	−2.08	220 (78.3%)	46 (16.4%)	15 (5.3%)
HOXB13_M26 (Mutant)	rs766929278	G-T	G153V	−4575.739	0.8	Decrease	1	−0.53	218 (77.6%)	48 (17.1%)	15 (5.3%)
HOXB13_M26 (Mutant)	rs575899185	G-C	Q138H	−4376.856	0.71	Decrease	6	−0.97	218 (77.6%)	48 (17.1%)	15 (5.3%)
HOXB13_M26 (Mutant)	rs770891609	A-C	Y137S	−4456.023	0.72	Decrease	8	−3.36	218 (77.6%)	48 (17.1%)	15 (5.3%)
HOXB13_M26 (Mutant)	rs769634543	G-A	G135E	−4593.777	0.73	Increase	2	0.17	222 (79.0%)	43 (15.3%)	16 (5.7%)
HOXB13_M26 (Mutant)	rs775273363	C-T	A128V	−4499.878	0.77	Increase	6	0.93	219 (77.9%)	47 (16.7%)	15 (5.3%)
HOXB13_M26 (Mutant)	rs201428095	G-A	R123H	−4274.708	0.67	Decrease	6	−0.85	217 (77.2%)	50 (17.8%)	14 (5.0%)
HOXB13_M26 (Mutant)	rs8556	C-A	S122R	−4745.173	0.84	Increase	3	0.04	217 (77.2%)	49 (17.4%)	15 (5.3%)
HOXB13_M26 (Mutant)	rs138213197	G-A	G84E	−4545.865	0.57	Increase	1	0.74	220 (78.3%)	46 (16.4%)	15 (5.3%)
HOXB13_M26 (Mutant)	rs772962401	A-G	Y80C	−4451.958	1.23	Decrease	6	−0.93	220 (78.3%)	45 (16.0%)	16 (5.7%)
HOXB13_M26 (Mutant)	rs778843798	T-C	C66R	−4763.567	1.68	Decrease	3	−0.83	222 (79.0%)	43 (15.3%)	16 (5.7%)
HOXB13_M26 (Mutant)	rs199813155	G-A	C63Y	−4596.328	1.8	Decrease	0	−0.04	218 (77.6%)	48 (17.1%)	15 (5.3%)
HOXB13_M26 (Mutant)	rs758166293	T-G	C63Q	−4663.925	1.08	Decrease	4	−0.97	219 (77.9%)	50 (17.8%)	12 (4.3%)
HOXB13_M26 (Mutant)	rs568967699	A-T	K61M	−4516.539	0.71	Decrease	0	−0.77	219 (77.9%)	47 (16.7%)	15 (5.3%)
HOXB13_M26 (Mutant)	rs770620686	C-T	P59L	−4024.038	1.8	Decrease	5	−0.39	217 (77.2%)	48 (17.1%)	16 (5.7%)
HOXB13_M26 (Mutant)	rs561048036	C-G	H30Q	−4675.44	2.02	Decrease	5	−0.72	222 (79.0%)	45 (16.0%)	14 (5.0%)
HOXB13_M26 (Mutant)	rs587780165	G-A	R25Q	−4440.227	1.52	Decrease	5	−0.21	223 (79.4%)	43 (15.3%)	15 (5.3%)
