# Supplementary material for: Computational Modeling of complete HOXB13 protein for predicting the functional effect of SNPs and the associated role in hereditary prostate cancer
Source: Sci Rep. 2017 Mar 8;7:43830. doi: 10.1038/srep43830 (PMC5363706; doi:10.1038/srep43830)
Supplement: Supplementary Dataset [file srep43830-s1.zip › SR Supplementary Data Files Complete/IMutant/Q138H.pdf]

I-Mutant2.0

I-Mutant2.0 Home

Biocomputing Unit

Contact us

Last Update 27/12/06

```
***************************************************************************
**                                                                       **
**                             I-Mutant v2.0                             **
**         Predictor of Protein Stability Changes upon Mutations         **
**                                                                       **
***************************************************************************


      SEQ File: fileseq.txt

      Position   WT  NEW   Stability  RI    pH    T
           138    Q    H    Decrease   6   7.0   25


      WT:  Aminoacid in Wild-Type Protein
      NEW: New Aminoacid after Mutation
      RI:  Reliability Index
      T:   Temperature in Celsius degrees
      pH:  -log[H+]



***************************************************************************
*                                                                         *
* Capriotti E, Fariselli P and Casadio R (2005). I-Mutant2.0: predicting  *
* stability changes upon mutation from the protein sequence or structure. *
* Nucl. Acids Res. 33: W306-W310.                                         *
* http://gpcr.biocomp.unibo.it/cgi/predictors/I-Mutant2.0/I-Mutant2.0.cgi *
*                                                                         *
***************************************************************************
```
