# Supplementary material for: Computational Modeling of complete HOXB13 protein for predicting the functional effect of SNPs and the associated role in hereditary prostate cancer
Source: Sci Rep. 2017 Mar 8;7:43830. doi: 10.1038/srep43830 (PMC5363706; doi:10.1038/srep43830)
Supplement: Supplementary Dataset [file srep43830-s1.zip › SR Supplementary Data Files Complete/IMutant/R215C.pdf]

I-Mutant2.0

I-Mutant2.0 Home

Biocomputing Unit

Contact us

Last Update 27/12/06

```
******************************************************************************
**                                                                          **
**                              I-Mutant v2.0                               **
**          Predictor of Protein Stability Changes upon Mutations           **
**                                                                          **
******************************************************************************


      SEQ File: fileseq.txt

      Position   WT  NEW   Stability  RI    pH    T
           215    R    C    Decrease   5   7.0   25


      WT:  Aminoacid in Wild-Type Protein
      NEW: New Aminoacid after Mutation
      RI:  Reliability Index
      T:   Temperature in Celsius degrees
      pH:  -log[H+]



******************************************************************************
*                                                                            *
* Capriotti E, Fariselli P and Casadio R (2005). I-Mutant2.0: predicting     *
* stability changes upon mutation from the protein sequence or structure.   *
* Nucl. Acids Res. 33: W306-W310.                                            *
* http://gpcr.biocomp.unibo.it/cgi/predictors/I-Mutant2.0/I-Mutant2.0.cgi   *
*                                                                            *
******************************************************************************
```
