# Supplementary material for: Computational Modeling of complete HOXB13 protein for predicting the functional effect of SNPs and the associated role in hereditary prostate cancer
Source: Sci Rep. 2017 Mar 8;7:43830. doi: 10.1038/srep43830 (PMC5363706; doi:10.1038/srep43830)
Supplement: Supplementary Data [file srep43830-s2.pdf]

Computational Modeling of complete HOXB13 protein for predicting the functional effect of SNP's and its associated role in hereditary prostate cancer

Gopalakrishnan Chandrasekaran[1], Eu Chang Hwang[2], Taek Won Kang[2] Dongdeuk Kwon[2], Kwangsung Park[2], Je-Jung Lee[3], and Vinoth-Kumar Lakshmanan[1*]

[1]Department of Biomedical Sciences, Chonnam National University Medical School,160 Baeksuh-Roh, Dong-Gu, Gwangju 61469, Republic of Korea
[2] Department of Urology, Chonnam National University Hwasun Hospital, Jeollanamdo, Republic of Korea
[3]Department of Research Center for Cancer Immunotherapy, Chonnam National University Hwasun Hospital, Jeollanamdo, Republic of Korea.

[*]Corresponding author: Prof. Vinoth Kumar Lakshmanan – vinoth.lakshmanan@gmail.com/vinoth@jnu.ac.kr

[*]Corresponding author

Vinoth-Kumar Lakshmanan Ph.D.
Associate Professor
Department of Biomedical Sciences
Chonnam National University Medical School
160 Baeksuh-Roh, Dong-Gu
Gwangju, Korea (ROK) 61469
Telephone: 061-379-2653
Fax: 061-379-2680
Mobile: 010-4341-7333
Email: vinoth.lakshmanan@gmail.com, vinoth@jnu.ac.kr
Website: www.jnu.ac.kr

Supplementary Data

Methods

Homology Modelling using MODELLER v9.1

Comparative modelling involves four main steps[34] (see Supplementary Fig. S2 online), which are as follows:

a. Template screening, identification and initial alignment – The protein template is selected from the PDB with the help of psiBLAST.
b. Final template selection and alignment – The final template is selected among all available templates based upon the % identity and E-value.
c. Model Building – The model is built by aligning the query sequence (NP_006352.2) against the template structures.
d. Model Evaluation and Selection – The generated models are evaluated for the low DOPE score ad high GA341 Score to prove it as the best model.
e. Model Validation – The evaluated and the selected model are validated for their features and properties with the help of the Ramachandran Plot[36] and PDBsum.

Sequence format conversion

The *HOXB13* protein sequence (NP_006352) obtained from NCBI needs to be first converted to a file format, which can be read and processed by MODELLER v9.1. The sequence was converted to a PIR file format. Similar to the FASTA format the PIR file format also consists of a header denoted by >P1 followed by the sequence identifier and details. The PIR file format contains the single letter standard amino acid codes with " ***** " in the end denoting the end of the protein sequence. The converted sequence in the PIR format was saved as "hoxb13.ali".

Target Template identification and initial alignment

A suitable template structure for developing the model was obtained using psiBLAST by setting PDB as the source database[66] for finding 3D structure templates. The resulting sequences of at least >30% similarity and identity were picked for comparative homology modelling. The Supplementary Table S8 online shows the results of psiBLAST. PDB ID 2CRA was found to have 100% identity with the query sequence and PDB ID 2LD5 and 2L7Z showed 78% identity with the query sequence and were chosen as the template for modelling the *HOXB13* protein. The respective ".pdb" files of the above-mentioned proteins were downloaded and kept in the same folder where the python script files were located. These three PDB ID structures – 2CRA,

2LD5 and 2L7Z were used as the template for modelling the complete *HOXB13* protein using comparative homology modelling by MODELLER v9.17. The distance tree of the query sequence and the protein structures from the PDB computed by psiBLAST can be found as Supplementary Fig. S4 online.

Final template selection

From the three chosen PDB structures (2CRA, 2LD5 and 2L7Z), the most appropriate structure for the query sequence (NP_006352 – *HOXB13*) was chosen by the "alignment.compare_structures()" command from MODELLER v9.17 to analyze the structural and sequence similarity between the template structures (2CRA, 2LD5 and 2L7Z) and the query sequence (NP_006352). The Python script for the final template selection was saved as "script2.py". The contents of the "script2.py" were given in the supplementary data. The "compare_structure" command mainly compares the RMS deviation, dihedral angles of the main and side chain atoms, identities, similarities, etc., between the sequence and the structure. We used this command and to generate the "dendrogram" with distance matrix for analyzing and selecting the best template among the three targets. The results were logged in the "script2.log" file. From the results, we found that 2LD5 and 2L7Z were both structurally and sequentially identical with the same crystallographic resolution of 1.0Å (as Supplementary Fig. S5 online). Conversely, the structure 2CRA was found to be diversified from both 2LD5 and 2L7Z with a distance score of 63.5. Hence, the structure 2L7Z was finally selected for modelling of complete *HOXB13* because of its high sequence and structural similarity to the query sequence.

Aligning the template 2L7Z with the query sequence (NP_006352)

The alignment of the query sequence (NP_006352) with the template structure 2L7Z was done with the help of the "algin2d()" command in MODELLER v9.17. The alignment algorithm used by the MODELLER v9.17 at this step was different from the other normal sequence alignment algorithms, since, in this case, alignment was being carried out between the sequence and the structure information. This was done with the help of the gap penalty function so that there was a greater reduction in the alignment errors when compared to other standard sequence-sequence alignment algorithms. The Python script for the alignment was saved as "script3.py'". The detailed contents of the "script3.py" were furnished in the supplementary data section. The "align2d()" command aligned the query sequence saved in PIR format as "hoxb13.ali" file with the template protein structure file saved as "2l7z.pdb".

The results were logged in the "script3.log" file, and the alignment was generated as "hoxb13-2l7z.pap" and "hoxb13-2l7z.ali" files. All the identical positions were marked with " ***** ". The output of the alignment between the query sequence and the template structure can be found as Supplementary Fig. S6 online.

Building of the complete *HOXB13* model

With the help of the aligned query sequence (NP_006352) and the template structure (2L7Z) data stored in the "hoxb13-2l7z.ali" file, MODELLER v9.17 calculated and predicted the 3D model of the complete *HOXB13* protein automatically using its default "automodel" class command. MODELLER v9.17 was instructed to generate 30 similar models of complete *HOXB13* protein based on the 2L7Z template structure and "hox13-2l7z.ali" file. The Python script for the model building was saved as "script4.py". The complete "script4" s given in the supplementary data.

The results of the model building script were stored in the "script4.log" file. MODELLER v9.17 calculated, set the parameters, and computed to build the model automatically. Since we instructed the program to generate 30 similar models, MODELLER v9.17 generated 30 similar models with a ".pdb" file and corresponding log files. The results of the script4 are given in Figure S7. MODELLER v9.17 successfully constructed 30 similar complete *HOXB13* models with their corresponding molpdf values, DOPE score and GA341 score, which were helpful in choosing the best model out of the 30 models generated. The entire results of script 4 can be found in Supplementary Fig. S7 online.

Evaluating the generated *HOXB13* models and selecting the best model

There are several criteria to select the best model among the various models generated by MODELLER v9.17. The most important and widely practised criteria includes selecting the model with the lowest DOPE score[67] and the highest GA341 score[68]. Accordingly, from the summary of the models generated (Figure S7.), we found that the 26th model "hoxb13.B99990026.pdb" had the lowest DOPE score of -10661.23047 and the highest GA341 score of 1.00000. Thus, the selected model "hoxb13.B99990026.pdb" was subjected to further validation of protein structure and folding properties with the help of the Ramachandran Plot and PDBSum.

Supplementary Tables

Table S1. List of nsSNP's predicted by SIFT as deleterious					
**Variant ID**	**NCBI AC. No**	**Nucleotide Variation**	**AA Variation**	**SIFT Score**	**SIFT Tolerance Index**
**rs752722523**	NP_006352	G-A	A282T	0.65	Tolerated
**rs763960333**	NP_006352	G-C	S281T	0.51	Tolerated
**rs200997384**	NP_006352	G-T	V278L	0.74	Tolerated
**rs191886930**	NP_006352	G-A	A276T	0.19	Tolerated
**rs761914407**	NP_006352	C-T	R215C	0	Affects Protein Function
**rs145059285**	NP_006352	G-A	A212T	0.32	Tolerated
**rs758190709**	NP_006352	C-T	A210V	0.29	Tolerated
**rs766169510**	NP_006352	G-C	Q205H	0.43	Tolerated
**rs761530565**	NP_006352	A-G	Q205R	0.87	Tolerated
**rs750945370**	NP_006352	G-T	A200S	0.23	Tolerated
**rs756654236**	NP_006352	G-A	A197T	0.16	Tolerated
**rs780355420**	NP_006352	T-A	F194I	0.13	Tolerated
**rs779330626**	NP_006352	A-G	Q188R	0	Affects Protein Function
**rs748353425**	NP_006352	G-A	M182I	0.16	Tolerated
**rs772349818**	NP_006352	T-C	M182T	0.05	Affects Protein Function
**rs570681642**	NP_006352	A-G	Q181R	0	Affects Protein Function
**rs777986934**	NP_006352	G-T	G177V	0	Affects Protein Function
**rs539086211**	NP_006352	G-A	G176D	0.03	Affects Protein Function
**rs747003841**	NP_006352	G-C	G176R	0.01	Affects Protein Function
**rs148649390**	NP_006352	G-T	A175S	0.86	Tolerated
**rs587780164**	NP_006352	G-A	D167N	0.01	Affects Protein Function
**rs751081605**	NP_006352	C-G	S162C	0.01	Affects Protein Function
**rs587780163**	NP_006352	C-T	A154V	0.01	Affects Protein Function
**rs766929278**	NP_006352	G-T	G153V	0	Affects Protein Function
**rs754280897**	NP_006352	G-A	G153S	0.45	Tolerated
**rs140373548**	NP_006352	C-A	L152M	0.08	Tolerated
**rs587780162**	NP_006352	G-A	V146M	0.52	Tolerated
**rs556045007**	NP_006352	C-T	A141V	0	Affects Protein Function
**rs575899185**	NP_006352	G-C	Q138H	0.01	Affects Protein Function
**rs770891609**	NP_006352	A-C	Y137S	0.01	Affects Protein Function
**rs745800027**	NP_006352	A-C	T136P	1	Tolerated
**rs769634543**	NP_006352	G-A	G135E	0.01	Affects Protein Function
**rs775273363**	NP_006352	C-T	A128V	0	Affects Protein Function

**rs762666370**	NP_006352	C-G	P124R	0.19	Tolerated
**rs201428095**	NP_006352	G-A	R123H	0.01	Affects Protein Function
**rs8556**	NP_006352	C-A	S122R	0	Affects Protein Function
**rs766909225**	NP_006352	C-G	P121A	0.09	Tolerated
**rs760111060**	NP_006352	G-A	G117E	0	Affects Protein Function
**rs533641489**	NP_006352	G-A	G117R	0	Affects Protein Function
**rs764401781**	NP_006352	C-T	T115M	0.06	Tolerated
**rs751698154**	NP_006352	C-A	P114T	0.56	Tolerated
**rs781117900**	NP_006352	C-A	A111E	0.16	Tolerated
**rs745889927**	NP_006352	G-A	A111T	0.46	Tolerated
**rs370361482**	NP_006352	C-G	P110A	1	Tolerated
**rs774095240**	NP_006352	C-T	A108V	0.09	Tolerated
**rs747755369**	NP_006352	G-C	A107P	0.34	Tolerated
**rs771674803**	NP_006352	T-G	L106R	0.32	Tolerated
**rs140492479**	NP_006352	C-T	T105I	0.34	Tolerated
**rs760196832**	NP_006352	C-T	A103V	0.1	Tolerated
**rs763448911**	NP_006352	C-T	A101V	0.04	Affects Protein Function
**rs764359688**	NP_006352	C-A	P99T	0.32	Tolerated
**rs751865027**	NP_006352	T-A	L97Q	0.19	Tolerated
**rs757433384**	NP_006352	C-A	L97M	0.03	Affects Protein Function
**rs756135357**	NP_006352	G-A	R94Q	0.1	Tolerated
**rs780012601**	NP_006352	T-G	S93A	0.33	Tolerated
**rs778563157**	NP_006352	G-A	G85D	0.27	Tolerated
**rs529392210**	NP_006352	G-A	G85S	0.91	Tolerated
**rs138213197**	NP_006352	G-A	G84E	0	Affects Protein Function
**rs772962401**	NP_006352	A-G	Y80C	0	Affects Protein Function
**rs746542615**	NP_006352	G-T	V78L	1	Tolerated
**rs763353615**	NP_006352	C-T	T73M	0	Affects Protein Function
**rs774579054**	NP_006352	G-T	G72V	0.05	Tolerated
**rs750621041**	NP_006352	C-T	P70L	0.15	Tolerated
**rs370934116**	NP_006352	C-A	P70T	0.61	Tolerated
**rs766443552**	NP_006352	G-T	V69L	0.21	Tolerated
**rs587780161**	NP_006352	C-T	P67S	0.85	Tolerated
**rs778843798**	NP_006352	T-C	C66R	0	Affects Protein Function
**rs199813155**	NP_006352	G-A	C63Y	0	Affects Protein Function
**rs758166293**	NP_006352	T-G	C63G	0.03	Affects Protein Function
**rs568967699**	NP_006352	A-T	K61M	0	Affects Protein Function
**rs770620686**	NP_006352	C-T	P59L	0.02	Affects Protein Function
**rs745306184**	NP_006352	G-T	A57S	0.58	Tolerated
**rs769074510**	NP_006352	C-T	P54S	0.54	Tolerated

**rs11551892**	NP_006352	G-C	A49P	0.33	Tolerated
**rs748333323**	NP_006352	A-G	N47S	1	Tolerated
**rs199799743**	NP_006352	C-T	T41M	0.03	Affects Protein Function
**rs773491778**	NP_006352	C-A	A39E	0.04	Affects Protein Function
**rs760874697**	NP_006352	G-T	A39S	0.98	Tolerated
**rs587780160**	NP_006352	C-A	A38E	0.05	Tolerated
**rs550726919**	NP_006352	C-A	H36N	0.22	Tolerated
**rs79344505**	NP_006352	C-A	L33M	0.04	Tolerated
**rs758169931**	NP_006352	C-G	S31C	0.18	Tolerated
**rs561048036**	NP_006352	C-G	H30Q	0	Affects Protein Function
**rs751338230**	NP_006352	A-G	H30R	0.15	Tolerated
**rs756970136**	NP_006352	G-T	A29S	0.47	Tolerated
**rs587780165**	NP_006352	G-A	R25Q	0.01	Affects Protein Function
**rs780947625**	NP_006352	G-A	G24R	0.01	Affects Protein Function
**rs745392080**	NP_006352	G-A	G23E	0.3	Tolerated
**rs769323553**	NP_006352	G-A	G23R	0.37	Tolerated
**rs539706443**	NP_006352	G-A	G22R	0.05	Affects Protein Function
**rs772484566**	NP_006352	C-T	A21V	0.01	Affects Protein Function
**rs747307642**	NP_006352	C-T	T8I	0.18	Tolerated
**rs771173385**	NP_006352	A-T	Y6F	0.61	Tolerated
**rs776869015**	NP_006352	G-T	G4V	0	Affects Protein Function
**rs546307661**	NP_006352	C-A	P3T	0.02	Affects Protein Function

Table S2. List of nsSNP's predicted by PolyPhen as deleterious						
**Variant ID**	**Nucleotide Variation**	**AA Variation**	**PolyPhen-2**			
			**Score**	**Predictions**	**Sensitivity**	**Specificity**
**rs752722523**	G-A	A282T	0	Benign	1	0
**rs763960333**	G-C	S281T	0	Benign	1	0
**rs200997384**	G-T	V278L	0.017	Benign	0.95	0.8
**rs191886930**	G-A	A276T	0.998	PD	0.27	0.99
**rs761914407**	C-T	R215C	1	PD	0	1
**rs145059285**	G-A	A212T	0	Benign	1	0
**rs758190709**	C-T	A210V	0.011	Benign	0.96	0.78
**rs766169510**	G-C	Q205H	0.968	PD	0.77	0.95
**rs761530565**	A-G	Q205R	0.538	PSD	0.88	0.91
**rs750945370**	G-T	A200S	0.996	PD	0.55	0.98
**rs756654236**	G-A	A197T	0.001	Benign	0.99	0.15
**rs780355420**	T-A	F194I	0.329	Benign	0.9	0.89
**rs779330626**	A-G	Q188R	0.985	PD	0.74	0.96
**rs748353425**	G-A	M182I	0.002	Benign	0.99	0.3
**rs772349818**	T-C	M182T	0.012	Benign	0.96	0.78
**rs570681642**	A-G	Q181R	0.993	PD	0.7	0.97
**rs777986934**	G-T	G177V	0.997	PD	0.41	0.98
**rs539086211**	G-A	G176D	0.061	Benign	0.94	0.84
**rs747003841**	G-C	G176R	0.314	Benign	0.9	0.89
**rs148649390**	G-T	A175S	0.006	Benign	0.97	0.75
**rs587780164**	G-A	D167N	0.958	PD	0.78	0.95
**rs751081605**	C-G	S162C	0.845	PD	0.83	0.93
**rs587780163**	C-T	A154V	0.03	Benign	0.95	0.82
**rs766929278**	G-T	G153V	1	PD	0	1
**rs754280897**	G-A	G153S	1	PD	0	1
**rs140373548**	C-A	L152M	0.998	PD	0.27	0.99
**rs587780162**	G-A	V146M	0.999	PD	0.14	0.99
**rs556045007**	C-T	A141V	0.958	PD	0.78	0.95
**rs575899185**	G-C	Q138H	0.998	PD	0.27	0.99
**rs770891609**	A-C	Y137S	0.999	PD	0.14	0.99
**rs745800027**	A-C	T136P	0	Benign	1	0
**rs769634543**	G-A	G135E	0.976	PD	0.76	0.96
**rs775273363**	C-T	A128V	0.999	PD	0.14	0.99
**rs762666370**	C-G	P124R	0.993	PD	0.7	0.97
**rs201428095**	G-A	R123H	1	PD	0	1

**rs8556**	C-A	S122R	0.044	Benign	0.94	0.83
**rs766909225**	C-G	P121A	0.005	Benign	0.97	0.74
**rs760111060**	G-A	G117E	0.055	Benign	0.94	0.84
**rs533641489**	G-A	G117R	0.537	PSD	0.88	0.9
**rs764401781**	C-T	T115M	0.404	Benign	0.89	0.9
**rs751698154**	C-A	P114T	0.001	Benign	0.99	0.15
**rs781117900**	C-A	A111E	0.03	Benign	0.95	0.82
**rs745889927**	G-A	A111T	0	Benign	1	0
**rs370361482**	C-G	P110A	0.906	PSD	0.82	0.94
**rs774095240**	C-T	A108V	0.412	Benign	0.92	0.86
**rs747755369**	G-C	A107P	0	Benign	1	0
**rs771674803**	T-G	L106R	0.999	PD	0.14	0.99
**rs140492479**	C-T	T105I	0.049	Benign	0.94	0.83
**rs760196832**	C-T	A103V	0.052	Benign	0.94	0.83
**rs763448911**	C-T	A101V	0.201	Benign	0.92	0.88
**rs764359688**	C-A	P99T	0.704	PSD	0.86	0.92
**rs751865027**	T-A	L97Q	1	PD	0	1
**rs757433384**	C-A	L97M	0.999	PD	0.14	0.99
**rs756135357**	G-A	R94Q	1	PD	0	1
**rs780012601**	T-G	S93A	0	Benign	1	0
**rs778563157**	G-A	G85D	0.827	PSD	0.84	0.93
**rs529392210**	G-A	G85S	0.271	Benign	0.91	0.88
**rs138213197**	G-A	G84E	1	PD	0	1
**rs772962401**	A-G	Y80C	1	PD	0	1
**rs746542615**	G-T	V78L	0.005	Benign	0.97	0.74
**rs763353615**	C-T	T73M	0.905	PSD	0.82	0.94
**rs774579054**	G-T	G72V	0.905	PSD	0.82	0.94
**rs750621041**	C-T	P70L	0.996	PD	0.55	0.98
**rs370934116**	C-A	P70T	0.996	PD	0.55	0.98
**rs766443552**	G-T	V69L	0	Benign	1	0
**rs587780161**	C-T	P67S	0.001	Benign	0.99	0.15
**rs778843798**	T-C	C66R	0.278	Benign	0.91	0.88
**rs199813155**	G-A	C63Y	0.997	PD	0.41	0.98
**rs758166293**	T-G	C63G	0.997	PD	0.41	0.98
**rs568967699**	A-T	K61M	1	PD	0	1
**rs770620686**	C-T	P59L	0.964	PD	0.78	0.95
**rs745306184**	G-T	A57S	0.002	Benign	0.99	0.3
**rs769074510**	C-T	P54S	0.032	Benign	0.95	0.82
**rs11551892**	G-C	A49P	0	Benign	1	0
**rs748333323**	A-G	N47S	0	Benign	1	0

**rs199799743**	C-T	T41M	0.768	PSD	0.85	0.92
**rs773491778**	C-A	A39E	0.116	Benign	0.93	0.86
**rs760874697**	G-T	A39S	0.001	Benign	0.99	0.15
**rs587780160**	C-A	A38E	0.061	Benign	0.94	0.84
**rs550726919**	C-A	H36N	0.925	PSD	0.81	0.94
**rs79344505**	C-A	L33M	0.037	Benign	0.94	0.82
**rs758169931**	C-G	S31C	0.808	PSD	0.84	0.93
**rs561048036**	C-G	H30Q	0.57	PSD	0.88	0.91
**rs751338230**	A-G	H30R	0.274	Benign	0.91	0.88
**rs756970136**	G-T	A29S	0.002	Benign	0.99	0.3
**rs587780165**	G-A	R25Q	0.998	PD	0.27	0.99
**rs780947625**	G-A	G24R	0.074	Benign	0.93	0.84
**rs745392080**	G-A	G23E	0.085	Benign	0.93	0.85
**rs769323553**	G-A	G23R	0.157	Benign	0.92	0.87
**rs539706443**	G-A	G22R	0.997	PD	0.41	0.98
**rs772484566**	C-T	A21V	0.001	Benign	0.99	0.15
**rs747307642**	C-T	T8I	0.652	PSD	0.87	0.91
**rs771173385**	A-T	Y6F	0.851	PSD	0.83	0.93
**rs776869015**	G-T	G4V	0	Benign	1	0
**rs546307661**	C-A	P3T	0.099	Benign	0.93	0.85
**PD**	**Probably Damaging**		**PSD**		**Possibly Damaging**	

Table S3. List of nsSNP's predicted by PANTHER as deleterious				
**Variant ID**	**NCBI AC. No**	**Nucleotide Variation**	**AA Variation**	**PANTHER Prediction**
**rs752722523**	NP_006352	G-A	A282T	probably begign
**rs763960333**	NP_006352	G-C	S281T	probably begign
**rs200997384**	NP_006352	G-T	V278L	probably begign
**rs191886930**	NP_006352	G-A	A276T	probably begign
**rs761914407**	NP_006352	C-T	R215C	probably damaging
**rs145059285**	NP_006352	G-A	A212T	probably begign
**rs758190709**	NP_006352	C-T	A210V	probably begign
**rs766169510**	NP_006352	G-C	Q205H	probably begign
**rs761530565**	NP_006352	A-G	Q205R	probably begign
**rs750945370**	NP_006352	G-T	A200S	probably begign
**rs756654236**	NP_006352	G-A	A197T	probably begign
**rs780355420**	NP_006352	T-A	F194I	probably begign
**rs779330626**	NP_006352	A-G	Q188R	probably damaging
**rs748353425**	NP_006352	G-A	M182I	probably begign
**rs772349818**	NP_006352	T-C	M182T	probably begign
**rs570681642**	NP_006352	A-G	Q181R	probably damaging
**rs777986934**	NP_006352	G-T	G177V	probably damaging
**rs539086211**	NP_006352	G-A	G176D	probably begign
**rs747003841**	NP_006352	G-C	G176R	probably begign
**rs148649390**	NP_006352	G-T	A175S	probably begign
**rs587780164**	NP_006352	G-A	D167N	probably begign
**rs751081605**	NP_006352	C-G	S162C	probably begign
**rs587780163**	NP_006352	C-T	A154V	probably begign
**rs766929278**	NP_006352	G-T	G153V	possibly damaging
**rs754280897**	NP_006352	G-A	G153S	possibly damaging
**rs140373548**	NP_006352	C-A	L152M	possibly damaging
**rs587780162**	NP_006352	G-A	V146M	probably begign
**rs556045007**	NP_006352	C-T	A141V	probably begign
**rs575899185**	NP_006352	G-C	Q138H	probably damaging
**rs770891609**	NP_006352	A-C	Y137S	probably damaging
**rs745800027**	NP_006352	A-C	T136P	probably begign
**rs769634543**	NP_006352	G-A	G135E	possibly damaging
**rs775273363**	NP_006352	C-T	A128V	probably damaging
**rs762666370**	NP_006352	C-G	P124R	probably begign
**rs201428095**	NP_006352	G-A	R123H	probably damaging
**rs8556**	NP_006352	C-A	S122R	probably damaging
**rs766909225**	NP_006352	C-G	P121A	probably begign

**rs760111060**	NP_006352	G-A	G117E	probably begign
**rs533641489**	NP_006352	G-A	G117R	probably begign
**rs764401781**	NP_006352	C-T	T115M	probably begign
**rs751698154**	NP_006352	C-A	P114T	probably begign
**rs781117900**	NP_006352	C-A	A111E	probably begign
**rs745889927**	NP_006352	G-A	A111T	probably begign
**rs370361482**	NP_006352	C-G	P110A	probably begign
**rs774095240**	NP_006352	C-T	A108V	probably begign
**rs747755369**	NP_006352	G-C	A107P	probably begign
**rs771674803**	NP_006352	T-G	L106R	probably begign
**rs140492479**	NP_006352	C-T	T105I	probably begign
**rs760196832**	NP_006352	C-T	A103V	probably begign
**rs763448911**	NP_006352	C-T	A101V	probably begign
**rs764359688**	NP_006352	C-A	P99T	probably begign
**rs751865027**	NP_006352	T-A	L97Q	probably begign
**rs757433384**	NP_006352	C-A	L97M	probably begign
**rs756135357**	NP_006352	G-A	R94Q	probably begign
**rs780012601**	NP_006352	T-G	S93A	probably begign
**rs778563157**	NP_006352	G-A	G85D	probably begign
**rs529392210**	NP_006352	G-A	G85S	probably begign
**rs138213197**	NP_006352	G-A	G84E	probably damaging
**rs772962401**	NP_006352	A-G	Y80C	probably damaging
**rs746542615**	NP_006352	G-T	V78L	probably begign
**rs763353615**	NP_006352	C-T	T73M	probably begign
**rs774579054**	NP_006352	G-T	G72V	probably begign
**rs750621041**	NP_006352	C-T	P70L	probably begign
**rs370934116**	NP_006352	C-A	P70T	probably begign
**rs766443552**	NP_006352	G-T	V69L	probably begign
**rs587780161**	NP_006352	C-T	P67S	probably begign
**rs778843798**	NP_006352	T-C	C66R	probably damaging
**rs199813155**	NP_006352	G-A	C63Y	probably damaging
**rs758166293**	NP_006352	T-G	C63G	probably damaging
**rs568967699**	NP_006352	A-T	K61M	probably damaging
**rs770620686**	NP_006352	C-T	P59L	probably damaging
**rs745306184**	NP_006352	G-T	A57S	probably begign
**rs769074510**	NP_006352	C-T	P54S	probably begign
**rs11551892**	NP_006352	G-C	A49P	probably begign
**rs748333323**	NP_006352	A-G	N47S	probably begign
**rs199799743**	NP_006352	C-T	T41M	probably begign
**rs773491778**	NP_006352	C-A	A39E	possibly damaging

**rs760874697**	NP_006352	G-T	A39S	possibly damaging
**rs587780160**	NP_006352	C-A	A38E	probably begign
**rs550726919**	NP_006352	C-A	H36N	probably begign
**rs79344505**	NP_006352	C-A	L33M	probably begign
**rs758169931**	NP_006352	C-G	S31C	probably begign
**rs561048036**	NP_006352	C-G	H30Q	probably damaging
**rs751338230**	NP_006352	A-G	H30R	probably damaging
**rs756970136**	NP_006352	G-T	A29S	probably begign
**rs587780165**	NP_006352	G-A	R25Q	probably damaging
**rs780947625**	NP_006352	G-A	G24R	probably begign
**rs745392080**	NP_006352	G-A	G23E	probably begign
**rs769323553**	NP_006352	G-A	G23R	probably begign
**rs539706443**	NP_006352	G-A	G22R	probably begign
**rs772484566**	NP_006352	C-T	A21V	probably begign
**rs747307642**	NP_006352	C-T	T8I	probably begign
**rs771173385**	NP_006352	A-T	Y6F	probably begign
**rs776869015**	NP_006352	G-T	G4V	probably begign
**rs546307661**	NP_006352	C-A	P3T	probably begign

Table S4. List of nsSNP's predicted by PROVEAN as deleterious					
**Variant ID**	**NCBI AC. No**	**Nucleotide Variation**	**AA Variation**	**PROVEAN SCORE**	**PROVEAN Prediction**
**rs752722523**	NP_006352	G-A	A282T	0.042	Neutral
**rs763960333**	NP_006352	G-C	S281T	0.664	Neutral
**rs200997384**	NP_006352	G-T	V278L	0.881	Neutral
**rs191886930**	NP_006352	G-A	A276T	-0.061	Neutral
**rs761914407**	NP_006352	C-T	R215C	-6.781	Deleterious
**rs145059285**	NP_006352	G-A	A212T	-0.156	Neutral
**rs758190709**	NP_006352	C-T	A210V	-0.297	Neutral
**rs766169510**	NP_006352	G-C	Q205H	-0.501	Neutral
**rs761530565**	NP_006352	A-G	Q205R	-0.446	Neutral
**rs750945370**	NP_006352	G-T	A200S	-0.349	Neutral
**rs756654236**	NP_006352	G-A	A197T	0.04	Neutral
**rs780355420**	NP_006352	T-A	F194I	-0.423	Neutral
**rs779330626**	NP_006352	A-G	Q188R	-3.397	Deleterious
**rs748353425**	NP_006352	G-A	M182I	-0.434	Neutral
**rs772349818**	NP_006352	T-C	M182T	-2.836	Deleterious
**rs570681642**	NP_006352	A-G	Q181R	-3.355	Deleterious
**rs777986934**	NP_006352	G-T	G177V	-7.026	Deleterious
**rs539086211**	NP_006352	G-A	G176D	0.346	Neutral
**rs747003841**	NP_006352	G-C	G176R	-0.784	Neutral
**rs148649390**	NP_006352	G-T	A175S	-0.063	Neutral
**rs587780164**	NP_006352	G-A	D167N	-2.914	Deleterious
**rs751081605**	NP_006352	C-G	S162C	-1.91	Neutral
**rs587780163**	NP_006352	C-T	A154V	-2.246	Neutral
**rs766929278**	NP_006352	G-T	G153V	-4.476	Deleterious
**rs754280897**	NP_006352	G-A	G153S	-1.401	Neutral
**rs140373548**	NP_006352	C-A	L152M	-1.02	Neutral
**rs587780162**	NP_006352	G-A	V146M	0.2	Neutral
**rs556045007**	NP_006352	C-T	A141V	-1.561	Neutral
**rs575899185**	NP_006352	G-C	Q138H	-2.605	Deleterious
**rs770891609**	NP_006352	A-C	Y137S	-7.227	Deleterious
**rs745800027**	NP_006352	A-C	T136P	4.834	Neutral
**rs769634543**	NP_006352	G-A	G135E	-2.745	Deleterious
**rs775273363**	NP_006352	C-T	A128V	-3.159	Deleterious
**rs762666370**	NP_006352	C-G	P124R	-1.079	Neutral
**rs201428095**	NP_006352	G-A	R123H	-4.202	Deleterious
**rs8556**	NP_006352	C-A	S122R	-3.662	Deleterious
**rs766909225**	NP_006352	C-G	P121A	-0.859	Neutral

**rs760111060**	NP_006352	G-A	G117E	-3.229	Deleterious
**rs533641489**	NP_006352	G-A	G117R	-3.205	Deleterious
**rs764401781**	NP_006352	C-T	T115M	0.287	Neutral
**rs751698154**	NP_006352	C-A	P114T	-0.506	Neutral
**rs781117900**	NP_006352	C-A	A111E	-0.244	Neutral
**rs745889927**	NP_006352	G-A	A111T	-0.392	Neutral
**rs370361482**	NP_006352	C-G	P110A	-0.994	Neutral
**rs774095240**	NP_006352	C-T	A108V	-1.634	Neutral
**rs747755369**	NP_006352	G-C	A107P	-1.472	Neutral
**rs771674803**	NP_006352	T-G	L106R	-1.488	Neutral
**rs140492479**	NP_006352	C-T	T105I	-0.984	Neutral
**rs760196832**	NP_006352	C-T	A103V	-1.321	Neutral
**rs763448911**	NP_006352	C-T	A101V	-1.803	Neutral
**rs764359688**	NP_006352	C-A	P99T	0.059	Neutral
**rs751865027**	NP_006352	T-A	L97Q	-2.115	Neutral
**rs757433384**	NP_006352	C-A	L97M	-0.739	Neutral
**rs756135357**	NP_006352	G-A	R94Q	-0.084	Neutral
**rs780012601**	NP_006352	T-G	S93A	-0.898	Neutral
**rs778563157**	NP_006352	G-A	G85D	-1.385	Neutral
**rs529392210**	NP_006352	G-A	G85S	0.15	Neutral
**rs138213197**	NP_006352	G-A	G84E	-6.485	Deleterious
**rs772962401**	NP_006352	A-G	Y80C	-6.313	Deleterious
**rs746542615**	NP_006352	G-T	V78L	1.552	Neutral
**rs763353615**	NP_006352	C-T	T73M	-1.882	Neutral
**rs774579054**	NP_006352	G-T	G72V	-1.918	Neutral
**rs750621041**	NP_006352	C-T	P70L	-2.255	Neutral
**rs370934116**	NP_006352	C-A	P70T	-0.825	Neutral
**rs766443552**	NP_006352	G-T	V69L	-0.842	Neutral
**rs587780161**	NP_006352	C-T	P67S	-0.805	Neutral
**rs778843798**	NP_006352	T-C	C66R	-4.527	Deleterious
**rs199813155**	NP_006352	G-A	C63Y	-4.179	Deleterious
**rs758166293**	NP_006352	T-G	C63G	-4.117	Deleterious
**rs568967699**	NP_006352	A-T	K61M	-2.423	Neutral
**rs770620686**	NP_006352	C-T	P59L	-1.999	Neutral
**rs745306184**	NP_006352	G-T	A57S	-0.017	Neutral
**rs769074510**	NP_006352	C-T	P54S	-0.417	Neutral
**rs11551892**	NP_006352	G-C	A49P	0.228	Neutral
**rs748333323**	NP_006352	A-G	N47S	0.712	Neutral
**rs199799743**	NP_006352	C-T	T41M	-0.582	Neutral
**rs773491778**	NP_006352	C-A	A39E	-1.178	Neutral

**rs760874697**	NP_006352	G-T	A39S	0.481	Neutral
**rs587780160**	NP_006352	C-A	A38E	-1.433	Neutral
**rs550726919**	NP_006352	C-A	H36N	-1.335	Neutral
**rs79344505**	NP_006352	C-A	L33M	-0.184	Neutral
**rs758169931**	NP_006352	C-G	S31C	-0.623	Neutral
**rs561048036**	NP_006352	C-G	H30Q	-1.909	Neutral
**rs751338230**	NP_006352	A-G	H30R	-2.13	Neutral
**rs756970136**	NP_006352	G-T	A29S	-0.24	Neutral
**rs587780165**	NP_006352	G-A	R25Q	-1.319	Neutral
**rs780947625**	NP_006352	G-A	G24R	-1.286	Neutral
**rs745392080**	NP_006352	G-A	G23E	0.537	Neutral
**rs769323553**	NP_006352	G-A	G23R	0.154	Neutral
**rs539706443**	NP_006352	G-A	G22R	-1.486	Neutral
**rs772484566**	NP_006352	C-T	A21V	-1.81	Neutral
**rs747307642**	NP_006352	C-T	T8I	-0.308	Neutral
**rs771173385**	NP_006352	A-T	Y6F	-0.009	Neutral
**rs776869015**	NP_006352	G-T	G4V	-0.234	Neutral
**rs546307661**	NP_006352	C-A	P3T	-0.486	Neutral

Table S5. List of nsSNP's predicted by nsSNPAnalyzer as deleterious								
Variant ID	Nucleotide Variation	SNP	nsSNPAnalyzer Predictions					
			Phenotype	Environment	Area Buried	Frac. Polar	Secondstr	ScopLink
**rs752722523**	G-A	A282T	Neutral	-	-	-	-	-
**rs763960333**	G-C	S281T	Neutral	-	-	-	-	-
**rs200997384**	G-T	V278L	Neutral	-	-	-	-	-
**rs191886930**	G-A	A276T	Neutral	-	-	-	-	-
**rs761914407**	C-T	R215C	Disease	P2C	0.177	0.812	C	d1ahdp_
**rs145059285**	G-A	A212T	Neutral	-	-	-	-	-
**rs758190709**	C-T	A210V	Neutral	-	-	-	-	-
**rs766169510**	G-C	Q205H	Neutral	-	-	-	-	-
**rs761530565**	A-G	Q205R	Neutral	-	-	-	-	-
**rs750945370**	G-T	A200S	Neutral	-	-	-	-	-
**rs756654236**	G-A	A197T	Neutral	-	-	-	-	-
**rs780355420**	T-A	F194I	Neutral	-	-	-	-	-
**rs779330626**	A-G	Q188R	Disease	-	-	-	-	-
**rs748353425**	G-A	M182I	Disease	-	-	-	-	-
**rs772349818**	T-C	M182T	Neutral	-	-	-	-	-
**rs570681642**	A-G	Q181R	Disease	-	-	-	-	-
**rs777986934**	G-T	G177V	Disease	-	-	-	-	-
**rs539086211**	G-A	G176D	Disease	-	-	-	-	-
**rs747003841**	G-C	G176R	Disease	-	-	-	-	-
**rs148649390**	G-T	A175S	Neutral	-	-	-	-	-
**rs587780164**	G-A	D167N	Disease	-	-	-	-	-
**rs751081605**	C-G	S162C	Disease	-	-	-	-	-
**rs587780163**	C-T	A154V	Neutral	-	-	-	-	-
**rs766929278**	G-T	G153V	Disease	-	-	-	-	-
**rs754280897**	G-A	G153S	Neutral	-	-	-	-	-
**rs140373548**	C-A	L152M	Disease	-	-	-	-	-
**rs587780162**	G-A	V146M	Neutral	-	-	-	-	-
**rs556045007**	C-T	A141V	Disease	-	-	-	-	-
**rs575899185**	G-C	Q138H	Disease	-	-	-	-	-
**rs770891609**	A-C	Y137S	Disease	-	-	-	-	-
**rs745800027**	A-C	T136P	Neutral	-	-	-	-	-
**rs769634543**	G-A	G135E	Disease	-	-	-	-	-
**rs775273363**	C-T	A128V	Disease	-	-	-	-	-
**rs762666370**	C-G	P124R	Disease	-	-	-	-	-
**rs201428095**	G-A	R123H	Disease	-	-	-	-	-
**rs8556**	C-A	S122R	Disease	-	-	-	-	-
**rs766909225**	C-G	P121A	Neutral	-	-	-	-	-

**rs760111060**	G-A	G117E	Disease	-	-	-	-	-
**rs533641489**	G-A	G117R	Disease	-	-	-	-	-
**rs764401781**	C-T	T115M	Disease	-	-	-	-	-
**rs751698154**	C-A	P114T	Neutral	-	-	-	-	-
**rs781117900**	C-A	A111E	Neutral	-	-	-	-	-
**rs745889927**	G-A	A111T	Neutral	-	-	-	-	-
**rs370361482**	C-G	P110A	Neutral	-	-	-	-	-
**rs774095240**	C-T	A108V	Neutral	-	-	-	-	-
**rs747755369**	G-C	A107P	Neutral	-	-	-	-	-
**rs771674803**	T-G	L106R	Neutral	-	-	-	-	-
**rs140492479**	C-T	T105I	Disease	-	-	-	-	-
**rs760196832**	C-T	A103V	Neutral	-	-	-	-	-
**rs763448911**	C-T	A101V	Disease	-	-	-	-	-
**rs764359688**	C-A	P99T	Neutral	-	-	-	-	-
**rs751865027**	T-A	L97Q	Neutral	-	-	-	-	-
**rs757433384**	C-A	L97M	Neutral	-	-	-	-	-
**rs756135357**	G-A	R94Q	Neutral	-	-	-	-	-
**rs780012601**	T-G	S93A	Neutral	-	-	-	-	-
**rs778563157**	G-A	G85D	Disease	-	-	-	-	-
**rs529392210**	G-A	G85S	Neutral	-	-	-	-	-
**rs138213197**	G-A	G84E	Disease	-	-	-	-	-
**rs772962401**	A-G	Y80C	Disease	-	-	-	-	-
**rs746542615**	G-T	V78L	Neutral	-	-	-	-	-
**rs763353615**	C-T	T73M	Disease	-	-	-	-	-
**rs774579054**	G-T	G72V	Disease	-	-	-	-	-
**rs750621041**	C-T	P70L	Disease	-	-	-	-	-
**rs370934116**	C-A	P70T	Disease	-	-	-	-	-
**rs766443552**	G-T	V69L	Disease	-	-	-	-	-
**rs587780161**	C-T	P67S	Neutral	-	-	-	-	-
**rs778843798**	T-C	C66R	Disease	-	-	-	-	-
**rs199813155**	G-A	C63Y	Disease	-	-	-	-	-
**rs758166293**	T-G	C63G	Neutral	-	-	-	-	-
**rs568967699**	A-T	K61M	Disease	-	-	-	-	-
**rs770620686**	C-T	P59L	Disease	-	-	-	-	-
**rs745306184**	G-T	A57S	Neutral	-	-	-	-	-
**rs769074510**	C-T	P54S	Neutral	-	-	-	-	-
**rs11551892**	G-C	A49P	Neutral	-	-	-	-	-
**rs748333323**	A-G	N47S	Neutral	-	-	-	-	-
**rs199799743**	C-T	T41M	Disease	-	-	-	-	-
**rs773491778**	C-A	A39E	Disease	-	-	-	-	-
**rs760874697**	G-T	A39S	Neutral	-	-	-	-	-

**rs587780160**	C-A	A38E	Disease	-	-	-	-	-	-
**rs550726919**	C-A	H36N	Neutral	-	-	-	-	-	-
**rs79344505**	C-A	L33M	Disease	-	-	-	-	-	-
**rs758169931**	C-G	S31C	Disease	-	-	-	-	-	-
**rs561048036**	C-G	H30Q	Disease	-	-	-	-	-	-
**rs751338230**	A-G	H30R	Disease	-	-	-	-	-	-
**rs756970136**	G-T	A29S	Neutral	-	-	-	-	-	-
**rs587780165**	G-A	R25Q	Disease	-	-	-	-	-	-
**rs780947625**	G-A	G24R	Disease	-	-	-	-	-	-
**rs745392080**	G-A	G23E	Neutral	-	-	-	-	-	-
**rs769323553**	G-A	G23R	Disease	-	-	-	-	-	-
**rs539706443**	G-A	G22R	Disease	-	-	-	-	-	-
**rs772484566**	C-T	A21V	Disease	-	-	-	-	-	-
**rs747307642**	C-T	T8I	Disease	-	-	-	-	-	-
**rs771173385**	A-T	Y6F	Neutral	-	-	-	-	-	-
**rs776869015**	G-T	G4V	Disease	-	-	-	-	-	-
**rs546307661**	C-A	P3T	Disease	-	-	-	-	-	-

Table S6. List of nsSNP's predicted by PhD-SNP server as deleterious				
**Variant ID**	**Nucleotide Variation**	**AA Variation**	**PhD-SNP Sequence & Profilebased Prediction**	**RI**
**rs752722523**	G-A	A282T	Neutral	8
**rs763960333**	G-C	S281T	Neutral	8
**rs200997384**	G-T	V278L	Neutral	9
**rs191886930**	G-A	A276T	Neutral	6
**rs761914407**	C-T	R215C	Disease	7
**rs145059285**	G-A	A212T	Neutral	7
**rs758190709**	C-T	A210V	Neutral	5
**rs766169510**	G-C	Q205H	Neutral	8
**rs761530565**	A-G	Q205R	Neutral	7
**rs750945370**	G-T	A200S	Neutral	5
**rs756654236**	G-A	A197T	Neutral	5
**rs780355420**	T-A	F194I	Neutral	4
**rs779330626**	A-G	Q188R	Disease	0
**rs748353425**	G-A	M182I	Neutral	5
**rs772349818**	T-C	M182T	Disease	0
**rs570681642**	A-G	Q181R	Neutral	0
**rs777986934**	G-T	G177V	Disease	8
**rs539086211**	G-A	G176D	Neutral	3
**rs747003841**	G-C	G176R	Disease	1
**rs148649390**	G-T	A175S	Neutral	7
**rs587780164**	G-A	D167N	Neutral	6
**rs751081605**	C-G	S162C	Neutral	5
**rs587780163**	C-T	A154V	Neutral	6
**rs766929278**	G-T	G153V	Disease	1
**rs754280897**	G-A	G153S	Neutral	6
**rs140373548**	C-A	L152M	Neutral	4
**rs587780162**	G-A	V146M	Neutral	6
**rs556045007**	C-T	A141V	Neutral	1
**rs575899185**	G-C	Q138H	Disease	6
**rs770891609**	A-C	Y137S	Disease	8
**rs745800027**	A-C	T136P	Neutral	7
**rs769634543**	G-A	G135E	Neutral	7
**rs775273363**	C-T	A128V	Neutral	1
**rs762666370**	C-G	P124R	Neutral	7
**rs201428095**	G-A	R123H	Disease	4
**rs8556**	C-A	S122R	Disease	1
**rs766909225**	C-G	P121A	Neutral	8
**rs760111060**	G-A	G117E	Neutral	7

**rs533641489**	G-A	G117R	Neutral	7
**rs764401781**	C-T	T115M	Neutral	7
**rs751698154**	C-A	P114T	Neutral	8
**rs781117900**	C-A	A111E	Neutral	2
**rs745889927**	G-A	A111T	Neutral	7
**rs370361482**	C-G	P110A	Neutral	7
**rs774095240**	C-T	A108V	Neutral	7
**rs747755369**	G-C	A107P	Neutral	0
**rs771674803**	T-G	L106R	Neutral	1
**rs140492479**	C-T	T105I	Neutral	7
**rs760196832**	C-T	A103V	Neutral	7
**rs763448911**	C-T	A101V	Neutral	6
**rs764359688**	C-A	P99T	Neutral	7
**rs751865027**	T-A	L97Q	Disease	3
**rs757433384**	C-A	L97M	Neutral	6
**rs756135357**	G-A	R94Q	Neutral	3
**rs780012601**	T-G	S93A	Neutral	6
**rs778563157**	G-A	G85D	Neutral	2
**rs529392210**	G-A	G85S	Neutral	6
**rs138213197**	G-A	G84E	Disease	4
**rs772962401**	A-G	Y80C	Disease	7
**rs746542615**	G-T	V78L	Neutral	9
**rs763353615**	C-T	T73M	Neutral	7
**rs774579054**	G-T	G72V	Neutral	6
**rs750621041**	C-T	P70L	Neutral	8
**rs370934116**	C-A	P70T	Neutral	6
**rs766443552**	G-T	V69L	Neutral	7
**rs587780161**	C-T	P67S	Neutral	8
**rs778843798**	T-C	C66R	Neutral	1
**rs199813155**	G-A	C63Y	Neutral	0
**rs758166293**	T-G	C63G	Neutral	5
**rs568967699**	A-T	K61M	Neutral	5
**rs770620686**	C-T	P59L	Neutral	7
**rs745306184**	G-T	A57S	Neutral	8
**rs769074510**	C-T	P54S	Neutral	7
**rs11551892**	G-C	A49P	Neutral	8
**rs748333323**	A-G	N47S	Neutral	9
**rs199799743**	C-T	T41M	Neutral	7
**rs773491778**	C-A	A39E	Neutral	5
**rs760874697**	G-T	A39S	Neutral	8
**rs587780160**	C-A	A38E	Neutral	3

**rs550726919**	C-A	H36N	Neutral	8
**rs79344505**	C-A	L33M	Neutral	6
**rs758169931**	C-G	S31C	Neutral	8
**rs561048036**	C-G	H30Q	Neutral	4
**rs751338230**	A-G	H30R	Neutral	4
**rs756970136**	G-T	A29S	Neutral	7
**rs587780165**	G-A	R25Q	Neutral	3
**rs780947625**	G-A	G24R	Neutral	2
**rs745392080**	G-A	G23E	Neutral	6
**rs769323553**	G-A	G23R	Neutral	4
**rs539706443**	G-A	G22R	Neutral	3
**rs772484566**	C-T	A21V	Neutral	7
**rs747307642**	C-T	T8I	Neutral	5
**rs771173385**	A-T	Y6F	Neutral	9
**rs776869015**	G-T	G4V	Neutral	4
**rs546307661**	C-A	P3T	Neutral	9

Table S7. PolymiRTS database results

dbSNP ID	miR ID	Conservation	miRSite	Function Class	context+ score change
**rs8064432**	hsa-miR-30a-3p	6	gaaACTGAAActt	D	-0.124
	hsa-miR-30d-3p	6	gaaACTGAAActt	D	-0.124
	hsa-miR-30e-3p	6	gaaACTGAAActt	D	-0.124
**rs8082520**	hsa-miR-4749-3p	2	caaGAGGGGCtgc	C	-0.247
	hsa-miR-6769a-3p	3	caagAGGGGCTgc	C	-0.168
	hsa-miR-4691-3p	4	caagaGTGGCTGc	D	-0.166
	hsa-miR-6777-3p	2	caAGAGTGGctgc	D	-0.186
**rs116931900**	hsa-miR-4761-5p	13	ggtgaCACCTTGg	C	-0.124
**rs145426191**	hsa-miR-6802-3p	5	tgcGGGGTGAccc	D	-0.168
	hsa-miR-6879-3p	10	tgcgGGGTGACcc	D	-0.141
	hsa-miR-4690-3p	5	tgcGGGCTGAccc	C	-0.13
	hsa-miR-5685	5	tgcGGGCTGAccc	C	-0.127
**rs79812861**	hsa-miR-6872-3p	2	agcGGCATGGtgc	D	-0.11
	hsa-miR-767-5p	5	agcggcATGGTGC	D	-0.054
**rs61123825**	hsa-miR-4286	3	tGTGGGGAggggg	D	-0.098
	hsa-miR-6887-3p	3	tgtggGGAGGGGg	D	-0.148
	hsa-miR-1260a	3	tGTGGGAAggggg	C	-0.08
	hsa-miR-1260b	3	tGTGGGAAggggg	C	-0.08
	hsa-miR-188-3p	3	TGTGGGAAggggg	C	-0.207
	hsa-miR-3127-3p	4	tgtggGAAGGGGg	C	-0.171

	hsa-miR-3156-3p	3	tGTGGGAAggggg	C	-0.092
	hsa-miR-6734-3p	2	tgtGGGAAGGggg	C	-0.121
	hsa-miR-6756-3p	3	tgtggGAAGGGGg	C	-0.143
	hsa-miR-1321	21	aacccCCTCCCTc	D	-0.076
**rs148791210**	hsa-miR-4739	21	aacccCCTCCCTc	D	-0.085
	hsa-miR-4756-5p	21	aacccCCTCCCTc	D	-0.104
	hsa-miR-3609	2	caggTCACTTTct	D	0.057
**rs142428986**	hsa-miR-548ah-5p	2	caggTCACTTTct	D	0.048
	hsa-miR-4670-5p	3	caGGTCGCTttct	C	-0.285
	hsa-miR-3609	2	agcaggTCACTTT	C	0.057
	hsa-miR-548ah-5p	2	agcaggTCACTTT	C	0.048
**rs8065028**	hsa-miR-3677-5p	3	agcaGGCCACTtt	D	-0.212
	hsa-miR-4796-3p	2	agcaggCCACTTT	D	0.029
	hsa-miR-3619-3p	6	gccccgTGGTCCA	C	-0.121
**rs8065051**	hsa-miR-4776-5p	6	gccccgTGGTCCA	C	-0.127
	hsa-miR-744-5p	2	GCCCCGCggtcca	D	-0.286
**rs8069252**	hsa-miR-660-3p	7	acgAGGAGGAgga	C	-0.122
	hsa-miR-6749-3p	22	acgaggGGGAGGA	D	-0.177
	hsa-miR-499b-5p	2	cctgAAGTCTGga	D	0.027
**rs189589365**	hsa-miR-205-3p	6	cCTGAAATctgga	C	0.127
	hsa-miR-488-5p	2	cctgaaATCTGGA	C	-0.01
	hsa-miR-1270	2	TCTCCAAgattga	C	0.001
**rs1056656**	hsa-miR-4531	2	TCTCCAAgattga	C	-0.065
	hsa-miR-620	2	TCTCCAAgattga	C	0.001
**rs184053751**	hsa-miR-6777-3p	2	ggcAGAGTGGtgc	D	-0.001
**rs144241488**	hsa-miR-3912-5p	3	aggTGGACAAttg	D	-0.075
	hsa-miR-1224-3p	2	AGGTGGGcaattg	C	-0.156
	hsa-miR-4251	21	catttTTCTCAGc	D	-0.091
**rs187003057**	hsa-miR-539-5p	21	cattTTTCTCAgc	D	0.026
	hsa-miR-3662	21	CATTTTActcagc	C	0.103
	hsa-miR-8077	21	cattttACTCAGC	C	-0.173
	hsa-miR-4777-5p	8	tttctATCTAGAg	D	-0.136
	hsa-miR-517-5p	8	tttctaTCTAGAG	D	-0.18
	hsa-miR-196a-5p	9	tttCTACCTAgag	C	-0.136
**rs146552906**	hsa-miR-196b-5p	9	tttCTACCTAgag	C	-0.133
	hsa-miR-3927-3p	9	ttTCTACCTAgag	C	-0.359
	hsa-miR-4650-3p	11	tTTCTACCtagag	C	-0.216
	hsa-miR-6831-5p	9	ttTCTACCTAgag	C	-0.391
	hsa-miR-4777-5p	8	ttttctATCTAGA	D	-0.136
**rs2280354**	hsa-miR-4305	8	ttttcTGTCTAGA	C	-0.256
	hsa-miR-4524b-3p	9	ttttCTGTCTAga	C	-0.087

**rs192244427**	hsa-miR-3165	5	acTCCACCAgggt	D	-0.13
	hsa-miR-4456	6	actCCACCAGggt	D	-0.165
	hsa-miR-6880-5p	4	aCTCCACCAgggt	D	-0.34
	hsa-miR-8071	5	acTCCACCAgggt	D	-0.137
	hsa-miR-1287-5p	5	acTCCAGCAgggt	C	-0.123
	hsa-miR-3135b	4	aCTCCAGCAgggt	C	-0.302
	hsa-miR-3692-5p	6	actCCAGCAGggt	C	-0.158
	hsa-miR-370-3p	3	actcCAGCAGGgt	C	-0.154
	hsa-miR-6893-3p	3	actcCAGCAGGgt	C	-0.135
**rs183620920**	hsa-miR-6755-3p	2	GACAACAcccttc	D	-0.068
**rs187442839**	hsa-miR-27a-5p	2	cAAGCCCAggctg	D	-0.112
	hsa-miR-378g	2	caAGCCCAGgctg	D	-0.14
	hsa-miR-661	2	caagCCCAGGCtg	D	-0.2
	hsa-miR-939-3p	2	caaGCCCAGGctg	D	-0.193
	hsa-miR-212-5p	2	caaGCCAAGGctg	C	-0.159
**rs141179592**	hsa-miR-1343-3p	3	gaCCAGGAAcctg	D	-0.078
	hsa-miR-6783-3p	3	gaCCAGGAAcctg	D	-0.088
	hsa-miR-3663-5p	4	GACCAGAaacctg	C	-0.183
	hsa-miR-7152-3p	4	GACCAGAaacctg	C	-0.136
**rs144967504**	hsa-miR-1343-3p	3	ggagaCCAGGAAc	D	-0.078
	hsa-miR-3663-5p	4	ggAGACCAGgaac	D	-0.085
	hsa-miR-6783-3p	3	ggagaCCAGGAAc	D	-0.088
	hsa-miR-6843-3p	4	gGAGACCAggaac	D	-0.056
	hsa-miR-6848-3p	4	gGAGACCAggaac	D	-0.111
	hsa-miR-1238-3p	3	ggagaCGAGGAAc	C	-0.105
	hsa-miR-4326	3	ggagacGAGGAAC	C	-0.166

Table S8. Results of template structure search (NCBI psiBLAST)

S. No	PDB ID	AA Length	Identity (%)	Chain	Query cover (%)	Total Score	E-Value
**1**	2LD5	67	78	A	23	118	7E-34
**2**	2L7Z	67	78	A	23	118	7E-34
**3**	2CRA	58	100	A	20	112	1E-31

MODELLER v9.17 Python Scripts

"script2.py"

```
from modeller import *
env = environ()
aln = alignment(env)
for (pdb, chain) in (('2cra', 'A'), ('2l7z', 'A'), ('2ld5', 'A')):
    m = model(env, file=pdb, model_segment=('FIRST:'+chain, 'LAST:'+chain))
    aln.append_model(m, atom_files=pdb, align_codes=pdb+chain)
aln.malign()
aln.malign3d()
aln.compare_structures()
aln.id_table(matrix_file='family.mat')
env.dendrogram(matrix_file='family.mat', cluster_cut=-1.0)
```

"script3.py"

```
from modeller import *
env = environ()
aln = alignment(env)
mdl = model(env, file='2l7z', model_segment=('FIRST:A','LAST:A'))
aln.append_model(mdl, align_codes='2l7z', atom_files='2l7z.pdb')
aln.append(file='hoxb13.ali', align_codes='hoxb13')
aln.align2d()
aln.write(file='hoxb13-2l7z.ali', alignment_format='PIR')
aln.write(file='hoxb13-2l7z.pap', alignment_format='PAP')
```

"script4.py"

```
from modeller import *
from modeller.automodel import *
#from modeller import soap_protein_od
env = environ()
```

```
a = automodel(env, alnfile='hoxb13-2l7z.ali',
              knowns='2l7z', sequence='hoxb13',
              assess_methods=(assess.DOPE,
                              #soap_protein_od.Scorer(),
                              assess.GA341))
a.starting_model = 1
a.ending_model = 30
a.make()
```

Supplementary Figures

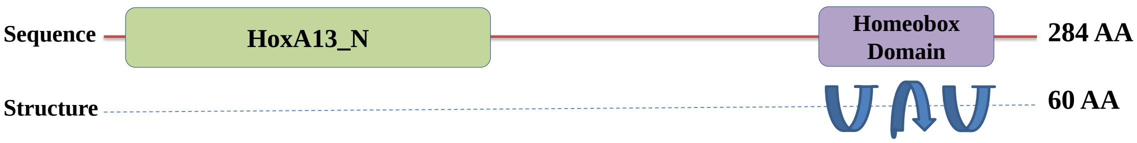

Figure S1. A graphical representation of the functional domains in the *HOXB13* protein. The figure depicts the location of the corresponding domains of *HOXB13* with insights into its secondary structure. It consists of two functional domains – Homeobox binding domain and HOXA13 N-terminal domain.

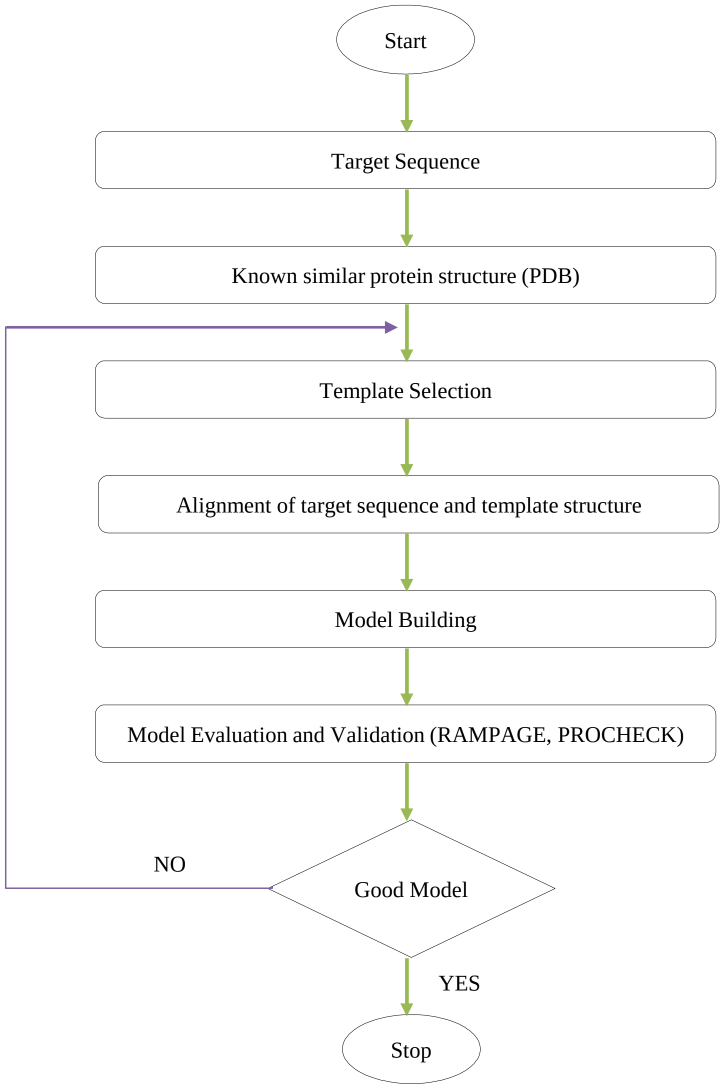

Figure S2. Steps in Homology Modelling. The figure depicts the work flow involved in the modelling of complete *HOXB13* protein using MODELLER v9.17

Predict Not Tolerated	Position	Seq Rep	Predict Tolerated
y w v t s r q p n l k i h g f e d c a	1M	0.11	M
c w m f i y l v h r g t n s p a k q	2E	0.11	D E
w h y f m i r q c l e k v d t g	3P	0.15	N S A P
w m y f h i c q r l e v k n t d s a	4G	0.15	P G
w	5N	0.18	c y m h i F v l P g r q D A e T N S K
	6Y	0.19	c w p m d k q n g r i t s E v A H I F Y
w f m y h	7A	0.20	c i l q e v d R k P g N s T A
w	8T	0.21	c m f H y p I l r G q V d k e N A S T
	9L	0.21	c w p D g n i t e s M v H R k f Q y A L
c w m f i y l v r h p g s k q T	10D	0.49	A N E D
w	11G	0.49	c f y M h I p V L G n R t q d S A K E
w	12A	0.46	m c q d r h p e k I v F y L G T A S N
c w f m y i h g l n t s e V D R	13K	0.46	P Q A K
w m f c l v y r h p q	14D	0.46	t k g I E S A D N
h d w e p q n c r k y a f S T	15I	0.52	G I L V M
w c m f i y l v r h p a k q n S	16E	0.52	T G D E
w m i f y l c h r q e k p t d a V	17G	0.52	N S G
d h c g e w r k y p q t a N m V i	18L	0.52	S F L
	19L	0.52	w d e q n g M C K P T R S I H A F L y V
w h y f i m n q r d e l k c v t S	20G	0.53	P A G
w m f i c l y v r p q k	21A	0.53	n E T D H S G A
w m f i c l y v r	22G	0.54	h q p k A d E T S G N
	23G	0.56	w c m d P e k n r i Q t G S v H L A F y
w h y f m i r q e l k n v	24G	0.56	P T D G S A C
w h y f i m c l q n d v k t p	25R	0.56	S G E A R
w c m f i y v p a q s g K	26N	0.56	R T H E L D N
d c w g n e s t k y a P R v m H	27L	0.56	F I Q L
h w g d n r q e s k p	28V	0.56	f Y t A C L I V M
w h y f i m n q d e l c k	29A	0.56	V T P G R S A
w m i f c v l y p a t q e k g d S	30H	0.56	R N H
	31S	0.43	C w d m e k q n P g r i t v a S H L F Y
w h y d n r f q e k c	32P	0.44	g S t i M L V P A
w d c n e q	33L	0.56	k r m g t H y P F i V S A L
	34T	0.54	w C d P M e k q n r I S T G V A H F L y
w h y f m i n q d e c k L v	35S	0.55	T P R S G A
w	36H	0.52	m c i f e v l q d t Y n P k R s G A H
w c m d e i n r k v t g y Q L F A	37P	0.54	H S P
w h y f m i r d n	38A	0.54	e c k Q L v T P G S A
w h f m i q c e l d	39A	0.55	k n R v Y G P T S A
w	40P	0.47	d H r q c e m n k y f g P I S L T A v
w c d e q k n r i h l M	41T	0.40	P Y F V S T G A
	42L	0.53	c w d M P e q k n G i R S T H V A F L y
	43M	0.53	C w d P M e k q n G r t i S V A H L f Y
w c m d	44P	0.54	i g e f v n l k R Y H A T Q P S
w h f i r q d e c n l k Y	45A	0.55	P M V T A S G
	46V	0.55	c w d P m e k q n r G i T S V h A L F Y
w	47N	0.56	y f c m h I l P V G r N q d A k E T S
	48Y	0.56	c w m D k q n E r G i P T v l H A F S Y
w y f c m h i	49A	0.56	r n q L V k G E P D A T S
w f y	50P	0.56	m h i v l C r G q P N k A S T E D
w d g n r q k	51L	0.55	f C y E P H t M S L I A V

wc y mi h	**52D**	0.55	F l v t R n q G k AS P E D
	**53L**	0.56	w c d Me q n k P r i h T G S F y L V A
wc i d h n r f k l VME T	**54P**	0.57	Q G Y A S P
wy f h i c l r q n p k ME V	**55G**	0.50	T D A S G
	**56S**	0.50	w C h f I P My G VL n r d T Qk AE S
wh y f i mq n r d l k c v t E	**57A**	0.52	P S G A
wc mi h v	**58E**	0.57	g P l n t R Y q K D A S F E
wh y f mi r q c e n k L V	**59P**	0.57	D G T S A P
wh y f i m	**60P**	0.58	n q r D l E k C t VP S G A
c wf md y i v g h l t NAS E Q	**61K**	0.61	P R K
wc f my i h v g n a r L d K S	**62Q**	0.60	E P T Q
wh y f i mq l e k d n v t p a R G S	**63C**	0.60	C
wc y f m	**64H**	0.55	v l I r G P d Q H T N e K A S
wc f my i h v r n t g k S Q	**65P**	0.54	D E A L P
wh y f i mn q r d e l k t s V G	**66C**	0.51	A P C
wh y f i mn q r d e k c v t	**67P**	0.55	L G A S P
wmi f y n q d r e k H C	**68G**	0.60	V L T s G P A
c w d	**69V**	0.56	e k q n r GMh P I f S L T Y V A
w	**70P**	0.51	c mf y H d g i n l e Rk Q S A T V P
wc m	**71Q**	0.60	i v l r y k H e g F P T D N A S Q
wy f c mh i	**72G**	0.61	v l d k P NQ E R AG T S
wh f mi r q e c l d k n v Y G	**73T**	0.62	P A T S
wh y f mi r c e l d k v n g Q	**74S**	0.62	T P A S
f y	**75P**	0.62	c mh i Wl v q R d e GKNP A T S
h y f mi r q d n c l k v P WE	**76A**	0.58	G S T A
wh y mi r q c e l d	**77P**	0.62	k n v F g T P S A
d h g n e c s wr k y p q t a I F	**78V**	0.66	MV L
wh y f mi r q l c e k d n v T a	**79P**	0.66	S G P
mq h k i e t wv n s r c l d a p f G	**80Y**	0.67	Y
wmi f l c y v r h q p k t e a d N	**81G**	0.68	S G
mi q v f t e c s a k l p r n d g W	**82Y**	0.68	H Y
mwq s k i y t e n v a r c d p l g	**83F**	0.67	H F
wf mi y h l c r v e k t p d n a S	**84G**	0.67	Q G
wmi f l c v y r	**85G**	0.68	q h t k e a P d G S N
wmi f c y l v q e t k d a n R	**86G**	0.69	P H S G
h n k r q d g e p c t s a mv wl F	**87Y**	0.70	I Y
h q r k n d g e p c s a mv i wl	**88Y**	0.70	T F Y
wmi f l c v y r h t k e	**89S**	0.71	a Q n D G S P
wh y f mn q r d i e k l t p g S V	**90C**	0.73	A C
wf c mi y v h l d p t e n a k S G	**91R**	0.73	Q R
h d n e g wc s r p k q t a f	**92V**	0.73	I Y L V M
wh f y mi c r l e d k	**93S**	0.74	Q P N V T AG S
wc f my i v l d g e a	**94R**	0.71	P N Q k S T R H
wmi f l c v y	**95S**	0.70	r q t k e P d A H G N S
f my i h c l r e v k	**96S**	0.71	d T GWP NQ A S
wd c g n p k r h t S a mf	**97L**	0.71	E Y VQ I L
c wf d mi y v g p s h n l a t Q	**98K**	0.74	E R K
wf y h mc i r l k v Q e	**99P**	0.75	n g D A T P S
wh y f i mn q r d e v t K P	**100C**	0.75	A L G S C

Predict Not Tolerated	Position	Seq Rep	Predict Tolerated
w f y m c r e i q H d k v g	**101A**	0.76	n L P S T A
w m c i	**102Q**	0.77	f r v l e k t P d n A H S G Y Q
w m i f y	**103A**	0.77	l c r q v e k t H d N P S G A
w h f m i q r d n e l c k Y	**104A**	0.78	G T V P S A
	**105T**	0.78	c w d e k q P r N M i G h V L T F Y S A
	**106L**	0.78	c w m D k q n r P G E i t S v h A F L Y
w y f c h i l	**107A**	0.78	r d N Q T E M V P S K G A
w c f m h	**108A**	0.79	i v l p r n G T Q Y A K S D E
w	**109Y**	0.79	c m d i n v e h g t l P F S Q A Y r K
w	**110P**	0.79	m i h c f q l n d R v k T E P Y S G A
g w y h d n r f q k	**111A**	0.81	s C E P L i T A V M
w c f m i y v l h r q A P K N G	**112E**	0.81	S T E D
w f m h i l Y V n q R	**113T**	0.82	E K D C P G A S T
w y f h m c i L	**114P**	0.83	d e n Q V k G R P T A S
	**115T**	0.83	c w d m k q n E r P I G T f S H Y L A V
w f m h i c r l q e k v d	**116A**	0.84	N Y G T A S P
w f m y i h c r l e k v d T	**117G**	0.85	P Q N A S G
c w m f i y l v r h t p g q S K	**118E**	0.85	A N D E
c w f m i y l v h g t n a k P Q	**119E**	0.84	R S D E
c w p d m e k q n r t i a S	**120Y**	0.84	G H V L Y F
w h y f m i r c e l d k n v	**121P**	0.84	A G Q T S P
w f m y h i c l r q e v d	**122S**	0.84	P K T A N G S
w f c m d y i v l n t e s g q k P H	**123R**	0.84	A R
w f m c i y l v H e	**124P**	0.85	q d g s k T N R P A
w f c m y i d h v l p n e Q	**125T**	0.85	R S G A T K
c w m f i y l v h t p n a k R Q G	**126E**	0.85	D S E
k q d n r g e p h t m a i S	**127F**	0.85	W C Y V L F
w f m y h i c r l e k v p d	**128A**	0.86	T Q N G S A
d h n g k r e c q s p t a w v i y L M	**129F**	0.86	F
w c h q p r e t f m k v a s i g d L	**130Y**	0.85	N Y
w m i f c l y v r	**131P**	0.86	t e k a S d n G H Q P
w h y f m i r q l c e k d v n p a T	**132G**	0.86	S G
c w m e q r i n t s v a h G	**133Y**	0.86	P K L D F Y
w y f m i r q d e c l n k H	**134P**	0.86	V S G T A P
w f m y i h c l q e v k p d	**135G**	0.86	T N R A S G
w h y f m i q c e d l n k v g T A	**136T**	0.86	R S P
h q r d e c n k w i p v l g s f A	**137Y**	0.86	M T Y
c w f m i y v l t s a n d k H P R	**138Q**	0.86	E G Q
w f y m c e q d i l k n	**139P**	0.85	g R H V T A S P
d w c g n e k p s h q t A R i	**140M**	0.85	F Y L M V
w h y f i m n q r d e l k c v T S	**141A**	0.86	G A P
m w i v f l c	**142S**	0.86	r p h t a Y k e Q n D G S
h w m i q n r d e k c l f t p g V	**143Y**	0.86	S A Y
d g h n e s w k r y p q t a v C	**144L**	0.86	F I M L
m w i f v c l y r p q h t a k n E G	**145D**	0.86	S D
w h y d f n r e k c p g l t i	**146V**	0.85	A S Q M V
w m c	**147S**	0.74	q r i e d k y l N H V F g t P A S
w h y d g r q f e k c p t i M S N	**148V**	0.74	A L V
g y h d n r q f e k s c p m i T W	**149V**	0.85	A L V
c w i y v h g l n F t	**150Q**	0.85	a d M k R E S Q P

w f m h c i l r q e v k p	**151T**	0.85	d Y N A G S T
d c e g n p k r h q t a	**152L**	0.85	V W M Y F S I L
w h y f i m q n d e l c k v t p R A	**153G**	0.85	S G
w h y f m i q r n d l k v C P	**154A**	0.85	E T S G A
w f y m i c q l e d v n k	**155P**	0.61	T R G A S H P
w h y f i m n q r d e l k c T V	**156G**	0.83	A S P G
c w f m i y l v h r g s p a N K T	**157E**	0.84	Q D E
w h y i m r q n d l c k v T	**158P**	0.84	E F G S A P
c w d f m i y v g p s h n a t e L	**159R**	0.86	Q K R
m i w v f l c r q a t k e Y P n G	**160H**	0.85	S D H
w c m i f v l y t p a s k q g N	**161D**	0.86	R H D E
w f y m c r l e i g k d v n	**162S**	0.85	H Q P A S T
d c w e g n k r s h t A I F	**163L**	0.85	M Q V P Y L
d c g h w n y s e t a k P f v m	**164L**	0.86	R Q I L
w f c m y d v l e n g q a t H k	**165P**	0.85	I R S P
w h g d n r e s k c p f a L	**166V**	0.85	T Y Q I V M
c w f m i y v r g n t s a k	**167D**	0.86	H L P Q D E
w m f i c y l v r h e k	**168S**	0.86	a P d Q N T G S
c w f m i d v g s l a t n e H r	**169Y**	0.85	K P Q Y
c w f m i y v l g t r n s a k d H	**170Q**	0.85	P E Q
w y f i m q r l c e k n d v t	**171S**	0.85	A G H S P
f m i v h p l t d s g a n q r Y	**172W**	0.85	C E K W
w h y f m i r c e l d k n v g Q	**173A**	0.85	P S T A
w h y r c g e d q k n f m a v P	**174L**	0.79	S T I L
w h y f m i	**175A**	0.77	r e c l d k n v Q P G S A T
w m i f l v c y r q p t k e H d	**176G**	0.80	A G S N
w h y f i m r n d e l k c v t Q	**177G**	0.79	P A S G
c f m d i v p g s h l a t e q N	**178W**	0.80	Y R K W
m i w v f c y r q a t k e	**179N**	0.80	L P H S G D N
m w i f v l c y r p q t e d	**180S**	0.80	H N A K S G
m w i f v c l y r t a k e s H n	**181Q**	0.80	G P D Q
g w h n y r e s k c p t	**182M**	0.81	Q D F I L A M V
w m i f	**183C**	0.81	v l p C h t a q r E Y s K g d N
w h y f i n q r d e l k v p s T G	**184C**	0.81	M A C
w f y m c h i l r v	**185Q**	0.79	g k d N T E Q P S A
w c f y m i h d v n t e	**186G**	0.79	p q s a L r G K
c w m f i y l v h r t g n s p a k q	**187E**	0.79	D E
w f m i y h c r l v k t n e s	**188Q**	0.80	D G A P Q
w f y c	**189N**	0.80	m h i v l r q P K D T E A G S N
w c y f m h v i t e s a g d r k	**190P**	0.80	L N P Q
w y f m i q r d e n c k v H L	**191P**	0.80	T G A S P
W m h f i	**192G**	0.80	y q d l e k R C N V P T A S G
c w	**193P**	0.75	m d e k Q r i N T v l G F S A P Y H
d c w e p k r s q t H y a	**194F**	0.71	v G N M i F L
h c q n e r k t d m s v p y i f a	**195W**	0.70	G L W
w c f y v h l p t s n a q D r E	**196K**	0.70	M I G K
w m i c y r q l k v p d n	**197A**	0.72	F H E G T A S
w h y f m i q r d n e l c k v	**198A**	0.72	G T P S A
c w p e n k q r s t h a m	**199F**	0.72	V D Y G I L F
w h y f m i r n d e l c k	**200A**	0.72	V Q T S G P A

Predict Not Tolerated	Position	Seq Rep	Predict Tolerated
w m c i f y l v r h t p k q s n A	**201D**	0.73	E G D
w y	**202S**	0.72	f c m H i l P g r n V q T D A K S E
w y f h m c i	**203S**	0.75	r q L d P N k T E G A S V
w y h f m i c l n q d e R	**204G**	0.74	T V K G P S A
	**205Q**	0.49	c w m d P i n G V H e t f S Q a L y R k
w m i c f v y r q t k	**206H**	0.64	P E A L d S G N H
w h y f	**207P**	0.74	i M n d c r e k L v T Q P s G A
w c f m y i v r L	**208P**	0.76	k e T D G N Q A H S P
c w m f i y l h r g t n p k a q	**209D**	0.76	V S E D
w h y f i n	**210A**	0.76	q r d e k c L t M p V S G A
w h y f i m q r e	**211C**	0.77	k d v t L p N G A C S
w y f m r c e q d g H k n i L	**212A**	0.78	P A V T S
h n r k q d g e p c t s a m i w L	**213F**	0.78	V Y F
c w f d m i y v g s l n a e k T H	**214R**	0.79	P Q R
m w f c y i h v l d e n q t p a g k	**215R**	0.79	S R
w m f c i y l v r q p H k	**276A**	0.62	g d T E N S A
c w f d m i y v s g p h n a l t e q	**277K**	0.61	R K
h w d g n r q y s k p c	**278V**	0.61	A M T F E I L V
c w f m i y v h l t s n p g a d q r	**279K**	0.61	E K
w m f i c l v y r h q a k	**280N**	0.54	G P E D N S T
w f y h m c r e g l q k d P V A	**281S**	0.54	I N T S
w h f y m i r c e q l d k p	**282A**	0.53	N G V T A S
w f y h m c r e g l q k d v p a	**283T**	0.24	I N S T
y w v t s r q n m l k i h g f e d c a	**284P**	0.08	P

Figure S3. SIFT prediction for the effect of amino acid variations in the non-homeobox region of *HOXB13* gene. The figure predicts the effect of amino acid substitution over the entire *HOXB13* protein sequence. The above figure corresponds to the SIFT output which is modified and presented only for the amino acids (1-215 and 276-284 AA) corresponding to the non-homeobox region of human *HOXB13* gene**.**

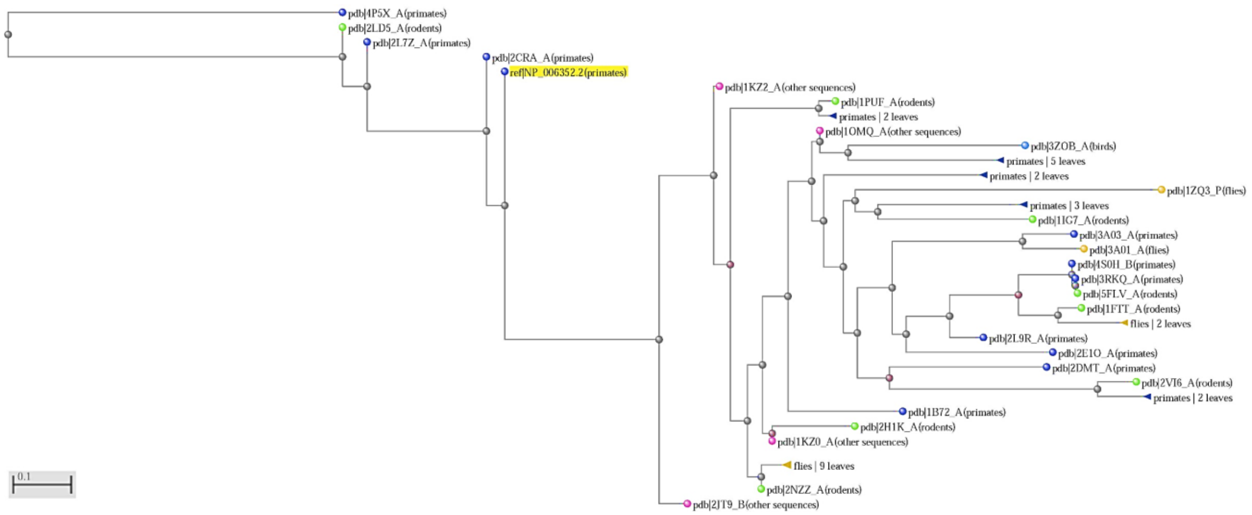

Figure S4. Distance tree (BLAST tree view). The evolutionary distance tree view for the query sequence NP_006352 of the human *HOXB13* gene against the 3D protein structures from PDB by psiBLAST. The query sequence is highlighted and the structures of 2CRA, 2LD5 and 2L7Z were found to be closely related to the query sequence. (Figure simulated using psiBLAST)

```
Sequence identity comparison (ID_TABLE):

   Diagonal       ... number of residues;
   Upper triangle ... number of identical residues;
   Lower triangle ... % sequence identity, id/min(length).

         2craA @1217zA @121d5A @1
2craA @1       70      25      25
217zA @1       36      73      67
21d5A @1       37     100      67


Weighted pair-group average clustering based on a distance matrix:


        .--------------------------------------------------------- 2craA @1.0    63.5000
        |
        |                                                      .--- 217zA @1.0     0.0000
        |                                                      |
      .---------------------------------------------------------+-- 21d5A @1.0

      +----+----+----+----+----+----+----+----+----+----+----+----+
    66.0400   54.6100   43.1800   31.7500   20.3200    8.8900   -2.5400
         60.3250   48.8950   37.4650   26.0350   14.6050    3.1750
```

Figure S5. Dendrogram of the protein models for template selection. The dendrogram depicting the sequence and structural similarity between the query sequence (NP_006352) and the templates (2CRA, 2LD5, 2L7Z) along with the crystallographic resolution and distance score. (MODELLER v9.17 output is modified and presented only with respect to the dendrogram. The complete log file is given as the supplementary material).

```
 _aln.pos         10        20        30        40        50        60
2l7z      M---------------------------------SH------M-------L------E----------
hoxb13    MEPGNYATLDGAKDIEGLLGAGGGRNLVAHSPLTSHPAAPTLMPAVNYAPLDLPGSAEPPKQCHPCPG
 _consrvd *                                 **      *       *      *

 _aln.p   70        80        90       100       110       120       130
2l7z      --------------------------------------------------------------------
hoxb13    VPQGTSPAPVPYGYFGGGYYSCRVSRSSLKPCAQAATLAAYPAETPTAGEEYPSRPTEFAFYPGYPGT
 _consrvd

 _aln.pos  140       150       160       170       180       190       200
2l7z      --------------------------------------------------------------------
hoxb13    YQPMASYLDVSVVQTLGAPGEPRHDSLLPVDSYQSWALAGGWNSQMCCQGEQNPPGPFWKAAFADSSG
 _consrvd

 _aln.pos    210       220       230       240       250       260       270
2l7z      -----------GRKKRVPYTKVQLKELEREYATNKFITKDKRRRISATTNLSERQVTIWFQNRRVKEK
hoxb13    QHPPDACAFRRGRKKRIPYSKGQLRELEREYAANKFITKDKRRKISAATSLSERQITIWFQNRRVKEK
 _consrvd            ***** ** * ** ******* ********** *** * ***** ************

 _aln.pos      280
2l7z      KVINKLK--TTS
hoxb13    KVLAKVKNSATP
 _consrvd **  * *   *
```

Figure S6. Sequence Alignment. Alignment of the query sequence (NP_006352) and the template structure (2L7Z) by “align2d()” function in MODELLER v9.17. The conserved regions are marked with “ * ”.

>> Summary of successfully produced models:

Filename	molpdf	DOPE score	GA341 score
hoxb13.B99990001.pdb	1885.69495	-10484.60254	1.00000
hoxb13.B99990002.pdb	1922.29309	-10409.19922	1.00000
hoxb13.B99990003.pdb	1842.38245	-10583.86133	1.00000
hoxb13.B99990004.pdb	1817.28308	-9984.49707	1.00000
hoxb13.B99990005.pdb	2038.60388	-10264.55859	0.99998
hoxb13.B99990006.pdb	1870.09277	-10574.50586	1.00000
hoxb13.B99990007.pdb	1816.82886	-10236.62109	1.00000
hoxb13.B99990008.pdb	1951.74414	-10313.67969	1.00000
hoxb13.B99990009.pdb	1849.80408	-10244.52930	1.00000
hoxb13.B99990010.pdb	2053.90625	-9857.58594	1.00000
hoxb13.B99990011.pdb	1971.08386	-10291.44434	1.00000
hoxb13.B99990012.pdb	1760.51611	-10441.24316	1.00000
hoxb13.B99990013.pdb	2071.74658	-9961.14844	1.00000
hoxb13.B99990014.pdb	1832.09875	-10223.19531	1.00000
hoxb13.B99990015.pdb	1922.36023	-10460.26270	1.00000
hoxb13.B99990016.pdb	1832.76843	-9883.15527	1.00000
hoxb13.B99990017.pdb	1876.18530	-10225.71875	1.00000
hoxb13.B99990018.pdb	1830.91150	-10327.13477	1.00000
hoxb13.B99990019.pdb	1969.15601	-9949.65625	1.00000
hoxb13.B99990020.pdb	1866.18628	-10174.37109	1.00000
hoxb13.B99990021.pdb	1777.33093	-10453.66211	1.00000
hoxb13.B99990022.pdb	2172.37231	-9960.09570	0.99943
hoxb13.B99990023.pdb	1922.39783	-10080.08105	1.00000
hoxb13.B99990024.pdb	1994.17981	-10181.44141	1.00000
hoxb13.B99990025.pdb	1855.46252	-10159.99902	1.00000
hoxb13.B99990026.pdb	1936.13440	-10661.23047	1.00000
hoxb13.B99990027.pdb	1940.19763	-10281.40625	1.00000
hoxb13.B99990028.pdb	1830.59180	-10607.12988	1.00000
hoxb13.B99990029.pdb	1976.05896	-10401.53516	1.00000
hoxb13.B99990030.pdb	1849.06604	-10516.29102	1.00000

Figure S7. Summary of the Generated Models. The figure depicts the summary of all the 30 *HOXB13* models generated by MODELLER v9.17 along with the molpdf value, DOPE and GA341 scores.

Secondary structure summary

Strand	Alpha helix	3-10 helix	Other	Total residues
0 (0.0%)	39 (13.7%)	0 (0.0%)	245 (86.3%)	284

(a)

3 helix-helix interactions

Helices		Helix types		Interaction type		No. interacting residues	
						Helix 1	Helix 2
1	2	H	H	N	C	4	2
1	3	H	H	I	I	8	7
2	3	H	H	I	I	4	5

(b)

3 helices

Start	End	Type	No. resid
Lys225	Ala237	H	13
Lys243	Ala252	H	10
Arg258	Lys273	H	16

(c)

18 beta turns

Turn	Sequence	Turn type	H-bond
Ala21-Gly24	AGGG	II	Yes
Thr34-Pro37	TSHP	IV	
His36-Ala39	HPAA	IV	
Ala39-Leu42	APTL	IV	
Met43-Val46	MPAV	IV	
Asn47-Pro50	NYAP	IV	
Asp52-Gly55	DLPG	IV	
Ser56-Pro59	SAEP	IV	
Gln62-Pro65	QCHP	IV	
Val69-Gly72	VPQG	VIII	
Gly216-Lys219	GRKK	IV	
Lys218-Ile221	KKRI	IV	Yes
Pro222-Lys225	PYSK	IV	
Asn238-Ile241	NKFI	VIII	
Ala276-Lys279	AKVK	IV	
Lys277-Asn280	KVKN	I	Yes
Lys279-Ala282	KNSA	IV	Yes
Asn280-Thr283	NSAT	VIII	

(d)

40 gamma turns

Start	End	Sequence	Turn type
Ala7	Leu9	ATL	INVERSE
Thr8	Asp10	TLD	INVERSE
Leu9	Gly11	LDG	INVERSE
Asp10	Ala12	DGA	INVERSE
Lys13	Ile15	KDI	INVERSE
Asp14	Glu16	DIE	INVERSE
Gly17	Leu19	GLL	INVERSE
Leu18	Gly20	LLG	INVERSE
Leu19	Ala21	LGA	INVERSE
Gly20	Gly22	GAG	INVERSE
Gly23	Arg25	GGR	INVERSE
Arg25	Leu27	RNL	INVERSE
Asn26	Val28	NLV	INVERSE
Ala29	Ser31	AHS	INVERSE
Pro37	Ala39	PAA	INVERSE
Pro40	Leu42	PTL	INVERSE
Pro44	Val46	PAV	INVERSE
Pro79	Gly81	PYG	INVERSE
Tyr80	Tyr82	YGY	INVERSE
Tyr88	Cys90	YSC	INVERSE
Ser89	Arg91	SCR	INVERSE
Cys90	Val92	CRV	INVERSE
Arg91	Ser93	RVS	INVERSE
Tyr109	Ala111	YPA	INVERSE
Glu118	Tyr120	EEY	INVERSE
Tyr120	Ser122	YPS	INVERSE
Tyr130	Gly132	YPG	INVERSE
Pro131	Tyr133	PGY	INVERSE
Val146	Val148	VSV	INVERSE
Ser147	Val149	SVV	INVERSE
Asp161	Leu163	DSL	INVERSE
Leu164	Val166	LPV	INVERSE
Pro165	Asp167	PVD	INVERSE
Ala210	Ala212	ACA	INVERSE
Cys211	Phe213	CAF	INVERSE
Arg215	Arg217	RGR	CLASSIC
Arg217	Lys219	RKK	INVERSE
Lys218	Arg220	KKR	INVERSE
Leu255	Glu257	LSE	INVERSE
Ser256	Arg258	SER	CLASSIC

(e)

Figure S8. Evaluation and validation of the hoxb13.B99990026.pdb modelled protein by PDBSum (a) The secondary structure summary (%) of the modelled *HOXB13* “hoxb13.B99990026.pdb” 3D protein structure. (b) The residues involved in the helix-helix interactions in the 3D protein structure is given in the figure. (c) The amino acid residues contributing to the alpha helical structure of the modelled *HOXB13* protein is denoted. (d) and (e) The Figure denotes the location, contributing amino acid, sequence and the associated turns (both the beta and gamma turns) present in the modelled *HOXB13* protein (Results computed from PDBSum).
