# Supplementary material for: Computational Modeling of complete HOXB13 protein for predicting the functional effect of SNPs and the associated role in hereditary prostate cancer
Source: Sci Rep. 2017 Mar 8;7:43830. doi: 10.1038/srep43830 (PMC5363706; doi:10.1038/srep43830)
Supplement: Supplementary Dataset [file srep43830-s1.zip › SR Supplementary Data Files Complete/IMutant/Y80C.pdf]

I-Mutant2.0

I-Mutant2.0 Home

Biocomputing Unit

Contact us

Last Update 27/12/06

```
**************************************************************************
**                                                                      **
**                            I-Mutant v2.0                             **
**          Predictor of Protein Stability Changes upon Mutations       **
**                                                                      **
**************************************************************************


     SEQ File: fileseq.txt

     Position   WT  NEW   Stability  RI    pH    T
           80    Y    C    Decrease   4   7.0   25


     WT:  Aminoacid in Wild-Type Protein
     NEW: New Aminoacid after Mutation
     RI:  Reliability Index
     T:   Temperature in Celsius degrees
     pH:  -log[H+]



**************************************************************************
*                                                                        *
* Capriotti E, Fariselli P and Casadio R (2005). I-Mutant2.0: predicting *
* stability changes upon mutation from the protein sequence or structure. *
* Nucl. Acids Res. 33: W306-W310.                                        *
* http://gpcr.biocomp.unibo.it/cgi/predictors/I-Mutant2.0/I-Mutant2.0.cgi *
*                                                                        *
**************************************************************************
```
