# Supplementary material for: Computational Modeling of complete HOXB13 protein for predicting the functional effect of SNPs and the associated role in hereditary prostate cancer
Source: Sci Rep. 2017 Mar 8;7:43830. doi: 10.1038/srep43830 (PMC5363706; doi:10.1038/srep43830)
Supplement: Supplementary Dataset [file srep43830-s1.zip › SR Supplementary Data Files Complete/PROVEAN/PROVEAN results.pdf]

J. Craig Venter™ INSTITUTE PROVEAN

Search

PROVEAN Result (Download)

PROVEAN Prediction - Job ID: 007702513445765

- **Query sequence** (fasta)
- **Supporting sequence set used for prediction**
  Number of sequences: **360** (fasta, E-values)
  Number of clusters: **30**
- **Score thresholds for prediction**
  (1) Default threshold is -2.5, that is:
  -Variants with a score equal to or below -2.5 are considered "deleterious,"
  -Variants with a score above -2.5 are considered "neutral."
  (2) How to use a more stringent threshold.

Variant	PROVEAN score	Prediction (cutoff= -2.5)
A282T	0.042	Neutral
S281T	0.664	Neutral
V278L	0.881	Neutral
A276T	-0.061	Neutral
V274F	-2.342	Neutral
R268Q	-3.161	Deleterious
R267C	-6.323	Deleterious
W263R	-11.339	Deleterious
R258H	-4.229	Deleterious
R258H	-4.229	Deleterious
R258C	-6.767	Deleterious
S254T	-1.250	Neutral
T253P	-4.906	Deleterious
S250L	-4.664	Deleterious
R247H	-4.232	Deleterious
R247C	-6.771	Deleterious
R246K	-2.539	Deleterious
D244N	-3.949	Deleterious
K239Q	-3.357	Deleterious
K239Q	-3.357	Deleterious
A236E	-3.981	Deleterious
R233R	0.000	Neutral
R229W	-4.438	Deleterious
Q227R	-3.390	Deleterious
Q227E	-2.543	Deleterious
P222R	-7.628	Deleterious
K218R	-2.543	Deleterious
R217L	-5.933	Deleterious
R217C	-6.781	Deleterious
G216C	-7.395	Deleterious
R215C	-6.781	Deleterious
A212T	-0.156	Neutral
A212T	-0.156	Neutral
A210V	-0.297	Neutral
Q205H	-0.501	Neutral
Q205R	-0.446	Neutral
A200S	-0.349	Neutral

A197T	0.040	Neutral
F194I	-0.423	Neutral
Q188R	-3.397	Deleterious
M182I	-0.434	Neutral
M182T	-2.836	Deleterious
Q181R	-3.355	Deleterious
G177V	-7.026	Deleterious
G176D	0.346	Neutral
G176R	-0.784	Neutral
A175S	-0.063	Neutral
D167N	-2.914	Deleterious
S162C	-1.910	Neutral
A154V	-2.246	Neutral
G153V	-4.476	Deleterious
G153S	-1.401	Neutral
L152M	-1.020	Neutral
V146M	0.200	Neutral
A141V	-1.561	Neutral
Q138H	-2.605	Deleterious
Y137S	-7.227	Deleterious
T136P	4.834	Neutral
G135E	-2.745	Deleterious
A128V	-3.159	Deleterious
P124R	-1.079	Neutral
R123H	-4.202	Deleterious
S122R	-3.662	Deleterious
P121A	-0.859	Neutral
G117E	-3.229	Deleterious
G117R	-3.205	Deleterious
T115M	0.287	Neutral
P114T	-0.506	Neutral
A111E	-0.244	Neutral
A111T	-0.392	Neutral
P110A	-0.994	Neutral
A108V	-1.634	Neutral
A107P	-1.472	Neutral
L106R	-1.488	Neutral
T105I	-0.984	Neutral
A103V	-1.321	Neutral
A101V	-1.803	Neutral
P99T	0.059	Neutral
L97Q	-2.115	Neutral
L97M	-0.739	Neutral
R94Q	-0.084	Neutral
S93A	-0.898	Neutral
G85D	-1.385	Neutral
G85S	0.150	Neutral
G84E	-6.485	Deleterious
Y80C	-6.313	Deleterious
V78L	1.552	Neutral
T73M	-1.882	Neutral
G72V	-1.918	Neutral

P70L	-2.255	Neutral
P70T	-0.825	Neutral
P70T	-0.825	Neutral
V69L	-0.842	Neutral
P67S	-0.805	Neutral
C66R	-4.527	Deleterious
C63Y	-4.179	Deleterious
C63G	-4.117	Deleterious
K61M	-2.423	Neutral
P59L	-1.999	Neutral
A57S	-0.017	Neutral
P54S	-0.417	Neutral
A49P	0.228	Neutral
N47S	0.712	Neutral
T41M	-0.582	Neutral
A39E	-1.178	Neutral
A39S	0.481	Neutral
A38E	-1.433	Neutral
A38E	-1.433	Neutral
H36N	-1.335	Neutral
L33M	-0.184	Neutral
S31C	-0.623	Neutral
H30Q	-1.909	Neutral
H30R	-2.130	Neutral
A29S	-0.240	Neutral
R25Q	-1.319	Neutral
G24R	-1.286	Neutral
G23E	0.537	Neutral
G23R	0.154	Neutral
G22R	-1.486	Neutral
A21V	-1.810	Neutral
T8I	-0.308	Neutral
Y6F	-0.009	Neutral
G4V	-0.234	Neutral
P3T	-0.486	Neutral

- Submitted at 21:06:53 EDT, Tuesday, Jul 5, 2016
- Started at 21:06:55 EDT, Tuesday, Jul 5, 2016
- Finished at 21:06:59 EDT, Tuesday, Jul 5, 2016

* The results are kept for 48 hours.
