# Supplementary material for: Computational Modeling of complete HOXB13 protein for predicting the functional effect of SNPs and the associated role in hereditary prostate cancer
Source: Sci Rep. 2017 Mar 8;7:43830. doi: 10.1038/srep43830 (PMC5363706; doi:10.1038/srep43830)
Supplement: Supplementary Dataset [file srep43830-s1.zip › SR Supplementary Data Files Complete/SIFT/AA prediction 201-284.pdf]

Predictions for positions 201 through 300

Threshold for intolerance is 0.05.
Amino acid color code: nonpolar, uncharged polar, basic, acidic.
Capital letters indicate amino acids appearing in the alignment, lower case letters result from prediction.
'Seq Rep' is the fraction of sequences that contain one of the basic amino acids. A low fraction indicates the position is either severely gapped or unalignable and has little information. Expect poor prediction at these positions.

Predict Not Tolerated	Position	Seq Rep	Predict Tolerated
w m c i f y l v r h t p k q s n A	**201D**	0.73	E G D
w y	**202S**	0.72	f c m H i l P g r n V q T D A K S E
w y f h m c i	**203S**	0.75	r q L d P N k T E G A S V
w y h f m i c l n q d e R	**204G**	0.74	T V K G P S A
	**205Q**	0.49	c w m d P i n G V H e t f S Q a L y R k
w m i c f v y r q t k	**206H**	0.64	P E A L d S G N H
w h y f	**207P**	0.74	i M n d c r e k L v T Q P s G A
w c f m y i v r L	**208P**	0.76	k e T D G N Q A H S P
c w m f i y l h r g t n p k a q	**209D**	0.76	V S E D
w h y f i n	**210A**	0.76	q r d e k c L t M p V S G A
w h y f i m q r e	**211C**	0.77	k d v t L p N G A C S
w y f m r c e q d g H k n i L	**212A**	0.78	P A V T S
h n r k q d g e p c t s a m i w L	**213F**	0.78	V Y F
c w f d m i y v g s l n a e k T H	**214R**	0.79	P Q R
m w f c y i h v l d e n q t p a g k	**215R**	0.79	S R
w h y f m i r q e l c d k n v p A	**216G**	0.78	S T G
y w v t s q p n m l k i h g f e d c a	**217R**	0.82	R
c w f m y i v h d l n e p a s q g r	**218K**	0.82	T K
y w v t s r q p n m l i h g f e d c a	**219K**	0.82	K
m w c f y i h v n l d e t q p a g k S	**220R**	0.82	R
w g y d h n f s e m p Q t l A	**221I**	0.82	K R C I V
y w v t s r q n m l k i h g f e d c a	**222P**	0.82	P
w v t s r q p n m l k i h g f e d c a	**223Y**	0.83	Y
w f y h m c r e l i q g k d v p n a	**224S**	0.83	S T
y w v t s r q p n m l i h g f e d c a	**225K**	0.83	K
	**226G**	0.83	c w d M P e k n Q r t s A I h G F L Y V
y w v t s r p n m l k i h g f e d c a	**227Q**	0.83	Q
d h g n e c w s y r k p q a f v m	**228L**	0.83	T I L
c w d m i y v g p s n t e H A q	**229R**	0.83	L F R K
c w f m i y v h r g n t s p a k D Q L	**230E**	0.83	E
y w v t s r q p n m k i h g f e d c a	**231L**	0.83	L
y w v t s r q p n m l k i h g f d c a	**232E**	0.83	E
c w f m i y v p g h a t e L D S Q	**233R**	0.83	N R K
y w v t s r q p n m l k i h g f d c a	**234E**	0.83	E
k q h n r d g e p t s a m v i w l C	**235Y**	0.83	F Y
w h m y i r d n e c k v p T	**236A**	0.83	G Q F L S A
	**237A**	0.83	w c d p m e k n Q g r h I S V L T F Y A
m w i v f l c y r p a t k e g d Q	**238N**	0.83	H S N

c w d f y g i p h s n t a e l r	**239K**	0.83	Q V M K
k q h n r d g e p c t s a m v i w l	**240F**	0.83	Y F
h d w n p e q c r g s k y t a f m V	**241I**	0.83	L I
w m f y c h i l r q e v k p g d a s	**242T**	0.83	N T
c w f d m i y v g s p h n a l t e q	**243K**	0.83	R K
c w f m i y l v h r g t n s p a k Q	**244D**	0.83	E D
c w f d m i y v g s p h n a l t e q	**245K**	0.83	R K
y w v t s q p n m l k i h g f e d c a	**246R**	0.83	R
c w d f m i v g p h n t q E S	**247R**	0.83	K A L Y R
c w d f m i y v g p s n a l T	**248K**	0.82	H Q E K R
h q w p d n e c r s k g y a t f m	**249I**	0.82	V L I
w f y h m i r q l c e k v d p t g N	**250S**	0.82	A S
w f y c m h d n i g l P s T E	**251A**	0.81	k V Q R A
w c p i M f n r y	**252A**	0.81	k e D G H Q L S V T A
w h y c r g e q d k f p m n v a I	**253T**	0.81	S L T
w m f i c l y v r h q p e k a d G	**254S**	0.81	T S N
y w v t s r q p n m k i h g f e d c a	**255L**	0.81	L
w f y h m c r e l i q g k d v p n a	**256S**	0.81	T S
c w m f i y l v h r t g n s p a k q	**257E**	0.81	D E
y w v t s q p n m l k i h g f e d c a	**258R**	0.81	R
y w v t s r p n m l k i h g f e d c a	**259Q**	0.81	Q
h q w p d n e r c k s g y a t m f l	**260I**	0.81	I V
w f c y h d i g e l p v n q a r s M	**261T**	0.80	K T
y w v t s r q p n m l k h g f e d c a	**262I**	0.80	I
y v t s r q p n m l k i h g f e d c a	**263W**	0.79	W
y w v t s r q p n m l k i h g e d c a	**264F**	0.79	F
y w v t s r p n m l k i h g f e d c a	**265Q**	0.77	Q
y w v t s r q p m l k i h g f e d c a	**266N**	0.76	N
m w f c y i h v l d n e t q p a g k S	**267R**	0.76	R
m w f c y i h v l d n e t q p a g k S	**268R**	0.76	R
h w d n r q y g e k s c p f t a i l	**269V**	0.66	M V
y w v t s r q p n m l i h g f e d c a	**270K**	0.64	K
c w f m i y v h r g n t s p a k	**271E**	0.64	L Q D E
y w v t s r q p n m l i h g f e d c a	**272K**	0.64	K
y w v t s r q p n m l i h g f e d c a	**273K**	0.64	K
h d w n e c g p r q s k y t A	**274V**	0.63	M F I L V
h p q w d c e k y a G t m S f	**275L**	0.62	R N L I V
w m f c i y l v r q p H k	**276A**	0.62	g d T E N S A
c w f d m i y v s g p h n a l t e q	**277K**	0.61	R K
h w d g n r q y s k p c	**278V**	0.61	A M T F E I L V
c w f m i y v h l t s n p g a d q r	**279K**	0.61	E K
w m f i c l v y r h q a k	**280N**	0.54	G P E D N S T
w f y h m c r e g l q k d P V A	**281S**	0.54	I N T S
w h f y m i r c e q l d k p	**282A**	0.53	N G V T A S
w f y h m c r e g l q k d v p a	**283T**	0.24	I N S T
y w v t s r q n m l k i h g f e d c a	**284P**	0.08	P
