# Supplementary material for: Computational Modeling of complete HOXB13 protein for predicting the functional effect of SNPs and the associated role in hereditary prostate cancer
Source: Sci Rep. 2017 Mar 8;7:43830. doi: 10.1038/srep43830 (PMC5363706; doi:10.1038/srep43830)
Supplement: Supplementary Dataset [file srep43830-s1.zip › SR Supplementary Data Files Complete/SIFT/SIFT aligned protein sequences.pdf]

>QUERY
MEPGNYATLDGAKDIEGLLGAGGGRNLVAHSPLTSHPAAPTLMPAVNYAPLDLPGSAEPP
KQCHPCPGVPQGTSPAPVPYGYFGGGYYSCRVSRSSLKPCAQAATLAAYPAETPTAGEEY
PSRPTEFAFYPGYPGTYQPMASYLDVSVVQTLGAPGEPRHDSLLPVDSYQSWALAGGWNS
QMCCQGEQNPPGPFWKAAFADSSGQHPPDACAFRRGRKKRIPYSKGQLRELEREYAANKF
ITKDKRRKISAATSLSERQITIWFQNRRVKEKKVLAKVKNSATP
>gi685597136 ref_XP_009188440.1 PREDICTED: homeobox protein Hox-B13 isoform X2
MEPGNYATLDGAKDIEGLLGAGGGRNLVAHSPLTSHPAAPTLMPAVNYAPLDLPGSAEPP
KQCHPCPGVPQGTSPAPVPYGYFGGGYYSCRVSRSSLKPCAQAATLAAYPAETPTAGEEY
PSRPTEFAFYPGYPGTYQPMASYLDVSVVQTLGAPGEPRHDSLLPVDSYQSWALAGGWNS
QMCCQGEQNPPGPFWKAAFADSSGQHPPDACAFRRGRKKRIPYSKGQLRELEREYAANKF
ITKDKRRKISAATSLSERQITIWFQNRRVKEKKVLAKXXXXXXX
>gi60653709 gb_AAX29548.1 homeobox B13, partial [synthetic construct]
MEPGNYATLDGAKDIEGLLGAGGGRNLVAHSPLTSHPAAPTLMPAVNYAPLDLPGSAEPP
KQCHPCPGVPQGTSPAPVPYGYFGGGYYSCRVSRSSLKPCAQAATLAAYPAETPTAGEEY
PSRPTEFAFYPGYPGTYQPMASYLDVSVVQTLGAPGEPRHDSLLPVDSYQSWALAGGWNS
QMCCQGEQNPPGPFWKAAFADSSGQHPPDACAFRRGRKKRIPYSKGQLRELEREYAANKF
ITKDKRRKISAATSLSERQITIWFQNRRVKEKKVLAKVKNSATP
>gi795250298 ref_XP_011854720.1 PREDICTED: homeobox protein Hox-B13 [Mandrillus
MEPGNYATLDGAKDIEGLLGAGGGRNLVAHSPLTSHPAAPTLMPAVNYAPLDLPGSVEPP
KQCHPCPGVPQGTSPAPVPYGYFGGGYYSCRVSRSSLKPCAQAATLAAYPAETPTAGEEY
PSRPTEFAFYPGYPGTYQPMASYLDVSVVQTLGAPGEPRHDSLLPVDSYQSWALAGGWNS
QMCCQGEQNPPGPFWKAAFADSSGQHPPDACAFRRGRKKRIPYSKGQLRELEREYAANKF
ITKDKRRKISAATSLSERQITIWFQNRRVKEKKVLAKVKNSATP
>gi635093043 ref_XP_008011214.1 PREDICTED: homeobox protein Hox-B13 [Chlorocebus
MEPGNYATLDGAKDIEGLLGAGGGRNLVAHSPLTSHPAAPTLMPAVNYAPLDLPGSAEPP
KQCHPCPGVPQGTSPAPVPYGYFGGGYYSCRVSRSSLKPCAQAATLAAYPAETPTAGEEY
PSRPTEFAFYPGYPGTYQPMASYLDVSVVQTLGAPGEPRHDSLLPVDSYQSWALAGGWNS
QMCCQGEQNPPGPFWKAAFADSSGQHPPDTCAFRRGRKKRIPYSKGQLRELEREYAANKF
ITKDKRRKISAATSLSERQITIWFQNRRVKEKKVLAKVKNSATP
>gi109114206 ref_XP_001089620.1 PREDICTED: homeobox protein Hox-B13 [Macaca
MEPGNYATLDGAKDIEGLLGAGGGRNLVAHSPLTSHPAAPTLMPAVNYAPLDLPGSAEPP
KQCHPCPGVPQGTSPAPVPYGYFGGGYYSCRVSRSSLKPCAQAATLAAYPAETPTAGEEY
PSRPTEFAFYPGYPGTYQPMASYLDVSVVQTLGTPGEPRHDSLLPVDSYQSWALAGGWNS
QMCCQGEQNPPGPFWKAAFADSSGQHPPDACAFRRGRKKRIPYSKGQLRELEREYAANKF
ITKDKRRKISAATSLSERQITIWFQNRRVKEKKVLAKVKNSATP
>gi795583451 ref_XP_011723765.1 PREDICTED: homeobox protein Hox-B13 isoform X3
MESGNYATLDGAKDIEGLLGAGGGRNLVAHSPLTSHPAAPTLMPAVNYAPLDLPGSAEPP
KQCHPCPGVPQGTSPAPVPYGYFGGGYYSCRVSRSSLKPCAQAATLAAYPAETPTAGEEY
PSRPTEFAFYPGYPGTYQPMASYLDVSVVQTLGAPGEPRHDSLLPVDSYQSWALAGGWNS
QMCCQGEQNPPGPFWKAAFADSSGQHPPDACAFRRGRKKRIPYSKGQLRELEREYAANKF
ITKDKRRKISAATSLSERQITIWFQNRRVKEKKVLAKVKNSATP
>gi559812841 gb_AHB20311.1 homeobox protein Hox-B13 variant [Homo sapiens]
MEPGNYATLDGAKDIEGLLGAGGGRNLVAHSPLTSHPAAPTLMPAVNYAPLDLPGSAEPP
KQCHPCPGVPQGTSPAPVPYGYFGGGYYSCRVSRSSLKPCAQAATLAAYPAETPTAGEEY
PSRPTEFAFYPGYPGTYQPMASYLDVSVVQTLGAPGEPRHDSLLPVDSYQSWALAGGWNS
QMCCQGEQNPPGPFWKAAFADSSGQHPPDACAFRRGRKKRIPYSKGQLRELEREYAANKF
ITKDKRRKISAATSLSERQITIWFQNRRVKKKKVLAKVKNSATP
>gi332259422 ref_XP_003278788.1 PREDICTED: homeobox protein Hox-B13 [Nomascus
MEPGNYATLDGAKDIEGLLGAGGGRNLVAHSPLTSHQAAPTLMPAVNYAPLDLPGSVEPP
KQCHPCPGVPQGTSPAPVPYGYFGGGYYSCRVPRSSLKPCAQAATLAAYPAETPTAGEEY
PSRPTEFAFYPGYPGTYQPMASYLDVSVVQTLGAPGEPRHDSLLPVDSYQSWALAGGWNS
QMCCQGEQNPPGPFWKAAFADSSGQHPPDACAFRRGRKKRIPYSKGQLRELEREYAANKF
ITKDKRRKISAATSLSERQITIWFQNRRVKEKKVLAKVKNSATP
>gi795525940 ref_XP_011901644.1 PREDICTED: homeobox protein Hox-B13 isoform X1
MEPGNYATLDGAKDIEGLLGAGGGRNLVAHSPLTSHPAAPTLMPAVNYAPLDLPGSAEPP
KQCHPCPGVPQGTSPAPVPYGYFGGGYYSCRVSRSSLKPCAQAATLAAYPAETPTAGEEY
PSRPTEFAFYPGYPGTYQPMASYLDVSVVQTLGAPGEPRHDSLLPVDSYQSWALAGGWNS
QMCCQGEQNPPGPFWKAAFADSSGQHPPDACAFRRGRKKRIPYSKGQLRELEREYAANKF
ITKDKRRKISAATSLSERQITIWFQNRRVKEKKVLAKVXXXXXX
>gi426237811 ref_XP_004012851.1 PREDICTED: homeobox protein Hox-B13 [Ovis aries]
MEPGNYTTLDGAKEIEGLLGAGGSRNLVTHSPLTSHPAAPTLLPAVNYGPLDLPGSAEPP
KQCHPCPGMPQGASPAPVPHGYFGGGYYSCRVSRSSLKPCAQAATLAAYPAETPASGEEY
PSRPTEFAFYPGYPGPYQPMASYLDVSVVQTLGAPGEPRHDSLLPVDSYQPWALTGGWNS
QMCCQGEQNPPGPFWKAAFADSSAQHPPDGCAFRRGRKKRIPYSKGQLRELEREYSANKF
ITKDKRRKISAATSLSERQITIWFQNRRVKEKKVLAKVKTSATP
>gi744588246 ref_XP_010986266.1 PREDICTED: homeobox protein Hox-B13 [Camelus
MEPGNYATLDGAKEIEGLLGTGASRNLVVHSPLTSHPAAPTLMPAVNYAPLDLPGSAEPP
KQCHPCPGVPQGAPPAPVPYGYLGGGYSSCRVSRSSLKPCAQGASLAAYPAETPASGEEY

```
ASRPTEFAFYPGYPGPYQPMASYLDMSVVQTLGGPGEPRHDSLLPVDSYQPWALTGGWNS
QMCCQGEQNPPGPFWKAAFADSSTQHPPDGCAFRRGRKKRIPYSKGQLRELEREYSANKF
ITKDKRRKISVATSLSERQITIWFQNRRVKEKKVLAKVKTSATP
>gi617633383  ref_XP_007530079.1 PREDICTED: homeobox protein Hox-B13 [Erinaceus
MEPGNYATLDGAKDLEGLLGTGGTRNLVAHSPASSHQAAPPLMPTVSYTPLELPGSAEPP
KQCHPCPPVPQGASPAPVPYSYFGGGYYSCRVSRSSLKPCAQAAPLTSYPAETPVAGEEY
PSRPTEFAFYSGYPGPYQPMASYLDVSVVQTLGAPGEPRHDSLLPVDTYQPWALTNGWNS
QMCCQGEQNPPGPFWKAAFADSSAQHLPDGCTYRRGRKKRIPYSKGQLRELEREYSTNKF
ITKDKRRKISAATSLSERQITIWFQNRRVKEKKVLAKVKTSSTX
>gi213511801  ref_NP_001133045.1 homeobox protein HoxB13aa [Salmo salar]
XXXXXXXXXELNKNMEGLVGANQCRNLMAHSALGGHPSS--LVHSSGYSAVDVSGSGEPG
KQCTPCPAVPQGSSTGPLPYGYFGNGYYPCRMGRGSLKSCTQPSALAEKYMDTPVTSEEY
PSRAKEFAFYHGYPSPYQSMASYLDVSVVQTLGA-GEPRHDTLLPMDSYQPWALTNGWGG
QMYCSKDQSQAGHLWKSALADVVA-HQHDGNSFRRGRKKRIPYTKVQLKELEKEYAANKF
ITKDKRRKISAGTNLSERQITIWFQNRRVKEKKFVAKVKTSAXX
>gi444517714  gb_ELV11732.1 Homeobox protein Hox-B13 [Tupaia chinensis]
MEPGNYATLDGAKDMEGLLGAGGGRNLVAHSPLTSHPAAPSLMPAVNYAPLDLPGSAEPP
KQCHPCPGVPQGASPAPVPYGYFGGGYYSCRVSRSSLKPCAQAATLAAYPAETPAAGEEY
PSRPTEFAFYPGYPGPYQPMASYLDVSVVQTLGAPGEPRHDSLLPVDGYQPWALTGGWNS
QMCCQGEQNPPGPFWKATFADSSTQHPPDGCSFRRGRKKRIPYSKGQLRELEREYAANKF
ITKDKRRKISAATSLSERQITIWFQNRRVKEKKVLAKVKTSATX
>gi640808225  ref_XP_008060633.1 PREDICTED: homeobox protein Hox-B13 [Carlito
MEPGNYATLDGAKDMEGLLGAGGGRNLVAHSPLASHPAAPTLMSAVNYAPLDLPGSAEPP
KQCHPCPGVPQGASPAPVPYGYFGGGYYSCRVSRSSLKPCAQAATLAAYPAETPAAGEEY
PSRPTEFAFYPGYPGTYQPMASYLDVSVVQTLGAPGEPRHDSLLPVDSYQSWALAGGWNS
QMCCQGEQNPPGPFWKAAFADSSAQHPPEGCAFRRGRKKRIPYSKGQLRELEREYAANKF
ITKDKRRKISVATSLSERQITIWFQNRRVKEKKVLAKVKNSATP
>gi556958424  ref_XP_005990116.1 PREDICTED: homeobox protein Hox-B13 [Latimeria
XXXXXENNLDENKNMEGLMGANQCRNLMAHSALGSHPSTLVHGSGYPTVEMSGSGSGEVA
KQCTPCPAVPQSSSTAPLPYGYFGSGYYSCRMGRGSLKSCTQPAALSSYYMDTPVASEDY
QARAKEFAFYHGYASPYQPVASYLDVSVVQTISGAGEPRHETLLPVDSYQPWALTNGWNS
QMYCSKDQTQPGHLWKSALADVVA-HQQDGSSFRRGRKKRIPYTKVQLKELEKEYATNKF
ITKDKRRKISAATNLSERQITIWFQNRRVKEKKVVAKIKPTTXX
>gi667260003  ref_XP_008567108.1 PREDICTED: homeobox protein Hox-B13 [Galeopterus
MEPGNYATLDGAKDIEGLLGAGGGRNLAAHSPLTSHPAAPTLMPAVNYAPLDLPGSVEPP
KQCHPCPGVPQGASPAPVPYGYFGGGYYSCRVSRSSLKPCAQAATLTAYPAETPAAGEEY
PSRPTEFAFYPGYPGPYQPMASYLDVSVVQTLGAPGEPRHDSLLPMDSYQPWALAGGWNS
QMCCQGEQNPPGPFWKAAFADASAQHPPDGCAFRRGRKKRIPYSKGQLRELEREYAANKF
ITKDKRRKISAATSLSERQITIWFQNRRVKEKKVLAKVKTSSTP
>gi831328207  ref_XP_012697304.1 PREDICTED: homeobox protein Hox-B13 [Clupea
XXXXXXXXXELNKNMEGLVGANQCRNLMAHSALGGHPSSLVHSSSYSGVDVPGSGSSEAG
KQCTPCPGVPQGSSTGPLPYGYFGNGYYPCRMGRGSLKSCTQPGALAEKYMDTPVTSEEY
PSRAKEFAFYHGYPSPYQSMASYLDVSVVQTLGT-GEPRHDTLLPMDSYQPWALTNGWGG
QMYCSKDQSQAGHLWKSALADVVA-HQHDGSSFRRGRKKRIPYTKVQLKELEKEYAANKF
ITKDKRRKISAATNLSERQITIWFQNRRVKEKKFVAKVKTSAXX
>gi642105600  emb_CDQ74032.1 unnamed protein product [Oncorhynchus mykiss]
XXXXXXXXXELNKNMEGLVGANQCRNLMAHSALGGHPSS--LVHSSGYSALDVSGSGEPG
KQCTPCPVVPQGSSTGPLPYGYFGNGYYPCRMGRGSLKSCTQPSALAEKYMDTPVTSEEY
PSRAKEFAFYHGYPSPYQSMASYLDVSVVQTLGS-GEPRHDTLLPMDSYQPWALTNGWGG
QMYCSKEQSQAGHLWKSALADVVA-HQHDGNSFRRGRKKRIPYTKMQLKELEKEYAANKF
ITKDKRRKISTGTNLSERQITIWFQNRRVKEKKFVAKVKTSAXX
>gi471380475  ref_XP_004378033.1 PREDICTED: homeobox protein Hox-B13 [Trichechus
MEPGNYATLDGAKDIEGLLGTGGGRNLVAHSPLTSHSAAPSLMPAANYAPLDLPGSAEPP
KQCHPCPGVPQGASPAPVPYGYFGGGYYSCRVSRSSLKPCAQAATLAAYPAETPAAGEEY
PSRPTEFAFYPGYPGPYQPMASYLDVSVVQTLGAPGEPRHDSLLPVDSYQPWALAGGWNS
QMCCQGEQNPPGPFWKAAFADSNAQHPPDGCAFRRGRKKRIPYSKGQLRELEREYAANKF
ITKDKRRKISAATSLSERQITIWFQNRRVKEKKVLAKVKTMATP
>gi674059771  ref_XP_008836513.1 PREDICTED: homeobox protein Hox-B13 [Nannospalax
MEHGNYATLDGAKDIEGLLGAGGGRNLVAHSPLASHPAAPTLMPAVNYAPLDLPGSAEPP
KQCHPCSGVPQGASSAPVPYGYFGGGYYSCRVSRSSLKPCAQTATLAAYPAETPAAGEEY
PSRPTEFAFYPGYPGPYQPMASYLDVSVVQTLGTPGEPRHDSLLPVDSYQPWALAGGWNS
QMCCQGEQNPPGHFWKAAFAESSAQHPPEGCAFRRGRKKRIPYSKGQLRELEREYAANKF
ITKDKRRKISAATSLSERQITIWFQNRRVKEKKVLAKVKTSATX
>gi344285419  ref_XP_003414459.1 PREDICTED: LOW QUALITY PROTEIN: homeobox protein
MEPGNYATLDGAKDIEGLLGTGGGRNLVAHSPLTSHSAAPTLMPAANYAPLDLPGSAEPP
KQCHPCPGVPQGASPAPVPYGYFGGGYYSCRVSRSSLKPCAQAATLAAYPAETPAAAEEY
PSRPTEFAFYPGYPGPYQPMASYLDVSVVQTLGGPGEPRHDSLLPVDSYQPWALAGGWNS
QMCCQGEQNAPGPFWKAAFADSNSQHPPDGCAFRRGRKKRIPYSKGQLRELEREYAANKF
ITKDKRRKISAATSLSERQITIWFQNRRVKEKKVLAKVKTITTP
```

```
>gi742113362  ref_XP_010872955.1 PREDICTED: homeobox protein Hox-B13 [Esox
XXXXXXXXXELNKNMEGLVGANQCRNLMAHSALGGHPSSLVHSSGYSSVDVSGSSSGELG
KQCTPCPAVPQGSSTAPLPYGYGFGNGYYPCRMGRGSLKSCTQPSALAEKYMDTPVTSEEY
PSRAKEFAFYHGYPSPYQSMASYLDVSVVQTLGA-GEPRHDTLLPMDSYQPWALTNGWGG
QMYCSKDQSQAGQLWKSALADVVA-HQHDGNSFRRGRKKRIPYTKVQLKELEKEYAANKF
ITKDKRRKISAVTNLSERQITIWFQNRRVKEKKFVAKVKTSAXX
>gi852732630  ref_XP_012893254.1 PREDICTED: homeobox protein Hox-B13 [Dipodomys
MEPGNYATLDGTKDIEGLLGAGGGRNLVAHSPLSSHPAAPTLMPAVNYAPLDLPGSAEPP
KQCHPCPGVPQGASPAPVPYGYFGGGYYSCRVSRSSLKPCAQTATLAAYPTETPAAGEEY
PSRPTEFAFYPGYPGPYQPMASYLDVSVVQTLGAPGEPRHDSLLPVDSYQPWALAGGWNS
QMCCQGEQNPPGPFWKAAFSDSSAQHPSDGCAFRRGRKKRIPYSKGQLRELEREYAANKF
ITKDKRRKISATTSLSERQITIWFQNRRVKEKKVLAKVKTSATP
>gi512996173  ref_XP_004859675.1 PREDICTED: homeobox protein Hox-B13
MEPGSYATLDGAKDMEGLLGAGGGRNLVAHSPLSSHPAAPTLMPAVNYAPLDLPGSPEPP
KQCHPCPGVPQGASPAPVPYGYFGGGYYSCRVSRSSLKPCAQAPTLAAYPSETPVAGEEY
PSRPTEFAFYPGYPGPYQPMASYLDVSVVQTLGAPGEPRHDSLLPVDGYQPWALAGGWNS
QMCCQGEQNPPGPFWKAAFADSNAQHPSDGCAFRRGRKKRIPYSKGQLRELEREYAVNKF
ITKDKRRKIAAATSLSERQITIWFQNRRVKEKKVLAKVKTSATP
>gi395826626  ref_XP_003786518.1 PREDICTED: homeobox protein Hox-B13 [Otolemur
MEPGNYATLDGAKDMEGLLGAGGSRNLVAHSPLTSHPATPTLMPAVNYAPLDLPGPTEPP
KQCHPCPGVPQGASPAPMPYGYFGGGYYSCRVSRSSLKPCAQAATLAAYPAETAASGEEY
PGRPTEFAFYPGYPGTYQPMASYLDVSVVQTLGAPGEPRHDSLLPVDSYQSWALAGGWNS
QMCCQGEQNPPGPFWKAAFADSSAQHPPEGCAFRRGRKKRIPYSKGQLRELEREYAANKF
ITKDKRRKISAATSLSERQITIWFQNRRVKEKKVLAKVKTSATP
>gi126308251  ref_XP_001367236.1 PREDICTED: homeobox protein Hox-B13 [Monodelphis
MESGAYATLDAGKDIEGLLGAGGCRNLVSHSPLASHPSAPALLPTANYTPIDLPGSGEPP
KQCHPCPGVPQGGSPTSVPYGYFGGGYYSCRVSRSSLKPCTQAATLAAYPTETTAAGEEY
PSRPAEFAFYPGYPGPYQPVASYLDVSVVQTLGGPGEPRHDTLLPVDSYQPWALASGWSG
QMCCQGEQNQPGHFWKTAFADSSAQHPPDGCSYRRGRKKRIPYSKGQLRELEREYAANKF
VTKDKRRKISAATSLSERQITIWFQNRRVKEKKVLAKIKTSATP
>gi528767552  gb_EPY87211.1 homeobox protein Hox-B13-like protein [Camelus ferus]
MEPGNYATLDGAKEIEGLLGTGASRNLVVHSPLTSHPAAPTLMPAVNYAPLDLPGSAEPP
KQCHPCPGVPQGAPPAPVPYGYFGGGYYSCRVSRSSLKPCAQGASLAAYPAETPASGEEY
ASRPTEFAFYPGYPGPYQPMASYLDMSVVQTLGGPGEPRHDSLLPVDSYQPWALTGGWNS
QMCCQGEQNPPGPFWKAAFADSSTQHPPDGCAFRRGRKKRIPYSKGQLRELEREYSANKF
ITKDKRRKISVATSLSERQITIWFQNRRVKEKKVLAKVKTSATP
>gi532208489  gb_JAB06503.1 homeobox protein Hox-B13 [Callithrix jacchus]
MEPGNYATLDGAKDMESLLGAGGGRNLVAHSPLTSHPAAPTLMPAVNYAPLDLPGSAEPP
KQCHPCSGVPQGASPAPVPYGYFGGGYYSCRVSRSSLKPCAQAATLAAYPAETPTAGEEY
PSRPTEFAFYPGYPGTYQPMASYLDVSVVQTLGASGEPRHDSLLPVDSYQSWALAGGWNS
QMCCQGEQNPPGPFWKAAFTDSSGQHPPDACAFRRGRKKRIPYSKGQLRELEREYAASKF
ITKDKRRKISAATSLSERQITIWFQNRRVKEKKVLAKVKNSTTP
>gi507654822  ref_XP_004633712.1 PREDICTED: homeobox protein Hox-B13 [Octodon
MEPGNYATLDGPKDMEGLLGAGGARNLVAHSPLASHPAAPTLMPAVNYAPLDLPGSAEPP
KQCHPCPGVPQGASPAPVPYGYFGGGYYSCRVSRSSLKPCAQAATLAAYPSETPAAGEEY
PSRPTEFAFYPGYPGAYQPMASYLDVSVVQTLGAPGEPRHDSLLPVDSYQPWALAGGWNS
QMCCQGEQNPPGPFWKAAFADSSAQHPSDGCAFRRGRKKRIPYSKGQLRELEREYAANKF
ITKDKRRKISAATSLSERQITIWFQNRRVKEKKVLAKVKTSATP
>gi488527225  ref_XP_004455434.1 PREDICTED: homeobox protein Hox-B13 isoform X1
MEPGNYATLDGAKDIEGLLGAGGGRNLVAHSPLTSHPAAPTLMPAASYAPLDLPGSAESS
KQCHPCPGLSQGAPSAPVPYGYFGGGYYSCRVSRSSLKPCAQAATLAAYPAETAAAGEEY
PSRPTEFAFYPGYPGPYQPMASYLDVSVVQTLGTPGEPRHDTLLPVDSYQPWALAGGWNS
QMCCQGEQNPPGPFWKATFADSSVQHPSEGCAFRRGRKKRIPYSKGQLRELEREYAANKF
ITKDKRRKIAAATSLSERQITIWFQNRRVKEKKVLAKVKTSATP
>gi817274366  ref_XP_012307257.1 PREDICTED: homeobox protein Hox-B13 [Aotus
MEPGNYATLDGPKDIEGLLGAGGGRNLVAHSPLTSHPAAATLMPAVNYAPLDLPGSAEPP
KQCHPCSGVPQGASPAPVPYGYFGGGYYSCRVSRSSLKPCAQAATLAAYPAETPTAGEEY
PSRPTEFAFYPGYPGTYQPMASYLDVSVVQTLGAPGEPRHDSLLPVDSYQSWALAGGWNS
QMCCQGEQNPPGPFWKAAFTDSSGQHPPDACAFRRGRKKRIPYSKGQLRELEREYAASKF
ITKDKRRKISAATSLSERQITIWFQNRRVKEKKVLAKVKNSATP
>gi585671866  ref_XP_006889396.1 PREDICTED: homeobox protein Hox-B13
MEPGNYATLDVAKDIEGLLGTGGGRSLVAHSPLTSHPAASALMPAANYAPLDLPGSAEPP
KQCHPCPGVPQGASPTPVPYGYFGGGYYSCRVSRSSLKPCAQAATLTAYPAETPAAGEEY
SSRPTEFAFYPGYPGPYQPMASYLDVSVVQTLGAPGEPRHDSLLPVDSYQPWALAGGWNS
QMCCQGDQNPPGPFWKAAFADANAQHPPDGCAFRRGRKKRIPYSKGQLRELEREYAANKF
ITKDKRRKISAATSLSERQITIWFQNRRVKEKKVLAKVKTSATP
>gi573901311  ref_XP_006638382.1 PREDICTED: homeobox protein Hox-B13 [Lepisosteus
XXXXXXXXXELNKNMEGLVGAGQCRNLMAHSALGGHPSTLVHGSGYSAVDVSGSGSGEPG
KQCAPCPAVPQGTSTAPLPYGYFGNGYYSCRMGRGSLKSCTQPGALSSYYMDTPVPSEEY
```

```
PSRAKEFAFYHGYASPYQPMASYLDVSVVQTISGPGETRHETLLPVDSYQPWALPNGWGS
QVYCPKDQSQAGHLWKSALADVVA-HQQDGSSFRRGRKKRIPYTKVQLKELEKEYAANKF
ITKDKRRKISAATNLSERQITIWFQNRRVKEKKFVAKVKSSTXX
>gi532071900  ref_XP_005321770.1 PREDICTED: homeobox protein Hox-B13 [Ictidomys
MEPGNYATLDGAKDIEGLLGAGGGRNLVAHSPLPSHPAAPTLMPAVNYGPLDLPGSAEPP
KQCHPCPGVPQRASPAPVPYGYFGGGYYSCRVSRSSLKPCAQAATLAAYPTETPAAGEEY
PSRPTEFAFYPGYPGPYQPMASYLDVSVVQTLGAPGEPRHDSLLPVDSYQPWALASGWNS
QMCCQGEQNPPGPFWKAAFADSSAQHPSEGCAFRRGRKKRIPYSKGQLRELEREYAANKF
ITKDKRRKISAATSLSERQITIWFQNRRVKEKKVLAKVKTSATP
>gi114149320  sp_Q1ECY2.1HXBDA_DANRE RecName: Full=Homeobox protein Hox-B13a
XXXXXENNLDELKNMEGLVGANQCRNLMAHSALSGHPSS--LVHGSSYPTVDVSSSAESG
KQCTPCPTVPQASSTGPIPYGYFGNSYYPCRMGRGSLKSCTQPSALAEKYMDTPVTSEEY
PTRAKEFAFYHSYPSPYQSMASYLDVSVVQTLGT-GEPRHDSLLPMDSYQPWALANGWGS
QMYCSKDQGQAGHLWKSALADVVA-HQHDGGSFRRGRKKRIPYTKVQLKELEKEYAANKF
ITKDKRRKISAVTNLSERQITIWFQNRRVKEKKFIAKVKNTAXX
>gi593727116  ref_XP_007109522.1 PREDICTED: homeobox protein Hox-B13 [Physeter
MEPGNYATLDGAKEIEGLLGAGGSRNLVAHSPLTSHPAAPTLMPAVNYAPLDLPGSAEPP
KQCHPCPGVPQGASPAPVPYGYFGGGYYSCRVSRSSLKPCAQAATLAAYPAETPAAGEEY
PSRPTEFAFYPGYPGPYQPMASYLDVSVVQTLGAPGEPRHDSLLPVDSYQPWALTGGWNS
QMCCQGEQNPPGPFWKAAFADSSAQHPPDGCAFRRGRKKRIPYSKGQLRELEREYSANKF
ITKDKRRKISAATSLSERQITIWFQNRRVKEKKVLAKVKTSAIP
>gi470606860  ref_XP_004314391.1 PREDICTED: homeobox protein Hox-B13 [Tursiops
MEPGNYATLDGAKEIEGLLGAGGSRNLVAHSPLTSHPAAPTLMPAVNYAPLDLPGSAEPP
KQCHPCPGVPQGASPAPVPYGYFGGGYYSCRVSRSSLKPCAQAATLAAYPAETPAAGEDY
PSRPTEFAFYPGYPGPYQPMASYLDVSVVQTLGAPGEPRHDSLLPVDSYQPWALTGGWNS
QMCCQGEQNPPGPFWKAAFADSSAQHPPDGCAFRRGRKKRIPYSKGQLRELEREYSANKF
ITKDKRRKISAATSLSERQITIWFQNRRVKEKKVLAKVKTSAIP
>gi291405863  ref_XP_002719356.1 PREDICTED: homeobox protein Hox-B13 [Oryctolagus
MESGNYATLDGAKDIEGLLGAGGGRNLVAHSPLSSHPAAPALMPAVNYAPLDLPGPAEPP
KQCHPCPGVPQGASPAPVPYGYFGGGYYSCRVSRSSLKSCAQAPSLAAYPAETPAAGEEY
PSRPTEFAFYPGYPGPYQPMASYLDVSVVQTLGAPGEPRHDSLLPVDSYQPWALAGGWNS
QMCCQGEQNPPGPFWKAAFADASAQHPPDGCAFRRGRKKRIPYSKGQLRELEREYAANKF
ITKDKRRKISAATSLSERQITIWFQNRRVKEKKVLAKVKTSATP
>gi594656397  ref_XP_007176570.1 PREDICTED: homeobox protein Hox-B13
MEPGNYATLDGAKEIEGLLGAGGSRNLVAHSPLTSHPAAPTLMPAVNYAPLDLPGSAEPP
KQCHPCPGVPQGASPAPVPYGYFGGGYYSCRVSRSSLKPCAQAATLAAYPAETPAAGEEY
PSRPTEFAFYPGYPGPYQPMASYLDVSVVQTLGAPGEPRHDSLLPVDSYQPWALTGGWNS
QMCCQGEQNPPGPFWKAAFADSSAQHPPDGCAFRRGRKKRIPYSKGQLRELEREYSANKF
ITKDKRRKISAATSLSERQITIWFQNRRVKEKKVLAKVKTSAIP
>gi634851243  ref_XP_007940148.1 PREDICTED: homeobox protein Hox-B13 [Orycteropus
MDPGNYATLDVAKDIEGLLATGGSRHLVAHSPLTSHPAAPTLMPAANYAPLDLPGSAEQP
KQCHPCPGVPQGASPAPVPYGYFGGGYYSCRVSRSSLKPCAQAATLAAYPAETPAAGEEY
PSRPTEFAFYPGYPGPYQPMASYLDVSVVQTLGAPGEPRHDSLLPVDSYQPWALAGSWNS
QMCCQGEQNQPGPFWKAAFADSNAQHPPDGCAFRRGRKKRIPYSKGQLRELEREYAANKF
ITKDKRRKISAATSLSERQITIWFQNRRVKEKKVLAKVKTSATX
>gi602709820  ref_XP_007465163.1 PREDICTED: homeobox protein Hox-B13 [Lipotes
MEPGNYATLDGAKEIEGLLGAGGSRNLVAHSPLTSHPAAPTLMPAVNYAPLDLPGSAEPP
KQCHPCPGVPQGASPAPVPYGYFGGGYYSCRVSRSSLKPCAQAATLAAYPAETPAAGEEY
PSRPTEFAFYPGYPGPYQPMASYLDVSVVQTLGAPGEPRHDSLLPVDSYQPWALTGGWNS
QMCCQGEQNPPGPFWKAAFADSSAQHPPDGCAFRRGRKKRIPYSKGQLRELEREYSANKF
ITKDKRRKISAATSLSERQITIWFQNRRVKEKKVLAKVKTSGIP
>gi826271801  ref_XP_012515604.1 PREDICTED: homeobox protein Hox-B13 [Propithecus
MELGNYATLDGAKDMEGLLGAGGGRNLVAHSPLTSHPATPTLMPAVNYAPLDLPGSAEPP
KQCHPCPGVPQGASPAPVPYGYFGGGYYSCRVSRSSLKPCAQGATLAAYPAETAAAGEEY
PGRPTEFAFYPGYPGTYQPMASYLDVSVVQTLGAPGEPRHDSLLPVDSYQSWALAGGWNS
QMCCQGEQNPPGPFWKAAFADSSAQHPPEGCAFRRGRKKRIPYSKGQLRELEREYSANKF
ITKDKRRKISAATSLSERQITIWFQNRRVKEKKVLAKVKTSAIP
>gi589917310  ref_XP_006971969.1 PREDICTED: homeobox protein Hox-B13 [Peromyscus
MEPGNYATLDGAKDIEGLLGAGGGRNLVPHSPLANHPAAPTLMPAVNYAPLDLPGSAEPP
KQCHPCPGVPQGASPAPVPYGYFGGGYYSCRVSRSSLKPCAQTATLATYPSETPAPGEEY
TSRPTEFAFYPGYPGPYQPMASYLDVSVVQTLGAPGEPRHDSLLPVDSYQPWALAGGWNS
QMCCQGEQNPPGPFWKAAFAEPNTQHPPDGCAFRRGRKKRIPYSKGQLRELEREYAANKF
ITKDKRRKISAATSLSERQITIWFQNRRVKEKKVLAKVKTSATP
>gi507543189  ref_XP_004655586.1 PREDICTED: homeobox protein Hox-B13 [Jaculus
MEPGTYATLDGAKDIEGLLGTGGGRNLAAHSPLASHPAAPTLMPAVNYAPLDLPGSAEPP
KQCHPCPGVPQGASPAPVPYGYFGGGYYSCRVSRSSLKPCAQAATLTAYPAETPAPGEEY
PSRPTEFAFYPGYPGTYQPMASYLDVSVVQTLGTPGEPRHDSLLPVDSYQPWALAGGWNS
QMCCQGEQNPPGPFWKAAFAESSVQHPPDGCAFRRGRKKRIPYSKGQLRELEREYAANKF
ITKDKRRRISAATSLSERQITIWFQNRRVKEKKVLAKVKSSATP
```

>gi395532657 ref_XP_003768386.1 PREDICTED: homeobox protein Hox-B13 [Sarcophilus
MESGAYATLDAGKDIEGLLGAGGCRNLVSHSPLASHPSASALLPTPNYTSIDLPGSGDPP
KQCHPCPGMPQGGSATSVPYGYFGGGYYSCRVSRSSLKPCTQAATLAAYPTETTAAGEEY
SSRPAEFAFYPGYPGPYQPVASYLDVSVVQTLGGPGEPRHDTLLPVDSYQPWALASGWSG
QMCCQGEQNQPGHFWKAAFADASAQHPQDGCSYRRGRKKRIPYSKGQLRELEREYAANKF
VTKDKRRKISAATSLSERQITIWFQNRRVKEKKVLAKIKTSATP
>gi597740267 ref_XP_007232293.1 PREDICTED: homeobox protein Hox-B13 [Astyanax
XXXXXXXXXELNKNMEGLVGANQCRNLMAHSALSAHPSSLVHGSSYSTVEVSTSSAAETS
KQCTPCPTVPQASSTGPIPYGYFGNSYYPCRMGRGSLKSCTQPSALAEKYMDTPVTSEEY
PSRAKEFAFYHGYPSPYQSMASYLDVSVVQTLGT-GEPRHDSLLPMDSYQPWALANGWGG
QMYCSKDQSQAGHLWKSALADVVA-HQHEGGSFRRGRKKRIPYTKVQLKELEKEYAANKF
ITKDKRRKISAVTNLSERQITIWFQNRRVKEKKFIAKVKTSAXX
>gi725576032 ref_XP_010340157.1 PREDICTED: homeobox protein Hox-B13 [Saimiri
MEPGNYATLDGAKDIEGLLGAGGGRNLVAHSPLTSHPAAPTLMPAVNYAPLDLPGSAEPP
KQCHPCSGAPQGASPAPVPYGYFGGGYYSCRVSRSSLKPCAQAGTLAAYPAETPTAGEEY
PSRPTEFAFYPGYPGTYQPMASYLDVSVVQTLGAPGEPRHDSLLPVDSYQSWALAGGWNS
QMCCQGEQNPPGPFWKAAFTDSSGQHLPDACAFRRGRKKRIPYSKGQLRELEREYAASKF
ITKDKRRKISAATSLSERQITIWFQNRRVKEKKVLAKVKNSATP
>gi742135928 ref_XP_010841045.1 PREDICTED: homeobox protein Hox-B13 [Bison bison
MEPGNYTTLDGAKEIEGLLGAGGSRNLVTHSPLTSHPSAPTLMPAVNYAPLDLPGSAEPP
KQCHPCPGVPQGASPAPVPYGYFGGGYYSCRVSRSSLKPCAQAATLAAYPAETPTSGEEY
PSRPTEFAFYPGYPGPYQPMASYLDVSVVQTLGAPGEPRHDSLLPVDSYQPWALTGGWNS
QMCCQGEQNPPGPFWKAAFADSSSQHPPEGCTFRRGRKKRIPYSKGQLRELEREYSANKF
ITKDKRRKISAATSLSERQITIWFQNRRVKEKKVLAKVKTSATP
>gi348562271 ref_XP_003466934.1 PREDICTED: homeobox protein Hox-B13 [Cavia
MEPGNYATLDGTKEMEGLLGAGGARNLVAHSPLASHPAAPTLMPAVNYAPLDLPGSGEPP
KQCHPCPGVPQGASPAPVPYGYFGGGYYSCRVSRSSLKPCAQAGTLAAYPSDTPAAGEEY
PSRPTEFAFYPGYPGAYQPMASYLDVSVVQTLGTPGEPRHDSLLPVDGYQPWALASGWNS
QMCCQGDQNPPGPFWKAAFAESSVQHPSDGCAFRRGRKKRIPYSKGQLRELEREYAANKF
ITKDKRRKISAATSLSERQITIWFQNRRVKEKKVLAKVKTSATP
>gi594050844 ref_XP_006049766.1 PREDICTED: homeobox protein Hox-B13 [Bubalus
MEPGNYTTLDGAKEIEGLLGAGGSRNLVTHSPLTSHPAAPTLMPAVNYAPLDLPGSAEPP
KQCHPCPGVPQGASPAPVPYGYFGGGYYSCRVSRSSLKPCAQAATLAAYPAETPTSGEEY
PSRPTEFAFYPGYPGPYQPMASYLDVSVVQTLGAPGEPRHDSLLPVDSYQPWALTGGWNS
QMCCQGEQNPPGPFWKAAFADSSSQHPPEGCAFRRGRKKRIPYSKGQLRELEREYSANKF
ITKDKRRKISAATSLSERQITIWFQNRRVKEKKVLAKVKTSTTX
>gi533160875 ref_XP_005394290.1 PREDICTED: homeobox protein Hox-B13 [Chinchilla
MEPGNYATLDGAKDMEGLLGAGGARNLVAHSPLASHPAAPTLMPAVNYAPLDLPGSAEPP
KQCHPCPGVPQGASPAPVPYGYFGGGYYSCRVSRSSLKPCAQAATLAAYPSETPAAGEEY
PSRPTEFAFYPGYPGAYQPMASYLDVSVVQTLGAPGEPRHDPLLPGDSYQPWALASGWSS
QMCCQGEQNPPGPFWKAAFADSSSQHPSDGCAFRRGRKKRIPYSKGQLRELEREYAANKF
ITKDKRRKISAATSLSERQITIWFQNRRVKEKKVLAKVKTSATP
>gi556738344 ref_XP_005964747.1 PREDICTED: homeobox protein Hox-B13 [Pantholops
MEPGNYTTLDGAKEIEGLLGAGGSRNLVTHSPLTSHPAAPTLLPAVNYAPLDLPGSAEQP
KQCHPCSGVPQGASPAPVPYGYFGGGYYSCRVSRSSLKPCAQAATLAAYPAETPASGEEY
PSRPTEFAFYPGYPGPYQPMASYLDVSVVQTLGAPGEPRHDSLLPVDSYQPWALTGGWNS
QMCCQGEQNPPGPFWKAAFADSSAQHPPDGCAFRRGRKKRIPYSKGQLRELEREYSANKF
ITKDKRRKISAATSLSERQITIWFQNRRVKEKKVLAKVKTSATP
>gi803246204 ref_XP_011983097.1 PREDICTED: homeobox protein Hox-B13 [Ovis aries
MEPGNYTTLDGAKEIEGLLGAGGSRNLVTHSPLTSHPAAPTLLPAVNYGPLDLPGSAEPP
KQCHPCPGMPQGASPAPVPYGYFGGGYYSCRVSRSSLKPCAQAATLAAYPAETPASGEEY
PSRPTEFAFYPGYPGPYQPMASYLDVSVVQTLGAPGEPRHDSLLPVDSYQPWALTGGWNS
QMCCQGEQNPPGPFWKAAFADSSAQHPPDGCAFRRGRKKRIPYSKGQLRELEREYSANKF
ITKDKRRKISAATSLSERQITIWFQNRRVKEKKVLAKVKTSATP
>gi354474760 ref_XP_003499598.1 PREDICTED: homeobox protein Hox-B13 [Cricetulus
MEHGNYATLDGAKDIEGLLGAGGGRNLVAHSPLASHSATPTLMPTVNYAPLDLPGSAEPP
KQCHPCPGVPQGASPAPVPYGYFGGGYYSCRVSRSSLKPCAQTATLATYPSETPAPGEEY
PGRPTEFAFYPGYPGPYQPMASYLDVSVVQTLGAPGEPRHDSLLPVDSYQPWALASGWNS
QMCCQGEQNPPGPFWKAAFAEPSTQHPPDGCAFRRGRKKRIPYSKGQLRELEREYATNKF
ITKDKRRKISAATSLSERQITIWFQNRRVKEKKVLAKVKTSATP
>gi532051321 ref_XP_005368419.1 PREDICTED: homeobox protein Hox-B13 [Microtus
MEPGNYATLDGAKDIEGLLGPGGSRNLVAHSPLASHPAAPTLMPTVNYAPLDLPGSAEPP
KQCHPCPGVPQGASPAPVPYGYFGGGYYSCRVSRSSLKPCAQTATLATYPSETPASGEEY
PSRPTEFAFYPGYPGPYQPMASYLDVSVVQTLGAPGEPRHDSLLPVDSYQPWALAGGWNS
QMCCQGEQNPPGPFWKATFAEPSTQHPPDGCAFRRGRKKRIPYSKGQLRELEREYAANKF
ITKDKRRKISAATSLSERQITIWFQNRRVKEKKVLAKVKTGATP
>gi311267502 ref_XP_003131599.1 PREDICTED: homeobox protein Hox-B13 [Sus scrofa]
MEPGNYATLDGAKEIEGLLGAGGSRNLVAHSPLTSHPAAPTLMPAVNYAPLDLPGSAEPP
KQCHPCSGVPQGASPAPVPYGYFGGGYYSCRVSRSSLKPCAQGATLAAYPAETPTAGEEY

```
PSRPTEFAFYPGYPGPYQPMASYLDVSVVQTLGTPAEPRHDSLLPVDSYQPWALTGGWNS
QVCCQGEQNPSGSFWKAAFADSSAQHPPEGCAFRRGRKKRIPYSKGQLRELEREYSANKF
ITKDKRRKISAATSLSERQITIWFQNRRVKEKKVLAKVKTSATP
>gi743729506  ref_XP_010958844.1 PREDICTED: homeobox protein Hox-B13 [Camelus
MEPGNYATLDGAKEIEGLLGTGASRNLVVHSPLTSHPAAPTLMPAVNYAPLDLPGSAEPP
KQCHPCPGVPQGAPPAPVPYGYFGGGYYSCRVSRSSLKPCAQGASLAAYPAETPTSGEEY
ASRPTEFAFYPGYPGPYQPMASYLDMSVVQTLGGPGEPRHDSLLPVDSYQPWALTGGWNS
QMCCQGEQNPPGPFWKAAFADSSTQHPPDGCAFRRGRKKRIPYSKGQLRELEREYSANKF
ITKDKRRKISVATSLSERQITIWFQNRRVKEKKVLAKVKTSATP
>gi359754103  gb_AEV59525.1 HOXB13 [Macropus eugenii]
MESGAYATLDAGKDIEGLLGAGGCRNLVSHSPLASHPSAPALLPTPNYTPIDLPGSGEPP
KQCHPCPGVPQGGSPTSVPYGYFGGGYYSCRVSRSSLKPCTQAATLAAYPPETTAAGEDY
PSRPAEFAFYPGYPGPYQPVASYLDVSVVQTLGGPGEPRHDTLLPVDSYQPWALASGWSG
QMCCQGEQNQPGHFWKAAFADASAQHPPDGCSYRRGRKKRIPYSKGQLRELEREYAANKF
VTKDKRRKISAATSLSERQITIWFQNRRVKEKKVLAKIKTNATP
>gi576886849  gb_AHH38096.1 Homeobox protein Hox-B13a [Ictalurus punctatus]
XXXXXXXXXELNKNMEGLVGANQCRNLIAHSALSGHPSSLVHGSGYSTVDVSTSSSGETS
KQCTPCPTVPQASSTGPIPYGYFGNSYYPCRMGRGSLKPCTQPSALAEKYMDTPVTSEEY
PTRAKEFAFYHGYHGPYQSMASYLDVSVVQTLGT-SEPRHDSLLPMDSYQPWALANGWGG
QMYCSKDQSQAGHLWKSALADVVA-HQHDGGSFRRGRKKRIPYTKVQLKELEKEYAANKF
ITKDKRRKISAVTNLSERQITIWFQNRRVKEKKFVAKVKSSAXX
>gi586448761  ref_XP_006832561.1 PREDICTED: homeobox protein Hox-B13
MEPGNYATLDMAKDMEGLLGTGGGRNLVTHSPLTSHPAAPTLMPAANYAPLDLPGSAEPS
KQCHPCPGVPQGASPAPVPYGYFGGGYYSCRVSRSSLKPCAQGTTLAAYPPDTPSAGEEY
PSRPTEFAFYPGYPGPYQPMASYLDVSVVQTLGAPGEPRHDSLLPVDSYQPWALAGSWNS
QMCCQGEQTPPGPFWKAAFADSNTQPPSDGCTFRRGRKKRIPYSKGQLRELEHEYAANKF
ITKEKRRKISAATSLSERQITIWFQNRRVKEKKVLAKVKTSMTP
>gi504159820  ref_XP_004591147.1 PREDICTED: homeobox protein Hox-B13 [Ochotona
MEPGNYATLDGAKDIEGLLGAGGGRNLVSHSPLSSHPAAPALVAAANYAPLDLPGSAEPP
KQCHPCPGMPQGASPAPVPYGYFGGGYYSCRVSRSSLKPCAQAPSLTAYPAETPAAGEEY
PSRPAEFAFYPGYPGPYQPVASYLDVSVVQTLGAPGEPRHDPLLPVDSYQPWALAGGWNS
QMCCQGEQNPPGPFWKTAFADAGAQHPPDGCAFRRGRKKRIPYSKGQLRELEREYAANKF
ITKDKRRKISAATSLSERQITIWFQNRRVKEKKVLAKVKTSATP
>gi524951862  ref_XP_005075932.1 PREDICTED: homeobox protein Hox-B13
MEPGNYATLDGAKDIEGLLGAG--RNLVAHSPLASHPAAPTLMPTVNYAPLDLPGSAEPP
KQCHPCPGVPQGASPAPVPYGYFGGGYYSCRVSRSSLKPCAQTATLATYPSETPAPGEEY
PSRPTEFAFYPGYPGPYQPMASYLDVSVVQTLGAPGEPRHDSLLPVDSYQPWALAGGWNS
QMCCQGEQNPPGPFWKAAFAEPSTQHPPDGCAFRRGRKKRIPYSKGQLRELEREYAANKF
ITKDKRRKISAATSLSERQITIWFQNRRVKEKKVLAKVKTSATP
>gi332692483  gb_AEE90163.1 Homeobox B13a [Anguilla anguilla]
XXXXXXXXXELNKNMEGLVGASQCRNLMAHSALGGHPSS--LVHSYTAGDVSGSGSGEAG
KQCAPCPTVPQSSSTTPLPYGYFGNSYYPCRMGRGSLKSCTQPSPLAEKYMDTPVTSEEY
SNRTKEFAFYHGYPSPYQPVASYLDVSVVQTLGT-GEPRHETLLPMDSYQPWALTNGWGS
QMYCAKDPSQSGNLWKSALVDVVA-HQHDGGSFRRGRKKRIPYTKVQLKELEKEYTANKF
ITKDKRRKISAVTNLSERQITIWFQNRRVKEKKLSAKVKNIAXX
>gi385654474  gb_AFI61975.1 Hox-B13a [Anguilla japonica]
XXXXXXXXXELNKNMEGLVGASQCRNLMAHSALGGHPSS--LVHSYTAGDVSGSGSGEAG
KQCAPCPTVPQSSSTTPLPYGYFGNSYYPCRMGRGSLKSCTQPSPLAEKYMDTPVTSEEY
SNRTKEFAFYHGYPSPYQPVASYLDVSVVQTLGT-GEPRHETLLPMDSYQPWALTNGWGS
QMYCAKDPSQSGNLWKSALVDVVA-HQHDGGSFRRGRKKRIPYTKVQLKELEKEYTANKF
ITKDKRRKISAVTNLSERQITIWFQNRRVKEKKLSAKVKNVAXX
>gi478520959  ref_XP_004434692.1 PREDICTED: homeobox protein Hox-B13
MEPGNYATLDGAKDIEGLLGAGGSRNLVAHPSLTSHPAAPTLMPAVNYAPLDLPGSAEPP
KQCHPCPGVPQGASPAPVPYGYFGGGYYSCRVSRSSLKPSAPAATLAAYPTETPAAGEEY
PSRPTEFAFYPGYPGPYQPMASYLDVSVVQTLGAPGEPRHDSLLPVDSYQPWALTGGWNS
QMCCQGEQNPPGPFWKAAFADSSAQHPPDGCAFRRGRKKRIPYSKGQLRELEREYSANKF
ITKDKRRKISAATSLSERQITIWFQNRRVKEKKVLAKVKTSTTP
>gi301128881  emb_CBL59344.1 HoxB13 [Scyliorhinus canicula]
XXXXXXXXXXXXXXMEGFVGAGQCRNLMAHSALGAHPSP--LVHGSGYSTVDVPGSGEPG
KQCTPCP-MPQASTAAPLPYGYFGSGYYSCRMGRNSIKPCTQPAALSSYYMDSPVPTDDY
PGRPKEFAFYHGYASAYQPMPSYLDVSVVQTISGAGEPRHEALLPMEGYQPWAL-NGWNS
QMCCSKDQAQSGHLWKSTLAD-VAVHQQDGSSFRRGRKKRIPYTKVQLKELEREYATNKF
ITKDKRRKISAATNLSERQITIWFQNRRVKEKKVIAKVKTNTXX
>gi1011609041  gb_KYO44343.1 homeobox protein Hox-B13 [Alligator mississippiensis]
MDPGTFTSHDSNKSMEGLMGAGQCRNLVAHSALGG-PSPPTLMHSSNYPTMDAAGPGEPS
KQCIPCPAVPQGSSTTPLPYGYFGSGYYSCRVARSSLKPCPQSSYPAEKYVDTPAAGEEF
QPRPTDFAFYPGYAGPYQPMASYLDVSVVQTMGGPGEARHETLLPVDSYQPWALTSGWNS
QLCCPKDQSQAGHFWKSAFADAVAQHPPDGCSFRRGRKKRIPYSKGQLKELEKEYASNKF
ITKDKRRKISAVTNLSERQITIWFQNRRVKEKKVIAKIKSSSTP
```

```
>gi664747354  ref_XP_008531051.1 PREDICTED: homeobox protein Hox-B13 [Equus
MEPGNYATLDGAKDIEGLLGAGGSRNLVAHSPLTSHPAAPTLMPAVNYAPLDLPGSAEPP
KQCHPYPGVPQGASPAPVPYGYFGGGYYSCRVSRSSLKPSAQAATLTAYPTETPATGEEY
PSRPTEFAFYPGYPGPYQPMASYLDVSVVQTLGAPGEPRHDSLLPVDSYQPWALTGGWNS
QMCCQGEQNPPGPFWKAAFADSSAQHPADGCGFRRGRKKRIPYSKGQLRELEREYSANKF
ITKDKRRKISAATSLSERQITIWFQNRRVKEKKVLAKVKTSATP
>gi557280818  ref_XP_006023393.1 PREDICTED: homeobox protein Hox-B13 [Alligator
MDPGTFTSHDSNKSMEGLMGAGQCRNLVAHSALGG-PSPSTLMHSSNYPTMDAAGPGEPS
KQCIPCPAVPQGSSTTPLPYGYFGSGYYSCRVARSSLKPCPQSSYPAEKYVDTPAAGEEF
QPRPTDFAFYPGYAGPYQPMASYLDVSVVQTMGGPGEARHETLLPVDSYQPWALTSGWNS
QLCCPKDQSQAGHFWKSAFADAVAQHPPDGCSFRRGRKKRIPYSKGQLKELEKEYASNKF
ITKDKRRKISAVTNLSERQITIWFQNRRVKEKKVIAKIKSSSTP
>gi359076815  ref_XP_003587468.1 PREDICTED: homeobox protein Hox-B13 [Bos taurus]
MEPSNYTTLDGAKEIEGLLGAGGSRNLVTHSPLTSHPSAPTLMPAVNYAPLDLPGSAEPP
KQCHPCPGVPQGASPAPVPYGYFGGGYYSCRVSRSSLKPCAQAATLAAYPAETPTSGEEY
PSRPTEFAFYPGYPGPYQPMASYLDVSVVQTLGAPGEPRHDSLLPVDSYQPWALTGGWNS
QMCCQGEQNPPGPFWKAAFADSSSQHPPEGCTFRRGRKKRIPYSKGQLRELEREYSANKF
ITKDKRRKISAATSLSERQITIWFQNRRVKEKKVLAKVKTSAPP
>gi632972216  ref_XP_007902551.1 PREDICTED: homeobox protein Hox-B13 isoform X2
XXXXXXAVMEGFVNT-GNFSANQCRNLMAHSALGGHPAP--LVHGSGYSTVDMSGSGEPG
KQCAPCPPLPQGSPAAPLPYGYFGSGYYSCRMGRGPIKSCTQPAALGDKYMDTPVPTDDY
PGRAKEFAFYHGYAGPYQPMPSYLDVSVVQTISGAGEPRHEALLPMDSYQPWAL-NGWNS
QMYC-KEQAQPPHLWKSTLADVVG-HQQDGGSFRRGRKKRIPYTKVQLKELEREYGTNKF
ITKDKRRKISAATNLSERQITIWFQNRRVKEKKIVAKVKSNTTX
>gi759185571  ref_XP_011380955.1 PREDICTED: homeobox protein Hox-B13 [Pteropus
MEPGSYATLDGAKDIEGLLGVGASRNLVAHSPLTSLPAAPTLMPAVNYAPLDLPGSAEPP
KQCHPCPGVPQGASPAPVPYGYFGGGYYSCRVSRSSLKPCAQAAALTAYPAETPTAGEEY
PSRPTEFAFYPGYPGPYQPMASYLDVSVVQTLGAPGEPRHEALLPMDSYQPWALTSGWNS
QMCCQGEQNPPGPFWKATCTDSSAQHPPDSCTIHRGRKKRIPYSKGQLRELEREYSANKF
ITKDKRRKISAVTSLSERQITIWFQNRRVKEKKVLTKVKTSATX
>gi472350967  ref_XP_004395168.1 PREDICTED: homeobox protein Hox-B13 [Odobenus
MEPGSYAGLDGAKDMEGLPGAEGSRNLVAHSPPTSHPAAPTLMPAVSYAPLDLPGSAEPP
KQCHSCPGLPQGAAPAPVPYGYFGGGYYSCRVSRSSLKPCAQAATLAAYPAETATAGEEY
PGRPTEFAFYPGYPGPYQPMASYLDVSVVQTLGAPGEPRHDSLLPVDSYQPWALTGGWNG
QMCCQGEQNPPGPFWKATFAESGAQHLPEGCTFRRGRKKRIPYSKGQLHELEREYSANKF
ITKDKRRKISAATSLSERQITIWFQNRRVKEKKVLAKVKTSTTP
>gi586539979  ref_XP_006924825.1 PREDICTED: homeobox protein Hox-B13 [Pteropus
MEPGSYATLDGAKDIEGLLGVGASRNLVAHSPLTSLPAAPTLMPAVNYAPLDLPGSAEPP
KQCHPCPGVPQGASPAPVPYGYFGGGYYSCRVSRSSLKPCAQAATLTAYPAETPTAGEEY
PSRPTEFAFYPGYPGPYQPMASYLDVSVVQTLGAPGEPHHEALLPMDSYQPWALTSGWNS
QMCCQGEQNPPGAFWKATCTDSSAQHPPDSCTIHRGRKKRIPYSKGQLRELEREYSANKF
ITKDKRRKISAVTSLSERQITIWFQNRRVKEKKVLAKVKTSATP
>gi157823841  ref_NP_001100511.1 homeobox protein Hox-B13 [Rattus norvegicus]
MEPGNYATLDGAKDIEGLLGAGGGRNLVAHSPLASHPAAPTLMPTVNYAPLDLPGSAEPP
KQCHPCPGVPQGASPAPVPYGYFGGGYYSCRVSRSSLKPCAQTATLATYPSETPAPGEEY
PSRPTEFAFYPGYPGPYQPMASYLDVSVVQTLGAPGEPRHDSLLPVDSYQPWALAGGWNS
QMCCQGEQNPPGPFWKAAFAEPSVQHPPDGCAFRRGRKKRIPYSKGQLRELEREYAANKF
ITKDKRRKISAATSLSERQITIWFQNRRVKEKKVLAKVKTSTTP
>gi410980861  ref_XP_003996792.1 PREDICTED: homeobox protein Hox-B13 [Felis
MEPGNYATLDGAKDMDGLPGAGGSRNLVAHSPLTSHPAAPTLMPAVSYAPLDLPGSAEPP
KQCHPCPGVPQGAAPAPVPYGYFGGGYYSCRVSRSSLKPCAQAATLAAYPAETAAAGEEY
PSRPTEFAFYPGYSGPYQPMASYLDVSVVQTLGAPGEPRHDSLLPVDSYQSWALTGGWNS
QMCCQGEQNPPGPFWKAAFADTSAQHPPEGCAFRRGRKKRIPYSKGQLRELEREYSANKF
ITKDKRRKISAATSLSERQITIWFQNRRVKEKKVLAKVKTSTTP
>gi829772881  ref_XP_012621526.1 PREDICTED: homeobox protein Hox-B13 [Microcebus
MELGNYATLDGAKDMEGLLGAGGGRNLVAHSPLTSHPATPTLMPAVNYAPLDLPGSAEPP
KQCHPCPGVPQGASPAPVPYGYFGGGYYSCRVSRSSLKPCAQAATLAAYPAETAAAAGEY
PGRPTEFAFYPGYPGTYQPMASYLDVSVVQTLGAPGEPRHDSLLPVDSYQSWALAGGWNS
QMCCQGEQNPPGPFWKAAFADSSAQHPSEGCAFRRGRKKRIPYSKGQLRELEREYSANKF
ITKDKRRKISAATSLSERQITIWFQNRRVKEKKVLAKVKTSATP
>gi220898176  gb_ACL81432.1 HoxA13 [Latimeria menadoensis]
XXXXXXXXXEVNKNMEGFPGGNFAANQCRN--LMAHPASLAPSTAYTSSEVPASGIAEPV
KQCSPCSAAQSSSSA-SLPYGYFGSSYYPCRMTHHNIKSCAQPSTFAEKYMDTSVSGEEF
TSRAKEFAFYQGYAGPYQPVPSYLDVPVVPTIGGPGEPRHEPLLPMETYQPWAITNGWNG
QVYCSKEQAQPNHLWKSTLPDVVS-HPSDANSFRRGRKKRVPYTKVQLKELEREYATNKF
ITKDKRRRISATTNLSERQVTIWFQNRRVKEKKVINKLKTTSXX
>gi1519038  gb_AAC52769.1 Hoxb-13 [Mus musculus]
MEPGNYATLDGAKDIEGLLGAGGGRNLVSHSPLASHPAAPTLMPTVNYAPLDLPGSAEPP
KQCHPCPGVPQGASPAPVPYGYFGGGYYSCRVSRSSLKPCAQTAALATYPSETPAPGEEY
```

PSRPTEFAFYPGYPGPYQPMASYLDVSVVQTLGAPGEPRHDSLLPVDSYQPWALAGGWNS
QMCCQGEQNPPGPFWKAAFAEPSVQHPPDGCAFRRGRKKRIPYSKGQLRELEREYAANKF
ITKDKRRKISAATSLSERQITIWFQNRRVKEKKVLAKVKTSTTP
>gi731207261 ref_XP_010618416.1 PREDICTED: homeobox protein Hox-B13 isoform X1
MEPGGYATLDGAKDMEGLLGAGGGRNLLAHSPLSSHPAAPALMPAVNYAPLDLPGSAEPP
KQCHPCPGVPQGGSPAPVPYGYFGGGYYSCRVSRSSLKPCAQAAALAAYPSEPPAAGEEY
PSRPTEFAFYPGYPGAYQPMASYLDVSVVQTLGAPGEPRHDTLLPVDGYQPWALAGGWNS
QMCCQGEQNPPGPFWK-AFADSSAPHPSDGCAFRRGRKKRIPYSKGQLRELEREYAANKF
ITKDKRRKISAATSLSERQITIWFQNRRVKEKKVLAKVKTSATP
>gi20278975 gb_AAM18671.1AF479755_1 homeobox A13 [Heterodontus francisci]
XXXXXXXXXEINKNMDGFSGGNFTANQCRN--LMAHPASLAPSSAYTSSEVPASGIAEPV
KQCSPCPAA-QTSSSAALPYGYFGSSYYPCRMSHHSIKSCAQPSTLSGYPADKTSAGEEF
TSRAKEFAFYQGYAGPYQPMPSYLDVPVVPTISAPGEPRHDTLLPMESYQPWAITNGWNG
QVCCSKEQPQATHLWKSSLPDVVS-HPSDASSFRRGRKKRVPYTKVQLKELEREYATNKF
ITKDKRRRISASTNLSERQVTIWFQNRRVKEKKVLTKLKSNSXX
>gi585176637 ref_XP_006740078.1 PREDICTED: homeobox protein Hox-B13-like
MEPGSYAALDGAKDLEVLPGAGGSRHLVAHCPPTGHPAAPTLMPAVSHAPLDLPGSAEPP
KQCQPCPGLPQGAAPAPVPYGYFGGGYYSCRVSRSSLKPCAQAAPLAAYSAETATAGEQY
PSRPTEFAFYPGYPGPYQPMASYLDVSVVQTLGAPGEPRHESLLPVDSYQPWALTGGWNG
QMCCQGEQNPPGPFWKAAFAESGTQHPPDGCTFRRGRKKRIPYSKGQLRELEREYSANKF
ITKDKRRKISAATSLSERQITIWFQNRRVKEKKVLAKVKTSTTP
>gi560984071 ref_XP_006214251.1 PREDICTED: homeobox protein Hox-B13 [Vicugna
MEPGSYATLDGVKEIEGLLGTGASRNLVVHSPLTSHPAAPTLMPAVNYAPLDLPGSAEPP
KQCHPCPGVPQGPPPAPVPYGYFGGGYYSCRVSRSSLKPCAQGASLAAYPAETPASGEEY
ASRPTEFAFYPGYPGPYQPMASYLDMSVVQTLGGPGEPRHDSLLPVDSYQPWAITGGWNS
QMCCQGEQNPPGHFWKAAFADSSTQHPPDGCAFRRGRKKRIPYSKGQLRELEREYSANKF
ITKDKRRKISAATSLSERQITIWFQNRRVKEKKVLAKVKTSATX
>gi73966255 ref_XP_548180.2 PREDICTED: homeobox protein Hox-B13 [Canis lupus
MEPGDYATLDGAKDLEGLSVVGGGRNLVAPSPLATHAAAPPLMPAVSYAPLDLPGPAEPP
KQCHPCPGVPQGAAPAPVPYGYFGGGYYSCRVARSSLKPCAQAATLAAYPAETATAGEEY
PSRPTEFAFYPGYPGPYQPMASYLDVSVVQTLGAPGEPRHDSLLPVDSYQPWALTGGWNG
QMCCQGEQNPPSAFWKAAFAESGTQHPPDGCAFRRGRKKRIPYSKGQLRELEREYSANKF
ITKDKRRKISAATSLSERQITIWFQNRRVKEKKVLAKVKTSTTX
>gi511890491 ref_XP_004764951.1 PREDICTED: homeobox protein Hox-B13 [Mustela
MEPGDYATLDGAKAMEGLPGAGGSRNLAARSPLPSHPAAAGAPPAVSYAPLDLPGSAEPP
KQCHPCPGVPQGTAPAAVPYGYFGGGYYSCRVSRSSLKPCAQAATLAAYPAETAAPGEEY
PSRPTEFAFYPGYPGPYQPMASYLDVSVVQTLGAPGEPRHDSLLPVDSYQPWSLTGGWNG
QMCCQGEQNPPGPFWKAAFAESGVQHPPDGCAFRRGRKKRIPYSKGQLRELELEYSANKF
ITKDKRRKISAATSLSERQITIWFQNRRVKEKKVLAKVKTTTNP
>gi254212171 gb_ACT65746.1 Hoxa13 [Leucoraja erinacea]
XXXXXXXXXEINKSMDGFSGGNFTANQCRN--LMAHPAS--LAPSSAYSSSELPASGEPV
KQCSPCPAA-QTSSSASLPYGYFGSSYYPCRMSHHSIKSCAQPSTLSGYPADKTSAGEEF
TTRAKEFAFYQGYAGPYQPMPSYLDVPVVPTISAPGEPRHETLLPMESYQPWAITNGWNG
QVCCSKEQPQPTHLWKSSLPDVVS-HPSDASSFRRGRKKRVPYTKVQLKELEREYATNKF
ITKDKRRRISASTNLSERQVTIWFQNRRVKEKKVLTKLKSNSXX
>gi558229251 ref_XP_006138460.1 PREDICTED: homeobox protein Hox-A13 [Pelodiscus
XXXXXXXXXEINKNMDGFHAGNQCRNLMAHPASLAAPSTAA----YTSSEAPASGMAEPA
KQCSPCSAAVQSSSGAALPYGYFGSGYYPCRMTHHNIKSCAQPSTFADKYMDTSVSGEEF
TSRAKELAFYPGYAGPYQPVPSYLDMPVVPTIGGPGEPRHEPLLPMESYQPWAITSGWNG
QVYCPKEQTQPPHLWKSTLPDVVS-HPSDASSYRRGRKKRVPYTKVQLKELEREYATNKF
ITKDKRRRISATTNLSERQVTIWFQNRRVKEKKVINKLKTTSXX
>gi752394348 ref_XP_011220149.1 PREDICTED: LOW QUALITY PROTEIN: homeobox protein
MEPGDYATLDGAKDLEGLPAAGGGRHLAAHSPLTSHPAAPALMPAVSYAPLDLPGSAEPP
KQCHPCPGVPQGPAPAPVPYGYFGGGYYSCRVSRSALKPCAQAX-LAAYPAEPATAGEEY
PSRPTEFAFYPGYPGPYQPMASYLDVSVVQTLGAPGEPRHDSLLQVDSYQPWALAGGWNG
QMCCQGEQNPPGPFWKAAFAESGAQHPPEGCAFRRGRKKRIPYSKGQLRELEREYSANKF
ITKDKRRKISAATSLSERQITIWFQNRRVKEKKVLAKVKTTTTX
>gi507952779 ref_XP_004684354.1 PREDICTED: homeobox protein Hox-B13 [Condylura
MEPGSFATLEGAKDVDGLLGAGGSRNLAAHSPLISHPAAPTLMPTVNYAPLDLPGPAEPP
KQCHPCPPVPQRVSPAPVPYGYFGGGYYSCRVSHSSLKPCAQAATLAAYPAEAPAAGEEY
PSRPTEFAFYPGYPGPYQPVASYLDVSVVQTLGAPGEPRHDSLLPVDSYQPWALSSGWNS
QMCCQGEQNPPGPFWKAAFADSSVQHPPDGCAIRRGRKKRVPYSKGQLRELEREYSANKF
ITKDKRRKISAATSLSERQITIWFQNRRVKEKKVLAKVKTSSTP
>gi507664676 ref_XP_004707141.1 PREDICTED: homeobox protein Hox-B13 [Echinops
MEPGNYATLDLAKDMEGLLGTGGARHLAPHYSLTSHPAAPTLMPAANYAPAELPGSAEPP
KQCHPCPGVPQGASPAPVPYGYFGGGYYSCRVSRSSLKPSAQAATLAAYPAETPAPGEEY
PSRATEFAFYPGYPGPYQPMASYLDVSVVPTLGASGEPRHDALLPVDSYQPWPLAGGWNS
QMCCQGEPNPPGPFWKAPFADSNSQHPPDGCAFRRGRKKRIPYSKGQLRELEREYSANKF
ITKDKRRKISAATSLSERQITIWFQNRRVKEKKVLSKVKTSATP

>gi224045310 ref_XP_002195397.1 PREDICTED: homeobox protein Hox-A13 [Taeniopygia
XXXXXXXXXEINKNMDGFAGSNFAANPCRNLMAHPAPLAAPSAAAYTSSEAPAAGMAEPA
KQCSPCSAAVQSSSGAALPYGYFGSGYYPCRMTHHNIKSCAQPSTFADKYMDTSVSGEEF
TSRAKEFAFYQGYAGPYQPVPGYLDMPVVPAIGGPGEPRHDPLLPMDSYQPWAITNGWNG
QVYCPKEQSQPPHLWKSTLPDVVS-HPSDANSYRRGRKKRVPYTKVQLKELEREYATNKF
ITKDKRRRISATTNLSERQVTIWFQNRRVKEKKVINKLKTTSXX
>gi400180333 gb_AFP73300.1 Hoxa13beta [Polyodon spathula]
XXXXXXXXXEVSKNMEGFAGGNFAANQCRN--LMAHPASLAPSTAYTSSEVPVSGMAEPV
KQCSPCSAAQSSSSA-SLPYGYFGSGYYPCRMAHHSIKSCAQPSAYAEKYMDTSGSGEEY
TSRAKEFAFYQGYASPYQPVPSYIDVPVVPTISGPGEPRHESLLPVESYQPWAITNGWNG
QVYCSKEQTQPTHLWKSTIQDVVS-HPGDVGSFRRGRKKRVPYTKVQLKELEREYATNKF
ITKDKRRRISAQTNLSERQVTIWFQNRRVKEKKVVHKTKSTSXX
>gi641724374 ref_XP_008152078.1 PREDICTED: homeobox protein Hox-B13 [Eptesicus
MEPGNYAAADGAKDLEGLLGAGGSRNLLAHSPLASHPAAPMLMPAVNYAPLDLPGSSEPP
KQCHPCPGAPQGASPAPVPYGYFGGGYYSCRLARGSLKPCAQAAALAAYPAEPPAAAEDY
PGRHAEFAFYPGYPAPYQPMASYLDVSVVQTLGAPGEPRHDALLPVDSYQPWALPGGWNS
QMCCPGDQNPPGSFWKTAFADSSAQHPPEGCTFHRGRKKRIPYSKGQLRELEREYSANKF
ITKDKRRKISVATSLSERQITIWFQNRRVKEKKVLAKVKTNTTX
>gi693302455 gb_AIS36044.1 HoxA13 [Ichthyophis bannanicus]
XXXXXXXXXEMNKNLEGFAGSNFAGNQCRN--LMAHPASLAPSASYPAGDAPAPGVAEPG
KQCSPCPG----SSGAALPYGYFGSGYYPCRVTHNAIKSCAQPSTFADKYMDTSVAGEEF
GSRAKEFAFYQGYAAPYQAVPGYLDMPVVPTLGGPGEPRHESLLPMESYQPWAIPNGWNG
QVYCSKEQTQPPHLWKSTLSDVVS-HPSDANSYRRGRKKRVPYTKIQLKELEREYASNKF
ITKDKRRRISATTNLSERQVTIWFQNRRVKEKKVINKLKSTSXX
>gi542179924 ref_XP_005468708.1 PREDICTED: homeobox protein Hox-B13a
XXXXXXXXXDLNKNMEGLVAASQCRNIMAHSALGGHPSSL-VHSSGGYSTVDVSASGSAA
PSGKQCAAVPHQSSSAALPYSYFGNGYYPCRMGRGPLKSCTQAAALSSQYMDTTVSSDEY
PNRAKEFAFYHGYASPYQSMASYLDVSVVQTLGT-GEPRHDTLLPMDSYQPWALTNAWGG
QMYCSKDQGQAGHLWKSALADVVA-HQNDGLSFRRGRKKRIPYTKVQLKELEKEYAANKF
ITKDKRRKISAVTNLSERQITIWFQNRRVKEKKFVAKVKSSTXX
>gi119370791 sp_Q1KKY3.1HXBDA_TAKRU RecName: Full=Homeobox protein Hox-B13a
XXXXXXXXXDLNKNMEGLVGSGQCRNIMAHSALGGHPSGL-VHSSTGYSTVDVTATSSSG
KQCAPCPAATQSSSAAALPYSYFGNGYYPCRMGRGSLKSCTQAAALSSQYMDTTVNSDEY
SNRAKEFAFYHSYPSPYQSMASYLDVSVVQTLGA-GEPRHDTLLPMDSYQPWALTNGWGG
QMYCSKDQGQAGHLWKSALADVVA-HQHDGSPFRRGRKKRIPYTKVQLKELEKEYAANKF
ITKDKRRKISAATNLSERQITIWFQNRRVKEKKFVAKVKSNAXX
>gi678126544 gb_AIM47931.1 HOXA13x [Pantodon buchholzi]
XXXXXXXXXEVNKNMDGFAGGNFAANQCRN--LMGHPASLAPSSAYSSADVPVSGMGEPV
KQCSPCSAAQSSSSA-SLPYSYFGSGYYPCRMSHHSIKSCAQSSAYGEKYMDTSVSGEDF
SSRAKEFAFYPGYPGPYQPVPGYLDVPVVSGMSGPAEPRHDPLLPMESYQPWAITNGWNG
QVYCAKEQSQPNHMWKASIQDAVS-HAGDGGAFRRGRKKRVPYTKVQLKELEREYATNKF
ITKDKRRRISAQTNLSERQVTIWFQNRRVKEKKVVNKLKTASXX
>gi808884419 gb_KKF31392.1 Homeobox protein Hox-B13a [Larimichthys crocea]
XXXXXXXXXDLNKNMEGLVGSGQCRNIMAHSALGGHPSGL-VHSSAGYSTVDVSASGSGE
KQCSPCPAAAQSSSAAALPYSYFGNGYYPCRMGRGSLKSCTQAAALSSQYMDTTVNSDEY
SNRAKEFAFYHGYPSPYQSMASYLDVSVVQTLGT-GEPRHDTLLPMDSYQPWALTNGWGG
QMYCSKDQGQAGHLWKSALADVVA-HQHDGSPFRRGRKKRIPYTKVQLKELEKEYAANKF
ITKDKRRKISAATNLSERQITIWFQNRRVKEKKFVAKVKTSAXX
>gi505802773 ref_XP_004608753.1 PREDICTED: homeobox protein Hox-B13 [Sorex
MEPGPYAALDGAKDVDGLLGAAAGRNLVAHSPLASPPAAPSLMPAVNYGPLDLPGSAEPP
KQCPPCPPGPPQASPAPVPYSYFGGGYYSCRVSRSSLKPCAPAPTLATYPTEAPVAGEEY
PSRPTEFAFYPGYPGPYQPMASYLDVSVVQTLGTPGEPRHDSLLPVDSYQPWALPGGWNG
QMCCQGEQTPPGPFWKAAFADPASQHPPD--SFRRGRKKRIPYSKGQLRELEREYSVNKF
ITKDKRRKISAATSLSERQITIWFQNRRVKEKKVLSKVKPSATP
>gi34484442 gb_AAQ72843.1 HoxB13a [Sphoeroides nephelus]
XXXXXXXXXDLNKNMEGLVGSGQCRNIMAHSALGGHPSGL-VHSSAGYSTVDVTATSSSG
KQCGPCPGATQSSSAAALPYSYFGNGYYPCRMGRGSLKSCTQAAALSSQYMDTTVNSDDY
SNRAKEFAFYHSYPSPYQSMASYLDVSVVQTLGA-GEPRHDTLLPMDSYQPWALTNGWGG
QMYCSKDQGQAGHLWKSALADVVA-HQHDGSPFRRGRKKRIPYTKIQLKELEKEYAANKF
ITKDKRRKISAATNLSERQITIWFQNRRVKEKKFGAKVKSSAXX
>gi519777295 gb_AGP03474.1 HoxA13 [Ambystoma mexicanum]
XXXXXXXXXEINKNMDGFPGANQCRNLMAHPAPLGPPGAAYSAPGGS------EGPAEAG
KQCSPCSA--QGSSGAALPYGYFGSGYYPCRVGHHGIKSCAQPSSFADKYMDTSSAGEEF
TSRAKEFAFYQGYAGPYQPVPGYLDMPMVPTVGGPGEPRHEPLLPMEPYQPWALTNGWNG
QVYCSKEQGQPPHLWKSSLPDVVS-HPSDANSYRRGRKKRVPYTKVQLKELEREYATNKF
ITKDKRRRISATTNLSERQVTIWFQNRRVKEKKVINKLKTTSXX
>gi657803392 ref_XP_008328783.1 PREDICTED: homeobox protein Hox-B13a
XXXXXXXXXDLNKNMEGLVGASQCRNIMAHSALGGHPSGL-MHSSAGYSTVDVSGSASAG
KQCSPCPAAHQSSSATTLPYGYFSNGYYPCRMGRGSLKSCTQAAALSSQYMDTTVNADEY

```
SNRAKEFAFYHGYPSPYQSMASYLDVSVVQTLGT-GEPRHDTLLPMDSYQPWALTNGWGG
QMYCSKDQGQAGHLWKSALADVVA-HQHDGSPFRRGRKKRIPYTKVQLKELEKEYAANKF
ITKDRRRKISAMTNLSERQITIWFQNRRVKEKKFVAKVKPSAXX
>gi554874539  ref_XP_005947176.1 PREDICTED: homeobox protein Hox-B13a
XXXXXXXXXELNKNMEGLVGSGQCRNIMAHSALGGHPSSL-VHSSGGYSTVDVSASGSAA
SSGKQCAAVPHQSSSAALPYSYFGNGYYPCRMGRAPLKSCTQAAALSSQYMDTTVSSDEY
PNRAKEFAFYHGYASPYQSMASYLDVSVVQTLGT-GEPRHDTLLPMDSYQPWALTNAWGG
QMYCSKDQGQAGHLWKSALADVVA-HQNDSLSFRRGRKKRIPYTKVQLKELEKEYAANKF
ITKDKRRKISAVTNLSERQITIWFQNRRVKEKKFVAKVKSSTXX
>gi499036069  ref_XP_004568702.1 PREDICTED: homeobox protein Hox-B13a [Maylandia
XXXXXXXXXELNKNMEGLVGSGQCRNIMAHSALGGHPSSL-VHSSGGYSTMDVSASASSG
KQCAAVPH-QNSSAATALPYSYFGNGYYPCRMGRAPLKSCTQAAALSSQYMDTTVSSDEY
PNRAKEFAFYHGYASPYQSMASYLDVSVVQTLGT-GEPRHDTLLPMDSYQPWALTNAWGG
QMYCSKDQGQAGHLWKSALADVVA-HQNDSLSFRRGRKKRIPYTKVQLKELEKEYAANKF
ITKDKRRKISAVTNLSERQITIWFQNRRVKEKKFVAKVKSSTXX
>gi301607689  ref_XP_002933431.1 PREDICTED: homeobox protein Hox-A13 [Xenopus
XXXXXXXXXEMNKNMDGFPAANQCRNLIGHHAP--------LAPSSAYPPSEVPVSAEPA
KQCNPCSAV-QSTPNASLPYGYFGSGYYPCRMSHHNIKSCSQPSSFAEKYMDTSSAGEDF
PSRPKEFAFYQSYPGPYQPVPSYLDMPVVSTIGTAGEPRHEPLLPMDGYQAWPITNGWNG
QVYCAKDQAQPTHLWKSSLPDVV--HQSDSSSYRRGRKKRVPYTKVQLKELEREYATNKF
ITKDKRRRISATTNLSERQVTIWFQNRRVKEKKVINKLKSTSXX
>gi584023332  ref_XP_006806308.1 PREDICTED: homeobox protein Hox-B13a-like
XXXXXXXXXELNKNMEGLIGSGQCRNIMAHSALGGHPSSL-VHSSGGYSTVDVSASGSAA
SSGKQCAAVPHQSSSAALPYSYFGNGYYPCRMGRAPLKSCTQAAALSSQYMDTTVSSDEY
PNRAKEFAFYHGYASPYQSMASYLDVSVVQTLGT-GEPRHDTLLPMDSYQPWALTNAWGG
QMYCSKDQGQAGHLWKSALADVVA-HQNDSLSFRRGRKKRIPYTKVQLKELEKEYAANKF
ITKDKRRKISAVTNLSERQITIWFQNRRVKEKKFVAKVKSSTXX
>gi548549273  ref_XP_005754834.1 PREDICTED: homeobox protein Hox-B13a [Pundamilia
XXXXXXXXXELNKNMEGLVGSGQCRNIMAHSALGGHPSSL-VHSSGGYSSVDVSASASSG
KQCAAVPH-QNSSAATALPYSYFGNGYYPCRMGRAPLKSCTQAAALSSQYMDTTVSSDEY
PNRAKEFAFYHGYASPYQSMASYLDVSVVQTLGT-GEPRHDTLLPMDSYQPWALTNAWGG
QMYCSKDQGQASHLWKSALADVVA-HQNDSLSFRRGRKKRIPYTKVQLKELEKEYAANKF
ITKDKRRKISAVTNLSERQITIWFQNRRVKEKKFVAKVKSSTXX
>gi657574876  ref_XP_008291162.1 PREDICTED: homeobox protein Hox-B13 [Stegastes
XXXXXXXXXDLNKNMEGLVGSGQCRNIMAHSALGGHPSGL-VHSSAGYSTVDVSASASSG
KQCAAGPCVPHQSSSAALPYSYFGNGYYPCRMGRGSLKSCTQAAALSSQYMDTTVSSDEY
PNRAKEFAFYHGYPSPYQSMASYLDVSVVQTLGT-GEPRHDTLLPMDSYQPWALTNGWGG
QMYCSKDQGQAGHLWKSALADVVA-HQHDGSPFRRGRKKRIPYTKVQLKELEKEYAANKF
ITKDKRRKISAVTNLSERQITIWFQNRRVKEKKFVAKVKSSVXX
>gi28268588  emb_CAC44975.2 homeobox protein, Hoxa13 [Xenopus laevis]
XXXXXXXXXEMNKNMDGFPAANQCRNLIGHHAP--------LPPSSAYPSSEVPVSAEPS
KQCNPCSAV-QSTPNGSLPYGYFGSGYYPCRMSHHNIKSCSQPSSFADKYMDTSSAGKDF
PSRPKEFAFYQSYPGPYQPVPSYLDMPVVSTIGSTGKPRHEPLLPMDGYQAWPITNGWNG
QVYCAKDQAQPTHLWKSSLPDVV--HQSDSSSYRRGRKKRVPYTKVQLKELEREYGTNKF
ITKDKRRRISATTNLSERQVTIWFQNRRVKEKKVINKLKSTSXX
>gi736251858  ref_XP_010788281.1 PREDICTED: homeobox protein Hox-B13a [Notothenia
XXXXXXXXXDLNKNMEGLVGASQCRNIMAHSALGGHPSGL-VHSSTGYPTVEVSASGSAG
KQCAPCPGAAQSSSAASLPYSYFGNGYYPCRMGRGSVKSCTQAAALSSQYMDTTVNPDDY
PNRAKEFAFYHGYPSPYQSMASYLDVSVVQTLGS--EPRHDTLLPMDSYQPWALTNGWGG
QMYC-KDQGQAGHLWKSALADAVA-HQHDGSHFRRGRKKRIPYTKLQLKELEKEYAANKF
ITKDKRRKISAGTNLSERQITIWFQNRRVKEKKFVAKVKTPTXX
>gi530624973  ref_XP_005302035.1 PREDICTED: homeobox protein Hox-B13 [Chrysemys
MDPGTFASAESTKSMEGLMGAGQCRNLVGHSALGG-PPPPALMQGSTYSTLDVPGAGEPS
KQCIPCPAIPQSSSTTPLPYGYFGSGYYSCRVARSALKPCPQSSYSADKYMDTPAAADDY
QTRPTELAFYPGYAGPYQPRASYLDVSVVQTIGGPGEPRHETLLPVDSYQPWALTGGWNS
QMCCPKDQSQAGHFWKSAFAD-VGQPPPDACSFRRGRKKRIPYTKGQLKELEKEYASSKF
ITKDKRRKIAAATNLTERQITIWFQNRRVKEKKVVAKVKTSSIP
>gi290760363  gb_ADD54543.1 HOXA13 [Pantherophis guttatus]
MEAGFHAAAAAAAAGTNFVAANQCRNLMVHPASLAGAGSAAAAAAAAYPSSEAPGMAETG
KQCSPCSAAVQGSSPAALPYSYFSGGYFPCSVNHHSLKSCAQPSSFADKYMETSVSSEDF
PSRAKEFAFYQGYAGPYQPMPGYLDMPVVPTIGGRGEPRHDSMLPVEGYQPWAIPNGWNG
QVYCPKEQNQPPHLWKSALPDVVS-HPSDANSYRRGRKKRVPYTKVQLKELEREYATNKF
ITKDKRRRISAATNLSERQVTIWFQNRRVKEKKVINKLKTSSXX
>gi551520979  ref_XP_005811980.1 PREDICTED: homeobox protein Hox-B13 [Xiphophorus
XXXXXXXXXDLNKNMEGLVGSGQCRNLMAHSAAAAAFGGHAVHSSAGYSTVDVSTSSSSG
KQCGPCPAAAQSSSAAALPYSYFGNSYYPCRMGRSSIKSCTQAAALSSQYMDTTVSPDEY
PNRAKEFTFYHGYANPYQSMGSYLDVSVVQTLGS-GDPRHETLLPMDSYQPWALTNGWGG
QMYCSKDQTQAGHLWKTALADVVA-HQHEGSPFRRGRKKRIPYTKVQLKELEKEYAANKF
ITKDKRRKISAVTNLSERQITIWFQNRRVKEKKFVAKVKSSAXX
```

```
>gi658861163  ref_XP_008413342.1 PREDICTED: homeobox protein Hox-B13 [Poecilia
XXXXXXXXXDLNKNMEGLVGSGQCRNLMAHSAAAAAFGGHAVHSSAGYSTVDVSTSSSSG
KQCGPCPAAAQSSSAAALPYSYFGNSYYPCRMGRSSIKSCTQAAALSSQYMDTTVNPDEY
PNRAKEFTFYHGYANPYQSMASYLDVSVVQTLGS-GNPRHETLLPMDSYQPWALTNGWGG
QMYCSKDQAQAGHLWKTALADVV-VHQHEGSPFRRGRKKRIPYTKVQLKELEKEYAANKF
ITKDKRRKISAVTNLSERQITIWFQNRRVKEKKFVAKVKSSAXX
>gi617509429  ref_XP_007542580.1 PREDICTED: homeobox protein Hox-B13a [Poecilia
XXXXXXXXXDLNKNMEGVVGSGQCRNLMAHSAAAAAFGGHAVHSSAGYSTVDVSTSSSSG
KQCGPCPAAAQSSSAAALPYSYFGNSYYPCRMGRSSIKSCTQAAALSSQYMDTTVNPDEY
PNRAKEFTFYHGYANPYQSMASYLDVSVVQTLGS-GDPRHETLLPMDSYQPWALTNGWGG
QMYCSKDQAQAGHLWKTALADVVA-HQHEGSPFRRGRKKRIPYTKVQLKELEKEYAANKF
ITKDKRRKISAVTNLSERQITIWFQNRRVKEKKFVAKVKSSAXX
>gi47229429  emb_CAF99417.1 unnamed protein product [Tetraodon nigroviridis]
XXXXXXXXXEVSKNMEGFAGGNFAANQCRN--LMGHPAS--LAPSTAYSSCDVPASAEPV
KQCSPCSAAQNSSSA-SLPYGYFGSGYYPCRMSHHSIKSCAAPSAYGEKYMDTSATSDDF
PSRTKEFTFYPTYPGPYQPVPSYLDVPVVPTISAPSEARHESLLPVDSYQPWTLPNGWNS
QVYCAKEQPQPGHMWKSSVADAAS-HTGESGSYRRGRKKRVPYTKVQLKELEREYAANKF
ITKDKRRRISAQTNLSERQVTIWFQNRRVKEKKVVNKLKTISXX
>gi831555258  ref_XP_012729648.1 PREDICTED: homeobox protein Hox-A13 [Fundulus
XXXXXXXXXDASKNMEGFPGGNFAANQCRNLMAHPASLAPSAAAAYSSGDASASAVGEPV
KQCSPCSAA-QNSTSASLPYGYFG---YPCRMSHHSIKSCAQPSAYGEKYMDTAASGEEF
SSRAKEFAFYPTYPGPYQPVPSYLDVPVVPAISAASEARHESLLPMESYQPWTLPNGWNG
QVYCAKEQPQPGHMWKSSLPDAVSHAAGDVGSFRRGRKKRVPYTKVQLKELEREYAANKF
ITKDKRRRISAQTNLSERQVTIWFQNRRVKEKKIVNKLKTISXX
>gi591374792  ref_XP_007062082.1 PREDICTED: homeobox protein Hox-B13 [Chelonia
MDPGTFASAEELDEMEGLMGAGGCRNLVAHSAVGG-PPPPAFVQGSNYPTLDAPGPGEPS
KQCIPCPAISQSSSTTPLPYGYFGSGYYSCRVARSSLKPCPQSSYSAEKYVDTPAAADDY
QTRPTELAFYPGYAGPYQPMASYLDVSVVQTIGGPGEPRHETLLPVDSYQPWALTSGWNS
QMCCPKDQNQAGHLWKSAFSD-AAQHPPDAGSFRRGRKKRVPYSKGQLKELEKEYASSKF
ITKDKRRKIAAATSLTERQITIWFQNRRVKEKKVVAKVKTSSTP
>gi432871359  ref_XP_004071926.1 PREDICTED: homeobox protein Hox-B13 [Oryzias
XXXXXXXXXDMSKNMDGLVGASQCRNIMAHSAAAAALGGHAMHSSAGFSTVDVSGSGETA
ATSAAAAAMPHQSSSAALPYGYFGNGYYPCRMGRGSLKSCTQAAALSSQYMDTTVSPDEY
PNHAKEFAFYHGYPSPYQSMASYLDVSVVQTLGA-GEPRHDSLLPMDGYQPWAL-NGWGG
QMYCSKEQAQAGHLWKSALADVVA-HQHDGSPFRRGRKKRIPYTKIQLKELEKEYAANKF
ITKDKRRKISAVTNLSERQITIWFQNRRVKEKKFVAKVKSSAXX
>gi602643660  ref_XP_007428288.1 PREDICTED: homeobox protein Hox-A13 [Python
XXXXXXXXXXXXXXXXXXXXXXXXXXXXXXXXXXXXXXXXXXXXXXXXYPSSEAPGMAEPG
KQCSPCSAAVQGASPAALPYSYFGSGYFPCSVNHHSLKSCAQPSSFADKYMETSVAGEDF
PSRAKEFAFYQGYAGPYQPVPGYLDMPVVPTLGGRGEPRHDSMLPVEGYQPWAIPNGWNG
QVYCPKEQNQPPHLWKSALPDVVS-HPSDANSYRRGRKKRVPYTKVQLKELEREYATNKF
ITKDKRRRISAATNLSERQVTIWFQNRRVKEKKVINKLKTSSXX
>gi961915500  ref_XP_014859829.1 PREDICTED: homeobox protein Hox-A13b-like
XXXXXXXXXERSKNMEGFTGGNFAANQCRN--LLAHPTSLAPSTTYAASDVPVSGVGEPG
KQCSPCSAT-QSSPNASLPYGYFGSGYYPCRVSHGGVKACAQPSGYGEKYADSSVSGE--
---AKEFAFYQGYSSPYQ--ASYLDVPVVPALSAPPEPR-ESLLPMEPYQPWAITNGWSG
QVYCTKEQSQANSLWKSSLQDSLSGGMD--SSVRRGRKKRVPYTKVQLKELEREYATNKF
ITKDKRRRISAQTNLTERQVTIWFQNRRVKEKKVVNKFKSSSXX
>gi961860111  ref_XP_014892304.1 PREDICTED: homeobox protein Hox-A13b-like
XXXXXXXXXERSKNMEGFTGGNFAANQCRN--LLAHPTSLAPSTTYAASDVPVSGVGEPG
KQCSPCSAT-QSSPNASLPYGYFGSGYYPCRVSHGGVKACAQPSGYGEKYADSSVSGE--
---AKEFAFYQGYSSPYQT--SYLDVPVVPALSAPPEPR-ESLLPMEPYQPWAITNGWSG
QVYCTKEQSQANSLWKSSLQDSLSGGMD--SSVRRGRKKRVPYTKVQLKELEREYATNKF
ITKDKRRRISAQTNLTERQVTIWFQNRRVKEKKVVNKFKSSSXX
>gi219879168  gb_ACL50963.1 homeobox protein, partial [Cyprinus carpio]
XXXXXXXXXDMSKNMEGFVGGNFAANQCRN--LIAHPSSLAPSTTYTSSEVPGPGMGEPV
KQCSPCSAV-QNSPSASLPYGYFGGSYYPCRMP----KSCTQPTSYGEKYMDTSVSGEEF
PSRAKEFAFYQGYSGPYQPVPSYLDVPVVPALSTPAEPRHESLLPVETYQPWAITNGWSS
PVYCPKDQTQSNSLWKSSIQDSVSG--TDGASVRRGRKKRVPYTKVQLKELEREYATNKF
ITKDKRRRISAHTNLTERQVTIWFXXXXXXXXXXXXXXXXXXXX
>gi219879156  gb_ACL50957.1 homeobox protein, partial [Carassius gibelio]
XXXXXXXXXDMSKNMEGFVGGNFAANQCRN--LIAHPSSLAPSTTYTSSEVPGPGMGEPV
KQCGPCSAV-QNSPSASLPYGYFGGSYYPCRMP----KSCTQPTSYGEKYMDTSVSGEEF
PSRAKEFAFYQGYSGPYQPVPSYLDVPVVPALSTPAEPRHESLLPVETYQPWAITNGWSS
PVYCPKDQTQSNSLWKSSIQDSVSG--TDGASVRRGRKKRVPYTKVQLKELEREYATNKF
ITKDKRRRISAHTNLTERQVTIWFXXXXXXXXXXXXXXXXXXXX
>gi219879164  gb_ACL50961.1 homeobox protein, partial [Carassius auratus auratus]
XXXXXXXXXDMSKNMEGFVGGNFAANQCRN--LIAHPSS--LAPSTTYTSSEVPGMGEPV
KQCSPCSAV-QNSPSASLPYGYFGGSYYPCRMP----KSCTQPSSYGEKYMDTSVSGEEF
```

```
PSRAKEFAFYQGYSGPYQPVASYLDVPVVPALSTPAEPRHESLLPVETYQPWAITNGWSS
PMYCPKDQTQSNSLWESSIQDTVSG--GDGASVRRGRKKRVPYTKVQLKELEREYATNKF
ITKDKRRRISAHTNLTERQVTIWFXXXXXXXXXXXXXXXXXXX
>gi123204435  gb_ABM73553.1 homeodomain protein, partial [Megalobrama
XXXXXXXXXDMSKNMEGFVGGNFAANQCRN--LIAHPSS--LAPSTTYSSSEVPGMGEPV
KQCSPCSAV-QNSPSASLPYGYFGGSYYPCRMP----KSCTQPTSYGEKYMDTSVSGEEF
SSRAKEFAFYQGYSGPYQPVPSYLDVPVVPALSAPAEPRHESLLPVETYQPWAITNGWSS
PVYCPKDQTQSNTLWKSSIQGT------DGASVRRGRKKRVPYTKVQLKELEREYATNKF
ITKDKRRRISAHTNLTERQVTIWFXXXXXXXXXXXXXXXXXXX
>gi465985114  ref_XP_004265734.1 PREDICTED: homeobox protein Hox-A13 [Orcinus
XXXXXXXXXXXXXXXXXXXXXXXXXXXXXXXXXXXXXXXXXXXXXXXXXXXXXXXXXXXX
KQCSPCSAAAQSSSPAALPYGYFGSGYYPCRMGPHAIKSCAQPASAADKYMDTAPAAEEF
SSRAKEFAFYQGYPGPYQPVPGYLDMPVVPGLGGPGESRHEPLLPMESYQPWALPNGWNG
QMYCPKEQAQPSHLWKSTLPDVVS-HPSDASSYRRGRKKRVPYTKVQLKELEREYATNKF
ITKDKRRRISATTNLSERQVTIWFQNRRVKEKKVINKLKTTSXX
>gi426355784  ref_XP_004045287.1 PREDICTED: homeobox protein Hox-A13 [Gorilla
XXXXXXXXXXXXXXXXXXXXXXXXXXXXXXXXXXXXXXXXXXXXXXXXXXXXXXXXXXXX
KQCSPCSAAAQSSSPAALPYGYFGSGYYPCRMGPHAIKSCAAAAAFADKYMDTAPAAEEF
SSRAKEFAFYQGYAGPYQPMPGYLDMPVVPGLGGPGESRHEPLLPMESYQPWALPNGWNG
QMYCPKEQAQPPHLWKSTLPDVVS-HPSDASSYRRGRKKRVPYTKVQLKELEREYATNKF
ITKDKRRRISATTNLSERQVTIWFQNRRVKEKKVINKLKTTSXX
>gi1034052196  ref_XP_016801065.1 PREDICTED: homeobox protein Hox-A13 [Pan
XXXXXXXXXXXXXXXXXXXXNQCRNLMAHPAPLA-PGAASAYSSAGGPGPAGPAGAEAA
KQCSPCSAAAQSSSPAALPYGYFGSGYYPCRMGPHAIKSCAAAAAFADKYMDTAPAAEEF
SSRAKEFAFYQGYAGPYQPMPGYLDMPVVPGLGGPGESRHEPLLPMESYQPWALPNGWNG
QMYCPKEQAQPPHLWKSTLPDVVS-HPSDASSYRRGRKKRVPYTKVQLKELEREYATNKF
ITKDKRRRISATTNLSERQVTIWFQNRRVKEKKVINKLKTTSXX
>gi683901043  ref_XP_009084324.1 PREDICTED: homeobox protein Hox-A13 [Serinus
XXXXXXXXXXXXXXXXXXXXXXXXXXXXXXXXXXXXXXXXXXXXXXXXXXXXXXXXXXXX
XXXXXXXXXXXXXXXXXXXXXXXXXXXXXXXXXHNAIKSCAQPSTFADKYMDTSVSGEEF
TSRAKEFAFYQGYAGPYQPVPGYLDMPVVPAIGGPGEPRHDPLLPMDSYQPWAITNGWNG
QVYCPKEQSQPPHLWKSTLPDVVS-HPSDANSYRRGRKKRVPYTKVQLKELEREYATNKF
ITKDKRRRISATTNLSERQVTIWFQNRRVKEKKVINKLKTTSXX
>gi729725749  ref_XP_010583800.1 PREDICTED: homeobox protein Hox-B13, partial
XXXXXXXXXXXXXXXXXXXXXXXXXXXXXXXXXXXXXXXXXXXXXXXXXXXXXXXXXXXX
XXXXXXXXXXXPAASAPLPYGYFGSGYYSCRVARSSLKTGPQGPFPTEKYLDPPAGSEDF
QSRPAEFAFYPGYGAPYQPVAGYLDVSVVPGLGGPGEARHETLLPVDGYQPWALGGGWSS
QMCCPKEQGQAGYLLKSAFADSAGQLPPDGCSFRRGRKKRVPYSKGQLKELEKEYASSKF
ITRDKRRKISAATNLTERQITIWFQNRRVKEKKVVAKIKSSSSX
>gi663243102  ref_XP_008498385.1 PREDICTED: homeobox protein Hox-B13, partial
XXXXXXXXXXXXXXXXXXXXXXXXXXXXXXXXXXXXXXXXXXXXXXXXXXXXXXXXXXXX
XXXXXXXXXXXXXXXXXPYGYFGSGYYSCRVSRSALKAGAQGPFPTEKYLDPSGATEDF
QSRPAEFAFYPGYGAPYQPVAGYLDVSVVPGLGGPGEARHETLLPVDGYQPWALGGGWSS
QMCCPKEQGQAGYLLKSAFADSAGQLPPDGCSFRRGRKKRVPYSKGQLKELEKEYASSKF
ITRDKRRKISAATNLTERQITIWFQNRRVKEKKVVAKIKTSSSX
>gi339792792  gb_AEK12777.1 homeodomain protein HOXA13, partial [Eurycea
XXXXXXXXXXXXXXXXXXGSNFAANQCRN--LMAHPAPLAPGAAYSSAPSGAEGSSEAG
KQCSPCSA-AQSSSGASLPYGYFGSGYYPCRVGHHAIKSCAQPSSFADKYMDTSSAGDEF
TSRAKEFAFYQGYAGPYQPVPGYLDMPMVPTVGGPGEPRHEPLLPMETYQPWAITNGWNG
QVYCSKEQAQPPHLWKSALPD-VASHPSDANAYRRGRKKRVPYTKVQLKELEREYATNKF
ITKDKRRRISATTNLSERQVXXXXXXXXXXXXXXXXXXXXXXXX
>gi768393729  ref_XP_011592537.1 PREDICTED: homeobox protein Hox-B13, partial
XXXXXXXXXXXXXXXXXXXXXXXXXXXXXXXXXXXXXXXXXXXXXXXXXXXXXXXXXXXX
XXXXXXXXXXXXXXXXXXYGYFGSGYYSCRVARSSLKAGPQGPFPTEKYLDPPAGSEDF
QSRPAEFAFYPGYGAPYQPVAGYLDVSVVPGLGGPGEARHETLLPVDGYQPWALGGGWSS
QMCCPKEQGQAGYLLKSAFADSAGQLPTDGCSFRRGRKKRVPYSKGQLKELEKEYASSKF
ITRDKRRKISAATNLTERQITIWFQNRRVKEKKVVAKIKSSSSX
>gi670977729  ref_XP_008686954.1 PREDICTED: homeobox protein Hox-B13, partial
XXXXXXXXXXXXXXXXXXXXXXXXXXXXXXXXXXXXXXXXXXXXXXXXXXXXXXXXXXXX
XXXXXXXXXXXXXXXXXXXXXXXXXXXXXCRVSRSALKPCAQAASLAAYPAEPATAWEEY
PSRPTEFAFYPGYPGPYQPMASYLDVSVVQTLGAPGEPRHDSLLQADSYQPWALAGGWTG
QMCCQGEQNPPGPFWKAAFAESGAQHPPEGCAFRRGRKKRIPYSKGQLRELEREYSANKF
ITKDKRRKISAATSLSERQITIWFQNRRVKEKKVLAKVKTTTTP
>gi327263844  ref_XP_003216727.1 PREDICTED: homeobox protein Hox-C13 [Anolis
XXXXXXXXXXXAAAMEGLAGASHCRDLIAHPALGRHSSGLGGHQGSVYAEIPAPDAA---
RQCPPPPPPPPPAASSLGYGYFGGSYYGCRLSHAQQKPCSYHPAAAEKYPPEPSPSEEL
SSRAKEFAFYPSFASSYQAVPGYLDVSVVPGISGHPEPRHDALLPMDGYQHWALSNGWDG
QVYCSKEQPQSGHLWKSPFPDVVPL-QPEVSSYRRGRKKRVPYTKIQLKELEKEYAASKF
ITKEKRRRISATTNLSERQVTIWFQNRRXXXXXXXXXXXXXXXX
```

```
>gi1040268871  gb_OBS77383.1 hypothetical protein A6R68_16224 [Neotoma lepida]
XXPGKAPSMDG---LGGSCPASHCRDLLPHPVLGRPPAPLGAPQGAVYTDIPAP---EAA
RQCAP-PPAPPTSSSATLGYGYFGGSYYGCRLSHNQQKPCAYHPGDKYPEPSGALPGDDL
SSRAKEFAFYPSFASSYQAMPGYLDVSVVPGISGHPEPRHDALIPVEGYQHWALSNGWDS
QVYCSKEQSQSAHLWKSPFPDVVPL-QPEVSSYRRGRKKRVPYTKVQLKELEKEYAASKF
ITKEKRRRISATTNLSERQVTIWFQNRRXXXXXXXXXXXXXXXX
>gi303303637  gb_ACF34401.2 homeobox C13 [Capra hircus]
XXPGKAPSMDG---LGNSCPASHCRDLLPHPVLGRPPAPLGAPQGAVYTDIPAP---EAA
RQCAP-PPAPPTSSSATLGYGYFGGSYYGCRLSHNQQKPCAYHSGDKYPEPSGALPGDDL
SSRAKEFAFYPSFASSYQAMPGYLDVSVVPGISGHPEPRHDALIPVEGYQHWALSNGWDS
QVYCSKEQSQSAHLWKSPFPDVVPL-QPEVNSYRRGRKKRVPYTKVQLKELEKEYAASKF
ITKEKRRRISATTNLSERQVTIWFQNRRXXXXXXXXXXXXXXXX
>gi724877051  ref_XP_010372185.1 PREDICTED: homeobox protein Hox-C13
XXPGKAPSMDG---LGSSCPASHCRDLLPHPVLGRPPAPLGAPQGAVYTDIPAP---EAA
RQCAP-PPAPPTSSSATLGYGYFGGSYYGCRLSHNQQKPCAYHPGDKYPEPSGALPGDDL
SSRAKEFAFYPSFASSYQAMPGYLDVSVVPGISGHPEPRHDALIPVEGYQHWALSNGWDS
QVYCSKEQSQSAHLWKSPFPDVVPL-QPEVSSYRRGRKKRVPYTKVQLKELEKEYAASKF
ITKEKRRRISATTNLSERQVTIWFQNRRXXXXXXXXXXXXXXXX
>gi544470246  ref_XP_005571103.1 PREDICTED: homeobox protein Hox-C13 [Macaca
XXPGKAPSMDG---LGSSCPASHCRDLLPHPVLGRPPAPLGAPQGAVYTDIPAP---EAA
RQCAP-PPAPPTSSSATLGYGYFGGSYYGCRLSHNQQKPCAYHPGDKYPEPSGALPGDDL
SSRAKEFAFYPSFASSYQAMPGYLDVSVVPGISGHPEPRHDALIPVEGYQHWALSNGWDS
QVYCSKEQSQSAHLWKSPFPDVVPL-QPEVSSYRRGRKKRVPYTKVQLKELEKEYAASKF
ITKEKRRRISATTNLSERQVTIWFQNRRXXXXXXXXXXXXXXXX
>gi338726307  ref_XP_001916847.2 PREDICTED: LOW QUALITY PROTEIN: homeobox protein
XXPGKAPSMDG---LGSSCPASHCRDLLPHPVLGRPPAPLGAPQGSVYTDIPTP---EAA
RQCAP-PPAPPTSSSATLGYGYFGGSYYGCRLSHNQQKPCAYHPGDKYPEPSGALPGDDL
SSRAKEFAFYPSFASSYQAMPGYLDVSVVPGISGHPEPRHDALIPVEGYQHWALSNGWDS
QVYCXKEQSQSAHLWKSPFPDVVPL-QPEVSSYRRGRKKRVPYTKVQLKELEKEYAASKF
ITKEKRRRISATTNLSERQVTIWFQNRRXXXXXXXXXXXXXXXX
>gi297692036  ref_XP_002823376.1 PREDICTED: homeobox protein Hox-C13 [Pongo
XXPGKAPSMDG---LGSSCPASHCRDLLPHPVLGRPPAPLGAPQGAVYTDIPAP---EAA
RQCAP-PPAPPTSSSATLGYGYFGGSYYGCRLSHNQQKPCAYHPGDKYPEPSGALPGDDL
SSRAKEFAFYPSFASSYQAMPGYLDVSVVPGISGHPEPRHDALIPVEGYQHWALSNGWDS
QVYCSKEQSQSAHLWKSPFPDVVPL-QPEVSSYRRGRKKRVPYTKVQLKELEKEYAASKF
ITKEKRRRISATTNLSERQVTIWFQNRRXXXXXXXXXXXXXXXX
>gi397521957  ref_XP_003831048.1 PREDICTED: homeobox protein Hox-C13 [Pan
XXPGKAPSMDG---LGSSCPASHCRDLLPHPVLGRPPAPLGAPQGAVYTDIPAP---EAA
RQCAP-PPAPPTSSSATLGYGYFGGSYYGCRLSHNQQKPCAYHPGDKYPEPSGALPGDDL
SSRAKEFAFYPSFASSYQAMPGYLDVSVVPGISGHPEPRHDALIPVEGYQHWALSNGWDS
QVYCSKEQSQSAHLWKSPFPDVVPL-QPEVSSYRRGRKKRVPYTKVQLKELEKEYAASKF
ITKEKRRRISATTNLSERQVTIWFQNRRXXXXXXXXXXXXXXXX
>gi1018974175  ref_XP_001510976.4 PREDICTED: homeobox protein Hox-A13, partial
XXXXXXXXXXXXXXXXXXXXXXXXXXXXXXXXXXXXXXXXXXXXXXXXXXXXXXXXXXXV
KQCSPCSAAAQSSSSGALPYGYFGSGYYPCRMGHHAIKSCAQPSAAADKYMDTPAAADDF
GSRAKEFAFYQGYAGPYQPVPGYLEMPVVPGLGGPAEPRHEPLLPMESYQPWALPNGWNG
QVYCPKEQPQPPHLWKSTLPDVVS-HPSDANSYRRGRKKRVPYTKVQLKELEREYATNKF
ITKDKRRRISATTNLSERQVTIWFQNRRVKEKKVINKLKTTSXX
>gi675430530  ref_XP_008929297.1 PREDICTED: homeobox protein Hox-D13, partial
XXXXXXXXXXXXXXXXXXXXXXXXXXXXXXXXXXXXXXXXXXXXXXXXXXXXXXXXXXXX
XXXXXXXXXXXXXXXPALGYGYFGNGYYSCRMSHNALKSPPHASFPVEKYMDTSVPANEV
SSRAKEVSFYQGYTTPYQHVPGYIDM--VSTFGS-GEPRHETYISMEGYQSWTLANGWNS
QVYCAKDQTQSSHFWKSSFPGDVALNQPDMCVYRRGRKKRVPYTKLQLKELENEYAINKF
INKDKRRRISAATNLSERQVTIWFQNRRVKDKKIVSKLKDNVSX
>gi795174618  ref_XP_011799454.1 PREDICTED: homeobox protein Hox-D13, partial
XXXXXXXXXXXXXXXXXXXXXXXXXXXXXXXXXXXXXXXXXXXXXXXXXXXXXXXXXXXX
XXXXXXXXXXXXXXXXXLGYGYFGNGYYSCRMSHNALKSSPHASFPVEKYMDSSVPANEV
PARAKEVSFYQGYTSPYQHVPGYIDM--VSTFGS-GEPRHEAYISMEGYQSWTLANGWNS
QVYCAKDQPQGSHFWKSSFPGDVALNQPDMCVYRRGRKKRVPYTKLQLKELENEYAINKF
INKDKRRRISAATNLSERQVTIWFQNRRVKDKKIVSKLKDTVSX
>gi699633156  ref_XP_009892936.1 PREDICTED: homeobox protein Hox-D13, partial
XXXXXXXXXXXXXXXXXXXXXXXXXXXXXXXXXXXXXXXXXXXXXXXXXXXXXXXXXXXX
XXXXXXXXXXXXXXXXXXXXGYFGNGYYSCRMSHNALKSPPHASFPVEKYMDTSVPANEV
SSRAKEVSFYQGYTTPYQHVPGYIDM--VSTFGS-GEPRHETYISMEGYQSWTLANGWNS
QVYCAKDQTQSSHFWKSSFPGDVALNQPDMCVYRRGRKKRVPYTKLQLKELENEYAINKF
INKDKRRRISAATNLSERQVTIWFQNRRVKDKKIVSKLKDNVSX
>gi374498883  gb_AEZ53160.1 Hox D13, partial [Streptopelia risoria]
XXXXXXXXXXXXXXXXXXXXXXXXXXXXXXXXXXXXXXXXXXXXXXXXXXXXXXXXXXXX
XXXXXXXXXXXXXXXXXXXYGYFGNGYYSCRMSHNALKSPPHASFPVEKYMDTSVPANEV
```

```
SSRAKEVSFYQGYTTPYQHVPGYIDM--VSTFGS-GEPRHETYISMEGYQSWTLANGWNS
QVYCAKDQTQSSHFWKSSFPGDVALNQPDMCVYRRGRKKRAPYTKLQLKELENEYAINKF
INKDKRRRISAATNLSERQVTIWFQNRRVKDKKIVSKLKDNVXX
>gi24637241  gb_AAN63624.1AF438315_1 transcription factor HoxC13 [Pleurodeles
XXXXXXXXXXXXXXMEGLCPSNHCRDFMAHPALGRGPQGP------VYTDIPAP---EAA
RQC----PAPTASSNAALGYGYFGGSYYGCRLSH------THNVNLQQKPCSYQEPAEDL
PSRAKEFAFYPSFSGSYQTVPGYLDMSVVPGISGHPEPRHDALIPMEGYQHWTLSNGWDG
QVYCPKEQGQAGHLWKSPFPDVVPL-QPEVSSYRRGRKKRVPYTKAQLKELEKEYAASKF
ITKEKRRRISAATGLSERQVTIWFQNRRXXXXXXXXXXXXXXXX
>gi584059997  ref_XP_006774469.1 PREDICTED: homeobox protein Hox-B13 [Myotis
XXXXXXXXXXXXXXXXXXXXXXXXXXXXXXXXXXXXXXXXXXXXXXXXXXXXXXXXXXXX
XXXXXXXXXXXXXXXXXXXXXXXXXXXXXXXXXXXXXXXXPATLAAYPAEPPAAGEDY
PSRHAEFAFYPGYPAPYQPMASYLDVSVVQTLGAPGEPRHDALLPVDSYQPWALPGGWNS
QMCCQGEQNPPGSFWKTAFADSSAQHPPEGCTFHRARKKRIPYSKGQLRELEREYSANKF
ITKDKRRKISVATSLSERQITIWFQNRRVKEKKVLAKVKTNATX
>gi884764069  ref_XP_012986341.1 PREDICTED: LOW QUALITY PROTEIN: homeobox protein
XXXXXXXXXXXXXXXXXXXXXXXXXXXXXXXXXXXXXXXXXXXXXXXXXXXXXXXXXXXX
XXXXXXXXXXXXAAAAPLPYGYFGSGYYSCRVARSALKAGAQAPFXGEKYLEPPAGAEDF
QSRPAEFAFYPGYGAPYQPVAGYLDVSVVPGLGGPGDARHETLLPVDGYQPWALGGGWSS
QMYCPKEQGQAGYLLKSAFADSAGQLPPDGCSFRRGRKKRVPYSKVQLRELEKEYASSKF
ITRDKRRKISAATNLTERQITIWFQNRRVKEXXXXXXXXXXXXX
>gi66773958  sp_Q5EU41.1HXD13_CARPS RecName: Full=Homeobox protein Hox-D13
XXXXXXXXXXXXXXXXXXXXXXXXXXXXXXXXXXXXXXXXXXXXXXXXXXXXXXXXXXXX
XXXXXXXXXXXXXXXXXLGYGYFGNGYYSCRMSHNALKSSPH-SSLGGFPVESSVPANEV
PARAKEVSFYQGYTSPYQHVPGYIDM--VSTFGS-GEPRHEAYISMEGYQSWTLANGWNS
QVYCAKDQPQGSHFWKSSFPGDVALNQPDMCVYRRGRKKRVPYTKLQLKELENEYAINKF
INKDKRRRISAATNLSERQVTIWFQNRRVKDKKIVSKLKDTVSX
>gi224016307  gb_ACN32429.1 homeobox D13, partial [Mesoplodon densirostris]
XXXXXXXXXXXXXXXXXXXXXXXXXXXXXXXXXXXXXXXXXXXXXXXXXXXXXXXXXXXX
XXXXXXXXXXXXXXXXXLGYGYFGNGYYSCRMSHNALKSSPHASFPVEKYMDSSVPANEV
PARAKEVSFYQGYTSPYQHVPGYIDM--VSTFGS-GEPRHEAYISMEGYQSWTLANGWNS
QVYCAKDQPQGSHFWKSSFPGDVALNQPDMCVYRRGRKKRVPYTKLQLKELENEYAINKF
INKDKRRRISAATNLSERQVTIWFQNRRVKDKKIVSKLKXXXXX
>gi224016295  gb_ACN32423.1 homeobox D13, partial [Megaptera novaeangliae]
XXXXXXXXXXXXXXXXXXXXXXXXXXXXXXXXXXXXXXXXXXXXXXXXXXXXXXXXXXXX
XXXXXXXXXXXXXXXXXLGYGYFGNGYYSCRMSHNALKSSPHASFPVEKYMDSSVPANEV
PARAKEVSFYQGYTSPYQHVPGYIDM--VSTFGS-GEPRHEAYISMEGYQSWTLANGWNS
QVYCAKDQPQGSHFWKSSFAGDVALNQPDMCVYRRGRKKRVPYTKLQLKELENEYAINKF
INKDKRRRISAATNLSERQVTIWFQNRRVKDKKIISKLKXXXXX
>gi224016303  gb_ACN32427.1 homeobox D13, partial [Kogia breviceps]
XXXXXXXXXXXXXXXXXXXXXXXXXXXXXXXXXXXXXXXXXXXXXXXXXXXXXXXXASSA
KECPAPGAAAAAPPGAPLGYGYFGNGYYSCRMSHNALKSSPHASFPVEKYMDSSVPANEV
PARAKEVSFYQGYTSPYQHVPGYIDM--VSTFGS-GEPRHEAYISMEGYQSWTLANGWNS
QVYCAKDQPQGSHFWKSSFPGDVALNQPDMCVYRRGRKKRVPYTKLQLKELENEYAINKF
INKDKRRRISAATNLSERQVTIWFQNRRVKDKKIVSKLKXXXXX
>gi224016309  gb_ACN32430.1 homeobox D13, partial [Stenella coeruleoalba]
XXXXXXXXXXXXXXXXXXXXXXXXXXXXXXXXXXXXXXXXXXXXXXXXXXXXXXXXASSA
KECPAPGAAAAAPPGAPLGYGYFGNGYYSCRMSHNALKSSPHASFPVEKYMDSSVPANEV
PARAKEVSFYQGYTSPYQHVPGYIDM--VSTFGS-GEPRHEAYISMEGYQSWTLANGWNS
QVYCAKDQPQGSHFWKSSFPGDVALNQPDMCVYRRGRKKRVPYTKLQLKELENEYAINKF
INKDKRRRISAATNLSERQVTIWFQNRRVKDKKIVSKLKXXXXX
>gi374498861  gb_AEZ53149.1 Hox D13, partial [Crocodylus niloticus]
XXXXXXXXXXXXXXXXXXXXXXXXXXXXXXXXXXXXXXXXXXXXXXXXXXXXXXXXXXXX
XXXXXXXXXXXXXXXXXXYGYFGNGYYSCRMAHGALKAAPHASFPVEKYMDTSVPANEV
SSRAKEVSFYQGYPSPYQHVPGYIDM--VSTFGS-GEPRHETYISMEGYQSWTLANGWNS
QVYCAKDQTQSSHFWKSSFPGDVALNQPDMCVYRRGRKKRVPYTKLQLKELENEYAINKF
INKDKRRRISAATNLSERQVTIWFQNRRVKDKKIVSXXXXXXXX
>gi224016313  gb_ACN32432.1 homeobox D13, partial [Phocoena phocoena]
XXXXXXXXXXXXXXXXXXXXXXXXXXXXXXXXXXXXXXXXXXXXXXXXXXXXXXXXASSA
KECSAPGAAAAAPPGAPLGYGYFGNGYYSCRMSHNALKSSPHASFPVEKYMDSSVPANEV
PARAKEVSFYQGYTSPYQHVPGYIDM--VSTFGS-GEPRHEAYISMEGYQSWTLANGWNS
QVYCAKDQPQGSHFWKSSFPGDVALNQPDMCVYRRGRKKRVPYTKLQLKELENEYAINKF
INKDKRRRISAATNLSERQVTIWFQNRRVKDKKIVSKLKXXXXX
>gi224016305  gb_ACN32428.1 homeobox D13, partial [Ziphius cavirostris]
XXXXXXXXXXXXXXXXXXXXXXXXXXXXXXXXXXXXXXXXXXXXXXXXXXXXXXXXXXXX
XXXXXXXXXXXXXXXXXLGYGYFGNGYYSCRMSHNALKSSPHASFPVEKYMDSSVPANEV
PARAKEVSFYQGYTSPYQHVPGYIDM--VSTFGS-GEPRHEAYISMEGYQSWTLANGWNS
QVYCAKDQPQGSHFWKSSFPGDVALNQPDMCVYRRGRKKRVPYTKLQLKELENEYAINKF
INKDKRRRISAATNLSERQVTIWFQNRRVKDKKIVSKLKXXXXX
```

```
>gi224016311  gb_ACN32431.1 homeobox D13, partial [Lagenorhynchus albirostris]
XXXXXXXXXXXXXXXXXXXXXXXXXXXXXXXXXXXXXXXXXXXXXXXXXXXXXXXXASSA
KECPAPGAAAAAPPGAPLGYGYFGNGYYSCRMSHNALKSSPHASFPVEKYMDSSVPANEV
PARAKEVSFYQGYTSPYQHVPGYIDM--VSTFGS-GEPRHEAYISMEGYQSWTLANGWNS
QVYCAKDQPQGSHFWKSSFPGDVALNQPDMCVYRRGRKKRVPYTKLQLKELENEYAINKF
INKDKRRRISAATNLSERQVTIWFQNRRVKDKKIVSKLKXXXXX
>gi224016299  gb_ACN32425.1 homeobox D13, partial [Balaenoptera acutorostrata]
XXXXXXXXXXXXXXXXXXXXXXXXXXXXXXXXXXXXXXXXXXXXXXXXXXXXXXXXXXXX
XXXXXXXXXXXXXXXXXLGYGYFGNGYYSCRMSHNALKSSPHASFPVEKYMDSSVPANEV
PARAKEVSFYQGYTSPYQHVPGYIDM--VSTFGS-GEPRHEAYISMEGYQSWTLANGWNS
QVYCAKDQPQGSHFWKSSFPGDVALNQPDMCVYRRGRKKRVPYTKLQLKELENEYAINKF
INKDKRRRISAATNLSERQVTIWFQNRRVKDKKIVSKLKXXXXX
>gi700384804  ref_XP_009932409.1 PREDICTED: homeobox protein Hox-D13, partial
XXXXXXXXXXXXXXXXXXXXXXXXXXXXXXXXXXXXXXXXXXXXXXXXXXXXXXXXXXXX
XXXXXXXXXXXXXXXXXXXXXXXXXGYYSCRMSHNALKSPPHASFPVEKYMDTSVPANEV
SSRAKEVSFYQGYTTPYQHVPGYIDM--VSTFGS-GEPRHETYISMEGYQSWTLANGWNS
QVYCAKDQTQSSHFWKSSFPGDVALNQPDMCVYRRGRKKRVPYTKLQLKELENEYAINKF
INKDKRRRISAATNLSERQVTIWFQNRRVKDKKIVSKLKDNVSX
>gi543378707  ref_XP_005532554.1 PREDICTED: homeobox protein Hox-B13
XXXXXXXXXXXXXXXXXXXXXXXXXXXXXXXXXXXXXXXXXXXXXXXXXXXXXXXXXXXX
XXXXXXXXXXXXXXXXXLPYGYFGSGFYSCRVSRSSLKPAPQGPFPNDKYLEAASGSEDF
QSRPAEFAFYPGYGAPYQPVPGYLDVAVVPGLG---EPRPEALLPVDGHQPWALGGGWGG
QMCCPKEQGQPGFLLKSAFAGKARREPQEGCALRRGRKKRVPYSKGQLKELEKEFASSKF
ITRDRRRKISAATNLSERQITIWFQNRRVKEKKVVAKIKNSAXX
>gi699659589  ref_XP_009898225.1 PREDICTED: homeobox protein Hox-B13, partial
XXXXXXXXXXXXXXXXXXXXXXXXXXXXXXXXXXXXXXXXXXXXXXXXXXXXXXXXXXXX
XXXXXXXXXXXXXXXXXXXXXXXXXXXXXCRVSRSSLKTGPQGPFPGEKYLDTPAGSEDF
QSRPTEFTFYPGYGAPYQPVTGYLDVSVVPGLGGPGDARHETLLPVDSYQPWALGSSWSS
QMCCSKEQGQAWYHLKSAFAASAGQLPPDGCSFRRGRKKRVPYSKGQLKELEKEYASSKF
ITRDKRRKISAATNLTERQITIWFQNRRVKEKKVVAKIKXXXXX
>gi310656724  gb_ADP02165.1 Hoxd13 [Typhlonectes natans]
XXXXXXXXXXXXXXMEGLRGEGSPGHQCRNLLASS-----------------APGCGYPG
QE-------RAEASKEALGYGYFGNRYYGVGIQQPPLKA-PPHASLGGFAMESTAPGNEG
ASRAKEVSFYQGYPNPYQHVPGYLDM--VSTFS-PGEPRHDTYISMEGYQSWTLANGWNS
QVYCAKDQAQTPPFWKSSFPGEVALNQPDLCVYRRGRKKRVPYTKPQLKELENEYAINKF
INKDKRRRISATTNLSERQVTIWFQNRRVKDKKLVSKLKETVXX
>gi565321354  gb_ETE72176.1 hypothetical protein L345_01994, partial [Ophiophagus
XXXXXXXXXXXXXXXXXXXXXXXXXXXXXXXXXXXXXXXXXXXXXXXXXXXXXXXXXXXX
XXXXXXXXXXXXXXXXXXXXXXXXXXXXXXXXXXXXXXXXXXXXXXXXXXMETSVSSEDF
PSRAKEFAFYQGYAGPYQPMAGYLDMPVVPTIGGRGEPRHDSMLPVEGYQPWAIPNGWNG
QVYCPKEQNQPPHLWKSALPDVVS-HPSDANSYRRGRKKRVPYTKVQLKELEREYATNKF
ITKDKRRRISAATNLSERQVTIWFQNRRVKEKKVINKLKTSSXX
>gi290760375  gb_ADD54552.1 HOXD13, partial [Gekko ulikovskii]
XXXXXXXXXXXXXXXXXXXXXXXXXXXXXXXXXXXXXXXXXXXXXXXXXXXXXXXXXXXX
XXXXXXXXXXXXXXXXXXXXXXXXXXYYSCRMAHGALKASPHAAFPGEKYMDVPSAGDPV
SSRAKEVSFYQGYTGPYQHVPGYLDVVSTFGSAAAGEPRHETYISMEGYQSWTLANGWNG
QVYCAKDQAQGSHFWKSSFPGDVALNQPDMCVYRRGRKKRVPYTKLQLKELESEYAVNKF
INKDKRRRISAATNLSERQVTIWFQNRRVKDKKIVSKLKDTVSX
>gi696966138  ref_XP_009553907.1 PREDICTED: homeobox protein Hox-D13 [Cuculus
XXXXXXXXXXXXXXXXXXXXXXXXXXXXXXXXXXXXXXXXXXXXXXXXXXXXXXXXXXXX
XXXXXXXXXXXXXXXXXXXXXXXXXXXXXXXXXXXXXXXXXXXXXXXXXXXXTSVPANEV
SSRAKEVSFYQGYTTPYQHVPGYIDM--VSTFGS-GEPRHETYISMEGYQSWTLANGWNS
QVYCAKDQTQSSHFWKSSFPGDVALNQPDMCVYRRGRKKRVPYTKLQLKELENEYAINKF
INKDKRRRISAATNLSERQVTIWFQNRRVKDKKIVSKLKDSVSX
>gi677975869  ref_XP_009072173.1 PREDICTED: homeobox protein Hox-B13, partial
XXXXXXXXXXXXXXXXXXXXXXXXXXXXXXXXXXXXXXXXXXXXXXXXXXXXXXXXXXXX
XXXXXXXXXXXXXXXXXXXXXXXXXXXXXXXXXXXXXXXXTQGSFPAEKYLEPPAGSEDF
QSRPAEFAFYPSYGAPYQPVAGYLDVSVVPGLGAPGEPRHETLLPVDGYQPWALGGGWSS
QMCCPKEQGQTGYLLKSAFADSPGQLPPDGCSFRRGRKKRVPYSKGQLKELEKEYASSKF
ITRDKRRKISAATNLTERQITIWFQNRRVKEKKVVAKIKSSTSX
>gi543746989  ref_XP_005514266.1 PREDICTED: homeobox protein Hox-D13 [Columba
XXXXXXXXXXXXXXXXXXXXXXXXXXXXXXXXXXXXXXXXXXXXXXXXXXXXXXXXXXXX
XXXXXXXXXXXXXXXXXXXXXXXXXXXXXXXXXXXXXXXXXXXXXXXXXXXXTSVPANEV
SSRAKEVSFYQGYTTPYQHVPGYIDM--VSTFGS-GEPRHETYISMEGYQSWTLANGWNS
QVYCAKDQTQSSHFWKSSFPGDVALNQPDMCVYRRGRKKRVPYTKLQLKELENEYAINKF
INKDKRRRISAATNLSERQVTIWFQNRRVKDKKIVSKLKDNVSX
>gi701389587  ref_XP_009994472.1 PREDICTED: homeobox protein Hox-D13, partial
XXXXXXXXXXXXXXXXXXXXXXXXXXXXXXXXXXXXXXXXXXXXXXXXXXXXXXXXXXXX
XXXXXXXXXXXXXXXXXXXXXXXXXXXXXXXXXXXXXXXXXXXXXXXEKYMDTSVPANEV
```

```
SSRAKEVSFYQGYTTPYQHVPGYIDM--VSTFGS-GEPRHETYISMEGYQSWTLANGWNS
QVYCAKDQTQSSHFWKSSFPGDVALNQPDMCVYRRGRKKRVPYTKLQLKELENEYAINKF
INKDKRRRISAATNLSERQVTIWFQNRRVKDKKIVSKLKDNVSX
>gi256014533  gb_ACU56821.1 HOXC13, partial [Pantherophis spiloides]
XENPNENNQTKIPAMEGLSGTSHCRDLISHPVLSRHSSTIGTHQGSVYTEIPAPDAA---
RQC----PAPPASSNATLGYSYFGGSYYGCRLSHSQQKPCAYHPAEKYPEPSGSLPNEEL
SSRAKEFAFYPSFASSYQAVPGYLDVSVVPGISGHPEPRHDALLPMEGYQHWALSNGWDG
QVYCAKEQSQSTHLWKSPFPDVVPL-QPEVNSYRRGRKKRVPYTKIQLKELEKEYAASKF
ITKEKXXXXXXXXXXXXXXXXXXXXXXXXXXXXXXXXXXXXXXX
>gi669281149  ref_XP_008633068.1 PREDICTED: homeobox protein Hox-D13 [Corvus
XXXXXXXXXXXXXXXXXXXXXXXXXXXXXXXXXXXXXXXXXXXXXXXXXXXXXXXXXXXX
XXXXXXXXXXXXXXXXXXXXXXXXXXXXXXXXXXXXXXXXXXXXXXXXXXXXXXXPANEV
SSRAKEVSFYQGYTTPYQHVPGYIDM--VSTFGS-GEPRHETYISMEGYQSWTLANGWNS
QVYCAKDQTQSSHFWKSSFPGDVALNQPDMCVYRRGRKKRVPYTKLQLKELENEYAINKF
INKDKRRRISAATNLSERQVTIWFQNRRVKDKKIVSKLKDNVSX
>gi118102888  ref_XP_001234927.1 PREDICTED: homeobox protein Hox-B13 [Gallus
XXXXXXXXXXXXXXXXXXXXXXXXXXXXXXXXXXXXXXXXXXXXXXXXXXXXXXXXXXXX
XXXXXXXXXXXXXXXXXXXXXXXXXXXXSCRVARSALKAGAQGPFPPEKYLDPPAGSEDF
QSRPAELAFYPGYGAPYQPVAGYLDVSVVPGLGGPGEPRHEALLPVDGYQPWALGGSWSG
QMCCPKEQGQAGYLLKSAFADAAAPLPPDGCAFRRGRKKRVPYSKGQLKELEKEYASSKF
ITRDKRRKISAATNLTERQITIWFQNRRVKEKKVVAKIKSSSXX
>gi554547429  ref_XP_005868266.1 PREDICTED: homeobox protein Hox-B13 [Myotis
MEPGNYAALDGAKDVEGLLGAGGGRRLLPHPPLFAYPGYPAPYPMASYLDVSVQTLGAPG
EPRHDALLPVDSYQPWALPGGW--------------------------------------
------FAFYPGYPAPYQPMASYLDVSVVQTLGAPGEPRHDALLPVDSYQPWALPGGWNS
QVCCQGEQNPSGSFWKTAFADSSAQHPPEGCTFHRGRKKRIPYSKGQLRELEREYSANKF
ITKDKRRKISVATSLSERQITIWFQNRRVKEKKVLAKVKTNATX
>gi694854743  ref_XP_009468862.1 PREDICTED: homeobox protein Hox-C13 [Nipponia
XXXXXXXXXXXXXXXXXXXXXXXXXXXXXXXXXXXXXXXXXXXXXXXXXXXXXXXXXXXX
XXXXXXXXXXXXXXXXXXXXXXXXXXXXXCRLSHSQQKPCAYHPGEKYPEAGGPLPGEEL
PTRAQEFAFYPGFPSSYQAVPGYLDVSVVPGLGGHPEPRHDALLPMEGYQHWALSNGWDG
QVYCSKEQSQSAHLWKSPFPDVVPL-QPEVSSYRRGRKKRVPYTKIQLKELEKEYAASKF
ITKEKRRRISATTNLSERQVTIWFQNRRXXXXXXXXXXXXXXXX
>gi529426764  ref_XP_005233213.1 PREDICTED: homeobox protein Hox-C13, partial
XXXXXXXXXXXXXXXXXXXXXXXXXXXXXXXXXXXXXXXXXXXXXXXXXXXXXXXXXXXX
XXXXXXXXXXXXXXXXXXXXXXXXGGYYGCRLSHSQQKPCAYHPGEKYPEAGGPLPGEEL
PSRAKEFAFYPGFPSSYQAVPGYLDVSVVPGLGGXXXXXHDALLPMEGYQHWALSNGWDG
QVYCSKEQSQSAHLWKSPFPDVVPL-QPEVSSYRRGRKKRVPYTKIQLKELEKEYAASKF
ITKEKRRRISATTNLSERQVTIWFQNRRXXXXXXXXXXXXXXXX
>gi706150961  ref_XP_010206808.1 PREDICTED: homeobox protein Hox-B13-like,
XXXXXXXXXXXXXXXXXXXXXXXXXXXXXXXXXXXXXXXXXXXXXXXXXXXXXXXXXXXX
XXXXXXXXXXXXXXXXXXXXXXXXXXXXXXXXXXXXXXXXXXXXXXXXXXXXXXXAGSEDF
QSRPAEFAFYPGYGAPYQPVAGYLDVSVVPGLGGPGEARHETLLPVDGYQPWALGGGWSS
QMCCPKEQGQAGYLLKSAFPDSAGPLPPEGCSFRRGRKKRVPYSKGQLKELEKEYASSKF
ITRDKRRKISAATNLTERQITIWFQNRRVKEKKVVAKIKGSSSX
>gi719780455  ref_XP_010222089.1 PREDICTED: LOW QUALITY PROTEIN: homeobox protein
XXXXXXXXXXXXXXXXXXXXXXXXXXXXXXXXXXXXXXXXXXXXXXXXXXXXXXXXXXXX
XXXXXXXXXXXXXXXXXXXXXXXXXXXXXXXXXXXXXXXXXXXXXXXXAKAPAGGEDS
QSPPAELAFYPSYGAPYQPVASYLDVSVVQTLGAPGEARHETLLPVDSYQPWALSGGWDX
QMCCPKEQSQAGYFLKSAFADAAAQLPPDGCSFRRGRKKRVPYSKGQLKELEKEYASNKF
ITRDKRRKISAATNLTERQITIWFQNRRVKEKKVVAKIKSSGTP
>gi290760370  gb_ADD54548.1 HOXD13, partial [Anguis fragilis]
XXXXXXXXXXXXXXXXXXXXXXXXXXXXXXXXXXXXXXXXXXXXXXXXXXXXXXXXXXXX
XXXXXXXXXXXXXXXXXXXXXXXXGGYYSCRMAHGALKASPHAAFPVEKYMDSVPSGSDV
SSR----AFYQGYAGPYQHVPGYLDVVSTFGSAAAGEPRHETYISMEGYQSWTLANGWNS
QVYCAKDQAQGSHFWKSSFPGDVAVNQPDLCVYRRGRKKRVPYTKLQLKELESEYALNKF
INKDKRRRISAATNLSERQVTIWFQNRRVKDKKIVSKLKDSVSX
>gi328682541  gb_AEB33352.1 homeobox protein D13, partial [Protopterus annectens]
XXXXXXXXXXXXXXMEGLKGDGLSSTHYRNSMHCSRSAPAFVCPVSEHTNHALYDSPKDC
PPCPPCPGSTAPSTP-SFSYGYFGNGYYSCRMSHNSLKSSAHASVGGEKYVDDSVTGNDV
SPRTKGLAFYQGYPGPYPRVPGYIDM--VPTLGSSVESRHDPFIPVDGYQPWPPTNSWNS
QIYCAKEQTQGSHFWKSPYTGDGALSQADVNSYRRGRKKRVPYTKLQLKELEREYAVNKF
ITKDKRRKIAAATSLSERQVTIXXXXXXXXXXXXXXXXXXXXXX
>gi541961056  ref_XP_005447460.1 PREDICTED: LOW QUALITY PROTEIN: homeobox protein
XXXXXXXXXXXXXXXXXXXXXXXXXXXXXXXXXXXXXXXXXXXXXXXXXXXXXXXXXXXX
XXXXXXXXXXXXXXXXXXXXXXXXXXXXXCRLSHSQQKPCAYHPGEKYPEAGGPLPGEEL
PSRAKEFAFYPGFPSSYQAVPGYLDVSVVPGLGGHPEPRHDPLLPMEGYQHWALSNGWDG
QVYCSKEQSQSAHLWKSPFPDVVPL-QPEVSSYRRGRKKRVPYTKIQLKELEKEYAASKF
ITKEKRRRISATTNLSERQVTIWFQNRRXXXXXXXXXXXXXXXX
```

>gi733928584 ref_XP_010725156.1 PREDICTED: homeobox protein Hox-C13-like,
XXXXXXXXXXXXXXXXXXXXXXXXXXXXXXXXXXXXXXXXXXXXXXXXXXXXXXXXXXXX
XXXXXXXXXXXXXXXXXXXXXXXXXXXXXXXXXXXXXXXXXXXXPSEKFTPNSTLPGEEL
PSRPKEFAFYPGFASSYQAVPGYLDVSVVPGLGAHPEPRHDALLPMEGYQHWALSNGWDG
QVYCSKEQSQSAHLWKSPFPDVVPL-QPEVSSYRRGRKKRVPYTKIQLKELEKEYAASKF
ITKEKRRRISATTNLSERQVTIWFQNRRXXXXXXXXXXXXXXXX
>gi686600796 ref_XP_009276657.1 PREDICTED: homeobox protein Hox-C13 [Aptenodytes
XXXXXXXXXXXXXXXXXXXXXXXXXXXXXXXXXXXXXXXXXXXXXXXXXXXXXXXXXXXX
XXXXXXXXXXXXXXXXXXXXXXXXXXXXXCRLSHSQQKPCAYHPGEKYPEAGGPLPGEEL
PSRAKEFAFYPGFPSSYQAVPGYLDVSVVPGLGGHPEPRHDALPPLEGYQHWALSNGWDG
QVYCSKEQSQSAHLWKSPFPDVVPL-QPEVSSYRRGRKKRVPYTKIQLKELEKEYAASKF
ITKEKRRRISATTNLSERQVTIWFQNRRXXXXXXXXXXXXXXXX
>gi704535956 ref_XP_010191822.1 PREDICTED: homeobox protein Hox-B13-like,
XXXXXXXXXXXXXXXXXXXXXXXXXXXXXXXXXXXXXXXXXXXXXXXXXXXXXXXXXXXX
XXXXXXXXXXXXXXXXXXXXXXXXXXXXXXXXXXXXXXXXXXXXXXXXXXXXXXXXSEDF
QSRPAEFAFYPSYGAPYQPVAGYLDVSVVPGLGGPGEARHETLLPVDGYQPWA--HGWSS
QMCCPKEQGQAGYLLKSAFADSTAQLPPDGCSFRRGRKKRVPYSKGQLKELEQEYASSKF
ITRDKRRKISAVTNLSERQIITIWFQNRRVKEKKVVAKIKSSTSX
>gi555983692 ref_XP_005903741.1 PREDICTED: homeobox protein Hox-A13 [Bos mutus]
XXXXXXXXXXXXXXXXXXXXXXXXXXXXXXXXXXXXXXXXXXXXXXXXXXXXXXXXXXXX
XXXXXXXXXXXXXXXXXXXXXXXXXXXXXXXXXXXXXXXXXXXXXXXXXXXTAGPAAEEF
SSRAKEFAFYQGYPGPYQPVPGYLDMPVVPGLGGPGESRHEPLLPMESYQPWALPNGWNG
QMYCPKEQAQPPHLWKSTLPDVVS-HPSDASSYRRGRKKRVPYTKVQLKELEREYATNKF
ITKDKRRRISATTNLSERQVTIWFQNRRVKEKKVINKLKTTSXX
>gi591300470 ref_XP_007077192.1 PREDICTED: homeobox protein Hox-A13 [Panthera
XXXXXXXXXXXXXXXXXXXXXXXXXXXXXXXXXXXXXXXXXXXXXXXXXXXXXXXXXXXX
XXXXXXXXXXXXXXXXXXXXXXXXXXXXXXXXXXXXXXXXXXXXXXXXXXMARGPAAEEF
SSRAKEFAFYQGYPGPYQPVPGYLDMPVVPGLGGPGESRHEPLLPMESYQPWALPNGWNG
QMYCPKEQAQPPHLWKSTLPDVVS-HPSDASSYRRGRKKRVPYTKVQLKELEREYATNKF
ITKDKRRRISATTNLSERQVTIWFQNRRVKEKKVINKLKTTSXX
>gi255683553 gb_ACU27366.1 HoxA13, partial [Lepidochelys olivacea]
XXXXXXXXXEINKNMEGFHAGNQCRNLMAHPASLAAPSTA----AYTSSEAPASGMAESA
KQCSPCSAAVQSSSGAALPYGYFGSGYYPCRMTHHNIKSCAQPSTFADKYMDTSVSGEEF
TSRAKDWAMLPGPTTRARVFGYACGAHNWRSWRA----RHEPLLPMESYQPWQLQ--WLN
GQCTA--QDQPAPL--ESLPDVVS-HPSDASSYRRGRKKRVPYTKVQLKELEREYATNKF
ITKDKRRRINXXXXXXXXXXXXXXXXXXXXXXXXXXXXXXXXXX
>gi255683559 gb_ACU27369.1 HoxA13, partial [Eretmochelys imbricata]
XXXXXXXXXEINKNMEGFHAGNQCRNLMAHPASLAAPSTA----AYTSSEARASGMAESA
KQCSPCSAAVQSSSGAALPYGYFGSGYYPCRMTHHNIKSCAQPSTFADKYMDTSVSGEEF
TSRAKDWAMLPGPTNRARVFGYACGAHNWRSWRA----RHEPLLPMESYQPWQ--SQWLN
GQCTA--QDQPAPL--ESLPDVVS-HPSDASSYRRGRKKRVPYTKVQLKELEREYATNKF
ITKDKRRRINXXXXXXXXXXXXXXXXXXXXXXXXXXXXXXXXXX
>gi558170872 ref_XP_006109670.1 PREDICTED: LOW QUALITY PROTEIN: homeobox protein
XXXXXXXXXXXXXXXXXXXXXXXXXXXXXXXXXXXXXXXXXXXXXXXXXXXXXXXXXXXX
XXXXXXXXXXXXXXXXXXXXXXXXXXXXXXXXXXXXXXXXXQAATLAAYPAEPPAAGEDY
PSRPRRSSFXPGGTAPYQAYGQVFDVSVVQTLGAPGEPRHDALLPVDSYQPWALPGGWNS
QMCCQGEQNPPGSFWKTAFADSSTQHPLEGCTFHRGRKKRIPYSKGQLRELEREYSANKF
ITKDKRRKISVATSLSERQITIWFQNRRVKEKKVLAKVKTNATX
>gi255683557 gb_ACU27368.1 HoxA13, partial [Caretta caretta]
XXXXXXXXXEINKNMEGFHAGNQCRNLMAHPASLAAPST----RAYTSSEAPASGMAESA
KQCSPCSAAVQSSSGAALPYGYFGSGYYPCRMTHHNIKSCAQPSTFADKYMDTSVSGEEF
TSRAKDWAMLPGPTTRARVFGYACGAHNWRSWRA----RHEPLLPMESYQPWQ--SQWLN
GQCTA--QDQPAPL--ESLPDVVS-HPSDASSYRRGRKKRVPYTKVQLKELEREYATNKF
ITKDKRRRINXXXXXXXXXXXXXXXXXXXXXXXXXXXXXXXXXX
>gi874491859 ref_XP_012961112.1 PREDICTED: LOW QUALITY PROTEIN: homeobox protein
XXXXXXXXXXXXXXXXXXXXXXXXXXXXXXXXXXXXXXXXXXXXXXXXXXXXXXXXXXXX
XXXXXXXXXXXXXXXXXXXXXXXXXXXXXXXXXXXXXXXXXGPFFPPEKYLDPPAGSEDF
QSRPAEFAFYPSYGAPYQPVAGYLDXXXXXGLGGPGEARHETLLPVDGYQPWALGGSWSG
QMCCPKEQGQAGYLLKSAFADATTQLPSDGCSFRRGRKKRVPYSKGQLKELEKEYASSKF
ITRDKRRKIAAATNLTERQITIWFQNRRVKEKKVVAKIKSSSTP
>gi726998761 ref_XP_010389649.1 PREDICTED: homeobox protein Hox-C13 [Corvus
XXXXXXXXXXXXXXXXXXXXXXXXXXXXXXXXXXXXXXXXXXXXXXXXXXXXXXXXXXXX
XXXXXXXXXXXXXXXXXXXXXXXXXXXXXXXXXXXXXXXXXXXXXXXXXXXXXXXGGEDL
PARAKEFAFYPGFPGSYQAVPGYLDVSVVPALGAHPEPRHEALLPMEGYQHWALSNGWDG
QVYCSKEQSQSAHLWKSPFPDVVPL-QPEVSSYRRGRKKRVPYTKIQLKELEKEYAASKF
ITKEKRRRISATTNLSERQVTIWFQNRRXXXXXXXXXXXXXXXX
>gi697489159 ref_XP_009675381.1 PREDICTED: homeobox protein Hox-C13, partial
XXXXXXXXXXXXXXXXXXXXXXXXXXXXXXXXXXXXXXXXXXXXXXXXXXXXXXXXXXXX
XXXXXXXXXXXXXXXXXXXXXXXXXXXXXXXXXXXXXXXXXXXXXXXXXXXXXPLPSEEL

PSRAKEFAFAYPGFASSYQTVPGYLDMSVVPALGGHPEPRHDALLPMEGYQHWALSNGWDG
QMYCSKEQSQSAHLWKSPFPDVVPL-QPEVSSYRRGRKKRVPYTKIQLKELEKEYAASKF
ITKEKRRRISATTNLSERQVTIWFQNRRXXXXXXXXXXXXXXXX
>gi525030647 ref_XP_005062707.1 PREDICTED: homeobox protein Hox-C13a-like,
XXXXXXXXXXXXXXXXXXXXXXXXXXXXXXXXXXXXXXXXXXXXXXXXXXXXXXXXXXXX
XXXXXXXXXXXXXXXXXXXXXXXXXXXXXXXXXXXXXXXXXXXXXXXXXXXXXXXXXXXX
XXXXXXXAFYPGFPGSYQAVPGYLDVSVVPALGAHPEPRHEPLLPMEGYQPWALANGWDG
QVYCPKEQPQPAHLWKSPFPDVVPL-QPEGSSFRRGRKKRVPYSKVQLKELEKEFAASKF
ITKEKRRRISASTSLSERQVTIWFQNRRVKEKKALSKAKXXXXX
>gi222159495 gb_ACM47252.1 Hox A13x, partial [Sigmops bathyphilus]
XXXXXXXXXEMNRNMEGFAGGNFAANQCRN--LLAHPTSLAPSTTYTSSEVPVSGMGEPV
KQCSPCSAT-QSSPNASLPYGYFGSSYYQCRMSHSSVKSCAQPSPYGDKYMDTSVSGEEF
TSRAKEFAFYQGYSGPYQPVSSYLDVPAVPALSCPTEPRHESLLPMEPYQPWAIANGWNG
QVYCAKEQPQSNALWKSSIQXXXXXXXXXXXXXXXXXXXXXXXXXXXXXXXXXXXXXXXX
XXXXXXXXXXXXXXXXXXXXXXXXXXXXXXXXXXXXXXXXXXXX
>gi187936703 dbj_BAG32149.1 Hox13 homolog, partial [Lethenteron camtschaticum]
XXXXXXXXXXXXXXXXXXXXXXXXXXXXXXXXXXXXXXXXXXXXXXXXXXXXXXXXXXXX
XXXXXXXXXXXXXXXXXXXXXXXXXXXXXXXXXXXXXXXXXXXXXXXXXXXXXXXXXDDY
TSAAKDFAFYPSYGSAYHHMPGYLDMPVVPSLAPPSEGRHDPILHMDGYQPWALPNGWNG
QMYCAKEQSQLSHLWKSPLAD-GSQPPHEMSLFRRGRKKRVPYTKVQLKELEREYAANKF
ITKDKRRKISAAISLSERQVTIWFQNRRVKEKKVVSKMKTTXXX
>gi690457118 ref_XP_009327741.1 PREDICTED: homeobox protein Hox-C13, partial
XXXXXXXXXXXXXXXXXXXXXXXXXXXXXXXXXXXXXXXXXXXXXXXXXXXXXXXXXXXX
XXXXXXXXXXXXXXXXXXXXXXXXGGYYGCRLSHSQQKPCAYHPGEKYPEAGGPLPGEEL
PSRAKEFAFYPGFPSSYQAVPGYLDVSV------------------------------DG
QVYCSKEQSQSAHLWKSPFPDVVPL-QPEVSSYRRGRKKRVPYTKIQLKELEKEYAASKF
ITKEKRRRISATTNLSERQVTIWFQNRRVKEKKVVSKTKTAXXX
>gi559163908 gb_AHB11499.1 homeobox protein A13, partial [Calotes versicolor]
MEAGFHAAAAAAAAGSNFVAANQCRNLMAHPASLAAPGSSAAAYTSSEAPPAAPGMAESG
KQCSPCSAAVQGSSPAALPYGYFGSGYFPCGMNHHALKSCAQPASFADKYMDTSGAGEDF
TSRAKEFAFYQGYAGPYQPVPGYLDMPVVPTLGGRGEPRHDSVLPMEGYQPWAIPNGWNG
QVYCPKEQNQPPXXXXXXXXXXXXXXXXXXXXXXXXXXXXXXXXXXXXXXXXXXXXXXXX
XXXXXXXXXXXXXXXXXXXXXXXXXXXXXXXXXXXXXXXXXXXX
>gi559163610 gb_AHB11350.1 homeobox protein A13, partial [Bipes biporus]
XXXXXXXXXXXXXXXXXXXXNQCRNLMAHPASLAAPGGTAAYTSSEAPAAGMVEPGAAV
KQCSPCSAAVQSSSPAALPYGYFGSGYFPCRMSHHNLKSCAQPSSFADKYMDTSVAGEDF
TSRAKEFAFYQGYAGPYQPVPGYLDMPVVPTIGGPGEPRHDSVLPVEGYQPWAITNGWNG
QVYCPKEQNQPPXXXXXXXXXXXXXXXXXXXXXXXXXXXXXXXXXXXXXXXXXXXXXXXX
XXXXXXXXXXXXXXXXXXXXXXXXXXXXXXXXXXXXXXXXXXXX
>gi559163543 gb_AHB11317.1 homeobox protein A13, partial [Amphisbaena caeca]
XXXXXXXXXXXXXXXXXXXXXQCRNLMAHPASLAAPGSTAAYTSSEAPAAGMAETGAAV
KQCSPCSAAVQSSSPAALPYGYFSSGYFPCRMSHHNLKSCAQPASLADKYMDTSVAGEDF
TSRAKEFAFYQGYAGPYQPVPGYLDMPVVPTIGGPGEPRHDPALPVEGYQPWAITNGWNG
QVYCPKEQNQPPXXXXXXXXXXXXXXXXXXXXXXXXXXXXXXXXXXXXXXXXXXXXXXXX
XXXXXXXXXXXXXXXXXXXXXXXXXXXXXXXXXXXXXXXXXXXX
>gi704260001 ref_XP_010150706.1 PREDICTED: homeobox protein Hox-C13, partial
XXXXXXXXXXXXXXXXXXXXXXXXXXXXXXXXXXXXXXXXXXXXXXXXXXXXXXXXXXXX
XXXXXXXXXXXXXXXXXXXXXXXXXXXXXXXXXXXQQKPCAYHPGEKYPEAGGPLPGEEL
PSRAKEFAFYPGFPSSYQAVPGYLDVSVVPGLGGHPEPR--------------------G
QVYCSKEQSQSAHLWKSPFPDVVPL-QPEVSSYRRGRKKRVPYTKIQLKELEKEYAASKF
ITKEKRRRISATTNLSERQVTIWFQNRRXXXXXXXXXXXXXXXX
>gi559163971 gb_AHB11530.1 homeobox protein A13, partial [Plestiodon fasciatus]
XXXXXXXXXXXXXXXXXXXXXNQCRNLMAHPASLAAPGSASAYTSSEAPA--AAGMAEPG
NKCSPCSAAVQSSSPAALPYGYFGSGYYPCRMNHHNLKSCAQPSSFADKYMDTSVAGEDF
TSRAKEFAFYPGYAGPYQPVPGYLDMPVVPTIGGPGEPRHDSMLPMEGYQPWAITNGWNG
QVYCPKEQNQPPXXXXXXXXXXXXXXXXXXXXXXXXXXXXXXXXXXXXXXXXXXXXXXXX
XXXXXXXXXXXXXXXXXXXXXXXXXXXXXXXXXXXXXXXXXXXX
>gi559163730 gb_AHB11410.1 homeobox protein A13, partial [Varanus salvator]
MEAGFHAAAAAAAAGTNFVSANQCRNLMAHPASLAGPGSAAAYTSSEAPAAAGMAEPAAV
KQCSPCSAAVQSPPPAALPYGYFGSGYFPCGMNHHSLKSCAQPASFADKYMDTSVAGEDF
TARAKEFAFYQGYAGPYQPVPGYLDMPVVPALGGRGEPRHDSVL--DGYQPWAITNGWNG
QVYCPKEQNQPPXXXXXXXXXXXXXXXXXXXXXXXXXXXXXXXXXXXXXXXXXXXXXXXX
XXXXXXXXXXXXXXXXXXXXXXXXXXXXXXXXXXXXXXXXXXXX
>gi269204564 gb_ACZ28806.1 mutant HoxA13, partial [Lacerta agilis]
XXXXXXXXXXXXXXXXXXXXXQCRNLMAHPASLAAPGSAAAYTSSEAPAAAAGMAEPSV
KQCSPCSAAVQSSSPAALPYGYFGSGYFPCRMSHHNLKSCAQPASFADKYMDTSVAGEDF
TSRAKEFAFYQGYAGPYQPVPGYLDMPVVPTIGGPGEPRHDSMLPMESYQPWAITNGWNG
QVYCPKEQNQPPHXXXXXXXXXXXXXXXXXXXXXXXXXXXXXXXXXXXXXXXXXXXXXXX
XXXXXXXXXXXXXXXXXXXXXXXXXXXXXXXXXXXXXXXXXXXX

```
>gi222159485  gb_ACM47247.1 Hox A13x, partial [Hiodon alosoides]
XXXXXXXXXEKNKNMDGFAGGNFAANQCRN--LXAHPASLAPSTAYSSGEVPVSGIGESA
KQCSPCSA-AQSSSSASLPYGYFGSGYYPCRMSHHSIKSCSQPSSYGEKYMDTSVSGEEF
TSRAKEFAFYQGYAGPYQPVPSYLDVPVVPGMSGPAETRHEPLLPMESYQPWAITNGWNS
QVYCXXXXXXXXXXXXXXXXXXXXXXXXXXXXXXXXXXXXXXXXXXXXXXXXXXXXXXXX
XXXXXXXXXXXXXXXXXXXXXXXXXXXXXXXXXXXXXXXXXXXX
>gi559164035  gb_AHB11562.1 homeobox protein A13, partial [Hemidactylus
XXXXXXXXXXXXXXXXXXXXXXQCRNLMAH-PASLAAPGSAAAAAYASSEAPAAGMAEPG
KQCSPCSAAVQSSSPAALPYGYFGSGYYPCRMSHHNLKSCAQPASFADKYMDTSVAGEDF
TSRAKEFAFYQGYAGPYQPVPGYLDMPVVPTIGGPAEPRHESMLPMEGYQPWAIPNGWNG
QVYCPKEQNQPPXXXXXXXXXXXXXXXXXXXXXXXXXXXXXXXXXXXXXXXXXXXXXXXX
XXXXXXXXXXXXXXXXXXXXXXXXXXXXXXXXXXXXXXXXXXXX
>gi222708647  gb_ACM67125.1 HoxA13, partial [Lepisosteus platostomus]
XXXXXXXXXEVNKNMEGFAGGNFAANQCRN--LLAHPASLAPSTAYTSSEVPVSGMAEPV
KQCSPCSAAQSSSSA-SLPYGYFGSGYYPCRMTHHSIKSCAQPSTYAEKYMDTSVSGEEF
TSRAKEFAFYQGYAGPYQPVPSYLDVPVVPTISAPGEPRHEPLLPVESYQPWAXXXXXXX
XXXXXXXXXXXXXXXXXXXXXXXXXXXXXXXXXXXXXXXXXXXXXXXXXXXXXXXXXXXX
XXXXXXXXXXXXXXXXXXXXXXXXXXXXXXXXXXXXXXXXXXXX
>gi290760385  gb_ADD54560.1 HOXD13, partial [Sphenodon punctatus]
XXXXXXXXXXXXXXXXXXXXXXXXXXXXXXXXXXXXXXXXXXXXXXXXXXXXXXXXXXXX
XXXXXXXXXXXXXXXXXXXXXXXXXXYYSCRMSHNPLKAAPHASLSGEKYMDTSGPANEV
SSRAKEVSFYQGYPNPYQHVPGYIDM--VSTFGS-GEPRHETYISMEGYQSWTLANGWNS
QVYCAKDQAQGSHFWKSPFPGDVALNQPDMCVYRRGRKKRVPYTKLQLKELENEYTINKF
INKDKRRXXXXXXXXXXXXXXXXXXXXXXXXXXXXXXXXXXXXX
>gi559163786  gb_AHB11438.1 homeobox protein A13, partial [Naja atra]
MEAGFHAAAAAAAASTNFVAANQCRNLMAHPASLAGPGSAAAAAYPSSEAPSAAGMAETG
KQCSPCSAAVQGSSPAALPYNYFSSGYFPCSVNHHSLKSCAQPSSFADKYMETSVSSEDF
PSRAKEFAFYQGYAGPYQPMPSYLDMPVVPTIGGRGEPRHDSMLPVEGYQPWSIPNGWNG
QVYCPKEQNQPPXXXXXXXXXXXXXXXXXXXXXXXXXXXXXXXXXXXXXXXXXXXXXXXX
XXXXXXXXXXXXXXXXXXXXXXXXXXXXXXXXXXXXXXXXXXXX
>gi559163846  gb_AHB11468.1 homeobox protein A13, partial [Indotyphlops braminus]
MEAGFHAAAAAAAAGTNFVAANQCRNLMAHPASLTGPGSAAAAYPSSEAPSAAGMAEAGV
KQCSPCSAAVQNPSPAALPYGYFGSGYFPCSVNNASLKSCSQPSSFADKYMDTSAASEDF
PSRAKEFAFYQGYAGPYQPMPGYLDVPVVPTLGGRGEPRHDSMLPMEGYQPWAITSGWNG
QVYCPKEQNQPPXXXXXXXXXXXXXXXXXXXXXXXXXXXXXXXXXXXXXXXXXXXXXXXX
XXXXXXXXXXXXXXXXXXXXXXXXXXXXXXXXXXXXXXXXXXXX
>gi318088612  gb_ADV40868.1 homeodomain protein HoxC13a, partial [Opsariichthys
XXXXNENNQNKSQIMEGLSGATHCRELISHPALGRHSGTIATHQGSVYSDISSP---ETG
RQC----PAPQTSSSASLSYGYFGNPYYGCRLSHSQQKPCSYHPAEKYAETSSALPAEEL
SSRAKEFAFYPSFASSYQAVPGYLDMSVVPSISAHPEPRHDALIPMEGYQHWALSNGWDG
QVYCSKEQTQSSHXXXXXXXXXXXXXXXXXXXXXXXXXXXXXXXXXXXXXXXXXXXXXXX
XXXXXXXXXXXXXXXXXXXXXXXXXXXXXXXXXXXXXXXXXXXX
>gi318088602  gb_ADV40863.1 homeodomain protein HoxC13a, partial
XXXXNENNQNKSQIMEGLSGATHCRELISHPALGRHSGTIATHQGSVYSDISSP---ETG
RQC----PAPQTSSSASLSYGYFGNPYYGCRLSHSQQKPCSYHPAEKYAETSSALPAEEL
SSRAKEFAFYPSFASSYQTVPGYLDMSVVPSISAHPEPRHDALIPMEGYQHWALSNGWDG
QVYCSKEQTQSSHXXXXXXXXXXXXXXXXXXXXXXXXXXXXXXXXXXXXXXXXXXXXXXX
XXXXXXXXXXXXXXXXXXXXXXXXXXXXXXXXXXXXXXXXXXXX
>gi222708673  gb_ACM67138.1 HoxA13, partial [Apteronotus leptorhynchus]
XXXXXXXXXDMNKNMEGFVGANFSANQCRN--LMAHPPSLAPSSTYASTEVPVSGMGEPV
KQCSPCSAA-QSSPGATLPYGYFGSSYYPCRMSHHSVKSCAQPSPYGEKYMDTSVSGEEF
SSRAKEFAFYQGYSGPYQPVPSYLDVPVVPALSAPAEPRHESVLPVETYQPWAXXXXXXX
XXXXXXXXXXXXXXXXXXXXXXXXXXXXXXXXXXXXXXXXXXXXXXXXXXXXXXXXXXXX
XXXXXXXXXXXXXXXXXXXXXXXXXXXXXXXXXXXXXXXXXXXX
>gi222708649  gb_ACM67126.1 HoxA13, partial [Amia calva]
XXXXXXXXXEVNKSMEGFAGGNFAANQCRN--LMAHPASLAPSTAYTSSEVPVSGMAEPV
KQCSPCSAAQSSSSA-SLPYGYFGSGYYPCRMTHHSIKSCAQPASYAEKYMDTSGSGEDF
TSRAKEFAFYQGYAGPYQPVPSYLDVPVVPAISGPGEPRHEPLLPMESYQPWAXXXXXXX
XXXXXXXXXXXXXXXXXXXXXXXXXXXXXXXXXXXXXXXXXXXXXXXXXXXXXXXXXXXX
XXXXXXXXXXXXXXXXXXXXXXXXXXXXXXXXXXXXXXXXXXXX
>gi222708671  gb_ACM67137.1 HoxA13, partial [Chanos chanos]
XXXXXXXXXNINKSMEGFVGSNFTANQCRN--LVSHPTSLAPSTTYASSEVPVSGMSEAV
KQCSPCSAV-QSSPGASLPYGYFGSGYYQCRMSHHNSKSCAQPSSYGEKYMDTSISGEEF
PSRAKEYTFYQGYPGPYQPVPSYLDMPVVPALSAPAEPRHDSLLPVETYQPWTXXXXXXX
XXXXXXXXXXXXXXXXXXXXXXXXXXXXXXXXXXXXXXXXXXXXXXXXXXXXXXXXXXXX
XXXXXXXXXXXXXXXXXXXXXXXXXXXXXXXXXXXXXXXXXXXX
>gi222708655  gb_ACM67129.1 HoxA13, partial [Anguilla rostrata]
XXXXXXXXXEVNKNMEGFAGGNFAANQCRN--LMAHPASLAPSATYTSSEVPASGMGESV
KQCSPCSAAQSSTSA-SLPYGYFGSSYYPCRMSHSNIKSCAQPSSYAEKYMETSVSGEEF
```

```
TSRAKEFAFYQGYTSPYQPVPSYLDMPVVPTMSGPTEPRHDSLLPMESYQPWAXXXXXXX
XXXXXXXXXXXXXXXXXXXXXXXXXXXXXXXXXXXXXXXXXXXXXXXXXXXXXXXXXXXX
XXXXXXXXXXXXXXXXXXXXXXXXXXXXXXXXXXXXXXXXXXXX
>gi222708681  gb_ACM67142.1 HoxA13, partial [Gyrinocheilus sp. KDC-2009]
XXXXXXXXXDMNKNMEGFVGGNFAANQCRN--LIAHPSS--LAPSTTYTSSEVPGMGEPV
KQCSPCSAV-QNSPSASLPYGYFGGSYYQCRMP----KSCTQPTSYGEKYMDTSVSGEEF
PSRAKEFAFYQGYSGPYQPVPSYLDVNVVPALSAPAEPRHESLLPVETYQPWAXXXXXXX
XXXXXXXXXXXXXXXXXXXXXXXXXXXXXXXXXXXXXXXXXXXXXXXXXXXXXXXXXXXX
XXXXXXXXXXXXXXXXXXXXXXXXXXXXXXXXXXXXXXXXXXXX
>gi318088600  gb_ADV40862.1 homeodomain protein HoxC13a, partial
XXXXNENNQNKTKIMEGLSGATPCRDRISHPALGRHSGTIATHQGSVDLDIFSP---ETG
RQC----PAPQTSSSASLSYGYFGNPYYGCRLSHSQEKPCSYHPAEKYAETSSALPAEEL
SSRAKEFAFYPSFASSYQTVPGYLDMSVVPSISAHPEPRHDALIPMEGYQHWALSNGWDG
QVYCSKEQTQSSHXXXXXXXXXXXXXXXXXXXXXXXXXXXXXXXXXXXXXXXXXXXXXXX
XXXXXXXXXXXXXXXXXXXXXXXXXXXXXXXXXXXXXXXXXXXX
>gi318088598  gb_ADV40861.1 homeodomain protein HoxC13a, partial
XXXXNENNQNKSQIMEGLSGATHCRELISHPALGRHSGTIATHQGSVYSDISSP---ETG
RQC----PAPQTSSSASLSYGYFGNPYYGSRLSHSQQKPCSYHPAEKYAETSSALPAEEL
SSRAKEFAFYPSFASSYQAVPGYLDMSVVPSISAHPEPRHDALIPMEGYQHWALSNGWDG
QVYCSKEQTQSSHXXXXXXXXXXXXXXXXXXXXXXXXXXXXXXXXXXXXXXXXXXXXXXX
XXXXXXXXXXXXXXXXXXXXXXXXXXXXXXXXXXXXXXXXXXXX
>gi222708679  gb_ACM67141.1 HoxA13, partial [Misgurnus anguillicaudatus]
XXXXXXXXXDMNKNMEGFVGGNFAANQCRN--LIAHPSS--LAPSTTYTSSEVPGMGEPV
KQCSPCSA--QNSPGASLPYGYFGGSYYPCRMP----KSCTQPTSYGEKYMDTSVSGEEF
PSRAKEFAFYQGYSGPYQPVPSYLDVPVVPALSAPAEPRHESLLPVETYQPWAXXXXXXX
XXXXXXXXXXXXXXXXXXXXXXXXXXXXXXXXXXXXXXXXXXXXXXXXXXXXXXXXXXXX
XXXXXXXXXXXXXXXXXXXXXXXXXXXXXXXXXXXXXXXXXXXX
>gi318088608  gb_ADV40866.1 homeodomain protein HoxC13a, partial [Xenocypris
XXXXNENIQNKSQIMEGLRGATHCRELISHPALGRHSGTIATHQGSAYSDISSP---ETG
RQC----PAPQTSSRASLSYGYFGNPYYGCRLSHSNLQQKHGSYNPAEKYAETSSPAEEL
SSRAKEFAFYPSFASSYQAVPGYLDMSVVPSISAHPEPRHDALIPMEGYQHWALSNGWDG
QVYCSKEQTQSSHXXXXXXXXXXXXXXXXXXXXXXXXXXXXXXXXXXXXXXXXXXXXXXX
XXXXXXXXXXXXXXXXXXXXXXXXXXXXXXXXXXXXXXXXXXXX
>gi559164131  gb_AHB11610.1 homeobox protein C13, partial [Dibamus bourreti]
XXXXXXXXXXXXXAMEGLSGASHCRDLITHPVLGRHSSTIGTHQGSVYTDIPAPDAA---
RQC----PAPPASSNATLGYGYFGGSYYGCRLSHSQQEPCSYHPAEKYPEPSGSLPSEEL
STRAKEFAFYPSFASSYQAVPGYLDVSVVPGISGHPEPRHDALLPMEGYQHWALSNGWDG
QVYCSKEQSQSXXXXXXXXXXXXXXXXXXXXXXXXXXXXXXXXXXXXXXXXXXXXXXXXX
XXXXXXXXXXXXXXXXXXXXXXXXXXXXXXXXXXXXXXXXXXXX
>gi318088606  gb_ADV40865.1 homeodomain protein HoxC13a, partial [Elopichthys
XXXXNENNQNKTQIMEGLSGATLCRELISHPALGRHSGTIATHQGSVYSDIFSP---ETG
RQC----PAPQTSSSASLSYGYFGNPYYGCRLSHSQQKPCSSHPAEKYAETSSALPAEEL
SSRAKQFAFYPSFARSYQAVPGYLDMSVLPSISAHPEPRYNALIPMEGYQHWALSNGWDG
HVYCSKEQKQHSHXXXXXXXXXXXXXXXXXXXXXXXXXXXXXXXXXXXXXXXXXXXXXXX
XXXXXXXXXXXXXXXXXXXXXXXXXXXXXXXXXXXXXXXXXXXX
>gi318088610  gb_ADV40867.1 homeodomain protein HoxC13a, partial [Culter
XXXXXXXNLNKSPIMEGLSGATHCRKLISHPALGRHSGTIATHQGSVYSDISPP---ETG
RLC----PAPQTSSSASLTYGYFGNPYYGCRLSHSQQKPCSYHPAEKYAETSSALPAEEL
SGRAKEFAFYPSFASSYQAVPGYLDMSLVPSISAHPEPRHDALIPMEGYHHWALSNGWDG
QVYCSKEQTQSSHXXXXXXXXXXXXXXXXXXXXXXXXXXXXXXXXXXXXXXXXXXXXXXX
XXXXXXXXXXXXXXXXXXXXXXXXXXXXXXXXXXXXXXXXXXXX
>gi222708677  gb_ACM67140.1 HoxA13, partial [Carassius auratus]
XXXXXXXXXDMSKNMEGFVGGNFAANQCRN--LIAHPSS--LAPSTTYTSSEVPGMGEPG
RQCSPCSAV-HTSPSASLPYGYFGGSYYQCRMP----KTCTQPSSYGEKYMDTSVSGEDF
PSRAKEFAFYQGYSGSYQPVPSYLDVPVVPA-----EPRHESLLPVETYQPWAXXXXXXX
XXXXXXXXXXXXXXXXXXXXXXXXXXXXXXXXXXXXXXXXXXXXXXXXXXXXXXXXXXXX
XXXXXXXXXXXXXXXXXXXXXXXXXXXXXXXXXXXXXXXXXXXX
>gi222708657  gb_ACM67130.1 HoxA13, partial [Dorosoma cepedianum]
XXXXXXAADEINKNMEGFAGASQCRNLMAH--------APSLAPSSGYSDVPPSGMSEPG
KQCSPCSA-AQSSSNASLPYGYFGSGYYPCRMSHHTVKPCAQPS-YADKYMDTSVPGEDF
PSRPKEFAFYQGYPGPYQPVPSYLDMPVVPALSGSAEPRHEPLLPVDSYQPWAXXXXXXX
XXXXXXXXXXXXXXXXXXXXXXXXXXXXXXXXXXXXXXXXXXXXXXXXXXXXXXXXXXXX
XXXXXXXXXXXXXXXXXXXXXXXXXXXXXXXXXXXXXXXXXXXX
>gi700431350  ref_XP_009954457.1 PREDICTED: homeobox protein Hox-A13, partial
XXXXXXXXXXXXXXXXXXXXXXXXXXXXXXXXXXXXXXXXXXXXXXXXXXXXXXXXXXXX
XXXXXXXXXXXXXXXXXXXXXXXXXXXXXXXXXXXXXXXXXXXXXXXXXXXXXXXXXXXX
XXXXXXXXXXXXXXXXXXXXXXXXXXXXXXXXXXXXXXXXXXXXXXXXXXXXXXXXXXXX
XMCCPKEQGQAGYLLKSAFADSSGQLPPDGCSFRRGRKKRVPYSKGQLKELEKEYASSKF
ITRDKRRKISAATNLTERQITIWFQNRRVKEKKVVAKIKSSSSX
```

```
>gi387231673  gb_AFJ72547.1 homeobox B13, partial [Rousettus leschenaultii]
XXXXXXXXXXXXXXXXXXXXXXXXXXXXXXXXXXXXXXXXXXXXXXXXXXXXXXXXXXXX
XXXXXXXXXXXXXXXXXVPFGYFGGGYYSCRVSRSSLKSCAQAATLTAYPAETPSAGEEY
PSRPTEFAFYPGYPGSYQPMASYLDVSVVQTLGAXGEPRHEALLPMDSYQPWALTGGWNS
QMCCQGEXXXXXXXXXXXXXXXXXXXXXXXXXXXXXXXXXXXXXXXXXXXXXXXXXXXXX
XXXXXXXXXXXXXXXXXXXXXXXXXXXXXXXXXXXXXXXXXXXX
>gi661918810  gb_AIE39454.1 Hoxd13, partial [Trachemys scripta]
XXXXXXXXXXXXXXXXXXXXXXXQCRNFLSSPVFGAHSGRSAAAPGFAYAPERSGSAARSD
PPSKECPGSAAPSATPALGYGYFGNGYYSCRMSHNALKSSPHASFPVEKYMDTSVPANEV
SSRAKEVSLYQGYTNPYQHVPGYIDM--VSTFGS-GEPRHETYISMEGYQSWTLANGWNS
QVYCAKDQAQSSHFWKSSFPGDVALNQPDMCVYRRXXXXXXXXXXXXXXXXXXXXXXXXX
XXXXXXXXXXXXXXXXXXXXXXXXXXXXXXXXXXXXXXXXXXXX
>gi387231677  gb_AFJ72549.1 homeobox B13, partial [Rhinolophus affinis]
XXXXXXXXXXXXXXXXXXXXXXXXXXXXXXXXXXXXXXXXXXXXXXXXXXXXXXXXXXXX
XXXXXXXXXXXXXXXXXVPFGYFGGGYYSCRVSRSSLKPCAQAATLAAYPTETPAAGEEY
PSRPTEFAFYPGYPGPYQPVASYLDVSVVQSLGAPGEPRHDALLPVDSYQPWALTGGWNS
QMCCXXXXXXXXXXXXXXXXXXXXXXXXXXXXXXXXXXXXXXXXXXXXXXXXXXXXXXXX
XXXXXXXXXXXXXXXXXXXXXXXXXXXXXXXXXXXXXXXXXXXX
>gi387231659  gb_AFJ72540.1 homeobox B13, partial [Rhinolophus marshalli]
XXXXXXXXXXXXXXXXXXXXXXXXXXXXXXXXXXXXXXXXXXXXXXXXXXXXXXXXXXXX
XXXXXXXXXXXXXXXXXVPFGYFGGGYYSCRVSRSSLKPCAQAATLAAYPTETPAAGEEY
PSRPTEFAFYPGYPGPYQPVASYLDVSVVQSLGAPGEPRHDALLPVDSYQPWAFTGGWNS
QMCCQAEXXXXXXXXXXXXXXXXXXXXXXXXXXXXXXXXXXXXXXXXXXXXXXXXXXXXX
XXXXXXXXXXXXXXXXXXXXXXXXXXXXXXXXXXXXXXXXXXXX
>gi325260872  gb_ADZ04662.1 homeobox C9 [Notophthalmus viridescens]
XXXXXXXXXXXXXXVDSLIGHEGDELLASRFPATGAHPAAARPSGLVPDCADFPSCSFAP
K------PTVFTTSWAPVPYSHHQPHITDARYVRSWLEPLSGAVSFPSFAATSRHYGDSF
PGRRGECG--PGDPRSY---PDYM-------YGSPGDFRDRTPQIIPSPESEALGSSKHK
DDKAELDPNNPVANWIHA---------------RSTRKKRCPYTKYQTLELEKEFLFNMY
LTRDRRYEVARVLNLTERQVKIWFQXXXXXXXXXXXXXXXXXXX
>gi387231661  gb_AFJ72541.1 homeobox B13, partial [Rhinolophus ferrumequinum]
XXXXXXXXXXXXXXXXXXXXXXXXXXXXXXXXXXXXXXXXXXXXXXXXXXXXXXXXXXXX
XXXXXXXXXXXXXXXXXXXGYFGGGYYSCRVSRSSLKPCAQAATLAAYPTETPAAGEEY
PSRPTEFAFYPGYPGPYQPVASYLDVSVVQSLAAPGEPRHDALLPVDSYQPWALTGGWNS
QMCCQAEXXXXXXXXXXXXXXXXXXXXXXXXXXXXXXXXXXXXXXXXXXXXXXXXXXXXX
XXXXXXXXXXXXXXXXXXXXXXXXXXXXXXXXXXXXXXXXXXXX
>gi387231655  gb_AFJ72538.1 homeobox B13, partial [Rhinolophus rex]
XXXXXXXXXXXXXXXXXXXXXXXXXXXXXXXXXXXXXXXXXXXXXXXXXXXXXXXXXXXX
XXXXXXXXXXXXXXXXXXXXXYFGGGYYSCRVSRSSLKPCAQAATLAAYPTETPAAGEEY
PSRPTEFAFYPGYPGPYQPVASYLDVSVVQSLGAPGEPRHDALLPVDSYQPWAFTGGWNS
QMCCQTEXXXXXXXXXXXXXXXXXXXXXXXXXXXXXXXXXXXXXXXXXXXXXXXXXXXXX
XXXXXXXXXXXXXXXXXXXXXXXXXXXXXXXXXXXXXXXXXXXX
>gi387231679  gb_AFJ72550.1 homeobox B13, partial [Hipposideros larvatus]
XXXXXXXXXXXXXXXXXXXXXXXXXXXXXXXXXXXXXXXXXXXXXXXXXXXXXXXXXXXX
XXXXXXXXXXXXXXXXXXXXXYFGGGYYSCRVSRSSLKPCAQAATLAAYPTETPAAGEEY
PSRPTEFAFYPGYPGPYQPVASYLDVSVVQSLGAPGEPRHDALLPVDSYQPWAFTGGWNS
QMCCQAEXXXXXXXXXXXXXXXXXXXXXXXXXXXXXXXXXXXXXXXXXXXXXXXXXXXXX
XXXXXXXXXXXXXXXXXXXXXXXXXXXXXXXXXXXXXXXXXXXX
>gi387231667  gb_AFJ72544.1 homeobox B13, partial [Chaerephon plicatus]
XXXXXXXXXXXXXXXXXXXXXXXXXXXXXXXXXXXXXXXXXXXXXXXXXXXXXXXXXXXX
XXXXXXXXXXXXXXXXXXXXXXFGGGYYSCRLSRSSLKPCAQAATLAAYPAETPGAGEEY
PSRHTEFAFYPGYPGPYQPVASYLDVSVVQTLGAPGEPRHDALMPVESYQPWALTGGWNS
QVCCQGEXXXXXXXXXXXXXXXXXXXXXXXXXXXXXXXXXXXXXXXXXXXXXXXXXXXXX
XXXXXXXXXXXXXXXXXXXXXXXXXXXXXXXXXXXXXXXXXXXX
>gi387231227  gb_AFJ72324.1 homeobox A13, partial [Hipposideros pomona]
XXXXXXXXXXXXXXXXXXXXXXXXXXXXXXXXXXXXXXXXXXXXXXXXXXXXXXXXXXXX
XXXXXXXXXXXXXXXXXXXXXXXXXXXXXXXXXXXXXXXXXXXXXXXXXXXXXXXXXXXX
XXXXXXXXXXXXXXXXXXXXXXXXXXXXXXXXXXXXXXXXXXXXXXXXXXXXXXXXXXXX
XXXXXXXXXXXXXXXXXXXXXXVVSHPSDSSSYRRGRKKRVPYTKVQLKELEREYATNKF
ITKDKRRRISATTNLSERQVTIWFQNRRVKEKKVINKLKTTSXX
>gi387231653  gb_AFJ72537.1 homeobox B13, partial [Rhinolophus paradoxolophus]
XXXXXXXXXXXXXXXXXXXXXXXXXXXXXXXXXXXXXXXXXXXXXXXXXXXXXXXXXXXX
XXXXXXXXXXXXXXXXXXXXXXXGGGYYSCRVSRSSLKPCAQAATLAAYPTETPAAGEEY
PSRPTEFAFYPGYPGPYQPVASYLDVSVVQSLGAPGEPRHDALLPVDSYQPWAFTGGWNS
QMCCQTEXXXXXXXXXXXXXXXXXXXXXXXXXXXXXXXXXXXXXXXXXXXXXXXXXXXXX
XXXXXXXXXXXXXXXXXXXXXXXXXXXXXXXXXXXXXXXXXXXX
>gi699575612  ref_XP_009876156.1 PREDICTED: homeobox protein Hox-A13-like,
XXXXXXXXXXXXXXXXXXXXXXXXXXXXXXXXXXXXXXXXXXXXXXXXXXXXXXXXXXXX
XXXXXXXXXXXXXXXXXXXXXXXXXXXXXXXXXXXXXXXXXXXXXXXXXXXXXXXXXXXX
```

```
XXXXXXXXXXXXXXXXXXXXXXXXXXXXXXXXXXXXXXXXXXXXXXXXXXXXXXXXXXXX
XXXXXXXXXXXXXLLDFSIPDVVS-HPPDANSYRRGRKKRVPYTKVQLKELEREYATNKF
ITKDKRRRISATTNLSERQVTIWFQNRRVKEKKVINKLKTTSXX
>gi676248171  gb_KFO12043.1 Homeobox protein Hox-A13, partial [Balearica
XXXXXXXXXXXXXXXXXXXXXXXXXXXXXXXXXXXXXXXXXXXXXXXXXXXXXXXXXXXX
XXXXXXXXXXXXXXXXXXXXXXXXXXXXXXXXXXXXXXXXXXXXXXXXXXXXXXXXXXXX
XXXXXXXXXXXXXXXXXXXXXXXXXXXXXXXXXXXXXXXXXXXXXXXXXXXXXXXXXXXX
XXXXXXXXXXXXXXXXXXXXXDVVSHPSDANSYRRGRKKRVPYTKVQLKELEREYATNKF
ITKDKRRRISATTNLSERQVTIWFQNRRVKEKKVINKLKXXXXX
>gi723145109  ref_XP_010289701.1 PREDICTED: LOW QUALITY PROTEIN: homeobox protein
XXXXXXXXXXXXXXXXXXXXXXXXXXXXXXXXXXXXXXXXXXXXXXXXXXXXXXXXXXXX
XXXXXXXXXXXXXXXXXXXXXXXXXXXXXXXXXXXXXXXXXXXXXXXXXXXXXXXXXXXX
XXXXXXXXXXXXXXXXXXXXXXXXXXXXXXXXXXXXXXXXXXXXXXXXXXXXXXXXXXXX
XXXXXXXXXXXXXXXXXXXXGDVALNQPDMCVYRRGRKKRVPYTKLQLKELENEYAINKF
INKDKRRRISAATNLSERQVTIWFQNRRVKDKKIVSKLKDNVSX
>gi697016891  ref_XP_009586001.1 PREDICTED: LOW QUALITY PROTEIN: homeobox protein
XXXXXXXXXXXXXXXXXXXXXXXXXXXXXXXXXXXXXXXXXXXXXXXXXXXXXXXXXXXX
XXXXXXXXXXXXXXXXXXXXXXXXXXXXXXXXXXXXXXXXXXXXXXXXXXXXXXXXXXXX
XXXXXXXXXXXXXXXXXXXXXXXXXXXXXXXXXXXXXXXXXXXXXXXXXXXXXXXXXXXX
XXXXXXXXXXXXXXXXXXXXGDVALNQPDMCVYRRGRKKRVPYTKLQLKELENEYAINKF
INKDKRRRISAATNLSERQVTIWFQNRRVKDKKIVSKLKDNVSX
>gi677147237  gb_KFP40928.1 Homeobox protein Hox-D13, partial [Chlamydotis
XXXXXXXXXXXXXXXXXXXXXXXXXXXXXXXXXXXXXXXXXXXXXXXXXXXXXXXXXXXX
XXXXXXXXXXXXXXXXXXXXXXXXXXXXXXXXXXXXXXXXXXXXXXXXXXXXXXXXXXXX
XXXXXXXXXXXXXXXXXXXXXXXXXXXXXXXXXXXXXXXXXXXXXXXXXXXXXXXXXXXX
XXXXXXXXXXXXXXXXXXXXGDVALNQPDMCVYRRGRKKRVPYTKLQLKELENEYAINKF
INKDKRRRISAATNLSERQVTIWFQNRRVKDKKIVSKLKDNVSX
>gi256014558  gb_ACU56833.1 HOXC13, partial [Ichthyophis cf. kohtaoensis
XXXXNERHRDKSPAMEGLCPSSHCRDLISHPALGRHSSAIGAHQGSIYTDISSPDAA---
RQCQ--APSAAAASNATMGYGYFGGSYYGCRLSHSQQKPCSYHPAEKYPEPSGSLPGEEL
SSRAKEFAFYPSFTGSYQAVPGYLDVSVAPGISGHSEPRHDALIPMEGYQHWALXXXXXX
XXXXXXXXXXXXXXXXXXXXXXXXXXXXXXXXXXXXXXXXXXXXXXXXXXXXXXXXXXXX
XXXXXXXXXXXXXXXXXXXXXXXXXXXXXXXXXXXXXXXXXXXX
>gi679242416  gb_KFW96016.1 Homeobox protein Hox-A13, partial [Phalacrocorax
XXXXXXXXXXXXXXXXXXXXXXXXXXXXXXXXXXXXXXXXXXXXXXXXXXXXXXXXXXXX
XXXXXXXXXXXXXXXXXXXXXXXXXXXXXXXXXXXXXXXXXXXXXXXXXXXXXXXXXXXX
XXXXXXXXXXXXXXXXXXXXXXXXXXXXXXXXXXXXXXXXXXXXXXXXXXXXXXXXXXXX
XXXXXXXXXXXXXXXXXXXXXDVVSHPSDANSYRRGRKKRVPYTKVQLKELEREYATNKF
ITKDKRRRISATTNLSERQVTIWFQNRRVKEKKVINKXXXXXXX
>gi683471095  gb_KFZ65304.1 Homeobox protein Hox-B13, partial [Caprimulgus
XXXXXXXXXXXXXXXXXXXXXXXXXXXXXXXXXXXXXXXXXXXXXXXXXXXXXXXXXXXX
XXXXXXXXXXXXXXXXXXXXXXXXXXXXXXXXXXXXXXXXXXXXXXXXXXXXXXXXXXXX
XXXXXXXXXXXXXXXXXXXXXXXXXXXXXXXXXXXXXXXXXXXXXXXXXXXXXXXXXXXX
XXXXXXXXXXXXXXXXXXXXXXXXXXXPDGCSFRRGRKKRVPYSKGQLKELEKEYASSKF
ITRDKRRKISAATNLTERQITIWFQNRRVKEKKVVAKIKSSNSX
>gi9438781  gb_AAB21366.2 homeobox gene, partial [Mus sp.]
XXXXXXXXXXXXXXXXXXXXXXXXXXXXXXXXXXXXXXXXXXXXXXXXXXXXXXXXXXXX
XXXXXXXXXXXXXXXXXXXXXXXXXXXXXXXXXXXXXXXXXXXXXXXXXXXXXXXXXXXX
XXXXXXXXXXXXXXXXXXXXXXXXXXXXXXXXXXXXXXXXXXXXXXXXXXXXXXXXXXXX
XXXXXXXXXXXXXXXXXXXXXDVALNQPDMCVYRRGRKKRVPYTKLQLKELENEYAINKF
INKDKRRTISRATNLSERQVTIWFQNRRVKDKKIVSKLKDTVSX
>gi676827072  gb_KFP20597.1 Homeobox protein Hox-B13, partial [Egretta garzetta]
XXXXXXXXXXXXXXXXXXXXXXXXXXXXXXXXXXXXXXXXXXXXXXXXXXXXXXXXXXXX
XXXXXXXXXXXXXXXXXXXXXXXXXXXXXXXXXXXXXXXXXXXXXXXXXXXXXXXXXXXX
XXXXXXXXXXXXXXXXXXXXXXXXXXXXXXXXXXXXXXXXXXXXXXXXXXXXXXXXXXXX
XXXXXXXXXXXXXXXXXXXXXXXXXXXPEGCSFRRGRKKRVPYSKGQLKELEREYASSKF
ITRDKRRKISAATNLTERQITIWFQNRRVKEKKVVAKIKSSSSX
>gi387231651  gb_AFJ72536.1 homeobox B13, partial [Myotis ricketti]
XXXXXXXXXXXXXXXXXXXXXXXXXXXXXXXXXXXXXXXXXXXXXXXXXXXXXXXXXXXX
XXXXXXXXXXXXXXXXXXXXXXXXXXXXSCRVSRSSLKSCAQAATLSAYPAETPPAGEEY
PSRPTEFAFYPGYPGSYQPMASYLDVSVVQTLGAPGEPRHEALLPMDSYQPWALTSGWNS
QMCCQGEXXXXXXXXXXXXXXXXXXXXXXXXXXXXXXXXXXXXXXXXXXXXXXXXXXXXX
XXXXXXXXXXXXXXXXXXXXXXXXXXXXXXXXXXXXXXXXXXXX
>gi677498936  gb_KFQ88341.1 Homeobox protein Hox-A13, partial [Phoenicopterus
XXXXXXXXXXXXXXXXXXXXXXXXXXXXXXXXXXXXXXXXXXXXXXXXXXXXXXXXXXXX
XXXXXXXXXXXXXXXXXXXXXXXXXXXXXXXXXXXXXXXXXXXXXXXXXXXXXXXXXXXX
XXXXXXXXXXXXXXXXXXXXXXXXXXXXXXXXXXXXXXXXXXXXXXXXXXXXXXXXXXXX
XXXXXXXXXXXXXXXXXXXXXXXXXXXXXXXXYRRGRKKRVPYTKVQLKELEREYATNKF
ITKDKRRRISATTNLSERQVTIWFQNRRVKEKKVINKLKXXXXX
```

```
>gi683474613  gb_KFZ68822.1 Homeobox protein Hox-D13, partial [Podiceps
XXXXXXXXXXXXXXXXXXXXXXXXXXXXXXXXXXXXXXXXXXXXXXXXXXXXXXXXXXXX
XXXXXXXXXXXXXXXXXXXXXXXXXXXXXXXXXXXXXXXXXXXXXXXXXXXXXXXXXXXX
XXXXXXXXXXXXXXXXXXXXXXXXXXXXXXXXXXXXXXXXXXXXXXXXXXXXXXXXXXXX
XXXXXXXXXXXXXXXXXXXXGDVALNQPDMCVYRRGRKKRVPYTKLQLKELENEYAINKF
INKDKRR--SPATNLSERQVTIWFQNRRVKDKKIVSKLKDNVSX
>gi387231675  gb_AFJ72548.1 homeobox B13, partial [Rhinolophus sinicus]
XXXXXXXXXXXXXXXXXXXXXXXXXXXXXXXXXXXXXXXXXXXXXXXXXXXXXXXXXXXX
XXXXXXXXXXXXXXXXXXXXXXXXXXXXXXXXXXXXXXPCAQAATLAAYPTETPAAGEEY
PSRPTEFAFYPGYPGSYQPATSYLDVSVVQSLGAPGEPRHDALLPVDSYQPWALTGGWNS
QMYCQGEXXXXXXXXXXXXXXXXXXXXXXXXXXXXXXXXXXXXXXXXXXXXXXXXXXXXX
XXXXXXXXXXXXXXXXXXXXXXXXXXXXXXXXXXXXXXXXXXXX
>gi677248448  gb_KFP58726.1 Homeobox protein Hox-C13, partial [Cariama cristata]
XXXXXXXXXXXXXXXXXXXXXXXXXXXXXXXXXXXXXXXXXXXXXXXXXXXXXXXXXXXX
XXXXXXXXXXXXXXXXXXXXXXXXXXXXXXXXXXXXXXXXXXXXXXXXXXXXXXXXXXXX
XXXXXXXXXXXXXXXXXXXXXXXXXXXXXXXXXXXXXXXXXXXXXXXXXXXXXXXXXXXX
XXXXXXXXXXXXXXXXXXXXXDVVPLQPEVSSYRRGRKKRVPYTKIQLKELEKEYAASKF
ITKEKRRRISATTNLSERQVTIWFQNRRXXXXXXXXXXXXXXXX
>gi678138507  gb_KFV07679.1 Homeobox protein Hox-B13, partial [Tauraco
XXXXXXXXXXXXXXXXXXXXXXXXXXXXXXXXXXXXXXXXXXXXXXXXXXXXXXXXXXXX
XXXXXXXXXXXXXXXXXXXXXXXXXXXXXXXXXXXXXXXXXXXXXXXXXXXXXXXXXXXX
XXXXXXXXXXXXXXXXXXXXXXXXXXXXXXXXXXXXXXXXXXXXXXXXXXXXXXXXXXXX
XXXXXXXXXXXXXXXXXXXXXXXXXXXXXXXXXXXXXXXXXXYSKGQLKELEKEYASSKF
ITRDKRRKISAATNLTERQITIWFQNRRVKEKKVVAKIKSSSSX
>gi700349256  ref_XP_009918725.1 PREDICTED: LOW QUALITY PROTEIN: homeobox protein
XXXXXXXXXXXXXXXXXXXXXXXXXXXXXXXXXXXXXXXXXXXXXXXXXXXXXXXXXXXX
XXXXXXXXXXXXXXXXXXXXXXXXXXXXXXXXXXXXXXXXXXXXXXXXXXXXXXXXXXXX
XXXXXXXXXXXXXXXXXXXXXXXXXXXXXXXXXXXXXXXXXXXXXXXXXXXXXXXXXXXX
XXXXXXXXXXXXXXXXXXXXXXVVPLQPEVSSYRRGRKKRVPYTKIQLKELEKEYAASKF
ITKEKRRRISATTNLSERQVTIWFQNRRXXXXXXXXXXXXXXXX
>gi677225924  gb_KFP51492.1 Homeobox protein Hox-C13a, partial [Cathartes aura]
XXXXXXXXXXXXXXXXXXXXXXXXXXXXXXXXXXXXXXXXXXXXXXXXXXXXXXXXXXXX
XXXXXXXXXXXXXXXXXXXXXXXXXXXXXXXXXXXXXXXXXXXXXXXXXXXXXXXXXXXX
XXXXXXXXXXXXXXXXXXXXXXXXXXXXXXXXXXXXXXXXXXXXXXXXXXXXXXXXXXXX
XXXXXXXXXXXXXXXXXXXXXXXXXXXXEVSSYRRGRKKRVPYTKIQLKELEKEYAASKF
ITKEKRRRISATTNLSERQVTIWFQNRRXXXXXXXXXXXXXXXX
>gi121308926  dbj_BAF43728.1 transcription factor Hox11/13c [Metacrinus rotundus]
XXXXXXXXXXXXXXXXXXXXXXXXXXXXXXXXXXXXXXXXXXXXXXXXXXXXXPSQSFGA
KTCNLPSPYQSRSASSFM------------QVATSSERSGPYHASFPYPRTDCRAPNEDH
PKRPSSLQFNPSLPSPMERYPSSMSSAAAMYSGASSQGQHH--LPYLNYPSCATSASY--
-FNMAGDGGPPG--WMFTFA-----------TFPR-RTKRRPYSKVQIFELEKEFQLHQY
LTRDRRARLSQSLTLSERQVKIWFQNRRMKQKKLNEKEKXXXXX
>gi194591945  gb_ACF76520.1 HoxA13b, partial [Carassius cuvieri]
XXXXXXXXXXXXXXXXXXXXXXXXXXXXXXXXXXXXXXXXXXXXXXXXXXXXXXXXXXXX
XXXXXXXXXXXXXXXXXXXXXXXXXXXXXXXXXXXXXXXXXXXXXXXXXXXXXXXXXXXX
XXXXXXXXXXXXXXXXXXXXXXXXXXXXXXXXXXXXXXXXXXXXXXXXXXXXXXXXXXXX
XXXXXXXXXXXXXXXXXXXXXXXXXXXXXXXXXXXXRKKRVPYTKVQLKELEREYATNKF
ITKDKRRRISAHTNLTERQVTIWFQNRSMXXXXXXXXXXXXXXX
>gi195549518  gb_ACG49987.1 HoxA13, partial [Tadorna tadorna]
XXXXXXXXXXXXXXXXXXXXXXXXXXXXXXXXXXXXXXXXXXXXXXXXXXXXXXXXXXXX
XXXXXXXXXXXXXXXXXXXXXXXXXXXXXXXXXXXXXXXXXXXXXXXXXXXXXXXXXXXX
XXXXXXXXXXXXXXXXXXXXXXXXXXXXXXXXXXXXXXXXXXXXXXXXXXXXXXXXXXXX
XXXXXXXXXXXXXXXXXXXXXXXXXXXXXXXXXXXXRKKSVPYTKVQLKELEREYATNKF
ITKDKRRRISATTNLSERQVTIWFQNRSMXXXXXXXXXXXXXXX
>gi194591943  gb_ACF76519.1 HoxA13b, partial [Carassius auratus x Cyprinus
XXXXXXXXXXXXXXXXXXXXXXXXXXXXXXXXXXXXXXXXXXXXXXXXXXXXXXXXXXXX
XXXXXXXXXXXXXXXXXXXXXXXXXXXXXXXXXXXXXXXXXXXXXXXXXXXXXXXXXXXX
XXXXXXXXXXXXXXXXXXXXXXXXXXXXXXXXXXXXXXXXXXXXXXXXXXXXXXXXXXXX
XXXXXXXXXXXXXXXXXXXXXXXXXXXXXXXXXXXXRKKRVPYTKVQLKELERECATNKF
ITKDKRRRISAHTNLTERQVTIWFQNSRMXXXXXXXXXXXXXXX
>gi387231665  gb_AFJ72543.1 homeobox B13, partial [Cynopterus sphinx]
XXXXXXXXXXXXXXXXXXXXXXXXXXXXXXXXXXXXXXXXXXXXXXXXXXXXXXXXXXXX
XXXXXXXXXXXXXXXXXXXXXXXXXXXXSCRVSRSSLKPCAQAATLTAYPAETP-AGDEY
PSRPTEFAFYPGYPGPYQPMASYLDVSVVQTLGAPGEPRHEALLPMDSYXXXXXXXXXXX
XXXXXXXXXXXXXXXXXXXXXXXXXXXXXXXXXXXXXXXXXXXXXXXXXXXXXXXXXXXX
XXXXXXXXXXXXXXXXXXXXXXXXXXXXXXXXXXXXXXXXXXXX
>gi195549516  gb_ACG49986.1 HoxA13, partial [Trachemys scripta elegans]
XXXXXXXXXXXXXXXXXXXXXXXXXXXXXXXXXXXXXXXXXXXXXXXXXXXXXXXXXXXX
XXXXXXXXXXXXXXXXXXXXXXXXXXXXXXXXXXXXXXXXXXXXXXXXXXXXXXXXXXXX
```

```
XXXXXXXXXXXXXXXXXXXXXXXXXXXXXXXXXXXXXXXXXXXXXXXXXXXXXXXXXXXX
XXXXXXXXXXXXXXXXXXXXXXXXXXXXXXXXXXXXRKKRVPYTKVQLKELEREYATNKF
ITKDKRRRISATTNLSERQVTIWFQXXXXXXXXXXXXXXXXXXX
>gi34304667  gb_AAQ63439.1 Hox13, partial [Oikopleura dioica]
XXXXXXXXXXXXXXXXXXXXXXXXXXXXXXXXXXXXXXXXXXXXXXXXXXXXXXXXXXXX
XXXXXXXXXXXXXXXXXXXXXXXXXXXXXXXXXXXXXXXXXXXXXXXXXXXXXXXXXXXX
XXXXXXXXXXXXXXXXXXXXXXXXXXXXXXXXXXXXXXXXXXXXXXXXXXXXXXXXXXXX
XXXXXXXXXXXXXXXXXXXXXXXXXXXXXXXXXXXXRKKRQPYSKQQTRQLEQEYAQNKF
ITRQKREQISRDLSLTDRQVKIWFQNRRVKEKKXXXXXXXXXXX
>gi194396133  gb_ACF60484.1 HoxA13b, partial [triploid hybrids of tetraploid male
XXXXXXXXXXXXXXXXXXXXXXXXXXXXXXXXXXXXXXXXXXXXXXXXXXXXXXXXXXXX
XXXXXXXXXXXXXXXXXXXXXXXXXXXXXXXXXXXXXXXXXXXXXXXXXXXXXXXXXXXX
XXXXXXXXXXXXXXXXXXXXXXXXXXXXXXXXXXXXXXXXXXXXXXXXXXXXXXXXXXXX
XXXXXXXXXXXXXXXXXXXXXXXXXXXXXXXXXXXXRKKSVPYTKVQLKDLEREYATNKF
ITKDKRRRISAHTNLTERQVTIWFQNSSMXXXXXXXXXXXXXXX
>gi387231669  gb_AFJ72545.1 homeobox B13, partial [Aselliscus stoliczkanus]
XXXXXXXXXXXXXXXXXXXXXXXXXXXXXXXXXXXXXXXXXXXXXXXXXXXXXXXXXXXX
XXXXXXXXXXXXXXXXXXXXXXXXXXXXSCRVSRSSLKPCTQAATLAAYPTETPAAGEEY
PSRPTEFAFYPGYPGSYQPVASYLDVSVVQSLGAPGEPRHDALXXXXXXXXXXXXXXXXX
XXXXXXXXXXXXXXXXXXXXXXXXXXXXXXXXXXXXXXXXXXXXXXXXXXXXXXXXXXXX
XXXXXXXXXXXXXXXXXXXXXXXXXXXXXXXXXXXXXXXXXXXX
>gi429510514  gb_AFZ94995.1 transcription factor Hox11 [Petromyzon marinus]
XXXXXXXXXXXXXXXXXXXXXXXXXXXXXXXXXXXXXXXXXXXXXXXXXXXXXXXXXXXX
XXXXXXXXXXXXXXXXXXXXXXXXXXXXXXXXSSAAYRDCLSAAMLMKSGETAAYSQARY
GSANTSYNFYGGKVGRTGVLPQAFDQFLLENSSEGGDEKSEQQRRQQQQQPKATQSPRDA
DAEAAGDAELSCGGGGGGGGGTEKPGGSSGAAVQRSRKKRCPYTKFQIRELEREFFFNVY
INKEKRLQLSRLLNLTDRQVKIWFQNRRMKEKKLNXXXXXXXXX
>gi780155006  ref_XP_011682244.1 PREDICTED: homeobox protein Hox-A10, partial
XXXXXXXXXXXXXXXXXXXXXXXXXXXXXXXXXXXXXXXXXXXXXXXXXXXXXXXXXXXX
XXXXXXXXXXXXXXXXXXXXXXXXXXXXXXXXXXXXXXXXXXXXXXXXXXXXXXXXXXXX
XXXXXXXXXXXXXXXXXXXXXXXXXXXXXXXXXSPPTSLGHHVLSTHGKHGYSSGSANPH
PACGNGDDDPSRP---ESDSDHSVSHQAVSASYTRTRKKRKPYTKFQTFELEKEFLYNMY
LTRDRRSHISRALSLTERQVKIWFQNRRMKLKKMRAREENXXXX
>gi387231671  gb_AFJ72546.1 homeobox B13, partial [Rhinolophus pusillus]
XXXXXXXXXXXXXXXXXXXXXXXXXXXXXXXXXXXXXXXXXXXXXXXXXXXXXXXXXXXX
XXXXXXXXXXXXXXXXXXXXXXXXXXXXXXXXXXXXXXPCAQAATLAAYPTETPAAGEEY
PSRPTEFAFYPGYPGPYQPVASYLDVSVVQSLGAAGEPRHDALLPVDSXXXXXXXXXXXX
XXXXXXXXXXXXXXXXXXXXXXXXXXXXXXXXXXXXXXXXXXXXXXXXXXXXXXXXXXXX
XXXXXXXXXXXXXXXXXXXXXXXXXXXXXXXXXXXXXXXXXXXX
>gi387231657  gb_AFJ72539.1 homeobox B13, partial [Taphozous melanopogon]
XXXXXXXXXXXXXXXXXXXXXXXXXXXXXXXXXXXXXXXXXXXXXXXXXXXXXXXXXXXX
XXXXXXXXXXXXXXXXXXXXXXXXXXXXXXXXXXXXXXXXXXXXXXXXYPTETPAAGEEY
PSRPTEFAFYPGYPGSYQPATSYLDVSVVQSLGAPGEPRHDALLPVDSYQPWALTGXXXX
XXXXXXXXXXXXXXXXXXXXXXXXXXXXXXXXXXXXXXXXXXXXXXXXXXXXXXXXXXXX
XXXXXXXXXXXXXXXXXXXXXXXXXXXXXXXXXXXXXXXXXXXX
>gi387231649  gb_AFJ72535.1 homeobox B13, partial [Hipposideros armiger]
XXXXXXXXXXXXXXXXXXXXXXXXXXXXXXXXXXXXXXXXXXXXXXXXXXXXXXXXXXXX
XXXXXXXXXXXXXXXXXXXXXXXXXXXXXXXXXXXXXXXCAQAATLAAYPTETLAAGEEY
PSRPTEFAFYPGYPGSYQPATSYLDVSVVQSLGAPGEPRHDALLPVDSXXXXXXXXXXXX
XXXXXXXXXXXXXXXXXXXXXXXXXXXXXXXXXXXXXXXXXXXXXXXXXXXXXXXXXXXX
XXXXXXXXXXXXXXXXXXXXXXXXXXXXXXXXXXXXXXXXXXXX
>gi229298086  gb_EEN68716.1 hypothetical protein BRAFLDRAFT_191424, partial
XXXXXXXXXXXXXXXXXXXXXXXXXXXXXXXXXXXXXXXXXXXXXXXXXXXXXXXXXXXX
XXXXXXXXXXXXXXXXXXXXXXXXXXXXXXXXXXXXXXXXXXXXXXXXXXXXXXXXXXXX
XXXXXXXXXXXXXXXXXXXXXXXXXXXXXXXXXXXXXXXXXXXXXXXXXXXXXXXXXXXX
XXXXXXXXXXXXXXXXXXXXXXXXXXXXXXXXXXRTRKKRRPYSKPQLALLEDEYASQKF
LTKEKRKEISESSSLSERQVMIWFQNRRMKEKKLXXXXXXXXXX
>gi328682725  gb_AEB33444.1 homeobox protein B13, partial [Crocodylus siamensis]
XXXXXXXXXXXXXXXXXXXXXXXXXXXXXXXXXXXXXXXXXXXXXXXXXXXXXXXXXXXX
XXXXXXXXXXXXXXXXXXXXXXXXXXXXXXXXXXXXXXXXXXXXXXXXXXXXXXXXXXXX
XXXXXXXXXXXXXXXXXXXXXXXXXXXXXXXXXXXXXXXXXXXXXXXXXXXXXXXXXXXX
XXXXXXXXXXXXXXXXXXXXXXXXXXXXXXXXXXXXXXXXXXYSKGQLKELEKEYASNKF
ITKDKRRKISAVTNLSERQITIXXXXXXXXXXXXXXXXXXXXXX
>gi217035843  gb_ACJ74394.1 Hox15 [Branchiostoma lanceolatum]
XXXXXXXXXXXXXXXXXXXXXXXXXXXXXXXXXXXXXXXXXXXXXXXXXXXXXXXXXXXX
XXXXXXXXXXXXXXXXXXXXXXXXXXXXXXXXXXXXXXXXXXXXXXXXXXXXXXXXXXXX
XXXXXXXXXXXXXXXXXXXXXXXXXXXXXXXXXXXXXXXXXXXXXXXXXXXXXXXXXXXX
XXXXXXXXXXXXXXXXXXXXXXXXXXXXXXXXXXXXXXXXXXYSKPQLALLEDEYASQKF
LTKEKRKEISESSSLSERQVMIWFQNRRMKEKKLXXXXXXXXXX
```
