# Supplementary material for: Computational Modeling of complete HOXB13 protein for predicting the functional effect of SNPs and the associated role in hereditary prostate cancer
Source: Sci Rep. 2017 Mar 8;7:43830. doi: 10.1038/srep43830 (PMC5363706; doi:10.1038/srep43830)
Supplement: Supplementary Dataset [file srep43830-s1.zip › SR Supplementary Data Files Complete/SIFT/SIFT Predictions for Query SNP.pdf]

Predictions

Substitution at pos 3 from P to T is predicted to AFFECT PROTEIN FUNCTION with a score of 0.02.
Median sequence conservation: 3.41
Sequences represented at this position:29
WARNING!! This substitution may have been predicted to affect function just because the sequences used were not diverse enough. **There is LOW CONFIDENCE in this prediction.**

Substitution at pos 4 from G to V is predicted to AFFECT PROTEIN FUNCTION with a score of 0.00.
Median sequence conservation: 3.41
Sequences represented at this position:29
WARNING!! This substitution may have been predicted to affect function just because the sequences used were not diverse enough. **There is LOW CONFIDENCE in this prediction.**

Substitution at pos 6 from Y to F is predicted to be TOLERATED with a score of 0.61.
Median sequence conservation: 3.35
Sequences represented at this position:38

Substitution at pos 8 from T to I is predicted to be TOLERATED with a score of 0.18.
Median sequence conservation: 3.35
Sequences represented at this position:41

Substitution at pos 21 from A to V is predicted to AFFECT PROTEIN FUNCTION with a score of 0.01.
Median sequence conservation: 2.96
Sequences represented at this position:105

Substitution at pos 22 from G to R is predicted to AFFECT PROTEIN FUNCTION with a score of 0.05.
Median sequence conservation: 2.96
Sequences represented at this position:108

Substitution at pos 23 from G to R is predicted to be TOLERATED with a score of 0.37.
Median sequence conservation: 2.95
Sequences represented at this position:112

Substitution at pos 24 from G to R is predicted to AFFECT PROTEIN FUNCTION with a score of 0.01.
Median sequence conservation: 2.95
Sequences represented at this position:112

Substitution at pos 25 from R to Q is predicted to AFFECT PROTEIN FUNCTION with a score of 0.01.
Median sequence conservation: 2.95
Sequences represented at this position:112

Substitution at pos 29 from A to S is predicted to be TOLERATED with a score of 0.47.
Median sequence conservation: 2.95
Sequences represented at this position:112

Substitution at pos 30 from H to R is predicted to be TOLERATED with a score of 0.15.
Median sequence conservation: 2.95
Sequences represented at this position:112

Substitution at pos 31 from S to C is predicted to be TOLERATED with a score of 0.18.
Median sequence conservation: 3.02
Sequences represented at this position:86

Substitution at pos 33 from L to M is predicted to be TOLERATED with a score of 0.07.
Median sequence conservation: 2.95
Sequences represented at this position:111

Substitution at pos 36 from H to N is predicted to be TOLERATED with a score of 0.22.
Median sequence conservation: 2.97
Sequences represented at this position:103

Substitution at pos 38 from A to E is predicted to be TOLERATED with a score of 0.05.
Median sequence conservation: 3.00
Sequences represented at this position:108

Substitution at pos 39 from A to S is predicted to be TOLERATED with a score of 0.98.

```
     Median sequence conservation: 3.00
     Sequences represented at this position:109

Substitution at pos 41 from T to M is predicted to AFFECT PROTEIN FUNCTION with a score of 0.03.
     Median sequence conservation: 3.00
     Sequences represented at this position:80

Substitution at pos 47 from N to S is predicted to be TOLERATED with a score of 1.00.
     Median sequence conservation: 2.99
     Sequences represented at this position:111

Substitution at pos 49 from A to P is predicted to be TOLERATED with a score of 0.33.
     Median sequence conservation: 2.99
     Sequences represented at this position:111

Substitution at pos 54 from P to S is predicted to be TOLERATED with a score of 0.54.
     Median sequence conservation: 2.88
     Sequences represented at this position:114

Substitution at pos 57 from A to S is predicted to be TOLERATED with a score of 0.58.
     Median sequence conservation: 2.90
     Sequences represented at this position:103

Substitution at pos 59 from P to L is predicted to AFFECT PROTEIN FUNCTION with a score of 0.02.
     Median sequence conservation: 2.90
     Sequences represented at this position:114

Substitution at pos 61 from K to M is predicted to AFFECT PROTEIN FUNCTION with a score of 0.00.
     Median sequence conservation: 2.90
     Sequences represented at this position:121

Substitution at pos 63 from C to G is predicted to AFFECT PROTEIN FUNCTION with a score of 0.03.
     Median sequence conservation: 3.05
     Sequences represented at this position:119

Substitution at pos 66 from C to R is predicted to AFFECT PROTEIN FUNCTION with a score of 0.00.
     Median sequence conservation: 3.11
     Sequences represented at this position:101

Substitution at pos 67 from P to S is predicted to be TOLERATED with a score of 0.85.
     Median sequence conservation: 3.08
     Sequences represented at this position:109

Substitution at pos 69 from V to L is predicted to be TOLERATED with a score of 0.21.
     Median sequence conservation: 2.90
     Sequences represented at this position:112

Substitution at pos 70 from P to T is predicted to be TOLERATED with a score of 0.61.
     Median sequence conservation: 2.91
     Sequences represented at this position:102

Substitution at pos 72 from G to V is predicted to be TOLERATED with a score of 0.05.
     Median sequence conservation: 2.90
     Sequences represented at this position:121

Substitution at pos 73 from T to M is predicted to AFFECT PROTEIN FUNCTION with a score of 0.00.
     Median sequence conservation: 2.91
     Sequences represented at this position:123

Substitution at pos 78 from V to L is predicted to be TOLERATED with a score of 1.00.
     Median sequence conservation: 2.90
     Sequences represented at this position:131

Substitution at pos 80 from Y to C is predicted to AFFECT PROTEIN FUNCTION with a score of 0.00.
     Median sequence conservation: 2.93
     Sequences represented at this position:134

Substitution at pos 84 from G to E is predicted to AFFECT PROTEIN FUNCTION with a score of 0.00.
     Median sequence conservation: 2.96
```

```
    Sequences represented at this position:134

Substitution at pos 85 from G to S is predicted to be TOLERATED with a score of 0.91.
    Median sequence conservation: 2.96
    Sequences represented at this position:136

Substitution at pos 93 from S to A is predicted to be TOLERATED with a score of 0.33.
    Median sequence conservation: 2.79
    Sequences represented at this position:147

Substitution at pos 94 from R to Q is predicted to be TOLERATED with a score of 0.10.
    Median sequence conservation: 2.77
    Sequences represented at this position:141

Substitution at pos 97 from L to M is predicted to AFFECT PROTEIN FUNCTION with a score of 0.03.
    Median sequence conservation: 2.78
    Sequences represented at this position:141

Substitution at pos 99 from P to T is predicted to be TOLERATED with a score of 0.32.
    Median sequence conservation: 2.78
    Sequences represented at this position:149

Substitution at pos 101 from A to V is predicted to AFFECT PROTEIN FUNCTION with a score of 0.04.
    Median sequence conservation: 2.79
    Sequences represented at this position:152

Substitution at pos 103 from A to V is predicted to be TOLERATED with a score of 0.10.
    Median sequence conservation: 2.78
    Sequences represented at this position:154

Substitution at pos 105 from T to I is predicted to be TOLERATED with a score of 0.34.
    Median sequence conservation: 2.78
    Sequences represented at this position:155

Substitution at pos 106 from L to R is predicted to be TOLERATED with a score of 0.32.
    Median sequence conservation: 2.78
    Sequences represented at this position:155

Substitution at pos 107 from A to P is predicted to be TOLERATED with a score of 0.34.
    Median sequence conservation: 2.78
    Sequences represented at this position:156

Substitution at pos 108 from A to V is predicted to be TOLERATED with a score of 0.09.
    Median sequence conservation: 2.78
    Sequences represented at this position:157

Substitution at pos 110 from P to A is predicted to be TOLERATED with a score of 1.00.
    Median sequence conservation: 2.78
    Sequences represented at this position:158

Substitution at pos 111 from A to T is predicted to be TOLERATED with a score of 0.46.
    Median sequence conservation: 2.78
    Sequences represented at this position:161

Substitution at pos 114 from P to T is predicted to be TOLERATED with a score of 0.56.
    Median sequence conservation: 2.78
    Sequences represented at this position:165

Substitution at pos 115 from T to M is predicted to be TOLERATED with a score of 0.06.
    Median sequence conservation: 2.78
    Sequences represented at this position:166

Substitution at pos 117 from G to R is predicted to AFFECT PROTEIN FUNCTION with a score of 0.00.
    Median sequence conservation: 2.77
    Sequences represented at this position:169

Substitution at pos 121 from P to A is predicted to be TOLERATED with a score of 0.09.
    Median sequence conservation: 2.76
    Sequences represented at this position:168
```

```
Substitution at pos 122 from S to R is predicted to AFFECT PROTEIN FUNCTION with a score of 0.00.
    Median sequence conservation: 2.76
    Sequences represented at this position:168

Substitution at pos 123 from R to H is predicted to AFFECT PROTEIN FUNCTION with a score of 0.01.
    Median sequence conservation: 2.76
    Sequences represented at this position:168

Substitution at pos 124 from P to R is predicted to be TOLERATED with a score of 0.19.
    Median sequence conservation: 2.77
    Sequences represented at this position:169

Substitution at pos 128 from A to V is predicted to AFFECT PROTEIN FUNCTION with a score of 0.00.
    Median sequence conservation: 2.76
    Sequences represented at this position:172

Substitution at pos 135 from G to E is predicted to AFFECT PROTEIN FUNCTION with a score of 0.01.
    Median sequence conservation: 2.76
    Sequences represented at this position:172

Substitution at pos 136 from T to P is predicted to be TOLERATED with a score of 1.00.
    Median sequence conservation: 2.76
    Sequences represented at this position:172

Substitution at pos 137 from Y to S is predicted to AFFECT PROTEIN FUNCTION with a score of 0.01.
    Median sequence conservation: 2.76
    Sequences represented at this position:172

Substitution at pos 138 from Q to H is predicted to AFFECT PROTEIN FUNCTION with a score of 0.01.
    Median sequence conservation: 2.84
    Sequences represented at this position:171

Substitution at pos 141 from A to V is predicted to AFFECT PROTEIN FUNCTION with a score of 0.00.
    Median sequence conservation: 2.76
    Sequences represented at this position:171

Substitution at pos 146 from V to M is predicted to be TOLERATED with a score of 0.52.
    Median sequence conservation: 2.84
    Sequences represented at this position:170

Substitution at pos 152 from L to M is predicted to be TOLERATED with a score of 0.08.
    Median sequence conservation: 2.75
    Sequences represented at this position:170

Substitution at pos 153 from G to S is predicted to be TOLERATED with a score of 0.45.
    Median sequence conservation: 2.75
    Sequences represented at this position:170

Substitution at pos 154 from A to V is predicted to AFFECT PROTEIN FUNCTION with a score of 0.01.
    Median sequence conservation: 2.73
    Sequences represented at this position:169

Substitution at pos 162 from S to C is predicted to AFFECT PROTEIN FUNCTION with a score of 0.01.
    Median sequence conservation: 2.81
    Sequences represented at this position:170

Substitution at pos 167 from D to N is predicted to AFFECT PROTEIN FUNCTION with a score of 0.01.
    Median sequence conservation: 2.74
    Sequences represented at this position:171

Substitution at pos 175 from A to S is predicted to be TOLERATED with a score of 0.86.
    Median sequence conservation: 2.73
    Sequences represented at this position:154

Substitution at pos 176 from G to R is predicted to AFFECT PROTEIN FUNCTION with a score of 0.01.
    Median sequence conservation: 2.72
    Sequences represented at this position:159
```

Substitution at pos 177 from G to V is predicted to AFFECT PROTEIN FUNCTION with a score of 0.00.
Median sequence conservation: 2.73
Sequences represented at this position:158

Substitution at pos 181 from Q to R is predicted to AFFECT PROTEIN FUNCTION with a score of 0.00.
Median sequence conservation: 2.80
Sequences represented at this position:160

Substitution at pos 182 from M to T is predicted to AFFECT PROTEIN FUNCTION with a score of 0.05.
Median sequence conservation: 2.73
Sequences represented at this position:162

Substitution at pos 188 from Q to R is predicted to AFFECT PROTEIN FUNCTION with a score of 0.00.
Median sequence conservation: 2.73
Sequences represented at this position:160

Substitution at pos 194 from F to I is predicted to be TOLERATED with a score of 0.13.
Median sequence conservation: 2.87
Sequences represented at this position:142

Substitution at pos 197 from A to T is predicted to be TOLERATED with a score of 0.16.
Median sequence conservation: 2.75
Sequences represented at this position:144

Substitution at pos 200 from A to S is predicted to be TOLERATED with a score of 0.23.
Median sequence conservation: 2.85
Sequences represented at this position:143

Substitution at pos 205 from Q to R is predicted to be TOLERATED with a score of 0.87.
Median sequence conservation: 2.91
Sequences represented at this position:98

Substitution at pos 210 from A to V is predicted to be TOLERATED with a score of 0.29.
Median sequence conservation: 2.94
Sequences represented at this position:151

Substitution at pos 212 from A to T is predicted to be TOLERATED with a score of 0.32.
Median sequence conservation: 2.86
Sequences represented at this position:155

Substitution at pos 215 from R to C is predicted to AFFECT PROTEIN FUNCTION with a score of 0.00.
Median sequence conservation: 2.74
Sequences represented at this position:158

Substitution at pos 216 from G to C is predicted to AFFECT PROTEIN FUNCTION with a score of 0.00.
Median sequence conservation: 2.82
Sequences represented at this position:156

Substitution at pos 217 from R to C is predicted to AFFECT PROTEIN FUNCTION with a score of 0.00.
Median sequence conservation: 2.73
Sequences represented at this position:163

Substitution at pos 218 from K to R is predicted to AFFECT PROTEIN FUNCTION with a score of 0.00.
Median sequence conservation: 2.73
Sequences represented at this position:163

Substitution at pos 222 from P to R is predicted to AFFECT PROTEIN FUNCTION with a score of 0.00.
Median sequence conservation: 2.73
Sequences represented at this position:163

Substitution at pos 227 from Q to E is predicted to AFFECT PROTEIN FUNCTION with a score of 0.00.
Median sequence conservation: 2.73
Sequences represented at this position:166

Substitution at pos 229 from R to W is predicted to AFFECT PROTEIN FUNCTION with a score of 0.00.
Median sequence conservation: 2.73
Sequences represented at this position:166

Substitution at pos 233 from R to R is predicted to be TOLERATED with a score of 0.90.

```
    Median sequence conservation: 2.73
    Sequences represented at this position:166

Substitution at pos 236 from A to E is predicted to AFFECT PROTEIN FUNCTION with a score of 0.01.
    Median sequence conservation: 2.73
    Sequences represented at this position:166

Substitution at pos 239 from K to Q is predicted to be TOLERATED with a score of 0.07.
    Median sequence conservation: 2.73
    Sequences represented at this position:166

Substitution at pos 244 from D to N is predicted to AFFECT PROTEIN FUNCTION with a score of 0.00.
    Median sequence conservation: 2.73
    Sequences represented at this position:166

Substitution at pos 246 from R to K is predicted to AFFECT PROTEIN FUNCTION with a score of 0.00.
    Median sequence conservation: 2.74
    Sequences represented at this position:165

Substitution at pos 247 from R to C is predicted to AFFECT PROTEIN FUNCTION with a score of 0.00.
    Median sequence conservation: 2.74
    Sequences represented at this position:165

Substitution at pos 250 from S to L is predicted to AFFECT PROTEIN FUNCTION with a score of 0.00.
    Median sequence conservation: 2.74
    Sequences represented at this position:164

Substitution at pos 253 from T to P is predicted to AFFECT PROTEIN FUNCTION with a score of 0.01.
    Median sequence conservation: 2.74
    Sequences represented at this position:161

Substitution at pos 254 from S to T is predicted to be TOLERATED with a score of 0.08.
    Median sequence conservation: 2.74
    Sequences represented at this position:161

Substitution at pos 258 from R to C is predicted to AFFECT PROTEIN FUNCTION with a score of 0.00.
    Median sequence conservation: 2.74
    Sequences represented at this position:161

Substitution at pos 263 from W to R is predicted to AFFECT PROTEIN FUNCTION with a score of 0.00.
    Median sequence conservation: 2.79
    Sequences represented at this position:158

Substitution at pos 267 from R to C is predicted to AFFECT PROTEIN FUNCTION with a score of 0.00.
    Median sequence conservation: 2.88
    Sequences represented at this position:152

Substitution at pos 268 from R to Q is predicted to AFFECT PROTEIN FUNCTION with a score of 0.00.
    Median sequence conservation: 2.88
    Sequences represented at this position:152

Substitution at pos 274 from V to F is predicted to be TOLERATED with a score of 0.32.
    Median sequence conservation: 2.95
    Sequences represented at this position:126

Substitution at pos 276 from A to T is predicted to be TOLERATED with a score of 0.19.
    Median sequence conservation: 3.07
    Sequences represented at this position:123

Substitution at pos 278 from V to L is predicted to be TOLERATED with a score of 0.74.
    Median sequence conservation: 3.07
    Sequences represented at this position:121

Substitution at pos 281 from S to T is predicted to be TOLERATED with a score of 0.51.
    Median sequence conservation: 3.32
    Sequences represented at this position:107

Substitution at pos 282 from A to T is predicted to be TOLERATED with a score of 0.65.
    Median sequence conservation: 3.32
```

Sequences represented at this position:105
