# Supplementary material for: Computational Modeling of complete HOXB13 protein for predicting the functional effect of SNPs and the associated role in hereditary prostate cancer
Source: Sci Rep. 2017 Mar 8;7:43830. doi: 10.1038/srep43830 (PMC5363706; doi:10.1038/srep43830)
Supplement: Supplementary Dataset [file srep43830-s1.zip › SR Supplementary Data Files Complete/SIFT/SIFT results overview.pdf]

SIFT Results (BLink)

Results will be stored at http://sift.bii.a-star.edu.sg//sift-bin/format.pl?60d3dcc1e5_BLink for 24 hours

Please go to this link if your results do not appear below after 5 minutes.

A notification will be sent to gopalakrishnan.cnu@gmail.com once the results are ready for viewing.

Working................
275 sequences were selected to be closely related to your query sequence.
PSIBLAST alignment of submitted sequences
Alignment in FASTA format (for modification)
The alignment taken from PSIBLAST is returned in msf format.
Note: **X**es are placeholders at the beginning and end of sequences. While **-** means a gap in the alignment an X means a lack of information such as a partial alignment or incomplete sequence and do not contribute to the prediction.
Please check the sequences that have been chosen. If the sequences are too diverged from your query or the alignment is questionable, we suggest you modify the fasta-formatted file above and resubmit.

SIFT amino acid predictions for:
Positions 1 to 100
Positions 101 to 200
Positions 201 to 300

Scaled Probabilities for Entire Protein
May take some time to load!! *Please be patient if you do not see the table immediately.*
Amino acids with probabilities < .05 are predicted to be deleterious.

Predictions of substitutions entered
If you received a warning that the sequences were not diverse enough, you can have SIFT choose more diverse sequences here.

*** The following sequences have been removed because they were found to be over 90% identical with your protein query: gi685597136, gi60653709, gi795250298, gi635093043, gi109114206, gi795583451, gi387231671, gi559812841, gi332259422, gi387231669, gi795525940, gi426237811, gi744588246, gi387231665, gi444517714, gi640808225, gi667260003, gi471380475, gi674059771, gi344285419, gi852732630, gi512996173, gi395826626, gi528767552, gi532208489, gi387231651, gi507654822, gi488527225, gi817274366, gi585671866, gi532071900, gi593727116, gi470606860, gi291405863, gi594656397, gi634851243, gi387231653, gi602709820, gi387231667, gi387231679, gi387231655, gi387231661, gi826271801, gi387231659, gi387231677, gi589917310, gi387231673, gi507543189, gi725576032, gi742135928, gi348562271, gi594050844, gi533160875, gi556738344, gi803246204, gi354474760, gi532051321, gi311267502, gi743729506, gi586448761, gi504159820, gi524951862, gi478520959, gi664747354, gi359076815, gi759185571, gi586539979, gi157823841, gi410980861, gi829772881, gi1519038, gi731207261, gi560984071, gi73966255, gi752394348, gi507952779, gi670977729,.

WARNING: Original amino acid T at position 136 is not allowed by the prediction.
