# Supplementary material for: Computational Modeling of complete HOXB13 protein for predicting the functional effect of SNPs and the associated role in hereditary prostate cancer
Source: Sci Rep. 2017 Mar 8;7:43830. doi: 10.1038/srep43830 (PMC5363706; doi:10.1038/srep43830)
Supplement: Supplementary Dataset [file srep43830-s1.zip › SR Supplementary Data Files Complete/SIFT/SIFT Scaled probabilities for entire protein.pdf]

Probabilities

Each row corresponds to a position in the reference protein. Below each position is the fraction of sequences that contain one of the basic amino acids. A low fraction indicates the position is either severely gapped or unalignable and has little information. Expect poor prediction at these positions.
Each column corresponds to one of the twenty amino acids.
Each entry contains the score at a particular position (row) for an amino acid substitution (column).
Substitutions predicted to be intolerant are highlighted in red.

pos	A	C	D	E	F	G	H	I	K	L	M	N	P	Q	R	S	T	V	W	Y
1M 0.11	0.00	0.00	0.00	0.00	0.00	0.00	0.00	0.00	0.00	0.00	1.00	0.00	0.00	0.00	0.00	0.00	0.00	0.00	0.00	0.00
2E 0.11	0.01	0.00	0.23	1.00	0.00	0.00	0.00	0.00	0.01	0.00	0.00	0.00	0.01	0.01	0.00	0.00	0.00	0.00	0.00	0.00
3P 0.15	0.28	0.01	0.01	0.01	0.00	0.03	0.00	0.00	0.01	0.01	0.00	0.07	1.00	0.00	0.00	0.17	0.02	0.01	0.00	0.00
4G 0.15	0.02	0.00	0.01	0.00	0.00	1.00	0.00	0.00	0.00	0.00	0.00	0.00	0.05	0.00	0.00	0.01	0.00	0.00	0.00	0.00
5N 0.18	0.84	0.07	0.69	0.93	0.21	0.41	0.15	0.20	1.00	0.31	0.11	0.95	0.33	0.56	0.50	0.98	0.94	0.30	0.03	0.08
6Y 0.19	0.47	0.14	0.18	0.39	0.61	0.26	0.49	0.30	0.23	0.50	0.17	0.24	0.16	0.24	0.27	0.31	0.30	0.39	0.14	1.00
7A 0.20	1.00	0.05	0.15	0.11	0.03	0.26	0.05	0.05	0.18	0.08	0.03	0.37	0.26	0.10	0.15	0.48	0.50	0.12	0.01	0.04
8T 0.21	0.79	0.06	0.24	0.33	0.12	0.23	0.15	0.18	0.33	0.20	0.07	0.52	0.16	0.23	0.21	0.98	1.00	0.24	0.04	0.16
9L 0.21	0.67	0.11	0.22	0.32	0.43	0.25	0.41	0.28	0.42	1.00	0.35	0.26	0.17	0.48	0.41	0.33	0.32	0.35	0.11	0.60
10D 0.49	0.14	0.00	1.00	0.83	0.00	0.01	0.00	0.00	0.01	0.00	0.00	0.18	0.01	0.02	0.00	0.01	0.04	0.00	0.00	0.00
11G 0.49	0.78	0.07	0.62	1.00	0.11	0.40	0.16	0.22	0.93	0.35	0.12	0.51	0.28	0.60	0.54	0.75	0.59	0.32	0.03	0.12
12A 0.46	0.79	0.07	0.12	0.13	0.19	0.25	0.13	0.15	0.15	0.23	0.07	1.00	0.13	0.12	0.12	0.83	0.66	0.18	0.05	0.22
13K 0.46	0.11	0.00	0.03	0.02	0.00	0.01	0.00	0.00	1.00	0.01	0.00	0.01	0.06	0.08	0.04	0.01	0.01	0.02	0.00	0.00
14D 0.46	0.26	0.01	0.44	0.14	0.01	0.10	0.03	0.13	0.05	0.01	0.00	1.00	0.03	0.04	0.03	0.19	0.05	0.01	0.00	0.02
15I 0.52	0.00	0.00	0.00	0.00	0.01	0.06	0.00	0.10	0.00	0.17	1.00	0.00	0.00	0.00	0.00	0.02	0.02	0.23	0.00	0.00
16E 0.52	0.01	0.00	0.26	1.00	0.00	0.08	0.00	0.00	0.01	0.00	0.00	0.01	0.00	0.01	0.00	0.02	0.06	0.00	0.00	0.00
17G 0.52	0.01	0.00	0.01	0.00	0.00	1.00	0.00	0.00	0.00	0.00	0.00	0.08	0.00	0.00	0.00	0.16	0.00	0.02	0.00	0.00
18L 0.52	0.01	0.00	0.00	0.00	0.43	0.00	0.00	0.03	0.00	1.00	0.02	0.02	0.00	0.00	0.00	0.07	0.00	0.03	0.00	0.00
19L 0.52	0.58	0.30	0.17	0.23	0.61	0.27	0.56	0.53	0.32	0.78	0.30	0.26	0.35	0.25	0.37	0.50	0.37	1.00	0.15	0.83
20G 0.53	0.17	0.00	0.00	0.00	0.00	1.00	0.00	0.00	0.00	0.00	0.00	0.00	0.10	0.00	0.00	0.04	0.00	0.00	0.00	0.00
21A 0.53	1.00	0.01	0.15	0.10	0.00	0.40	0.18	0.00	0.03	0.01	0.00	0.09	0.02	0.02	0.02	0.30	0.10	0.01	0.00	0.01
22G 0.54	0.19	0.03	0.26	0.28	0.02	0.81	0.06	0.02	0.09	0.03	0.01	1.00	0.07	0.06	0.05	0.53	0.32	0.04	0.01	0.03
23G 0.56	0.69	0.19	0.23	0.30	0.74	0.49	0.58	0.40	0.33	0.68	0.23	0.34	0.25	0.41	0.37	0.50	0.43	0.53	0.19	1.00
24G 0.56	0.41	1.00	0.16	0.02	0.01	0.17	0.00	0.01	0.02	0.02	0.01	0.02	0.08	0.01	0.01	0.24	0.10	0.03	0.00	0.01
25R 0.56	0.27	0.01	0.01	0.13	0.00	0.08	0.00	0.00	0.01	0.01	0.00	0.01	0.02	0.01	1.00	0.08	0.01	0.01	0.00	0.00
pos	A	C	D	E	F	G	H	I	K	L	M	N	P	Q	R	S	T	V	W	Y
26N 0.56	0.02	0.00	0.20	0.10	0.00	0.03	0.08	0.00	0.05	0.13	0.00	1.00	0.01	0.02	0.06	0.03	0.06	0.01	0.00	0.01
27L 0.56	0.01	0.00	0.00	0.01	0.06	0.00	0.05	0.16	0.01	1.00	0.03	0.01	0.02	0.22	0.02	0.01	0.01	0.03	0.00	0.01
28V 0.56	0.44	0.51	0.01	0.03	0.07	0.01	0.01	0.84	0.03	0.65	1.00	0.02	0.03	0.02	0.02	0.03	0.11	0.99	0.01	0.11
29A 0.56	1.00	0.03	0.02	0.03	0.01	0.20	0.01	0.01	0.03	0.03	0.01	0.02	0.20	0.02	0.34	0.47	0.07	0.06	0.00	0.01
30H 0.56	0.00	0.00	0.01	0.00	0.00	0.01	1.00	0.00	0.01	0.00	0.00	0.32	0.00	0.00	0.15	0.03	0.00	0.00	0.00	0.00
31S 0.43	0.52	0.18	0.21	0.27	0.73	0.33	0.54	0.40	0.29	0.68	0.22	0.31	0.32	0.29	0.35	0.54	0.40	0.50	0.18	1.00
32P 0.44	1.00	0.03	0.01	0.02	0.02	0.05	0.01	0.11	0.02	0.13	0.11	0.01	0.42	0.02	0.02	0.08	0.09	0.37	0.00	0.01
33L 0.56	0.40	0.04	0.03	0.05	0.13	0.09	0.11	0.14	0.05	1.00	0.07	0.05	0.13	0.05	0.05	0.25	0.10	0.19	0.03	0.12
34T 0.54	0.54	0.19	0.21	0.27	0.71	0.48	0.55	0.41	0.29	0.72	0.25	0.31	0.21	0.29	0.35	0.43	0.45	0.50	0.18	1.00
35S 0.55	1.00	0.03	0.02	0.02	0.01	0.87	0.00	0.01	0.03	0.04	0.01	0.02	0.11	0.02	0.39	0.56	0.08	0.04	0.00	0.01
36H 0.52	0.92	0.07	0.17	0.15	0.11	0.46	1.00	0.08	0.33	0.15	0.06	0.22	0.29	0.16	0.33	0.34	0.17	0.15	0.03	0.20
37P 0.54	0.04	0.00	0.01	0.01	0.04	0.01	0.13	0.01	0.01	0.03	0.00	0.01	1.00	0.02	0.01	0.24	0.01	0.01	0.00	0.01
38A 0.54	1.00	0.05	0.05	0.05	0.02	0.68	0.01	0.03	0.06	0.09	0.03	0.05	0.35	0.06	0.04	0.90	0.22	0.09	0.01	0.02

**39A 0.55**	1.00	0.04	0.05	0.04	0.02	0.45	0.01	0.03	0.05	0.05	0.02	0.06	0.46	0.04	0.07	0.98	0.57	0.08	0.01	0.08
**40P 0.47**	0.81	0.14	0.11	0.15	0.18	0.19	0.12	0.53	0.17	0.58	0.16	0.16	0.25	0.14	0.13	0.55	0.81	1.00	0.05	0.18
**41T 0.40**	1.00	0.01	0.01	0.01	0.10	0.23	0.02	0.02	0.01	0.03	0.03	0.01	0.05	0.01	0.01	0.11	0.17	0.10	0.01	0.06
**42L 0.53**	0.61	0.18	0.21	0.28	0.71	0.34	0.56	0.39	0.30	0.79	0.23	0.31	0.27	0.30	0.40	0.41	0.44	0.57	0.18	1.00
**43M 0.53**	0.51	0.18	0.20	0.27	0.70	0.33	0.55	0.39	0.29	0.65	0.23	0.31	0.21	0.29	0.35	0.40	0.39	0.50	0.18	1.00
**44P 0.54**	0.30	0.03	0.05	0.06	0.07	0.06	0.29	0.05	0.12	0.08	0.03	0.08	0.89	0.60	0.15	1.00	0.54	0.07	0.02	0.16
**45A 0.55**	0.74	0.02	0.02	0.02	0.01	1.00	0.01	0.01	0.02	0.02	0.08	0.02	0.08	0.02	0.02	0.88	0.36	0.13	0.00	0.04
**46V 0.55**	0.54	0.17	0.20	0.26	0.67	0.34	0.52	0.38	0.28	0.64	0.21	0.29	0.21	0.28	0.33	0.46	0.39	0.50	0.18	1.00
**47N 0.56**	0.81	0.07	0.58	0.89	0.06	0.40	0.12	0.19	0.83	0.29	0.11	0.51	0.30	0.53	0.47	1.00	0.96	0.33	0.02	0.05
**48Y 0.56**	0.29	0.07	0.10	0.14	0.30	0.15	0.27	0.16	0.12	0.27	0.09	0.13	0.18	0.12	0.14	0.34	0.19	0.21	0.08	1.00
**49A 0.56**	0.50	0.02	0.33	0.26	0.02	0.22	0.03	0.05	0.21	0.15	0.03	0.12	0.33	0.14	0.12	1.00	0.60	0.16	0.01	0.01
**50P 0.56**	0.62	0.21	1.00	0.94	0.04	0.35	0.10	0.12	0.58	0.19	0.07	0.54	0.52	0.38	0.33	0.79	0.87	0.19	0.02	0.04
**pos**	**A**	**C**	**D**	**E**	**F**	**G**	**H**	**I**	**K**	**L**	**M**	**N**	**P**	**Q**	**R**	**S**	**T**	**V**	**W**	**Y**
**51L 0.55**	0.63	0.06	0.02	0.07	0.06	0.02	0.12	0.59	0.04	0.40	0.12	0.03	0.10	0.03	0.03	0.23	0.12	1.00	0.01	0.07
**52D 0.55**	0.41	0.02	1.00	0.66	0.06	0.20	0.05	0.04	0.24	0.07	0.03	0.18	0.54	0.19	0.17	0.48	0.15	0.08	0.01	0.02
**53L 0.56**	1.00	0.13	0.14	0.18	0.51	0.44	0.30	0.24	0.19	0.64	0.17	0.19	0.21	0.18	0.21	0.49	0.32	0.70	0.10	0.55
**54P 0.57**	0.11	0.00	0.01	0.03	0.01	0.06	0.01	0.01	0.01	0.01	0.02	0.01	1.00	0.05	0.01	0.54	0.03	0.02	0.00	0.07
**55G 0.50**	0.25	0.00	0.14	0.03	0.00	1.00	0.00	0.00	0.01	0.01	0.03	0.01	0.01	0.01	0.01	0.41	0.12	0.04	0.00	0.00
**56S 0.50**	0.76	0.20	0.49	0.78	0.25	0.37	0.25	0.26	0.72	0.43	0.32	0.43	0.30	0.56	0.47	1.00	0.54	0.38	0.07	0.33
**57A 0.52**	1.00	0.01	0.00	0.05	0.00	0.92	0.00	0.00	0.01	0.01	0.00	0.00	0.12	0.00	0.00	0.58	0.01	0.01	0.00	0.00
**58E 0.57**	0.16	0.01	0.15	1.00	0.21	0.05	0.04	0.03	0.14	0.05	0.02	0.06	0.05	0.09	0.07	0.21	0.06	0.05	0.01	0.08
**59P 0.57**	0.66	0.01	0.08	0.01	0.00	0.24	0.00	0.01	0.01	0.02	0.01	0.01	1.00	0.01	0.01	0.44	0.29	0.03	0.00	0.00
**60P 0.58**	1.00	0.10	0.07	0.07	0.03	0.67	0.01	0.03	0.08	0.07	0.03	0.05	0.34	0.05	0.05	0.44	0.13	0.24	0.01	0.02
**61K 0.61**	0.02	0.00	0.00	0.03	0.00	0.00	0.00	0.00	1.00	0.00	0.00	0.01	0.07	0.04	0.20	0.02	0.00	0.00	0.00	0.00
**62Q 0.60**	0.01	0.00	0.01	0.09	0.00	0.00	0.00	0.00	0.02	0.01	0.00	0.01	0.10	1.00	0.01	0.04	0.11	0.00	0.00	0.00
**63C 0.60**	0.01	1.00	0.00	0.00	0.00	0.03	0.00	0.00	0.00	0.00	0.00	0.00	0.00	0.00	0.03	0.03	0.00	0.00	0.00	0.00
**64H 0.55**	0.59	0.02	0.27	0.43	0.03	0.24	0.31	0.18	0.47	0.13	0.05	0.39	0.26	0.30	0.23	1.00	0.38	0.13	0.01	0.02
**65P 0.54**	0.09	0.00	0.06	0.08	0.00	0.01	0.00	0.00	0.01	0.10	0.00	0.01	1.00	0.04	0.00	0.02	0.01	0.00	0.00	0.00
**66C 0.51**	0.10	1.00	0.00	0.00	0.00	0.03	0.00	0.00	0.00	0.00	0.00	0.00	0.15	0.00	0.00	0.02	0.01	0.03	0.00	0.00
**67P 0.55**	0.14	0.00	0.00	0.00	0.00	0.07	0.00	0.00	0.00	0.06	0.00	0.00	1.00	0.00	0.00	0.85	0.01	0.01	0.00	0.00
**68G 0.60**	1.00	0.04	0.02	0.03	0.02	0.40	0.03	0.02	0.03	0.07	0.01	0.02	0.57	0.02	0.02	0.14	0.11	0.06	0.01	0.02
**69V 0.56**	1.00	0.04	0.04	0.06	0.15	0.10	0.11	0.13	0.06	0.21	0.10	0.06	0.11	0.06	0.07	0.20	0.31	0.59	0.04	0.34
**70P 0.51**	0.57	0.05	0.10	0.16	0.06	0.13	0.09	0.14	0.33	0.15	0.06	0.15	1.00	0.40	0.30	0.45	0.61	0.76	0.02	0.07
**71Q 0.60**	0.29	0.03	0.23	0.14	0.19	0.15	0.14	0.05	0.13	0.08	0.03	0.25	0.19	1.00	0.09	0.36	0.22	0.07	0.02	0.10
**72G 0.61**	0.36	0.01	0.12	0.25	0.01	0.58	0.02	0.04	0.17	0.06	0.02	0.22	0.20	0.24	0.26	1.00	0.61	0.05	0.00	0.01
**73T 0.62**	0.17	0.00	0.00	0.00	0.00	0.05	0.00	0.00	0.00	0.00	0.00	0.00	0.07	0.00	0.00	1.00	0.18	0.00	0.00	0.03
**74S 0.62**	0.21	0.00	0.00	0.00	0.00	0.01	0.00	0.00	0.00	0.00	0.00	0.01	0.17	0.04	0.00	1.00	0.09	0.00	0.00	0.00
**75P 0.62**	0.58	0.05	0.28	0.33	0.04	0.34	0.07	0.10	0.39	0.14	0.06	0.40	0.45	0.23	0.23	1.00	0.93	0.16	0.11	0.05
**pos**	**A**	**C**	**D**	**E**	**F**	**G**	**H**	**I**	**K**	**L**	**M**	**N**	**P**	**Q**	**R**	**S**	**T**	**V**	**W**	**Y**
**76A 0.58**	1.00	0.00	0.00	0.04	0.00	0.08	0.00	0.00	0.00	0.00	0.00	0.00	0.03	0.00	0.00	0.11	0.15	0.00	0.03	0.00
**77P 0.62**	1.00	0.04	0.05	0.04	0.14	0.17	0.01	0.03	0.05	0.04	0.02	0.07	0.69	0.04	0.04	0.87	0.50	0.08	0.01	0.02
**78V 0.66**	0.00	0.00	0.00	0.00	0.04	0.00	0.00	0.03	0.00	1.00	0.10	0.00	0.00	0.00	0.00	0.00	0.00	0.25	0.00	0.00
**79P 0.66**	0.04	0.00	0.00	0.00	0.00	0.24	0.00	0.00	0.00	0.00	0.00	0.00	1.00	0.00	0.00	0.12	0.02	0.01	0.00	0.00
**80Y 0.67**	0.00	0.00	0.00	0.00	0.00	0.03	0.00	0.00	0.00	0.00	0.00	0.00	0.00	0.00	0.00	0.00	0.00	0.00	0.00	1.00
**81G 0.68**	0.00	0.00	0.01	0.00	0.00	1.00	0.00	0.00	0.00	0.00	0.00	0.02	0.00	0.00	0.00	0.26	0.00	0.00	0.00	0.00
**82Y 0.68**	0.00	0.00	0.00	0.00	0.00	0.00	0.08	0.00	0.00	0.00	0.00	0.00	0.00	0.00	0.00	0.00	0.00	0.00	0.03	1.00
**83F 0.67**	0.00	0.00	0.00	0.00	1.00	0.01	0.08	0.00	0.00	0.00	0.00	0.00	0.00	0.00	0.00	0.00	0.00	0.00	0.00	0.00
**84G 0.67**	0.01	0.00	0.00	0.00	0.00	1.00	0.00	0.00	0.00	0.00	0.00	0.00	0.09	0.00	0.04	0.00	0.00	0.00	0.00	0.00
**85G 0.68**	0.14	0.02	0.27	0.08	0.01	0.76	0.06	0.01	0.08	0.02	0.01	1.00	0.22	0.06	0.04	0.91	0.08	0.02	0.01	0.03
**86G 0.69**	0.02	0.00	0.02	0.01	0.00	1.00	0.12	0.00	0.01	0.00	0.00	0.02	0.09	0.01	0.04	0.32	0.01	0.00	0.00	0.00

**87Y 0.70**	0.00	0.00	0.00	0.00	0.03	0.00	0.00	0.08	0.00	0.00	0.00	0.00	0.00	0.00	0.00	0.00	0.00	0.00	0.00	1.00
**88Y 0.70**	0.00	0.00	0.00	0.00	0.09	0.00	0.00	0.00	0.00	0.00	0.00	0.00	0.00	0.00	0.00	0.00	0.09	0.00	0.00	1.00
**89S 0.71**	0.05	0.01	0.27	0.03	0.00	0.47	0.02	0.00	0.03	0.01	0.00	0.10	1.00	0.06	0.01	0.84	0.03	0.01	0.00	0.01
**90C 0.73**	0.09	1.00	0.00	0.00	0.00	0.00	0.00	0.00	0.00	0.00	0.00	0.00	0.00	0.00	0.00	0.01	0.00	0.03	0.00	0.00
**91R 0.73**	0.00	0.00	0.00	0.00	0.00	0.04	0.00	0.00	0.01	0.00	0.00	0.00	0.00	0.07	1.00	0.04	0.00	0.00	0.00	0.00
**92V 0.73**	0.00	0.00	0.00	0.00	0.01	0.00	0.00	0.08	0.00	0.37	1.00	0.00	0.00	0.00	0.00	0.00	0.00	0.62	0.00	0.14
**93S 0.74**	0.33	0.01	0.02	0.02	0.01	0.36	0.01	0.01	0.02	0.01	0.01	0.11	0.08	0.05	0.01	1.00	0.25	0.14	0.00	0.01
**94R 0.71**	0.04	0.01	0.03	0.04	0.01	0.04	1.00	0.01	0.16	0.03	0.01	0.08	0.05	0.10	0.96	0.22	0.22	0.02	0.01	0.01
**95S 0.70**	0.43	0.03	0.35	0.11	0.02	0.61	0.44	0.01	0.11	0.03	0.01	0.67	0.13	0.08	0.06	1.00	0.11	0.04	0.01	0.04
**96S 0.71**	0.52	0.02	0.05	0.03	0.01	0.12	0.01	0.01	0.03	0.02	0.01	0.26	0.16	0.26	0.02	1.00	0.12	0.03	0.14	0.01
**97L 0.71**	0.02	0.01	0.01	0.11	0.03	0.01	0.01	0.36	0.01	1.00	0.03	0.01	0.01	0.19	0.01	0.02	0.02	0.12	0.01	0.12
**98K 0.74**	0.00	0.00	0.00	0.09	0.00	0.00	0.00	0.00	1.00	0.00	0.00	0.00	0.00	0.01	0.15	0.00	0.00	0.00	0.00	0.00
**99P 0.75**	0.26	0.02	0.14	0.05	0.01	0.08	0.01	0.02	0.04	0.03	0.01	0.07	0.55	0.05	0.02	1.00	0.32	0.04	0.01	0.01
**100C 0.75**	0.07	1.00	0.00	0.00	0.00	0.14	0.00	0.00	0.02	0.10	0.00	0.00	0.03	0.00	0.00	0.15	0.01	0.01	0.00	0.00
**pos**	**A**	**C**	**D**	**E**	**F**	**G**	**H**	**I**	**K**	**L**	**M**	**N**	**P**	**Q**	**R**	**S**	**T**	**V**	**W**	**Y**
**101A 0.76**	1.00	0.02	0.03	0.02	0.01	0.05	0.03	0.02	0.03	0.17	0.01	0.05	0.54	0.02	0.02	0.57	0.80	0.04	0.01	0.01
**102Q 0.77**	0.25	0.03	0.13	0.07	0.06	0.31	0.26	0.04	0.08	0.07	0.02	0.16	0.12	1.00	0.06	0.29	0.08	0.07	0.02	0.36
**103A 0.77**	1.00	0.07	0.28	0.12	0.03	0.52	0.22	0.03	0.12	0.06	0.03	0.32	0.45	0.08	0.07	0.46	0.16	0.10	0.01	0.04
**104A 0.78**	1.00	0.01	0.01	0.01	0.00	0.07	0.00	0.00	0.01	0.01	0.00	0.01	0.41	0.01	0.01	0.93	0.07	0.19	0.00	0.02
**105T 0.78**	1.00	0.15	0.18	0.23	0.87	0.44	0.46	0.34	0.25	0.56	0.33	0.31	0.27	0.25	0.30	0.95	0.69	0.49	0.16	0.94
**106L 0.78**	0.50	0.16	0.24	0.35	0.88	0.34	0.50	0.36	0.27	0.94	0.20	0.29	0.32	0.28	0.32	0.40	0.36	0.46	0.17	1.00
**107A 0.78**	1.00	0.01	0.07	0.13	0.01	0.42	0.01	0.02	0.38	0.04	0.16	0.09	0.34	0.09	0.06	0.35	0.12	0.20	0.00	0.00
**108A 0.79**	0.41	0.02	0.59	1.00	0.02	0.18	0.05	0.06	0.41	0.10	0.03	0.17	0.10	0.24	0.16	0.51	0.20	0.09	0.01	0.28
**109Y 0.79**	0.31	0.05	0.08	0.16	0.26	0.17	0.17	0.11	1.00	0.20	0.07	0.14	0.25	0.29	0.41	0.26	0.18	0.16	0.05	0.38
**110P 0.79**	1.00	0.10	0.18	0.27	0.10	0.57	0.09	0.08	0.21	0.16	0.06	0.16	0.32	0.14	0.19	0.53	0.23	0.20	0.03	0.45
**111A 0.81**	0.70	0.09	0.02	0.16	0.04	0.00	0.01	0.34	0.05	0.32	1.00	0.02	0.21	0.04	0.03	0.05	0.46	0.84	0.01	0.01
**112E 0.81**	0.01	0.00	1.00	0.33	0.00	0.04	0.00	0.00	0.02	0.00	0.00	0.02	0.01	0.01	0.00	0.18	0.22	0.00	0.00	0.00
**113T 0.82**	0.21	0.10	0.09	0.08	0.01	0.16	0.01	0.01	0.08	0.02	0.01	0.04	0.10	0.04	0.04	0.37	1.00	0.03	0.00	0.03
**114P 0.83**	0.64	0.03	0.05	0.06	0.01	0.13	0.02	0.03	0.08	0.05	0.02	0.07	0.55	0.07	0.29	1.00	0.56	0.08	0.01	0.01
**115T 0.83**	0.62	0.05	0.06	0.10	0.20	0.14	0.28	0.12	0.08	0.42	0.06	0.09	0.11	0.08	0.10	0.25	0.17	1.00	0.05	0.41
**116A 0.84**	0.84	0.01	0.03	0.02	0.01	0.22	0.01	0.01	0.02	0.02	0.01	0.15	1.00	0.02	0.02	0.91	0.36	0.03	0.00	0.20
**117G 0.85**	0.38	0.00	0.01	0.00	0.00	1.00	0.00	0.00	0.00	0.00	0.00	0.13	0.06	0.12	0.00	0.38	0.05	0.00	0.00	0.00
**118E 0.85**	0.10	0.00	0.40	1.00	0.00	0.01	0.00	0.00	0.02	0.00	0.00	0.19	0.01	0.01	0.00	0.02	0.00	0.00	0.00	0.00
**119E 0.84**	0.01	0.00	0.68	1.00	0.00	0.00	0.00	0.00	0.01	0.00	0.00	0.00	0.02	0.03	0.11	0.12	0.00	0.00	0.00	0.00
**120Y 0.84**	0.02	0.01	0.01	0.01	1.00	0.06	0.16	0.02	0.01	0.40	0.01	0.01	0.01	0.01	0.01	0.03	0.02	0.31	0.01	0.96
**121P 0.84**	0.09	0.01	0.01	0.01	0.00	0.17	0.00	0.00	0.01	0.01	0.00	0.01	1.00	0.21	0.00	0.58	0.30	0.01	0.00	0.00
**122S 0.84**	0.09	0.00	0.01	0.00	0.00	0.15	0.00	0.00	0.08	0.00	0.00	0.14	0.06	0.00	0.00	1.00	0.09	0.00	0.00	0.00
**123R 0.84**	0.07	0.00	0.00	0.00	0.00	0.00	0.01	0.00	0.01	0.00	0.00	0.00	0.01	0.00	1.00	0.00	0.00	0.00	0.00	0.00
**124P 0.85**	1.00	0.01	0.06	0.04	0.01	0.07	0.04	0.01	0.12	0.03	0.01	0.17	0.42	0.05	0.19	0.11	0.17	0.03	0.00	0.02
**125T 0.85**	0.14	0.00	0.01	0.02	0.00	0.10	0.01	0.01	1.00	0.01	0.00	0.01	0.01	0.03	0.06	0.10	0.26	0.01	0.00	0.00
**pos**	**A**	**C**	**D**	**E**	**F**	**G**	**H**	**I**	**K**	**L**	**M**	**N**	**P**	**Q**	**R**	**S**	**T**	**V**	**W**	**Y**
**126E 0.85**	0.00	0.00	0.08	1.00	0.00	0.03	0.00	0.00	0.00	0.00	0.00	0.00	0.00	0.02	0.01	0.13	0.00	0.00	0.00	0.00
**127F 0.85**	0.00	0.09	0.00	0.00	1.00	0.00	0.00	0.00	0.00	0.16	0.00	0.00	0.00	0.00	0.00	0.01	0.00	0.14	0.05	0.12
**128A 0.86**	1.00	0.00	0.00	0.00	0.00	0.09	0.00	0.00	0.00	0.00	0.00	0.08	0.00	0.07	0.00	0.15	0.05	0.00	0.00	0.00
**129F 0.86**	0.00	0.00	0.00	0.00	1.00	0.00	0.00	0.00	0.00	0.02	0.04	0.00	0.00	0.00	0.00	0.00	0.00	0.00	0.00	0.00
**130Y 0.85**	0.00	0.00	0.00	0.00	0.00	0.00	0.00	0.00	0.00	0.04	0.00	0.06	0.00	0.00	0.00	0.00	0.00	0.00	0.00	1.00
**131P 0.86**	0.14	0.02	0.17	0.07	0.01	0.28	0.29	0.01	0.07	0.02	0.01	0.20	1.00	0.62	0.05	0.16	0.06	0.02	0.01	0.02
**132G 0.86**	0.01	0.00	0.00	0.00	0.00	1.00	0.00	0.00	0.00	0.00	0.00	0.00	0.00	0.00	0.00	0.27	0.02	0.00	0.00	0.00
**133Y 0.86**	0.02	0.01	0.10	0.01	0.22	0.03	0.02	0.02	0.09	0.10	0.01	0.02	0.05	0.01	0.02	0.02	0.02	0.02	0.01	1.00
**134P 0.86**	0.86	0.01	0.01	0.01	0.00	0.20	0.01	0.01	0.01	0.01	0.01	0.01	1.00	0.01	0.01	0.20	0.33	0.15	0.00	0.00
**135G 0.86**	0.25	0.00	0.01	0.01	0.00	1.00	0.00	0.00	0.01	0.00	0.00	0.15	0.01	0.01	0.16	0.78	0.13	0.01	0.00	0.00
**136T 0.86**	0.03	0.00	0.00	0.00	0.00	0.01	0.00	0.00	0.00	0.00	0.00	0.00	1.00	0.00	0.12	0.30	0.02	0.00	0.00	0.00

**137Y**	**0.86**	0.04	0.00	0.00	0.00	0.01	0.00	0.00	0.00	0.00	0.00	0.05	0.00	0.00	0.00	0.00	0.01	0.07	0.00	0.00	1.00
**138Q**	**0.86**	0.00	0.00	0.00	0.07	0.00	0.07	0.01	0.00	0.01	0.00	0.00	0.00	0.03	1.00	0.05	0.00	0.00	0.00	0.00	0.00
**139P**	**0.85**	0.43	0.02	0.03	0.03	0.01	0.06	0.25	0.03	0.05	0.03	0.02	0.05	1.00	0.03	0.19	0.45	0.31	0.26	0.01	0.01
**140M**	**0.85**	0.02	0.00	0.00	0.00	0.08	0.00	0.00	0.03	0.00	0.20	0.68	0.00	0.00	0.00	0.02	0.00	0.01	1.00	0.00	0.12
**141A**	**0.86**	0.50	0.00	0.00	0.00	0.00	0.08	0.00	0.00	0.00	0.00	0.00	0.00	1.00	0.00	0.00	0.02	0.02	0.00	0.00	0.00
**142S**	**0.86**	0.09	0.02	0.47	0.11	0.02	0.99	0.08	0.01	0.10	0.02	0.01	0.43	0.06	0.18	0.05	1.00	0.08	0.02	0.01	0.10
**143Y**	**0.86**	0.12	0.00	0.00	0.00	0.00	0.01	0.00	0.00	0.00	0.00	0.00	0.00	0.01	0.00	0.00	0.07	0.00	0.01	0.00	1.00
**144L**	**0.86**	0.00	0.04	0.00	0.00	0.09	0.00	0.00	0.14	0.00	1.00	0.15	0.00	0.00	0.00	0.00	0.00	0.00	0.00	0.00	0.00
**145D**	**0.86**	0.00	0.00	1.00	0.01	0.00	0.04	0.00	0.00	0.00	0.00	0.00	0.01	0.00	0.00	0.00	0.06	0.00	0.00	0.00	0.00
**146V**	**0.85**	0.10	0.01	0.00	0.01	0.00	0.01	0.00	0.02	0.01	0.02	0.52	0.00	0.01	0.10	0.00	0.10	0.02	1.00	0.00	0.00
**147S**	**0.74**	0.43	0.05	0.06	0.06	0.15	0.18	0.10	0.06	0.07	0.08	0.03	0.09	0.43	0.05	0.05	1.00	0.24	0.13	0.02	0.08
**148V**	**0.74**	0.09	0.00	0.00	0.00	0.00	0.00	0.00	0.01	0.00	0.10	0.02	0.05	0.00	0.00	0.00	0.02	0.01	1.00	0.00	0.00
**149V**	**0.85**	0.08	0.00	0.00	0.00	0.00	0.00	0.00	0.01	0.00	0.09	0.00	0.00	0.00	0.00	0.00	0.00	0.02	1.00	0.04	0.00
**150Q**	**0.85**	0.06	0.00	0.06	0.27	0.04	0.03	0.03	0.02	0.20	0.04	0.12	0.04	1.00	0.76	0.23	0.29	0.05	0.02	0.01	0.02
**pos**		**A**	**C**	**D**	**E**	**F**	**G**	**H**	**I**	**K**	**L**	**M**	**N**	**P**	**Q**	**R**	**S**	**T**	**V**	**W**	**Y**
**151T**	**0.85**	0.23	0.02	0.06	0.03	0.01	0.32	0.02	0.02	0.04	0.02	0.01	0.17	0.05	0.03	0.02	0.35	1.00	0.03	0.01	0.08
**152L**	**0.85**	0.02	0.00	0.00	0.01	0.21	0.01	0.01	0.52	0.01	1.00	0.08	0.01	0.01	0.01	0.01	0.23	0.01	0.06	0.07	0.14
**153G**	**0.85**	0.05	0.00	0.00	0.00	0.00	1.00	0.00	0.00	0.00	0.00	0.00	0.00	0.00	0.00	0.05	0.45	0.00	0.00	0.00	0.00
**154A**	**0.85**	1.00	0.02	0.01	0.17	0.00	0.96	0.00	0.00	0.01	0.01	0.00	0.01	0.04	0.00	0.00	0.67	0.29	0.01	0.00	0.00
**155P**	**0.61**	0.26	0.02	0.03	0.02	0.01	0.21	0.28	0.01	0.03	0.02	0.01	0.03	1.00	0.02	0.11	0.28	0.07	0.03	0.00	0.01
**156G**	**0.83**	0.13	0.01	0.00	0.01	0.00	1.00	0.00	0.00	0.01	0.01	0.00	0.00	0.26	0.00	0.00	0.15	0.02	0.04	0.00	0.00
**157E**	**0.84**	0.00	0.00	0.17	1.00	0.00	0.00	0.00	0.00	0.01	0.00	0.00	0.01	0.00	0.06	0.00	0.00	0.04	0.00	0.00	0.00
**158P**	**0.84**	0.14	0.00	0.00	0.08	0.09	0.11	0.00	0.00	0.00	0.00	0.00	0.00	1.00	0.00	0.00	0.13	0.02	0.01	0.00	0.00
**159R**	**0.86**	0.00	0.00	0.00	0.00	0.00	0.00	0.00	0.00	0.07	0.04	0.00	0.00	0.00	0.06	1.00	0.00	0.00	0.00	0.00	0.00
**160H**	**0.85**	0.00	0.00	0.08	0.00	0.00	0.05	1.00	0.00	0.00	0.00	0.00	0.01	0.01	0.00	0.00	0.07	0.00	0.00	0.00	0.01
**161D**	**0.86**	0.01	0.00	0.98	1.00	0.00	0.01	0.17	0.00	0.01	0.00	0.00	0.03	0.01	0.01	0.13	0.01	0.00	0.00	0.00	0.00
**162S**	**0.85**	0.58	0.01	0.03	0.02	0.01	0.03	0.08	0.02	0.03	0.02	0.01	0.05	0.34	0.14	0.02	0.65	1.00	0.03	0.00	0.01
**163L**	**0.85**	0.01	0.00	0.00	0.00	0.05	0.00	0.00	0.04	0.00	1.00	0.07	0.00	0.09	0.08	0.00	0.00	0.01	0.09	0.00	0.15
**164L**	**0.86**	0.00	0.00	0.00	0.00	0.01	0.00	0.00	0.30	0.00	1.00	0.01	0.00	0.01	0.08	0.08	0.00	0.00	0.01	0.00	0.00
**165P**	**0.85**	0.01	0.00	0.00	0.00	0.00	0.00	0.01	0.08	0.02	0.00	0.00	0.00	1.00	0.01	0.08	0.17	0.01	0.00	0.00	0.00
**166V**	**0.85**	0.02	0.00	0.00	0.00	0.01	0.00	0.00	0.17	0.00	0.04	1.00	0.00	0.00	0.11	0.00	0.00	0.08	0.77	0.00	0.10
**167D**	**0.86**	0.02	0.00	0.90	1.00	0.00	0.01	0.07	0.00	0.02	0.10	0.00	0.01	0.13	0.14	0.01	0.01	0.01	0.00	0.00	0.00
**168S**	**0.86**	0.08	0.01	0.12	0.04	0.01	0.82	0.03	0.01	0.05	0.02	0.01	0.24	0.08	0.13	0.03	1.00	0.29	0.03	0.01	0.02
**169Y**	**0.85**	0.00	0.00	0.00	0.01	0.00	0.00	0.02	0.00	0.06	0.00	0.00	0.00	0.07	0.07	0.02	0.00	0.00	0.00	0.00	1.00
**170Q**	**0.85**	0.00	0.00	0.01	0.08	0.00	0.00	0.04	0.00	0.00	0.00	0.00	0.00	0.05	1.00	0.00	0.00	0.00	0.00	0.00	0.00
**171S**	**0.85**	0.06	0.00	0.01	0.00	0.00	0.07	0.19	0.00	0.00	0.00	0.00	0.01	1.00	0.00	0.00	0.34	0.01	0.01	0.00	0.00
**172W**	**0.85**	0.01	0.06	0.01	0.08	0.00	0.01	0.00	0.00	0.09	0.00	0.00	0.01	0.00	0.01	0.02	0.01	0.01	0.00	1.00	0.04
**173A**	**0.85**	1.00	0.00	0.00	0.00	0.00	0.00	0.00	0.00	0.00	0.00	0.00	0.00	0.06	0.04	0.00	0.07	0.17	0.00	0.00	0.00
**174L**	**0.79**	0.01	0.00	0.00	0.00	0.00	0.00	0.00	0.22	0.00	1.00	0.01	0.00	0.04	0.00	0.00	0.06	0.17	0.01	0.00	0.00
**175A**	**0.77**	0.97	0.07	0.07	0.07	0.03	0.60	0.02	0.05	0.08	0.07	0.04	0.09	0.42	0.19	0.06	0.86	1.00	0.13	0.01	0.02
**pos**		**A**	**C**	**D**	**E**	**F**	**G**	**H**	**I**	**K**	**L**	**M**	**N**	**P**	**Q**	**R**	**S**	**T**	**V**	**W**	**Y**
**176G**	**0.80**	0.09	0.00	0.03	0.01	0.00	0.27	0.01	0.00	0.01	0.00	0.00	1.00	0.01	0.01	0.01	0.40	0.01	0.00	0.00	0.00
**177G**	**0.79**	0.09	0.00	0.00	0.00	0.00	1.00	0.00	0.00	0.00	0.00	0.00	0.00	0.08	0.03	0.00	0.20	0.00	0.00	0.00	0.00
**178W**	**0.80**	0.01	0.00	0.00	0.01	0.00	0.01	0.01	0.00	0.12	0.01	0.00	0.05	0.01	0.01	0.10	0.01	0.01	0.00	1.00	0.06
**179N**	**0.80**	0.02	0.00	0.47	0.02	0.00	0.33	0.14	0.00	0.02	0.07	0.00	1.00	0.08	0.01	0.01	0.22	0.02	0.00	0.00	0.01
**180S**	**0.80**	0.13	0.00	0.02	0.01	0.00	1.00	0.07	0.00	0.14	0.00	0.00	0.10	0.00	0.00	0.00	0.75	0.01	0.00	0.00	0.00
**181Q**	**0.80**	0.01	0.00	0.17	0.01	0.00	0.06	0.02	0.00	0.01	0.00	0.00	0.02	0.07	1.00	0.00	0.01	0.00	0.00	0.00	0.00
**182M**	**0.81**	0.24	0.01	0.12	0.01	0.12	0.00	0.01	0.16	0.01	0.19	0.71	0.01	0.02	0.07	0.01	0.01	0.05	1.00	0.00	0.01
**183C**	**0.81**	0.23	0.19	0.83	0.36	0.05	0.61	0.21	0.04	0.53	0.08	0.03	1.00	0.17	0.25	0.34	0.48	0.22	0.06	0.02	0.41
**184C**	**0.81**	0.16	1.00	0.00	0.00	0.00	0.05	0.00	0.00	0.00	0.00	0.06	0.00	0.00	0.00	0.00	0.01	0.04	0.00	0.00	0.00
**185Q**	**0.79**	1.00	0.02	0.13	0.34	0.01	0.08	0.02	0.03	0.09	0.04	0.02	0.18	0.61	0.37	0.05	0.90	0.32	0.05	0.01	0.01
**186G**	**0.79**	0.12	0.01	0.03	0.04	0.01	0.40	0.02	0.02	1.00	0.13	0.01	0.04	0.05	0.06	0.14	0.07	0.04	0.03	0.01	0.01

pos	A	C	D	E	F	G	H	I	K	L	M	N	P	Q	R	S	T	V	W	Y
187E 0.79	0.00	0.00	0.94	1.00	0.00	0.00	0.00	0.00	0.01	0.00	0.00	0.00	0.00	0.01	0.00	0.00	0.00	0.00	0.00	0.00
188Q 0.80	0.11	0.00	0.05	0.01	0.00	0.08	0.00	0.00	0.01	0.00	0.00	0.01	0.11	1.00	0.00	0.02	0.01	0.01	0.00	0.00
189N 0.80	0.71	0.03	0.58	0.69	0.03	0.72	0.08	0.10	0.51	0.16	0.05	1.00	0.36	0.31	0.27	0.90	0.68	0.15	0.01	0.03
190P 0.80	0.03	0.00	0.03	0.03	0.01	0.03	0.01	0.01	0.04	0.11	0.01	0.12	0.25	1.00	0.03	0.03	0.02	0.01	0.00	0.01
191P 0.80	0.55	0.01	0.00	0.00	0.00	0.20	0.02	0.00	0.01	0.03	0.00	0.01	1.00	0.00	0.00	0.72	0.07	0.01	0.00	0.00
192G 0.80	0.49	0.16	0.06	0.07	0.04	1.00	0.03	0.04	0.08	0.07	0.03	0.17	0.35	0.06	0.12	0.66	0.38	0.21	0.03	0.05
193P 0.75	0.24	0.05	0.06	0.07	0.20	0.18	1.00	0.10	0.08	0.16	0.06	0.10	0.28	0.08	0.09	0.21	0.11	0.13	0.05	0.33
194F 0.71	0.03	0.01	0.01	0.01	0.45	0.09	0.02	0.13	0.01	1.00	0.11	0.10	0.01	0.02	0.01	0.01	0.02	0.07	0.01	0.02
195W 0.70	0.00	0.00	0.00	0.00	0.00	0.08	0.00	0.00	0.00	0.08	0.00	0.00	0.00	0.00	0.00	0.00	0.00	0.00	1.00	0.00
196K 0.70	0.02	0.00	0.02	0.04	0.00	0.09	0.01	0.09	1.00	0.01	0.07	0.01	0.01	0.02	0.03	0.01	0.01	0.01	0.00	0.00
197A 0.72	0.19	0.01	0.02	0.12	0.09	0.13	0.11	0.01	0.01	0.01	0.00	0.02	0.02	0.01	0.01	1.00	0.16	0.01	0.00	0.01
198A 0.72	1.00	0.01	0.01	0.01	0.00	0.17	0.00	0.00	0.01	0.01	0.00	0.01	0.32	0.00	0.00	0.62	0.29	0.01	0.00	0.00
199F 0.72	0.03	0.01	0.08	0.01	1.00	0.14	0.02	0.14	0.01	0.73	0.03	0.01	0.01	0.01	0.02	0.02	0.02	0.07	0.01	0.09
200A 0.72	1.00	0.03	0.02	0.02	0.01	0.24	0.00	0.01	0.03	0.02	0.01	0.02	0.64	0.10	0.02	0.23	0.11	0.06	0.00	0.01
pos	A	C	D	E	F	G	H	I	K	L	M	N	P	Q	R	S	T	V	W	Y
201D 0.73	0.02	0.00	1.00	0.09	0.00	0.34	0.00	0.00	0.01	0.00	0.00	0.02	0.01	0.01	0.00	0.01	0.01	0.00	0.00	0.00
202S 0.72	0.74	0.05	0.70	1.00	0.05	0.34	0.14	0.17	0.81	0.27	0.10	0.45	0.27	0.53	0.44	0.84	0.69	0.52	0.02	0.05
203S 0.75	0.39	0.03	0.08	0.21	0.02	0.25	0.02	0.03	0.11	0.07	0.02	0.08	0.08	0.07	0.06	0.40	0.16	1.00	0.01	0.02
204G 0.74	1.00	0.01	0.01	0.02	0.00	0.17	0.00	0.00	0.16	0.01	0.00	0.01	0.24	0.01	0.04	0.49	0.08	0.13	0.00	0.00
205Q 0.49	0.56	0.13	0.23	0.43	0.44	0.36	0.43	0.30	1.00	0.60	0.18	0.35	0.27	0.55	0.87	0.46	0.43	0.40	0.13	0.64
206H 0.64	0.07	0.01	0.13	0.07	0.01	0.25	1.00	0.01	0.04	0.12	0.01	0.38	0.07	0.03	0.03	0.13	0.04	0.01	0.01	0.03
207P 0.74	1.00	0.09	0.08	0.10	0.04	0.57	0.02	0.05	0.13	0.13	0.06	0.07	0.48	0.39	0.10	0.50	0.19	0.16	0.01	0.03
208P 0.76	0.20	0.01	0.10	0.07	0.01	0.12	0.28	0.01	0.07	0.04	0.01	0.13	1.00	0.15	0.04	0.53	0.08	0.02	0.00	0.01
209D 0.76	0.00	0.00	1.00	0.36	0.00	0.00	0.00	0.00	0.00	0.00	0.00	0.00	0.00	0.01	0.00	0.10	0.00	0.06	0.00	0.00
210A 0.76	1.00	0.08	0.06	0.07	0.03	0.92	0.01	0.04	0.08	0.13	0.16	0.05	0.20	0.05	0.06	0.50	0.14	0.29	0.01	0.02
211C 0.77	0.86	0.96	0.05	0.05	0.02	0.50	0.01	0.02	0.05	0.13	0.02	0.34	0.13	0.04	0.04	1.00	0.10	0.08	0.01	0.02
212A 0.78	0.24	0.01	0.01	0.01	0.00	0.01	0.01	0.02	0.01	0.03	0.01	0.02	0.10	0.01	0.01	1.00	0.32	0.24	0.00	0.00
213F 0.78	0.00	0.00	0.00	0.00	1.00	0.00	0.00	0.00	0.00	0.03	0.00	0.00	0.00	0.00	0.00	0.00	0.00	0.21	0.01	0.98
214R 0.79	0.00	0.00	0.00	0.00	0.00	0.00	0.05	0.00	0.02	0.00	0.00	0.00	0.06	0.08	1.00	0.00	0.04	0.00	0.00	0.00
215R 0.79	0.00	0.00	0.00	0.00	0.00	0.00	0.00	0.00	0.00	0.00	0.00	0.00	0.00	0.00	1.00	0.07	0.00	0.00	0.00	0.00
216G 0.78	0.02	0.00	0.00	0.00	0.00	1.00	0.00	0.00	0.00	0.00	0.00	0.00	0.00	0.00	0.00	0.08	0.14	0.00	0.00	0.00
217R 0.82	0.00	0.00	0.00	0.00	0.00	0.00	0.00	0.00	0.00	0.00	0.00	0.00	0.00	0.00	1.00	0.00	0.00	0.00	0.00	0.00
218K 0.82	0.00	0.00	0.00	0.00	0.00	0.00	0.00	0.00	1.00	0.00	0.00	0.00	0.00	0.00	0.00	0.00	0.05	0.00	0.00	0.00
219K 0.82	0.00	0.00	0.00	0.00	0.00	0.00	0.00	0.00	1.00	0.00	0.00	0.00	0.00	0.00	0.00	0.00	0.00	0.00	0.00	0.00
220R 0.82	0.00	0.00	0.00	0.00	0.00	0.00	0.00	0.00	0.00	0.00	0.00	0.00	0.00	0.00	1.00	0.01	0.00	0.00	0.00	0.00
221I 0.82	0.04	0.23	0.00	0.01	0.00	0.00	0.00	0.58	0.07	0.03	0.01	0.00	0.01	0.02	0.11	0.01	0.03	1.00	0.00	0.00
222P 0.82	0.00	0.00	0.00	0.00	0.00	0.00	0.00	0.00	0.00	0.00	0.00	0.00	1.00	0.00	0.00	0.00	0.00	0.00	0.00	0.00
223Y 0.83	0.00	0.00	0.00	0.00	0.00	0.00	0.00	0.00	0.00	0.00	0.00	0.00	0.00	0.00	0.00	0.00	0.00	0.00	0.00	1.00
224S 0.83	0.00	0.00	0.00	0.00	0.00	0.00	0.00	0.00	0.00	0.00	0.00	0.00	0.00	0.00	0.00	0.39	1.00	0.00	0.00	0.00
225K 0.83	0.00	0.00	0.00	0.00	0.00	0.00	0.00	0.00	1.00	0.00	0.00	0.00	0.00	0.00	0.00	0.00	0.00	0.00	0.00	0.00
pos	A	C	D	E	F	G	H	I	K	L	M	N	P	Q	R	S	T	V	W	Y
226G 0.83	0.44	0.15	0.17	0.23	0.72	0.56	0.45	0.45	0.24	0.75	0.20	0.26	0.21	0.26	0.29	0.33	0.33	1.00	0.15	0.92
227Q 0.83	0.00	0.00	0.00	0.00	0.00	0.00	0.00	0.00	0.00	0.00	0.00	0.00	0.00	1.00	0.00	0.00	0.00	0.00	0.00	0.00
228L 0.83	0.00	0.00	0.00	0.00	0.00	0.00	0.00	0.14	0.00	1.00	0.00	0.00	0.00	0.00	0.00	0.00	0.13	0.00	0.00	0.00
229R 0.83	0.03	0.00	0.00	0.02	0.12	0.01	0.03	0.01	1.00	0.10	0.00	0.01	0.01	0.03	0.33	0.01	0.02	0.01	0.00	0.01
230E 0.83	0.00	0.00	0.01	1.00	0.00	0.00	0.00	0.00	0.00	0.01	0.00	0.00	0.00	0.01	0.00	0.00	0.00	0.00	0.00	0.00
231L 0.83	0.00	0.00	0.00	0.00	0.00	0.00	0.00	0.00	0.00	1.00	0.00	0.00	0.00	0.00	0.00	0.00	0.00	0.00	0.00	0.00
232E 0.83	0.00	0.00	0.00	1.00	0.00	0.00	0.00	0.00	0.00	0.00	0.00	0.00	0.00	0.00	0.00	0.00	0.00	0.00	0.00	0.00
233R 0.83	0.00	0.00	0.03	0.01	0.00	0.00	0.00	0.00	1.00	0.02	0.00	0.19	0.00	0.03	0.90	0.03	0.00	0.00	0.00	0.00
234E 0.83	0.00	0.00	0.00	1.00	0.00	0.00	0.00	0.00	0.00	0.00	0.00	0.00	0.00	0.00	0.00	0.00	0.00	0.00	0.00	0.00
235Y 0.83	0.00	0.00	0.00	0.00	0.28	0.00	0.00	0.00	0.00	0.00	0.00	0.00	0.00	0.00	0.00	0.00	0.00	0.00	0.00	1.00

**236A 0.83**	1.00	0.01	0.01	0.01	0.08	0.07	0.00	0.01	0.01	0.14	0.00	0.01	0.02	0.07	0.01	0.17	0.04	0.02	0.00	0.01
**237A 0.83**	1.00	0.13	0.14	0.19	0.70	0.23	0.37	0.48	0.21	0.58	0.16	0.21	0.15	0.22	0.24	0.48	0.66	0.52	0.13	0.74
**238N 0.83**	0.00	0.00	0.01	0.00	0.00	0.00	0.07	0.00	0.00	0.00	0.00	1.00	0.00	0.02	0.00	0.22	0.00	0.00	0.00	0.00
**239K 0.83**	0.01	0.00	0.00	0.01	0.00	0.00	0.00	0.00	1.00	0.01	0.12	0.00	0.00	0.07	0.03	0.00	0.01	0.08	0.00	0.00
**240F 0.83**	0.00	0.00	0.00	0.00	1.00	0.00	0.00	0.00	0.00	0.00	0.00	0.00	0.00	0.00	0.00	0.00	0.00	0.00	0.00	0.26
**241I 0.83**	0.00	0.00	0.00	0.00	0.00	0.00	0.00	1.00	0.00	0.19	0.00	0.00	0.00	0.00	0.00	0.00	0.00	0.03	0.00	0.00
**242T 0.83**	0.00	0.00	0.00	0.00	0.00	0.00	0.00	0.00	0.00	0.00	0.00	0.19	0.00	0.00	0.00	0.01	1.00	0.00	0.00	0.00
**243K 0.83**	0.00	0.00	0.00	0.00	0.00	0.00	0.00	0.00	1.00	0.00	0.00	0.00	0.00	0.00	0.28	0.00	0.00	0.00	0.00	0.00
**244D 0.83**	0.00	0.00	1.00	0.20	0.00	0.00	0.00	0.00	0.00	0.00	0.00	0.00	0.00	0.01	0.00	0.00	0.00	0.00	0.00	0.00
**245K 0.83**	0.00	0.00	0.00	0.00	0.00	0.00	0.00	0.00	1.00	0.00	0.00	0.00	0.00	0.00	0.20	0.00	0.00	0.00	0.00	0.00
**246R 0.83**	0.00	0.00	0.00	0.00	0.00	0.00	0.00	0.00	0.00	0.00	0.00	0.00	0.00	0.00	1.00	0.00	0.00	0.00	0.00	0.00
**247R 0.83**	0.07	0.00	0.00	0.02	0.00	0.00	0.01	0.00	0.06	0.08	0.00	0.01	0.00	0.01	1.00	0.05	0.01	0.00	0.00	0.08
**248K 0.82**	0.00	0.00	0.00	0.18	0.00	0.00	0.08	0.00	0.94	0.00	0.00	0.00	0.00	0.16	1.00	0.00	0.01	0.00	0.00	0.00
**249I 0.82**	0.00	0.00	0.00	0.00	0.00	0.00	0.00	1.00	0.00	0.13	0.00	0.00	0.00	0.00	0.00	0.00	0.00	0.08	0.00	0.00
**250S 0.82**	0.13	0.00	0.00	0.00	0.00	0.00	0.00	0.00	0.00	0.00	0.00	0.04	0.00	0.00	0.00	1.00	0.00	0.00	0.00	0.00

**pos**	**A**	**C**	**D**	**E**	**F**	**G**	**H**	**I**	**K**	**L**	**M**	**N**	**P**	**Q**	**R**	**S**	**T**	**V**	**W**	**Y**
**251A 0.81**	1.00	0.00	0.01	0.04	0.00	0.01	0.01	0.01	0.05	0.02	0.01	0.01	0.02	0.09	0.27	0.02	0.03	0.08	0.00	0.00
**252A 0.81**	1.00	0.01	0.06	0.05	0.03	0.06	0.10	0.03	0.05	0.18	0.03	0.04	0.02	0.13	0.04	0.23	0.43	0.42	0.01	0.05
**253T 0.81**	0.03	0.01	0.01	0.01	0.01	0.01	0.00	0.04	0.01	0.32	0.02	0.02	0.01	0.01	0.01	0.09	1.00	0.03	0.00	0.00
**254S 0.81**	0.00	0.00	0.01	0.00	0.00	0.02	0.00	0.00	0.00	0.00	0.00	1.00	0.00	0.00	0.00	0.33	0.08	0.00	0.00	0.00
**255L 0.81**	0.00	0.00	0.00	0.00	0.00	0.00	0.00	0.00	0.00	1.00	0.00	0.00	0.00	0.00	0.00	0.00	0.00	0.00	0.00	0.00
**256S 0.81**	0.00	0.00	0.00	0.00	0.00	0.00	0.00	0.00	0.00	0.00	0.00	0.00	0.00	0.00	0.00	1.00	0.40	0.00	0.00	0.00
**257E 0.81**	0.00	0.00	0.08	1.00	0.00	0.00	0.00	0.00	0.00	0.00	0.00	0.00	0.00	0.00	0.00	0.00	0.00	0.00	0.00	0.00
**258R 0.81**	0.00	0.00	0.00	0.00	0.00	0.00	0.00	0.00	0.00	0.00	0.00	0.00	0.00	0.00	1.00	0.00	0.00	0.00	0.00	0.00
**259Q 0.81**	0.00	0.00	0.00	0.00	0.00	0.00	0.00	0.00	0.00	0.00	0.00	0.00	0.00	1.00	0.00	0.00	0.00	0.00	0.00	0.00
**260I 0.81**	0.00	0.00	0.00	0.00	0.00	0.00	0.00	0.61	0.00	0.00	0.00	0.00	0.00	0.00	0.00	0.00	0.00	1.00	0.00	0.00
**261T 0.80**	0.01	0.00	0.00	0.00	0.00	0.00	0.00	0.00	0.29	0.00	0.02	0.00	0.00	0.00	0.01	0.01	1.00	0.00	0.00	0.00
**262I 0.80**	0.00	0.00	0.00	0.00	0.00	0.00	0.00	1.00	0.00	0.00	0.00	0.00	0.00	0.00	0.00	0.00	0.00	0.00	0.00	0.00
**263W 0.79**	0.00	0.00	0.00	0.00	0.00	0.00	0.00	0.00	0.00	0.00	0.00	0.00	0.00	0.00	0.00	0.00	0.00	0.00	1.00	0.00
**264F 0.79**	0.00	0.00	0.00	0.00	1.00	0.00	0.00	0.00	0.00	0.00	0.00	0.00	0.00	0.00	0.00	0.00	0.00	0.00	0.00	0.00
**265Q 0.77**	0.00	0.00	0.00	0.00	0.00	0.00	0.00	0.00	0.00	0.00	0.00	0.00	0.00	1.00	0.00	0.00	0.00	0.00	0.00	0.00
**266N 0.76**	0.00	0.00	0.00	0.00	0.00	0.00	0.00	0.00	0.00	0.00	0.00	1.00	0.00	0.00	0.00	0.00	0.00	0.00	0.00	0.00
**267R 0.76**	0.00	0.00	0.00	0.00	0.00	0.00	0.00	0.00	0.00	0.00	0.00	0.00	0.00	0.00	1.00	0.01	0.00	0.00	0.00	0.00
**268R 0.76**	0.00	0.00	0.00	0.00	0.00	0.00	0.00	0.00	0.00	0.00	0.00	0.00	0.00	0.00	1.00	0.01	0.00	0.00	0.00	0.00
**269V 0.66**	0.00	0.00	0.00	0.00	0.00	0.00	0.00	0.01	0.00	0.01	0.27	0.00	0.00	0.00	0.00	0.00	0.00	1.00	0.00	0.00
**270K 0.64**	0.00	0.00	0.00	0.00	0.00	0.00	0.00	0.00	1.00	0.00	0.00	0.00	0.00	0.00	0.00	0.00	0.00	0.00	0.00	0.00
**271E 0.64**	0.00	0.00	0.18	1.00	0.00	0.00	0.00	0.00	0.00	0.06	0.00	0.00	0.00	0.09	0.00	0.00	0.00	0.00	0.00	0.00
**272K 0.64**	0.00	0.00	0.00	0.00	0.00	0.00	0.00	0.00	1.00	0.00	0.00	0.00	0.00	0.00	0.00	0.00	0.00	0.00	0.00	0.00
**273K 0.64**	0.00	0.00	0.00	0.00	0.00	0.00	0.00	0.00	1.00	0.00	0.00	0.00	0.00	0.00	0.00	0.00	0.00	0.00	0.00	0.00
**274V 0.63**	0.01	0.00	0.00	0.00	0.32	0.00	0.00	0.32	0.00	0.46	0.10	0.00	0.00	0.00	0.00	0.00	0.00	1.00	0.00	0.00
**275L 0.62**	0.02	0.01	0.01	0.01	0.03	0.02	0.00	0.62	0.01	0.40	0.03	0.20	0.00	0.00	0.07	0.03	0.02	1.00	0.00	0.02

**pos**	**A**	**C**	**D**	**E**	**F**	**G**	**H**	**I**	**K**	**L**	**M**	**N**	**P**	**Q**	**R**	**S**	**T**	**V**	**W**	**Y**
**276A 0.62**	1.00	0.01	0.12	0.19	0.01	0.09	0.04	0.01	0.05	0.02	0.01	0.42	0.04	0.04	0.03	0.49	0.19	0.02	0.00	0.01
**277K 0.61**	0.00	0.00	0.00	0.00	0.00	0.00	0.00	0.00	1.00	0.00	0.00	0.00	0.00	0.00	0.06	0.00	0.00	0.00	0.00	0.00
**278V 0.61**	0.05	0.01	0.00	0.27	0.06	0.00	0.00	0.48	0.01	0.74	0.05	0.00	0.01	0.01	0.01	0.01	0.06	1.00	0.00	0.01
**279K 0.61**	0.00	0.00	0.00	0.05	0.00	0.00	0.00	0.00	1.00	0.00	0.00	0.00	0.00	0.00	0.00	0.00	0.00	0.00	0.00	0.00
**280N 0.54**	0.03	0.01	0.27	0.10	0.00	0.07	0.02	0.01	0.03	0.01	0.00	0.35	0.09	0.02	0.02	0.73	1.00	0.01	0.00	0.01
**281S 0.54**	0.04	0.00	0.01	0.00	0.00	0.01	0.00	0.07	0.01	0.01	0.00	0.33	0.03	0.01	0.00	1.00	0.51	0.03	0.00	0.00
**282A 0.53**	0.86	0.02	0.03	0.02	0.01	0.09	0.01	0.02	0.03	0.02	0.01	0.06	0.05	0.02	0.02	1.00	0.65	0.34	0.00	0.01
**283T 0.24**	0.01	0.00	0.01	0.00	0.00	0.00	0.00	0.06	0.01	0.00	0.00	0.07	0.01	0.00	0.00	0.50	1.00	0.01	0.00	0.00

284P 0.08 0.00 0.00 0.00 0.00 0.00 0.00 0.00 0.00 0.00 0.00 0.00 0.00 1.00 0.00 0.00 0.00 0.00 0.00 0.00 0.00
