# Supplementary material for: Computational Modeling of complete HOXB13 protein for predicting the functional effect of SNPs and the associated role in hereditary prostate cancer
Source: Sci Rep. 2017 Mar 8;7:43830. doi: 10.1038/srep43830 (PMC5363706; doi:10.1038/srep43830)
Supplement: Supplementary Dataset [file srep43830-s1.zip › SR Supplementary Data Files Complete/SIFT/SIFT_Alignment.pdf]

Alignment

```
                1                                                  50
       QUERY    MEPGNYATLD GAKDIEGLLG AGGGRNLVAH SPLTSHPAAP TLMPAVNYAP
 gi685597136    MEPGNYATLD GAKDIEGLLG AGGGRNLVAH SPLTSHPAAP TLMPAVNYAP
  gi60653709    MEPGNYATLD GAKDIEGLLG AGGGRNLVAH SPLTSHPAAP TLMPAVNYAP
 gi795250298    MEPGNYATLD GAKDIEGLLG AGGGRNLVAH SPLTSHPAAP TLMPAVNYAP
 gi635093043    MEPGNYATLD GAKDIEGLLG AGGGRNLVAH SPLTSHPAAP TLMPAVNYAP
 gi109114206    MEPGNYATLD GAKDIEGLLG AGGGRNLVAH SPLTSHPAAP TLMPAVNYAP
 gi795583451    MESGNYATLD GAKDIEGLLG AGGGRNLVAH SPLTSHPAAP TLMPAVNYAP
 gi559812841    MEPGNYATLD GAKDIEGLLG AGGGRNLVAH SPLTSHPAAP TLMPAVNYAP
 gi332259422    MEPGNYATLD GAKDIEGLLG AGGGRNLVAH SPLTSHQAAP TLMPAVNYAP
 gi795525940    MEPGNYATLD GAKDIEGLLG AGGGRNLVAH SPLTSHPAAP TLMPAVNYAP
 gi426237811    MEPGNYTTLD GAKEIEGLLG AGGSRNLVTH SPLTSHPAAP TLLPAVNYGP
 gi744588246    MEPGNYATLD GAKEIEGLLG TGASRNLVVH SPLTSHPAAP TLMPAVNYAP
 gi617633383    MEPGNYATLD GAKDLEGLLG TGGTRNLVAH SPASSHQAAP PLMPTVSYTP
 gi213511801    XXXXXXXXXE LNKNMEGLVG ANQCRNLMAH SALGGHPSS- -LVHSSGYSA
 gi444517714    MEPGNYATLD GAKDMEGLLG AGGGRNLVAH SPLTSHPAAP SLMPAVNYAP
 gi640808225    MEPGNYATLD GAKDMEGLLG AGGGRNLVAH SPLASHPAAP TLMSAVNYAP
 gi556958424    XXXXXENNLD ENKNMEGLMG ANQCRNLMAH SALGSHPSTL VHGSGYPTVE
 gi667260003    MEPGNYATLD GAKDIEGLLG AGGGRNLAAH SPLTSHPAAP TLMPAVNYAP
 gi831328207    XXXXXXXXXE LNKNMEGLVG ANQCRNLMAH SALGGHPSSL VHSSSYSGVD
 gi642105600    XXXXXXXXXE LNKNMEGLVG ANQCRNLMAH SALGGHPSS- -LVHSSGYSA
 gi471380475    MEPGNYATLD GAKDIEGLLG TGGGRNLVAH SPLTSHSAAP SLMPAANYAP
 gi674059771    MEHGNYATLD GAKDIEGLLG AGGGRNLVAH SPLASHPAAP TLMPAVNYAP
 gi344285419    MEPGNYATLD GAKDIEGLLG TGGGRNLVAH SPLTSHSAAP TLMPAANYAP
 gi742113362    XXXXXXXXXE LNKNMEGLVG ANQCRNLMAH SALGGHPSSL VHSSGYSSVD
 gi852732630    MEPGNYATLD GTKDIEGLLG AGGGRNLVAH SPLSSHPAAP TLMPAVNYAP
 gi512996173    MEPGSYATLD GAKDMEGLLG AGGGRNLVAH SPLSSHPAAP TLMPAVNYAP
 gi395826626    MEPGNYATLD GAKDMEGLLG AGGSRNLVAH SPLTSHPATP TLMPAVNYAP
 gi126308251    MESGAYATLD AGKDIEGLLG AGGCRNLVSH SPLASHPSAP ALLPTANYTP
 gi528767552    MEPGNYATLD GAKEIEGLLG TGASRNLVVH SPLTSHPAAP TLMPAVNYAP
 gi532208489    MEPGNYATLD GAKDMESLLG AGGGRNLVAH SPLTSHPAAP TLMPAVNYAP
 gi507654822    MEPGNYATLD GPKDMEGLLG AGGARNLVAH SPLASHPAAP TLMPAVNYAP
 gi488527225    MEPGNYATLD GAKDIEGLLG AGGGRNLVAH SPLTSHPAAP TLMPAASYAP
 gi817274366    MEPGNYATLD GPKDIEGLLG AGGGRNLVAH SPLTSHPAAA TLMPAVNYAP
 gi585671866    MEPGNYATLD VAKDIEGLLG TGGGRSLVAH SPLTSHPAAS ALMPAANYAP
 gi573901311    XXXXXXXXXE LNKNMEGLVG AGQCRNLMAH SALGGHPSTL VHGSGYSAVD
 gi532071900    MEPGNYATLD GAKDIEGLLG AGGGRNLVAH SPLPSHPAAP TLMPAVNYGP
 gi114149320    XXXXXENNLD ELKNMEGLVG ANQCRNLMAH SALSGHPSS- -LVHGSSYPT
 gi593727116    MEPGNYATLD GAKEIEGLLG AGGSRNLVAH SPLTSHPAAP TLMPAVNYAP
 gi470606860    MEPGNYATLD GAKEIEGLLG AGGSRNLVAH SPLTSHPAAP TLMPAVNYAP
 gi291405863    MESGNYATLD GAKDIEGLLG AGGGRNLVAH SPLSSHPAAP ALMPAVNYAP
 gi594656397    MEPGNYATLD GAKEIEGLLG AGGSRNLVAH SPLTSHPAAP TLMPAVNYAP
 gi634851243    MDPGNYATLD VAKDIEGLLA TGGSRHLVAH SPLTSHPAAP TLMPAANYAP
 gi602709820    MEPGNYATLD GAKEIEGLLG AGGSRNLVAH SPLTSHPAAP TLMPAVNYAP
 gi826271801    MELGNYATLD GAKDMEGLLG AGGGRNLVAH SPLTSHPATP TLMPAVNYAP
 gi589917310    MEPGNYATLD GAKDIEGLLG AGGGRNLVPH SPLANHPAAP TLMPAVNYAP
 gi507543189    MEPGTYATLD GAKDIEGLLG TGGGRNLAAH SPLASHPAAP TLMPAVNYAP
 gi395532657    MESGAYATLD AGKDIEGLLG AGGCRNLVSH SPLASHPSAS ALLPTPNYTS
 gi597740267    XXXXXXXXXE LNKNMEGLVG ANQCRNLMAH SALSAHPSSL VHGSSYSTVE
 gi725576032    MEPGNYATLD GAKDIEGLLG AGGGRNLVAH SPLTSHPAAP TLMPAVNYAP
 gi742135928    MEPGNYTTLD GAKEIEGLLG AGGSRNLVTH SPLTSHPSAP TLMPAVNYAP
 gi348562271    MEPGNYATLD GTKEMEGLLG AGGARNLVAH SPLASHPAAP TLMPAVNYAP
 gi594050844    MEPGNYTTLD GAKEIEGLLG AGGSRNLVTH SPLTSHPAAP TLMPAVNYAP
 gi533160875    MEPGNYATLD GAKDMEGLLG AGGARNLVAH SPLASHPAAP TLMPAVNYAP
 gi556738344    MEPGNYTTLD GAKEIEGLLG AGGSRNLVTH SPLTSHPAAP TLLPAVNYAP
 gi803246204    MEPGNYTTLD GAKEIEGLLG AGGSRNLVTH SPLTSHPAAP TLLPAVNYGP
 gi354474760    MEHGNYATLD GAKDIEGLLG AGGGRNLVAH SPLASHSATP TLMPTVNYAP
 gi532051321    MEPGNYATLD GAKDIEGLLG PGGSRNLVAH SPLASHPAAP TLMPTVNYAP
 gi311267502    MEPGNYATLD GAKEIEGLLG AGGSRNLVAH SPLTSHPAAP TLMPAVNYAP
 gi743729506    MEPGNYATLD GAKEIEGLLG TGASRNLVVH SPLTSHPAAP TLMPAVNYAP
 gi359754103    MESGAYATLD AGKDIEGLLG AGGCRNLVSH SPLASHPSAP ALLPTPNYTP
 gi576886849    XXXXXXXXXE LNKNMEGLVG ANQCRNLIAH SALSGHPSSL VHGSGYSTVD
 gi586448761    MEPGNYATLD MAKDMEGLLG TGGGRNLVTH SPLTSHPAAP TLMPAANYAP
 gi504159820    MEPGNYATLD GAKDIEGLLG AGGGRNLVSH SPLSSHPAAP ALVAAANYAP
```

```
  gi524951862  MEPGNYATLD GAKDIEGLLG AG--RNLVAH SPLASHPAAP TLMPTVNYAP
  gi332692483  XXXXXXXXXE LNKNMEGLVG ASQCRNLMAH SALGGHPSS- -LVHSYTAGD
  gi385654474  XXXXXXXXXE LNKNMEGLVG ASQCRNLMAH SALGGHPSS- -LVHSYTAGD
  gi478520959  MEPGNYATLD GAKDIEGLLG AGGSRNLVAH PSLTSHPAAP TLMPAVNYAP
  gi301128881  XXXXXXXXXX XXXXMEGFVG AGQCRNLMAH SALGAHPSP- -LVHGSGYST
 gi1011609041  MDPGTFTSHD SNKSMEGLMG AGQCRNLVAH SALGG-PSPP TLMHSSNYPT
  gi664747354  MEPGNYATLD GAKDIEGLLG AGGSRNLVAH SPLTSHPAAP TLMPAVNYAP
  gi557280818  MDPGTFTSHD SNKSMEGLMG AGQCRNLVAH SALGG-PSPS TLMHSSNYPT
  gi359076815  MEPSNYTTLD GAKEIEGLLG AGGSRNLVTH SPLTSHPSAP TLMPAVNYAP
  gi632972216  XXXXXXAVME GFVNT-GNFS ANQCRNLMAH SALGGHPAP- -LVHGSGYST
  gi759185571  MEPGSYATLD GAKDIEGLLG VGASRNLVAH SPLTSLPAAP TLMPAVNYAP
  gi472350967  MEPGSYAGLD GAKDMEGLPG AEGSRNLVAH SPPTSHPAAP TLMPAVSYAP
  gi586539979  MEPGSYATLD GAKDIEGLLG VGASRNLVAH SPLTSLPAAP TLMPAVNYAP
  gi157823841  MEPGNYATLD GAKDIEGLLG AGGGRNLVAH SPLASHPAAP TLMPTVNYAP
  gi410980861  MEPGNYATLD GAKDMDGLPG AGGSRNLVAH SPLTSHPAAP TLMPAVSYAP
  gi829772881  MELGNYATLD GAKDMEGLLG AGGGRNLVAH SPLTSHPATP TLMPAVNYAP
  gi220898176  XXXXXXXXXE VNKNMEGFPG GNFAANQCRN --LMAHPASL APSTAYTSSE
    gi1519038  MEPGNYATLD GAKDIEGLLG AGGGRNLVSH SPLASHPAAP TLMPTVNYAP
  gi731207261  MEPGGYATLD GAKDMEGLLG AGGGRNLLAH SPLSSHPAAP ALMPAVNYAP
   gi20278975  XXXXXXXXXE INKNMDGFSG GNFTANQCRN --LMAHPASL APSSAYTSSE
  gi585176637  MEPGSYAALD GAKDLEVLPG AGGSRHLVAH CPPTGHPAAP TLMPAVSHAP
  gi560984071  MEPGSYATLD GVKEIEGLLG TGASRNLVVH SPLTSHPAAP TLMPAVNYAP
   gi73966255  MEPGDYATLD GAKDLEGLSV VGGGRNLVAP SPLATHAAAP PLMPAVSYAP
  gi511890491  MEPGDYATLD GAKAMEGLPG AGGSRNLAAR SPLPSHPAAA GAPPAVSYAP
  gi254212171  XXXXXXXXXE INKSMDGFSG GNFTANQCRN --LMAHPAS- -LAPSSAYSS
  gi558229251  XXXXXXXXXE INKNMDGFHA GNQCRNLMAH PASLAAPSTA A----YTSSE
  gi752394348  MEPGDYATLD GAKDLEGLPA AGGGRHLAAH SPLTSHPAAP ALMPAVSYAP
  gi507952779  MEPGSFATLE GAKDVDGLLG AGGSRNLAAH SPLISHPAAP TLMPTVNYAP
  gi507664676  MEPGNYATLD LAKDMEGLLG TGGARHLAPH YSLTSHPAAP TLMPAANYAP
  gi224045310  XXXXXXXXXE INKNMDGFAG SNFAANPCRN LMAHPAPLAA PSAAAYTSSE
  gi400180333  XXXXXXXXXE VSKNMEGFAG GNFAANQCRN --LMAHPASL APSTAYTSSE
  gi641724374  MEPGNYAAAD GAKDLEGLLG AGGSRNLLAH SPLASHPAAP MLMPAVNYAP
  gi693302455  XXXXXXXXXE MNKNLEGFAG SNFAGNQCRN --LMAHPASL APSASYPAGD
  gi542179924  XXXXXXXXXD LNKNMEGLVA ASQCRNIMAH SALGGHPSSL -VHSSGGYST
  gi119370791  XXXXXXXXXD LNKNMEGLVG SGQCRNIMAH SALGGHPSGL -VHSSTGYST
  gi678126544  XXXXXXXXXE VNKNMDGFAG GNFAANQCRN --LMGHPASL APSSAYSSAD
  gi808884419  XXXXXXXXXD LNKNMEGLVG SGQCRNIMAH SALGGHPSGL -VHSSAGYST
  gi505802773  MEPGPYAALD GAKDVDGLLG AAAGRNLVAH SPLASPPAAP SLMPAVNYGP
   gi34484442  XXXXXXXXXD LNKNMEGLVG SGQCRNIMAH SALGGHPSGL -VHSSAGYST
  gi519777295  XXXXXXXXXE INKNMDGFPG ANQCRNLMAH PAPLGPPGAA YSAPGGS---
  gi657803392  XXXXXXXXXD LNKNMEGLVG ASQCRNIMAH SALGGHPSGL -MHSSAGYST
  gi554874539  XXXXXXXXXE LNKNMEGLVG SGQCRNIMAH SALGGHPSSL -VHSSGGYST
  gi499036069  XXXXXXXXXE LNKNMEGLVG SGQCRNIMAH SALGGHPSSL -VHSSGGYST
  gi301607689  XXXXXXXXXE MNKNMDGFPA ANQCRNLIGH HAP------- -LAPSSAYPP
  gi584023332  XXXXXXXXXE LNKNMEGLIG SGQCRNIMAH SALGGHPSSL -VHSSGGYST
  gi548549273  XXXXXXXXXE LNKNMEGLVG SGQCRNIMAH SALGGHPSSL -VHSSGGYSS
  gi657574876  XXXXXXXXXD LNKNMEGLVG SGQCRNIMAH SALGGHPSGL -VHSSAGYST
   gi28268588  XXXXXXXXXE MNKNMDGFPA ANQCRNLIGH HAP------- -LPPSSAYPS
  gi736251858  XXXXXXXXXD LNKNMEGLVG ASQCRNIMAH SALGGHPSGL -VHSSTGYPT
  gi530624973  MDPGTFASAE STKSMEGLMG AGQCRNLVGH SALGG-PPPP ALMQGSTYST
  gi290760363  MEAGFHAAAA AAAAGTNFVA ANQCRNLMVH PASLAGAGSA AAAAAAAYPS
  gi551520979  XXXXXXXXXD LNKNMEGLVG SGQCRNLMAH SAAAAAFGGH AVHSSAGYST
  gi658861163  XXXXXXXXXD LNKNMEGLVG SGQCRNLMAH SAAAAAFGGH AVHSSAGYST
  gi617509429  XXXXXXXXXD LNKNMEGVVG SGQCRNLMAH SAAAAAFGGH AVHSSAGYST
   gi47229429  XXXXXXXXXE VSKNMEGFAG GNFAANQCRN --LMGHPAS- -LAPSTAYSS
  gi831555258  XXXXXXXXXD ASKNMEGFPG GNFAANQCRN LMAHPASLAP SAAAAYSSGD
  gi591374792  MDPGTFASAE ELDEMEGLMG AGGCRNLVAH SAVGG-PPPP AFVQGSNYPT
  gi432871359  XXXXXXXXXD MSKNMDGLVG ASQCRNIMAH SAAAAALGGH AMHSSAGFST
  gi602643660  XXXXXXXXXX XXXXXXXXXX XXXXXXXXXX XXXXXXXXXX XXXXXXXYPS
  gi961915500  XXXXXXXXXE RSKNMEGFTG GNFAANQCRN --LLAHPTSL APSTTYAASD
  gi961860111  XXXXXXXXXE RSKNMEGFTG GNFAANQCRN --LLAHPTSL APSTTYAASD
  gi219879168  XXXXXXXXXD MSKNMEGFVG GNFAANQCRN --LIAHPSSL APSTTYTSSE
  gi219879156  XXXXXXXXXD MSKNMEGFVG GNFAANQCRN --LIAHPSSL APSTTYTSSE
  gi219879164  XXXXXXXXXD MSKNMEGFVG GNFAANQCRN --LIAHPSS- -LAPSTTYTS
  gi123204435  XXXXXXXXXD MSKNMEGFVG GNFAANQCRN --LIAHPSS- -LAPSTTYSS
  gi465985114  XXXXXXXXXX XXXXXXXXXX XXXXXXXXXX XXXXXXXXXX XXXXXXXXXX
  gi426355784  XXXXXXXXXX XXXXXXXXXX XXXXXXXXXX XXXXXXXXXX XXXXXXXXXX
 gi1034052196  XXXXXXXXXX XXXXXXXXXX XNQCRNLMAH PAPLA-PGAA SAYSSAGGPG
  gi683901043  XXXXXXXXXX XXXXXXXXXX XXXXXXXXXX XXXXXXXXXX XXXXXXXXXX
```

gi729725749	XXXXXXXXXX	XXXXXXXXXX	XXXXXXXXXX	XXXXXXXXXX	XXXXXXXXXX
gi663243102	XXXXXXXXXX	XXXXXXXXXX	XXXXXXXXXX	XXXXXXXXXX	XXXXXXXXXX
gi339792792	XXXXXXXXXX	XXXXXXXXXG	SNFAANQCRN	--LMAHPAPL	APGAAYSSAP
gi768393729	XXXXXXXXXX	XXXXXXXXXX	XXXXXXXXXX	XXXXXXXXXX	XXXXXXXXXX
gi670977729	XXXXXXXXXX	XXXXXXXXXX	XXXXXXXXXX	XXXXXXXXXX	XXXXXXXXXX
gi327263844	XXXXXXXXXX	XAAAMEGLAG	ASHCRDLIAH	PALGRHSSGL	GGHQGSVYAE
gi1040268871	XXPGKAPSMD	G---LGGSCP	ASHCRDLLPH	PVLGRPPAPL	GAPQGAVYTD
gi303303637	XXPGKAPSMD	G---LGNSCP	ASHCRDLLPH	PVLGRPPAPL	GAPQGAVYTD
gi724877051	XXPGKAPSMD	G---LGSSCP	ASHCRDLLPH	PVLGRPPAPL	GAPQGAVYTD
gi544470246	XXPGKAPSMD	G---LGSSCP	ASHCRDLLPH	PVLGRPPAPL	GAPQGAVYTD
gi338726307	XXPGKAPSMD	G---LGSSCP	ASHCRDLLPH	PVLGRPPAPL	GAPQGSVYTD
gi297692036	XXPGKAPSMD	G---LGSSCP	ASHCRDLLPH	PVLGRPPAPL	GAPQGAVYTD
gi397521957	XXPGKAPSMD	G---LGSSCP	ASHCRDLLPH	PVLGRPPAPL	GAPQGAVYTD
gi1018974175	XXXXXXXXXX	XXXXXXXXXX	XXXXXXXXXX	XXXXXXXXXX	XXXXXXXXXX
gi675430530	XXXXXXXXXX	XXXXXXXXXX	XXXXXXXXXX	XXXXXXXXXX	XXXXXXXXXX
gi795174618	XXXXXXXXXX	XXXXXXXXXX	XXXXXXXXXX	XXXXXXXXXX	XXXXXXXXXX
gi699633156	XXXXXXXXXX	XXXXXXXXXX	XXXXXXXXXX	XXXXXXXXXX	XXXXXXXXXX
gi374498883	XXXXXXXXXX	XXXXXXXXXX	XXXXXXXXXX	XXXXXXXXXX	XXXXXXXXXX
gi24637241	XXXXXXXXXX	XXXXMEGLCP	SNHCRDFMAH	PALGRGPQGP	------VYTD
gi584059997	XXXXXXXXXX	XXXXXXXXXX	XXXXXXXXXX	XXXXXXXXXX	XXXXXXXXXX
gi884764069	XXXXXXXXXX	XXXXXXXXXX	XXXXXXXXXX	XXXXXXXXXX	XXXXXXXXXX
gi66773958	XXXXXXXXXX	XXXXXXXXXX	XXXXXXXXXX	XXXXXXXXXX	XXXXXXXXXX
gi224016307	XXXXXXXXXX	XXXXXXXXXX	XXXXXXXXXX	XXXXXXXXXX	XXXXXXXXXX
gi224016295	XXXXXXXXXX	XXXXXXXXXX	XXXXXXXXXX	XXXXXXXXXX	XXXXXXXXXX
gi224016303	XXXXXXXXXX	XXXXXXXXXX	XXXXXXXXXX	XXXXXXXXXX	XXXXXXXXXX
gi224016309	XXXXXXXXXX	XXXXXXXXXX	XXXXXXXXXX	XXXXXXXXXX	XXXXXXXXXX
gi374498861	XXXXXXXXXX	XXXXXXXXXX	XXXXXXXXXX	XXXXXXXXXX	XXXXXXXXXX
gi224016313	XXXXXXXXXX	XXXXXXXXXX	XXXXXXXXXX	XXXXXXXXXX	XXXXXXXXXX
gi224016305	XXXXXXXXXX	XXXXXXXXXX	XXXXXXXXXX	XXXXXXXXXX	XXXXXXXXXX
gi224016311	XXXXXXXXXX	XXXXXXXXXX	XXXXXXXXXX	XXXXXXXXXX	XXXXXXXXXX
gi224016299	XXXXXXXXXX	XXXXXXXXXX	XXXXXXXXXX	XXXXXXXXXX	XXXXXXXXXX
gi700384804	XXXXXXXXXX	XXXXXXXXXX	XXXXXXXXXX	XXXXXXXXXX	XXXXXXXXXX
gi543378707	XXXXXXXXXX	XXXXXXXXXX	XXXXXXXXXX	XXXXXXXXXX	XXXXXXXXXX
gi699659589	XXXXXXXXXX	XXXXXXXXXX	XXXXXXXXXX	XXXXXXXXXX	XXXXXXXXXX
gi310656724	XXXXXXXXXX	XXXXMEGLRG	EGSPGHQCRN	LLASS-----	----------
gi565321354	XXXXXXXXXX	XXXXXXXXXX	XXXXXXXXXX	XXXXXXXXXX	XXXXXXXXXX
gi290760375	XXXXXXXXXX	XXXXXXXXXX	XXXXXXXXXX	XXXXXXXXXX	XXXXXXXXXX
gi696966138	XXXXXXXXXX	XXXXXXXXXX	XXXXXXXXXX	XXXXXXXXXX	XXXXXXXXXX
gi677975869	XXXXXXXXXX	XXXXXXXXXX	XXXXXXXXXX	XXXXXXXXXX	XXXXXXXXXX
gi543746989	XXXXXXXXXX	XXXXXXXXXX	XXXXXXXXXX	XXXXXXXXXX	XXXXXXXXXX
gi701389587	XXXXXXXXXX	XXXXXXXXXX	XXXXXXXXXX	XXXXXXXXXX	XXXXXXXXXX
gi256014533	XENPNENNQT	KIPAMEGLSG	TSHCRDLISH	PVLSRHSSTI	GTHQGSVYTE
gi669281149	XXXXXXXXXX	XXXXXXXXXX	XXXXXXXXXX	XXXXXXXXXX	XXXXXXXXXX
gi118102888	XXXXXXXXXX	XXXXXXXXXX	XXXXXXXXXX	XXXXXXXXXX	XXXXXXXXXX
gi554547429	MEPGNYAALD	GAKDVEGLLG	AGGGRRLLPH	PPLFAYPGYP	APYPMASYLD
gi694854743	XXXXXXXXXX	XXXXXXXXXX	XXXXXXXXXX	XXXXXXXXXX	XXXXXXXXXX
gi529426764	XXXXXXXXXX	XXXXXXXXXX	XXXXXXXXXX	XXXXXXXXXX	XXXXXXXXXX
gi706150961	XXXXXXXXXX	XXXXXXXXXX	XXXXXXXXXX	XXXXXXXXXX	XXXXXXXXXX
gi719780455	XXXXXXXXXX	XXXXXXXXXX	XXXXXXXXXX	XXXXXXXXXX	XXXXXXXXXX
gi290760370	XXXXXXXXXX	XXXXXXXXXX	XXXXXXXXXX	XXXXXXXXXX	XXXXXXXXXX
gi328682541	XXXXXXXXXX	XXXXMEGLKG	DGLSSTHYRN	SMHCSRSAPA	FVCPVSEHTN
gi541961056	XXXXXXXXXX	XXXXXXXXXX	XXXXXXXXXX	XXXXXXXXXX	XXXXXXXXXX
gi733928584	XXXXXXXXXX	XXXXXXXXXX	XXXXXXXXXX	XXXXXXXXXX	XXXXXXXXXX
gi686600796	XXXXXXXXXX	XXXXXXXXXX	XXXXXXXXXX	XXXXXXXXXX	XXXXXXXXXX
gi704535956	XXXXXXXXXX	XXXXXXXXXX	XXXXXXXXXX	XXXXXXXXXX	XXXXXXXXXX
gi555983692	XXXXXXXXXX	XXXXXXXXXX	XXXXXXXXXX	XXXXXXXXXX	XXXXXXXXXX
gi591300470	XXXXXXXXXX	XXXXXXXXXX	XXXXXXXXXX	XXXXXXXXXX	XXXXXXXXXX
gi255683553	XXXXXXXXXE	INKNMEGFHA	GNQCRNLMAH	PASLAAPSTA	----AYTSSE
gi255683559	XXXXXXXXXE	INKNMEGFHA	GNQCRNLMAH	PASLAAPSTA	----AYTSSE
gi558170872	XXXXXXXXXX	XXXXXXXXXX	XXXXXXXXXX	XXXXXXXXXX	XXXXXXXXXX
gi255683557	XXXXXXXXXE	INKNMEGFHA	GNQCRNLMAH	PASLAAPST-	---RAYTSSE
gi874491859	XXXXXXXXXX	XXXXXXXXXX	XXXXXXXXXX	XXXXXXXXXX	XXXXXXXXXX
gi726998761	XXXXXXXXXX	XXXXXXXXXX	XXXXXXXXXX	XXXXXXXXXX	XXXXXXXXXX
gi697489159	XXXXXXXXXX	XXXXXXXXXX	XXXXXXXXXX	XXXXXXXXXX	XXXXXXXXXX
gi525030647	XXXXXXXXXX	XXXXXXXXXX	XXXXXXXXXX	XXXXXXXXXX	XXXXXXXXXX
gi222159495	XXXXXXXXXE	MNRNMEGFAG	GNFAANQCRN	--LLAHPTSL	APSTTYTSSE
gi187936703	XXXXXXXXXX	XXXXXXXXXX	XXXXXXXXXX	XXXXXXXXXX	XXXXXXXXXX
gi690457118	XXXXXXXXXX	XXXXXXXXXX	XXXXXXXXXX	XXXXXXXXXX	XXXXXXXXXX
gi559163908	MEAGFHAAAA	AAAAGSNFVA	ANQCRNLMAH	PASLAAPGSS	AAAYTSSEAP

```
gi559163610  XXXXXXXXXX XXXXXXXXXX XNQCRNLMAH PASLAAPGGT AAYTSSEAPA
gi559163543  XXXXXXXXXX XXXXXXXXXX XXQCRNLMAH PASLAAPGST AAYTSSEAPA
gi704260001  XXXXXXXXXX XXXXXXXXXX XXXXXXXXXX XXXXXXXXXX XXXXXXXXXX
gi559163971  XXXXXXXXXX XXXXXXXXXX XNQCRNLMAH PASLAAPGSA SAYTSSEAPA
gi559163730  MEAGFHAAAA AAAAGTNFVS ANQCRNLMAH PASLAGPGSA AAYTSSEAPA
gi269204564  XXXXXXXXXX XXXXXXXXXX XXQCRNLMAH PASLAAPGSA AAYTSSEAPA
gi222159485  XXXXXXXXXE KNKNMDGFAG GNFAANQCRN --L-AHPASL APSTAYSSGE
gi559164035  XXXXXXXXXX XXXXXXXXXX XXQCRNLMAH -PASLAAPGS AAAAAYASSE
gi222708647  XXXXXXXXXE VNKNMEGFAG GNFAANQCRN --LLAHPASL APSTAYTSSE
gi290760385  XXXXXXXXXX XXXXXXXXXX XXXXXXXXXX XXXXXXXXXX XXXXXXXXXX
gi559163786  MEAGFHAAAA AAAASTNFVA ANQCRNLMAH PASLAGPGSA AAAAYPSSEA
gi559163846  MEAGFHAAAA AAAAGTNFVA ANQCRNLMAH PASLTGPGSA AAAYPSSEAP
gi318088612  XXXXNENNQN KSQIMEGLSG ATHCRELISH PALGRHSGTI ATHQGSVYSD
gi318088602  XXXXNENNQN KSQIMEGLSG ATHCRELISH PALGRHSGTI ATHQGSVYSD
gi222708673  XXXXXXXXXD MNKNMEGFVG ANFSANQCRN --LMAHPPSL APSSTYASTE
gi222708649  XXXXXXXXXE VNKSMEGFAG GNFAANQCRN --LMAHPASL APSTAYTSSE
gi222708671  XXXXXXXXXN INKSMEGFVG SNFTANQCRN --LVSHPTSL APSTTYASSE
gi222708655  XXXXXXXXXE VNKNMEGFAG GNFAANQCRN --LMAHPASL APSATYTSSE
gi222708681  XXXXXXXXXD MNKNMEGFVG GNFAANQCRN --LIAHPSS- -LAPSTTYTS
gi318088600  XXXXNENNQN KTKIMEGLSG ATPCRDRISH PALGRHSGTI ATHQGSVDLD
gi318088598  XXXXNENNQN KSQIMEGLSG ATHCRELISH PALGRHSGTI ATHQGSVYSD
gi222708679  XXXXXXXXXD MNKNMEGFVG GNFAANQCRN --LIAHPSS- -LAPSTTYTS
gi318088608  XXXXNENIQN KSQIMEGLRG ATHCRELISH PALGRHSGTI ATHQGSAYSD
gi559164131  XXXXXXXXXX XXXAMEGLSG ASHCRDLITH PVLGRHSSTI GTHQGSVYTD
gi318088606  XXXXNENNQN KTQIMEGLSG ATLCRELISH PALGRHSGTI ATHQGSVYSD
gi318088610  XXXXXXXNLN KSPIMEGLSG ATHCRKLISH PALGRHSGTI ATHQGSVYSD
gi222708677  XXXXXXXXXD MSKNMEGFVG GNFAANQCRN --LIAHPSS- -LAPSTTYTS
gi222708657  XXXXXXAADE INKNMEGFAG ASQCRNLMAH --------AP SLAPSSGYSD
gi700431350  XXXXXXXXXX XXXXXXXXXX XXXXXXXXXX XXXXXXXXXX XXXXXXXXXX
gi387231673  XXXXXXXXXX XXXXXXXXXX XXXXXXXXXX XXXXXXXXXX XXXXXXXXXX
gi661918810  XXXXXXXXXX XXXXXXXXXX XXQCRNFLSS PVFGAHSGRS AAAPGFAYAP
gi387231677  XXXXXXXXXX XXXXXXXXXX XXXXXXXXXX XXXXXXXXXX XXXXXXXXXX
gi387231659  XXXXXXXXXX XXXXXXXXXX XXXXXXXXXX XXXXXXXXXX XXXXXXXXXX
gi325260872  XXXXXXXXXX XXXXVDSLIG HEGDELLASR FPATGAHPAA ARPSGLVPDC
gi387231661  XXXXXXXXXX XXXXXXXXXX XXXXXXXXXX XXXXXXXXXX XXXXXXXXXX
gi387231655  XXXXXXXXXX XXXXXXXXXX XXXXXXXXXX XXXXXXXXXX XXXXXXXXXX
gi387231679  XXXXXXXXXX XXXXXXXXXX XXXXXXXXXX XXXXXXXXXX XXXXXXXXXX
gi387231667  XXXXXXXXXX XXXXXXXXXX XXXXXXXXXX XXXXXXXXXX XXXXXXXXXX
gi387231227  XXXXXXXXXX XXXXXXXXXX XXXXXXXXXX XXXXXXXXXX XXXXXXXXXX
gi387231653  XXXXXXXXXX XXXXXXXXXX XXXXXXXXXX XXXXXXXXXX XXXXXXXXXX
gi699575612  XXXXXXXXXX XXXXXXXXXX XXXXXXXXXX XXXXXXXXXX XXXXXXXXXX
gi676248171  XXXXXXXXXX XXXXXXXXXX XXXXXXXXXX XXXXXXXXXX XXXXXXXXXX
gi723145109  XXXXXXXXXX XXXXXXXXXX XXXXXXXXXX XXXXXXXXXX XXXXXXXXXX
gi697016891  XXXXXXXXXX XXXXXXXXXX XXXXXXXXXX XXXXXXXXXX XXXXXXXXXX
gi677147237  XXXXXXXXXX XXXXXXXXXX XXXXXXXXXX XXXXXXXXXX XXXXXXXXXX
gi256014558  XXXXNERHRD KSPAMEGLCP SSHCRDLISH PALGRHSSAI GAHQGSIYTD
gi679242416  XXXXXXXXXX XXXXXXXXXX XXXXXXXXXX XXXXXXXXXX XXXXXXXXXX
gi683471095  XXXXXXXXXX XXXXXXXXXX XXXXXXXXXX XXXXXXXXXX XXXXXXXXXX
  gi9438781  XXXXXXXXXX XXXXXXXXXX XXXXXXXXXX XXXXXXXXXX XXXXXXXXXX
gi676827072  XXXXXXXXXX XXXXXXXXXX XXXXXXXXXX XXXXXXXXXX XXXXXXXXXX
gi387231651  XXXXXXXXXX XXXXXXXXXX XXXXXXXXXX XXXXXXXXXX XXXXXXXXXX
gi677498936  XXXXXXXXXX XXXXXXXXXX XXXXXXXXXX XXXXXXXXXX XXXXXXXXXX
gi683474613  XXXXXXXXXX XXXXXXXXXX XXXXXXXXXX XXXXXXXXXX XXXXXXXXXX
gi387231675  XXXXXXXXXX XXXXXXXXXX XXXXXXXXXX XXXXXXXXXX XXXXXXXXXX
gi677248448  XXXXXXXXXX XXXXXXXXXX XXXXXXXXXX XXXXXXXXXX XXXXXXXXXX
gi678138507  XXXXXXXXXX XXXXXXXXXX XXXXXXXXXX XXXXXXXXXX XXXXXXXXXX
gi700349256  XXXXXXXXXX XXXXXXXXXX XXXXXXXXXX XXXXXXXXXX XXXXXXXXXX
gi677225924  XXXXXXXXXX XXXXXXXXXX XXXXXXXXXX XXXXXXXXXX XXXXXXXXXX
gi121308926  XXXXXXXXXX XXXXXXXXXX XXXXXXXXXX XXXXXXXXXX XXXXXXXXXX
gi194591945  XXXXXXXXXX XXXXXXXXXX XXXXXXXXXX XXXXXXXXXX XXXXXXXXXX
gi195549518  XXXXXXXXXX XXXXXXXXXX XXXXXXXXXX XXXXXXXXXX XXXXXXXXXX
gi194591943  XXXXXXXXXX XXXXXXXXXX XXXXXXXXXX XXXXXXXXXX XXXXXXXXXX
gi387231665  XXXXXXXXXX XXXXXXXXXX XXXXXXXXXX XXXXXXXXXX XXXXXXXXXX
gi195549516  XXXXXXXXXX XXXXXXXXXX XXXXXXXXXX XXXXXXXXXX XXXXXXXXXX
 gi34304667  XXXXXXXXXX XXXXXXXXXX XXXXXXXXXX XXXXXXXXXX XXXXXXXXXX
gi194396133  XXXXXXXXXX XXXXXXXXXX XXXXXXXXXX XXXXXXXXXX XXXXXXXXXX
gi387231669  XXXXXXXXXX XXXXXXXXXX XXXXXXXXXX XXXXXXXXXX XXXXXXXXXX
gi429510514  XXXXXXXXXX XXXXXXXXXX XXXXXXXXXX XXXXXXXXXX XXXXXXXXXX
gi780155006  XXXXXXXXXX XXXXXXXXXX XXXXXXXXXX XXXXXXXXXX XXXXXXXXXX
```

```
gi387231671  XXXXXXXXXX XXXXXXXXXX XXXXXXXXXX XXXXXXXXXX XXXXXXXXXX
gi387231657  XXXXXXXXXX XXXXXXXXXX XXXXXXXXXX XXXXXXXXXX XXXXXXXXXX
gi387231649  XXXXXXXXXX XXXXXXXXXX XXXXXXXXXX XXXXXXXXXX XXXXXXXXXX
gi229298086  XXXXXXXXXX XXXXXXXXXX XXXXXXXXXX XXXXXXXXXX XXXXXXXXXX
gi328682725  XXXXXXXXXX XXXXXXXXXX XXXXXXXXXX XXXXXXXXXX XXXXXXXXXX
gi217035843  XXXXXXXXXX XXXXXXXXXX XXXXXXXXXX XXXXXXXXXX XXXXXXXXXX

             51                                               100
      QUERY  LDLPGSAEPP KQCHPCPGVP QGTSPAPVPY GYFGGGYYSC RVSRSSLKPC
gi685597136  LDLPGSAEPP KQCHPCPGVP QGTSPAPVPY GYFGGGYYSC RVSRSSLKPC
 gi60653709  LDLPGSAEPP KQCHPCPGVP QGTSPAPVPY GYFGGGYYSC RVSRSSLKPC
gi795250298  LDLPGSVEPP KQCHPCPGVP QGTSPAPVPY GYFGGGYYSC RVSRSSLKPC
gi635093043  LDLPGSAEPP KQCHPCPGVP QGTSPAPVPY GYFGGGYYSC RVSRSSLKPC
gi109114206  LDLPGSAEPP KQCHPCPGVP QGTSPAPVPY GYFGGGYYSC RVSRSSLKPC
gi795583451  LDLPGSAEPP KQCHPCPGVP QGTSPAPVPY GYFGGGYYSC RVSRSSLKPC
gi559812841  LDLPGSAEPP KQCHPCPGVP QGTSPAPVPY GYFGGGYYSC RVSRSSLKPC
gi332259422  LDLPGSVEPP KQCHPCPGVP QGTSPAPVPY GYFGGGYYSC RVPRSSLKPC
gi795525940  LDLPGSAEPP KQCHPCPGVP QGTSPAPVPY GYFGGGYYSC RVSRSSLKPC
gi426237811  LDLPGSAEPP KQCHPCPGMP QGASPAPVPH GYFGGGYYSC RVSRSSLKPC
gi744588246  LDLPGSAEPP KQCHPCPGVP QGAPPAPVPY GYLGGGYSSC RVSRSSLKPC
gi617633383  LELPGSAEPP KQCHPCPPVP QGASPAPVPY SYFGGGYYSC RVSRSSLKPC
gi213511801  VDVSGSGEPG KQCTPCPAVP QGSSTGPLPY GYFGNGYYPC RMGRGSLKSC
gi444517714  LDLPGSAEPP KQCHPCPGVP QGASPAPVPY GYFGGGYYSC RVSRSSLKPC
gi640808225  LDLPGSAEPP KQCHPCPGVP QGASPAPVPY GYFGGGYYSC RVSRSSLKPC
gi556958424  MSGSGSGEVA KQCTPCPAVP QSSSTAPLPY GYFGSGYYSC RMGRGSLKSC
gi667260003  LDLPGSVEPP KQCHPCPGVP QGASPAPVPY GYFGGGYYSC RVSRSSLKPC
gi831328207  VPGSGSSEAG KQCTPCPGVP QGSSTGPLPY GYFGNGYYPC RMGRGSLKSC
gi642105600  LDVSGSGEPG KQCTPCPVVP QGSSTGPLPY GYFGNGYYPC RMGRGSLKSC
gi471380475  LDLPGSAEPP KQCHPCPGVP QGASPAPVPY GYFGGGYYSC RVSRSSLKPC
gi674059771  LDLPGSAEPP KQCHPCSGVP QGASSAPVPY GYFGGGYYSC RVSRSSLKPC
gi344285419  LDLPGSAEPP KQCHPCPGVP QGASPAPVPY GYFGGGYYSC RVSRSSLKPC
gi742113362  VSGSSSGELG KQCTPCPAVP QGSSTAPLPY GYFGNGYYPC RMGRGSLKSC
gi852732630  LDLPGSAEPP KQCHPCPGVP QGASPAPVPY GYFGGGYYSC RVSRSSLKPC
gi512996173  LDLPGSPEPP KQCHPCPGVP QGASPAPVPY GYFGGGYYSC RVSRSSLKPC
gi395826626  LDLPGPTEPP KQCHPCPGVP QGASPAPMPY GYFGGGYYSC RVSRSSLKPC
gi126308251  IDLPGSGEPP KQCHPCPGVP QGGSPTSVPY GYFGGGYYSC RVSRSSLKPC
gi528767552  LDLPGSAEPP KQCHPCPGVP QGAPPAPVPY GYFGGGYYSC RVSRSSLKPC
gi532208489  LDLPGSAEPP KQCHPCSGVP QGASPAPVPY GYFGGGYYSC RVSRSSLKPC
gi507654822  LDLPGSAEPP KQCHPCPGVP QGASPAPVPY GYFGGGYYSC RVSRSSLKPC
gi488527225  LDLPGSAESS KQCHPCPGLS QGAPSAPVPY GYFGGGYYSC RVSRSSLKPC
gi817274366  LDLPGSAEPP KQCHPCSGVP QGASPAPVPY GYFGGGYYSC RVSRSSLKPC
gi585671866  LDLPGSAEPP KQCHPCPGVP QGASPTPVPY GYFGGGYYSC RVSRSSLKPC
gi573901311  VSGSGSGEPG KQCAPCPAVP QGTSTAPLPY GYFGNGYYSC RMGRGSLKSC
gi532071900  LDLPGSAEPP KQCHPCPGVP QRASPAPVPY GYFGGGYYSC RVSRSSLKPC
gi114149320  VDVSSSAESG KQCTPCPTVP QASSTGPIPY GYFGNSYYPC RMGRGSLKSC
gi593727116  LDLPGSAEPP KQCHPCPGVP QGASPAPVPY GYFGGGYYSC RVSRSSLKPC
gi470606860  LDLPGSAEPP KQCHPCPGVP QGASPAPVPY GYFGGGYYSC RVSRSSLKPC
gi291405863  LDLPGPAEPP KQCHPCPGVP QGASPAPVPY GYFGGGYYSC RVSRSSLKSC
gi594656397  LDLPGSAEPP KQCHPCPGVP QGASPAPVPY GYFGGGYYSC RVSRSSLKPC
gi634851243  LDLPGSAEQP KQCHPCPGVP QGASPAPVPY GYFGGGYYSC RVSRSSLKPC
gi602709820  LDLPGSAEPP KQCHPCPGVP QGASPAPVPY GYFGGGYYSC RVSRSSLKPC
gi826271801  LDLPGSAEPP KQCHPCPGVP QGASPAPVPY GYFGGGYYSC RVSRSSLKPC
gi589917310  LDLPGSAEPP KQCHPCPGVP QGASPAPVPY GYFGGGYYSC RVSRSSLKPC
gi507543189  LDLPGSAEPP KQCHPCPGVP QGASPAPVPY GYFGGGYYSC RVSRSSLKPC
gi395532657  IDLPGSGDPP KQCHPCPGMP QGGSATSVPY GYFGGGYYSC RVSRSSLKPC
gi597740267  VSTSSAAETS KQCTPCPTVP QASSTGPIPY GYFGNSYYPC RMGRGSLKSC
gi725576032  LDLPGSAEPP KQCHPCSGAP QGASPAPVPY GYFGGGYYSC RVSRSSLKPC
gi742135928  LDLPGSAEPP KQCHPCPGVP QGASPAPVPY GYFGGGYYSC RVSRSSLKPC
gi348562271  LDLPGSGEPP KQCHPCPGVP QGASPAPVPY GYFGGGYYSC RVSRSSLKPC
gi594050844  LDLPGSAEPP KQCHPCPGVP QGASPAPVPY GYFGGGYYSC RVSRSSLKPC
gi533160875  LDLPGSAEPP KQCHPCPGVP QGASPAPVPY GYFGGGYYSC RVSRSSLKPC
gi556738344  LDLPGSAEQP KQCHPCSGVP QGASPAPVPY GYFGGGYYSC RVSRSSLKPC
gi803246204  LDLPGSAEPP KQCHPCPGMP QGASPAPVPY GYFGGGYYSC RVSRSSLKPC
gi354474760  LDLPGSAEPP KQCHPCPGVP QGASPAPVPY GYFGGGYYSC RVSRSSLKPC
gi532051321  LDLPGSAEPP KQCHPCPGVP QGASPAPVPY GYFGGGYYSC RVSRSSLKPC
gi311267502  LDLPGSAEPP KQCHPCSGVP QGASPAPVPY GYFGGGYYSC RVSRSSLKPC
gi743729506  LDLPGSAEPP KQCHPCPGVP QGAPPAPVPY GYFGGGYYSC RVSRSSLKPC
gi359754103  IDLPGSGEPP KQCHPCPGVP QGGSPTSVPY GYFGGGYYSC RVSRSSLKPC
gi576886849  VSTSSSGETS KQCTPCPTVP QASSTGPIPY GYFGNSYYPC RMGRGSLKPC
```

```
gi586448761  LDLPGSAEPS KQCHPCPGVP QGASPAPVPY GYFGGGYYSC RVSRSSLKPC
gi504159820  LDLPGSAEPP KQCHPCPGMP QGASPAPVPY GYFGGGYYSC RVSRSSLKPC
gi524951862  LDLPGSAEPP KQCHPCPGVP QGASPAPVPY GYFGGGYYSC RVSRSSLKPC
gi332692483  VSGSGSGEAG KQCAPCPTVP QSSSTTPLPY GYFGNSYYPC RMGRGSLKSC
gi385654474  VSGSGSGEAG KQCAPCPTVP QSSSTTPLPY GYFGNSYYPC RMGRGSLKSC
gi478520959  LDLPGSAEPP KQCHPCPGVP QGASPAPVPY GYFGGGYYSC RVSRSSLKPS
gi301128881  VDVPGSGEPG KQCTPCP-MP QASTAAPLPY GYFGSGYYSC RMGRNSIKPC
gi1011609041 MDAAGPGEPS KQCIPCPAVP QGSSTTPLPY GYFGSGYYSC RVARSSLKPC
gi664747354  LDLPGSAEPP KQCHPYPGVP QGASPAPVPY GYFGGGYYSC RVSRSSLKPS
gi557280818  MDAAGPGEPS KQCIPCPAVP QGSSTTPLPY GYFGSGYYSC RVARSSLKPC
gi359076815  LDLPGSAEPP KQCHPCPGVP QGASPAPVPY GYFGGGYYSC RVSRSSLKPC
gi632972216  VDMSGSGEPG KQCAPCPPLP QGSPAAPLPY GYFGSGYYSC RMGRGPIKSC
gi759185571  LDLPGSAEPP KQCHPCPGVP QGASPAPVPY GYFGGGYYSC RVSRSSLKPC
gi472350967  LDLPGSAEPP KQCHSCPGLP QGAAPAPVPY GYFGGGYYSC RVSRSSLKPC
gi586539979  LDLPGSAEPP KQCHPCPGVP QGASPAPVPY GYFGGGYYSC RVSRSSLKPC
gi157823841  LDLPGSAEPP KQCHPCPGVP QGASPAPVPY GYFGGGYYSC RVSRSSLKPC
gi410980861  LDLPGSAEPP KQCHPCPGVP QGAAPAPVPY GYFGGGYYSC RVSRSSLKPC
gi829772881  LDLPGSAEPP KQCHPCPGVP QGASPAPVPY GYFGGGYYSC RVSRSSLKPC
gi220898176  VPASGIAEPV KQCSPCSAAQ SSSSA-SLPY GYFGSSYYPC RMTHHNIKSC
gi1519038    LDLPGSAEPP KQCHPCPGVP QGASPAPVPY GYFGGGYYSC RVSRSSLKPC
gi731207261  LDLPGSAEPP KQCHPCPGVP QGGSPAPVPY GYFGGGYYSC RVSRSSLKPC
gi20278975   VPASGIAEPV KQCSPCPAA- QTSSSAALPY GYFGSSYYPC RMSHHSIKSC
gi585176637  LDLPGSAEPP KQCQPCPGLP QGAAPAPVPY GYFGGGYYSC RVSRSSLKPC
gi560984071  LDLPGSAEPP KQCHPCPGVP QGPPPAPVPY GYFGGGYYSC RVSRSSLKPC
gi73966255   LDLPGPAEPP KQCHPCPGVP QGAAPAPVPY GYFGGGYYSC RVARSSLKPC
gi511890491  LDLPGSAEPP KQCHPCPGVP QGTAPAAVPY GYFGGGYYSC RVSRSSLKPC
gi254212171  SELPASGEPV KQCSPCPAA- QTSSSASLPY GYFGSSYYPC RMSHHSIKSC
gi558229251  APASGMAEPA KQCSPCSAAV QSSSGAALPY GYFGSGYYPC RMTHHNIKSC
gi752394348  LDLPGSAEPP KQCHPCPGVP QGPAPAPVPY GYFGGGYYSC RVSRSALKPC
gi507952779  LDLPGPAEPP KQCHPCPPVP QRVSPAPVPY GYFGGGYYSC RVSHSSLKPC
gi507664676  AELPGSAEPP KQCHPCPGVP QGASPAPVPY GYFGGGYYSC RVSRSSLKPS
gi224045310  APAAGMAEPA KQCSPCSAAV QSSSGAALPY GYFGSGYYPC RMTHHNIKSC
gi400180333  VPVSGMAEPV KQCSPCSAAQ SSSSA-SLPY GYFGSGYYPC RMAHHSIKSC
gi641724374  LDLPGSSEPP KQCHPCPGAP QGASPAPVPY GYFGGGYYSC RLARGSLKPC
gi693302455  APAPGVAEPG KQCSPCPG-- --SSGAALPY GYFGSGYYPC RVTHNAIKSC
gi542179924  VDVSASGSAA PSGKQCAAVP HQSSSAALPY SYFGNGYYPC RMGRGPLKSC
gi119370791  VDVTATSSSG KQCAPCPAAT QSSSAAALPY SYFGNGYYPC RMGRGSLKSC
gi678126544  VPVSGMGEPV KQCSPCSAAQ SSSSA-SLPY SYFGSGYYPC RMSHHSIKSC
gi808884419  VDVSASGSGE KQCSPCPAAA QSSSAAALPY SYFGNGYYPC RMGRGSLKSC
gi505802773  LDLPGSAEPP KQCPPCPPGP PQASPAPVPY SYFGGGYYSC RVSRSSLKPC
gi34484442   VDVTATSSSG KQCGPCPGAT QSSSAAALPY SYFGNGYYPC RMGRGSLKSC
gi519777295  ---EGPAEAG KQCSPCSA-- QGSSGAALPY GYFGSGYYPC RVGHHGIKSC
gi657803392  VDVSGSASAG KQCSPCPAAH QSSSATTLPY GYFSNGYYPC RMGRGSLKSC
gi554874539  VDVSASGSAA SSGKQCAAVP HQSSSAALPY SYFGNGYYPC RMGRAPLKSC
gi499036069  MDVSASASSG KQCAAVPH-Q NSSAATALPY SYFGNGYYPC RMGRAPLKSC
gi301607689  SEVPVSAEPA KQCNPCSAV- QSTPNASLPY GYFGSGYYPC RMSHHNIKSC
gi584023332  VDVSASGSAA SSGKQCAAVP HQSSSAALPY SYFGNGYYPC RMGRAPLKSC
gi548549273  VDVSASASSG KQCAAVPH-Q NSSAATALPY SYFGNGYYPC RMGRAPLKSC
gi657574876  VDVSASASSG KQCAAGPCVP HQSSSAALPY SYFGNGYYPC RMGRGSLKSC
gi28268588   SEVPVSAEPS KQCNPCSAV- QSTPNGSLPY GYFGSGYYPC RMSHHNIKSC
gi736251858  VEVSASGSAG KQCAPCPGAA QSSSAASLPY SYFGNGYYPC RMGRGSVKSC
gi530624973  LDVPGAGEPS KQCIPCPAIP QSSSTTPLPY GYFGSGYYSC RVARSALKPC
gi290760363  SEAPGMAETG KQCSPCSAAV QGSSPAALPY SYFSGGYFPC SVNHHSLKSC
gi551520979  VDVSTSSSSG KQCGPCPAAA QSSSAAALPY SYFGNSYYPC RMGRSSIKSC
gi658861163  VDVSTSSSSG KQCGPCPAAA QSSSAAALPY SYFGNSYYPC RMGRSSIKSC
gi617509429  VDVSTSSSSG KQCGPCPAAA QSSSAAALPY SYFGNSYYPC RMGRSSIKSC
gi47229429   CDVPASAEPV KQCSPCSAAQ NSSSA-SLPY GYFGSGYYPC RMSHHSIKSC
gi831555258  ASASAVGEPV KQCSPCSAA- QNSTSASLPY GYFG---YPC RMSHHSIKSC
gi591374792  LDAPGPGEPS KQCIPCPAIS QSSSTTPLPY GYFGSGYYSC RVARSSLKPC
gi432871359  VDVSGSGETA ATSAAAAAMP HQSSSAALPY GYFGNGYYPC RMGRGSLKSC
gi602643660  SEAPGMAEPG KQCSPCSAAV QGASPAALPY SYFGSGYFPC SVNHHSLKSC
gi961915500  VPVSGVGEPG KQCSPCSAT- QSSPNASLPY GYFGSGYYPC RVSHGGVKAC
gi961860111  VPVSGVGEPG KQCSPCSAT- QSSPNASLPY GYFGSGYYPC RVSHGGVKAC
gi219879168  VPGPGMGEPV KQCSPCSAV- QNSPSASLPY GYFGGSYYPC RMP----KSC
gi219879156  VPGPGMGEPV KQCGPCSAV- QNSPSASLPY GYFGGSYYPC RMP----KSC
gi219879164  SEVPGMGEPV KQCSPCSAV- QNSPSASLPY GYFGGSYYPC RMP----KSC
gi123204435  SEVPGMGEPV KQCSPCSAV- QNSPSASLPY GYFGGSYYPC RMP----KSC
gi465985114  XXXXXXXXXX KQCSPCSAAA QSSSPAALPY GYFGSGYYPC RMGPHAIKSC
gi426355784  XXXXXXXXXX KQCSPCSAAA QSSSPAALPY GYFGSGYYPC RMGPHAIKSC
```

gi1034052196	PAGPAGAEAA	KQCSPCSAAA	QSSSPAALPY	GYFGSGYYPC	RMGPHAIKSC
gi683901043	XXXXXXXXXX	XXXXXXXXXX	XXXXXXXXXX	XXXXXXXXXX	XXXHNAIKSC
gi729725749	XXXXXXXXXX	XXXXXXXXXX	XPAASAPLPY	GYFGSGYYSC	RVARSSLKTG
gi663243102	XXXXXXXXXX	XXXXXXXXXX	XXXXXXXXPY	GYFGSGYYSC	RVSRSALKAG
gi339792792	SGAEGSSEAG	KQCSPCSA-A	QSSSGASLPY	GYFGSGYYPC	RVGHHAIKSC
gi768393729	XXXXXXXXXX	XXXXXXXXXX	XXXXXXXXXY	GYFGSGYYSC	RVARSSLKAG
gi670977729	XXXXXXXXXX	XXXXXXXXXX	XXXXXXXXXX	XXXXXXXXXC	RVSRSALKPC
gi327263844	IPAPDAA---	RQCPPPPPPP	PPPAASSLGY	GYFGGSYYGC	RLSHAQQKPC
gi1040268871	IPAP---EAA	RQCAP-PPAP	PTSSSATLGY	GYFGGSYYGC	RLSHNQQKPC
gi303303637	IPAP---EAA	RQCAP-PPAP	PTSSSATLGY	GYFGGSYYGC	RLSHNQQKPC
gi724877051	IPAP---EAA	RQCAP-PPAP	PTSSSATLGY	GYFGGSYYGC	RLSHNQQKPC
gi544470246	IPAP---EAA	RQCAP-PPAP	PTSSSATLGY	GYFGGSYYGC	RLSHNQQKPC
gi338726307	IPTP---EAA	RQCAP-PPAP	PTSSSATLGY	GYFGGSYYGC	RLSHNQQKPC
gi297692036	IPAP---EAA	RQCAP-PPAP	PTSSSATLGY	GYFGGSYYGC	RLSHNQQKPC
gi397521957	IPAP---EAA	RQCAP-PPAP	PTSSSATLGY	GYFGGSYYGC	RLSHNQQKPC
gi1018974175	XXXXXXXXXV	KQCSPCSAAA	QSSSSGALPY	GYFGSGYYPC	RMGHHAIKSC
gi675430530	XXXXXXXXXX	XXXXXXXXXX	XXXXXPALGY	GYFGNGYYSC	RMSHNALKSP
gi795174618	XXXXXXXXXX	XXXXXXXXXX	XXXXXXXLGY	GYFGNGYYSC	RMSHNALKSS
gi699633156	XXXXXXXXXX	XXXXXXXXXX	XXXXXXXXXX	GYFGNGYYSC	RMSHNALKSP
gi374498883	XXXXXXXXXX	XXXXXXXXXX	XXXXXXXXXY	GYFGNGYYSC	RMSHNALKSP
gi24637241	IPAP---EAA	RQC----PAP	TASSNAALGY	GYFGGSYYGC	RLSH------
gi584059997	XXXXXXXXXX	XXXXXXXXXX	XXXXXXXXXX	XXXXXXXXXX	XXXXXXXXXX
gi884764069	XXXXXXXXXX	XXXXXXXXXX	XXAAAAPLPY	GYFGSGYYSC	RVARSALKAG
gi66773958	XXXXXXXXXX	XXXXXXXXXX	XXXXXXXLGY	GYFGNGYYSC	RMSHNALKSS
gi224016307	XXXXXXXXXX	XXXXXXXXXX	XXXXXXXLGY	GYFGNGYYSC	RMSHNALKSS
gi224016295	XXXXXXXXXX	XXXXXXXXXX	XXXXXXXLGY	GYFGNGYYSC	RMSHNALKSS
gi224016303	XXXXXXASSA	KECPAPGAAA	AAPPGAPLGY	GYFGNGYYSC	RMSHNALKSS
gi224016309	XXXXXXASSA	KECPAPGAAA	AAPPGAPLGY	GYFGNGYYSC	RMSHNALKSS
gi374498861	XXXXXXXXXX	XXXXXXXXXX	XXXXXXXXXY	GYFGNGYYSC	RMAHGALKAA
gi224016313	XXXXXXASSA	KECSAPGAAA	AAPPGAPLGY	GYFGNGYYSC	RMSHNALKSS
gi224016305	XXXXXXXXXX	XXXXXXXXXX	XXXXXXXLGY	GYFGNGYYSC	RMSHNALKSS
gi224016311	XXXXXXASSA	KECPAPGAAA	AAPPGAPLGY	GYFGNGYYSC	RMSHNALKSS
gi224016299	XXXXXXXXXX	XXXXXXXXXX	XXXXXXXLGY	GYFGNGYYSC	RMSHNALKSS
gi700384804	XXXXXXXXXX	XXXXXXXXXX	XXXXXXXXXX	XXXXXGYYSC	RMSHNALKSP
gi543378707	XXXXXXXXXX	XXXXXXXXXX	XXXXXXXLPY	GYFGSGFYSC	RVSRSSLKPA
gi699659589	XXXXXXXXXX	XXXXXXXXXX	XXXXXXXXXX	XXXXXXXXXC	RVSRSSLKTG
gi310656724	--APGCGYPG	QE-------R	AEASKEALGY	GYFGNRYYGV	GIQQPPLKA-
gi565321354	XXXXXXXXXX	XXXXXXXXXX	XXXXXXXXXX	XXXXXXXXXX	XXXXXXXXXX
gi290760375	XXXXXXXXXX	XXXXXXXXXX	XXXXXXXXXX	XXXXXXYYSC	RMAHGALKAS
gi696966138	XXXXXXXXXX	XXXXXXXXXX	XXXXXXXXXX	XXXXXXXXXX	XXXXXXXXXX
gi677975869	XXXXXXXXXX	XXXXXXXXXX	XXXXXXXXXX	XXXXXXXXXX	XXXXXXXXXX
gi543746989	XXXXXXXXXX	XXXXXXXXXX	XXXXXXXXXX	XXXXXXXXXX	XXXXXXXXXX
gi701389587	XXXXXXXXXX	XXXXXXXXXX	XXXXXXXXXX	XXXXXXXXXX	XXXXXXXXXX
gi256014533	IPAPDAA---	RQC----PAP	PASSNATLGY	SYFGGSYYGC	RLSHSQQKPC
gi669281149	XXXXXXXXXX	XXXXXXXXXX	XXXXXXXXXX	XXXXXXXXXX	XXXXXXXXXX
gi118102888	XXXXXXXXXX	XXXXXXXXXX	XXXXXXXXXX	XXXXXXXXSC	RVARSALKAG
gi554547429	VSVQTLGAPG	EPRHDALLPV	DSYQPWALPG	GW--------	----------
gi694854743	XXXXXXXXXX	XXXXXXXXXX	XXXXXXXXXX	XXXXXXXXXC	RLSHSQQKPC
gi529426764	XXXXXXXXXX	XXXXXXXXXX	XXXXXXXXXX	XXXXGGYYGC	RLSHSQQKPC
gi706150961	XXXXXXXXXX	XXXXXXXXXX	XXXXXXXXXX	XXXXXXXXXX	XXXXXXXXXX
gi719780455	XXXXXXXXXX	XXXXXXXXXX	XXXXXXXXXX	XXXXXXXXXX	XXXXXXXXXX
gi290760370	XXXXXXXXXX	XXXXXXXXXX	XXXXXXXXXX	XXXXGGYYSC	RMAHGALKAS
gi328682541	HALYDSPKDC	PPCPPCPGST	APSTP-SFSY	GYFGNGYYSC	RMSHNSLKSS
gi541961056	XXXXXXXXXX	XXXXXXXXXX	XXXXXXXXXX	XXXXXXXXXC	RLSHSQQKPC
gi733928584	XXXXXXXXXX	XXXXXXXXXX	XXXXXXXXXX	XXXXXXXXXX	XXXXXXXXXX
gi686600796	XXXXXXXXXX	XXXXXXXXXX	XXXXXXXXXX	XXXXXXXXXC	RLSHSQQKPC
gi704535956	XXXXXXXXXX	XXXXXXXXXX	XXXXXXXXXX	XXXXXXXXXX	XXXXXXXXXX
gi555983692	XXXXXXXXXX	XXXXXXXXXX	XXXXXXXXXX	XXXXXXXXXX	XXXXXXXXXX
gi591300470	XXXXXXXXXX	XXXXXXXXXX	XXXXXXXXXX	XXXXXXXXXX	XXXXXXXXXX
gi255683553	APASGMAESA	KQCSPCSAAV	QSSSGAALPY	GYFGSGYYPC	RMTHHNIKSC
gi255683559	ARASGMAESA	KQCSPCSAAV	QSSSGAALPY	GYFGSGYYPC	RMTHHNIKSC
gi558170872	XXXXXXXXXX	XXXXXXXXXX	XXXXXXXXXX	XXXXXXXXXX	XXXXXXXXXX
gi255683557	APASGMAESA	KQCSPCSAAV	QSSSGAALPY	GYFGSGYYPC	RMTHHNIKSC
gi874491859	XXXXXXXXXX	XXXXXXXXXX	XXXXXXXXXX	XXXXXXXXXX	XXXXXXXXXX
gi726998761	XXXXXXXXXX	XXXXXXXXXX	XXXXXXXXXX	XXXXXXXXXX	XXXXXXXXXX
gi697489159	XXXXXXXXXX	XXXXXXXXXX	XXXXXXXXXX	XXXXXXXXXX	XXXXXXXXXX
gi525030647	XXXXXXXXXX	XXXXXXXXXX	XXXXXXXXXX	XXXXXXXXXX	XXXXXXXXXX
gi222159495	VPVSGMGEPV	KQCSPCSAT-	QSSPNASLPY	GYFGSSYYQC	RMSHSSVKSC
gi187936703	XXXXXXXXXX	XXXXXXXXXX	XXXXXXXXXX	XXXXXXXXXX	XXXXXXXXXX

```
gi690457118  XXXXXXXXXX XXXXXXXXXX XXXXXXXXXX XXXXGGYYGC RLSHSQQKPC
gi559163908  PAAPGMAESG KQCSPCSAAV QGSSPAALPY GYFGSGYFPC GMNHHALKSC
gi559163610  AGMVEPGAAV KQCSPCSAAV QSSSPAALPY GYFGSGYFPC RMSHHNLKSC
gi559163543  AGMAETGAAV KQCSPCSAAV QSSSPAALPY GYFSSGYFPC RMSHHNLKSC
gi704260001  XXXXXXXXXX XXXXXXXXXX XXXXXXXXXX XXXXXXXXXX XXXXXQQKPC
gi559163971  --AAGMAEPG NKCSPCSAAV QSSSPAALPY GYFGSGYYPC RMNHHNLKSC
gi559163730  AAGMAEPAAV KQCSPCSAAV QSPPPAALPY GYFGSGYFPC GMNHHSLKSC
gi269204564  AAAGMAEPSV KQCSPCSAAV QSSSPAALPY GYFGSGYFPC RMSHHNLKSC
gi222159485  VPVSGIGESA KQCSPCSA-A QSSSSASLPY GYFGSGYYPC RMSHHSIKSC
gi559164035  APAAGMAEPG KQCSPCSAAV QSSSPAALPY GYFGSGYYPC RMSHHNLKSC
gi222708647  VPVSGMAEPV KQCSPCSAAQ SSSSA-SLPY GYFGSGYYPC RMTHHSIKSC
gi290760385  XXXXXXXXXX XXXXXXXXXX XXXXXXXXXX XXXXXXYYSC RMSHNPLKAA
gi559163786  PSAAGMAETG KQCSPCSAAV QGSSPAALPY NYFSSGYFPC SVNHHSLKSC
gi559163846  SAAGMAEAGV KQCSPCSAAV QNPSPAALPY GYFGSGYFPC SVNNASLKSC
gi318088612  ISSP---ETG RQC----PAP QTSSSASLSY GYFGNPYYGC RLSHSQQKPC
gi318088602  ISSP---ETG RQC----PAP QTSSSASLSY GYFGNPYYGC RLSHSQQKPC
gi222708673  VPVSGMGEPV KQCSPCSAA- QSSPGATLPY GYFGSSYYPC RMSHHSVKSC
gi222708649  VPVSGMAEPV KQCSPCSAAQ SSSSA-SLPY GYFGSGYYPC RMTHHSIKSC
gi222708671  VPVSGMSEAV KQCSPCSAV- QSSPGASLPY GYFGSGYYQC RMSHHNSKSC
gi222708655  VPASGMGESV KQCSPCSAAQ SSTSA-SLPY GYFGSSYYPC RMSHSNIKSC
gi222708681  SEVPGMGEPV KQCSPCSAV- QNSPSASLPY GYFGGSYYQC RMP----KSC
gi318088600  IFSP---ETG RQC----PAP QTSSSASLSY GYFGNPYYGC RLSHSQEKPC
gi318088598  ISSP---ETG RQC----PAP QTSSSASLSY GYFGNPYYGS RLSHSQQKPC
gi222708679  SEVPGMGEPV KQCSPCSA-- QNSPGASLPY GYFGGSYYPC RMP----KSC
gi318088608  ISSP---ETG RQC----PAP QTSSRASLSY GYFGNPYYGC RLSHSNLQQK
gi559164131  IPAPDAA--- RQC----PAP PASSNATLGY GYFGGSYYGC RLSHSQQEPC
gi318088606  IFSP---ETG RQC----PAP QTSSSASLSY GYFGNPYYGC RLSHSQQKPC
gi318088610  ISPP---ETG RLC----PAP QTSSSASLTY GYFGNPYYGC RLSHSQQKPC
gi222708677  SEVPGMGEPG RQCSPCSAV- HTSPSASLPY GYFGGSYYQC RMP----KTC
gi222708657  VPPSGMSEPG KQCSPCSA-A QSSSNASLPY GYFGSGYYPC RMSHHTVKPC
gi700431350  XXXXXXXXXX XXXXXXXXXX XXXXXXXXXX XXXXXXXXXX XXXXXXXXXX
gi387231673  XXXXXXXXXX XXXXXXXXXX XXXXXXXVPF GYFGGGYYSC RVSRSSLKSC
gi661918810  ERSGSAARSD PPSKECPGSA APSATPALGY GYFGNGYYSC RMSHNALKSS
gi387231677  XXXXXXXXXX XXXXXXXXXX XXXXXXXVPF GYFGGGYYSC RVSRSSLKPC
gi387231659  XXXXXXXXXX XXXXXXXXXX XXXXXXXVPF GYFGGGYYSC RVSRSSLKPC
gi325260872  ADFPSCSFAP K------PTV FTTSWAPVPY SHHQPHITDA RYVRSWLEPL
gi387231661  XXXXXXXXXX XXXXXXXXXX XXXXXXXXXX GYFGGGYYSC RVSRSSLKPC
gi387231655  XXXXXXXXXX XXXXXXXXXX XXXXXXXXXX XYFGGGYYSC RVSRSSLKPC
gi387231679  XXXXXXXXXX XXXXXXXXXX XXXXXXXXXX XYFGGGYYSC RVSRSSLKPC
gi387231667  XXXXXXXXXX XXXXXXXXXX XXXXXXXXXX XXFGGGYYSC RLSRSSLKPC
gi387231227  XXXXXXXXXX XXXXXXXXXX XXXXXXXXXX XXXXXXXXXX XXXXXXXXXX
gi387231653  XXXXXXXXXX XXXXXXXXXX XXXXXXXXXX XXXGGGYYSC RVSRSSLKPC
gi699575612  XXXXXXXXXX XXXXXXXXXX XXXXXXXXXX XXXXXXXXXX XXXXXXXXXX
gi676248171  XXXXXXXXXX XXXXXXXXXX XXXXXXXXXX XXXXXXXXXX XXXXXXXXXX
gi723145109  XXXXXXXXXX XXXXXXXXXX XXXXXXXXXX XXXXXXXXXX XXXXXXXXXX
gi697016891  XXXXXXXXXX XXXXXXXXXX XXXXXXXXXX XXXXXXXXXX XXXXXXXXXX
gi677147237  XXXXXXXXXX XXXXXXXXXX XXXXXXXXXX XXXXXXXXXX XXXXXXXXXX
gi256014558  ISSPDAA--- RQCQ--APSA AAASNATMGY GYFGGSYYGC RLSHSQQKPC
gi679242416  XXXXXXXXXX XXXXXXXXXX XXXXXXXXXX XXXXXXXXXX XXXXXXXXXX
gi683471095  XXXXXXXXXX XXXXXXXXXX XXXXXXXXXX XXXXXXXXXX XXXXXXXXXX
  gi9438781  XXXXXXXXXX XXXXXXXXXX XXXXXXXXXX XXXXXXXXXX XXXXXXXXXX
gi676827072  XXXXXXXXXX XXXXXXXXXX XXXXXXXXXX XXXXXXXXXX XXXXXXXXXX
gi387231651  XXXXXXXXXX XXXXXXXXXX XXXXXXXXXX XXXXXXXXSC RVSRSSLKSC
gi677498936  XXXXXXXXXX XXXXXXXXXX XXXXXXXXXX XXXXXXXXXX XXXXXXXXXX
gi683474613  XXXXXXXXXX XXXXXXXXXX XXXXXXXXXX XXXXXXXXXX XXXXXXXXXX
gi387231675  XXXXXXXXXX XXXXXXXXXX XXXXXXXXXX XXXXXXXXXX XXXXXXXXPC
gi677248448  XXXXXXXXXX XXXXXXXXXX XXXXXXXXXX XXXXXXXXXX XXXXXXXXXX
gi678138507  XXXXXXXXXX XXXXXXXXXX XXXXXXXXXX XXXXXXXXXX XXXXXXXXXX
gi700349256  XXXXXXXXXX XXXXXXXXXX XXXXXXXXXX XXXXXXXXXX XXXXXXXXXX
gi677225924  XXXXXXXXXX XXXXXXXXXX XXXXXXXXXX XXXXXXXXXX XXXXXXXXXX
gi121308926  XXXPSQSFGA KTCNLPSPYQ SRSASSFM-- ---------- QVATSSERSG
gi194591945  XXXXXXXXXX XXXXXXXXXX XXXXXXXXXX XXXXXXXXXX XXXXXXXXXX
gi195549518  XXXXXXXXXX XXXXXXXXXX XXXXXXXXXX XXXXXXXXXX XXXXXXXXXX
gi194591943  XXXXXXXXXX XXXXXXXXXX XXXXXXXXXX XXXXXXXXXX XXXXXXXXXX
gi387231665  XXXXXXXXXX XXXXXXXXXX XXXXXXXXXX XXXXXXXXSC RVSRSSLKPC
gi195549516  XXXXXXXXXX XXXXXXXXXX XXXXXXXXXX XXXXXXXXXX XXXXXXXXXX
 gi34304667  XXXXXXXXXX XXXXXXXXXX XXXXXXXXXX XXXXXXXXXX XXXXXXXXXX
gi194396133  XXXXXXXXXX XXXXXXXXXX XXXXXXXXXX XXXXXXXXXX XXXXXXXXXX
gi387231669  XXXXXXXXXX XXXXXXXXXX XXXXXXXXXX XXXXXXXXSC RVSRSSLKPC
```

```
gi429510514  XXXXXXXXXX XXXXXXXXXX XXXXXXXXXX XXXXXXXXXX XXSSAAYRDC
gi780155006  XXXXXXXXXX XXXXXXXXXX XXXXXXXXXX XXXXXXXXXX XXXXXXXXXX
gi387231671  XXXXXXXXXX XXXXXXXXXX XXXXXXXXXX XXXXXXXXXX XXXXXXXXPC
gi387231657  XXXXXXXXXX XXXXXXXXXX XXXXXXXXXX XXXXXXXXXX XXXXXXXXXX
gi387231649  XXXXXXXXXX XXXXXXXXXX XXXXXXXXXX XXXXXXXXXX XXXXXXXXXC
gi229298086  XXXXXXXXXX XXXXXXXXXX XXXXXXXXXX XXXXXXXXXX XXXXXXXXXX
gi328682725  XXXXXXXXXX XXXXXXXXXX XXXXXXXXXX XXXXXXXXXX XXXXXXXXXX
gi217035843  XXXXXXXXXX XXXXXXXXXX XXXXXXXXXX XXXXXXXXXX XXXXXXXXXX

             101                                                150
       QUERY AQAATLAAYP AETPTAGEEY PSRPTEFAFY PGYPGTYQPM ASYLDVSVVQ
gi685597136  AQAATLAAYP AETPTAGEEY PSRPTEFAFY PGYPGTYQPM ASYLDVSVVQ
 gi60653709  AQAATLAAYP AETPTAGEEY PSRPTEFAFY PGYPGTYQPM ASYLDVSVVQ
gi795250298  AQAATLAAYP AETPTAGEEY PSRPTEFAFY PGYPGTYQPM ASYLDVSVVQ
gi635093043  AQAATLAAYP AETPTAGEEY PSRPTEFAFY PGYPGTYQPM ASYLDVSVVQ
gi109114206  AQAATLAAYP AETPTAGEEY PSRPTEFAFY PGYPGTYQPM ASYLDVSVVQ
gi795583451  AQAATLAAYP AETPTAGEEY PSRPTEFAFY PGYPGTYQPM ASYLDVSVVQ
gi559812841  AQAATLAAYP AETPTAGEEY PSRPTEFAFY PGYPGTYQPM ASYLDVSVVQ
gi332259422  AQAATLAAYP AETPTAGEEY PSRPTEFAFY PGYPGTYQPM ASYLDVSVVQ
gi795525940  AQAATLAAYP AETPTAGEEY PSRPTEFAFY PGYPGTYQPM ASYLDVSVVQ
gi426237811  AQAATLAAYP AETPASGEEY PSRPTEFAFY PGYPGPYQPM ASYLDVSVVQ
gi744588246  AQGASLAAYP AETPASGEEY ASRPTEFAFY PGYPGPYQPM ASYLDMSVVQ
gi617633383  AQAAPLTSYP AETPVAGEEY PSRPTEFAFY SGYPGPYQPM ASYLDVSVVQ
gi213511801  TQPSALAEKY MDTPVTSEEY PSRAKEFAFY HGYPSPYQSM ASYLDVSVVQ
gi444517714  AQAATLAAYP AETPAAGEEY PSRPTEFAFY PGYPGPYQPM ASYLDVSVVQ
gi640808225  AQAATLAAYP AETPAAGEEY PSRPTEFAFY PGYPGTYQPM ASYLDVSVVQ
gi556958424  TQPAALSSYY MDTPVASEDY QARAKEFAFY HGYASPYQPV ASYLDVSVVQ
gi667260003  AQAATLTAYP AETPAAGEEY PSRPTEFAFY PGYPGPYQPM ASYLDVSVVQ
gi831328207  TQPGALAEKY MDTPVTSEEY PSRAKEFAFY HGYPSPYQSM ASYLDVSVVQ
gi642105600  TQPSALAEKY MDTPVTSEEY PSRAKEFAFY HGYPSPYQSM ASYLDVSVVQ
gi471380475  AQAATLAAYP AETPAAGEEY PSRPTEFAFY PGYPGPYQPM ASYLDVSVVQ
gi674059771  AQTATLAAYP AETPAAGEEY PSRPTEFAFY PGYPGPYQPM ASYLDVSVVQ
gi344285419  AQAATLAAYP AETPAAAEEY PSRPTEFAFY PGYPGPYQPM ASYLDVSVVQ
gi742113362  TQPSALAEKY MDTPVTSEEY PSRAKEFAFY HGYPSPYQSM ASYLDVSVVQ
gi852732630  AQTATLAAYP TETPAAGEEY PSRPTEFAFY PGYPGPYQPM ASYLDVSVVQ
gi512996173  AQAPTLAAYP SETPVAGEEY PSRPTEFAFY PGYPGPYQPM ASYLDVSVVQ
gi395826626  AQAATLAAYP AETAASGEEY PGRPTEFAFY PGYPGTYQPM ASYLDVSVVQ
gi126308251  TQAATLAAYP TETTAAGEEY PSRPAEFAFY PGYPGPYQPV ASYLDVSVVQ
gi528767552  AQGASLAAYP AETPASGEEY ASRPTEFAFY PGYPGPYQPM ASYLDMSVVQ
gi532208489  AQAATLAAYP AETPTAGEEY PSRPTEFAFY PGYPGTYQPM ASYLDVSVVQ
gi507654822  AQAATLAAYP SETPAAGEEY PSRPTEFAFY PGYPGAYQPM ASYLDVSVVQ
gi488527225  AQAATLAAYP AETAAAGEEY PSRPTEFAFY PGYPGPYQPM ASYLDVSVVQ
gi817274366  AQAATLAAYP AETPTAGEEY PSRPTEFAFY PGYPGTYQPM ASYLDVSVVQ
gi585671866  AQAATLTAYP AETPAAGEEY SSRPTEFAFY PGYPGPYQPM ASYLDVSVVQ
gi573901311  TQPGALSSYY MDTPVPSEEY PSRAKEFAFY HGYASPYQPM ASYLDVSVVQ
gi532071900  AQAATLAAYP TETPAAGEEY PSRPTEFAFY PGYPGPYQPM ASYLDVSVVQ
gi114149320  TQPSALAEKY MDTPVTSEEY PTRAKEFAFY HSYPSPYQSM ASYLDVSVVQ
gi593727116  AQAATLAAYP AETPAAGEEY PSRPTEFAFY PGYPGPYQPM ASYLDVSVVQ
gi470606860  AQAATLAAYP AETPAAGEDY PSRPTEFAFY PGYPGPYQPM ASYLDVSVVQ
gi291405863  AQAPSLAAYP AETPAAGEEY PSRPTEFAFY PGYPGPYQPM ASYLDVSVVQ
gi594656397  AQAATLAAYP AETPAAGEEY PSRPTEFAFY PGYPGPYQPM ASYLDVSVVQ
gi634851243  AQAATLAAYP AETPAAGEEY PSRPTEFAFY PGYPGPYQPM ASYLDVSVVQ
gi602709820  AQAATLAAYP AETPAAGEEY PSRPTEFAFY PGYPGPYQPM ASYLDVSVVQ
gi826271801  AQGATLAAYP AETAAAGEEY PGRPTEFAFY PGYPGTYQPM ASYLDVSVVQ
gi589917310  AQTATLATYP SETPAPGEEY TSRPTEFAFY PGYPGPYQPM ASYLDVSVVQ
gi507543189  AQAATLTAYP AETPAPGEEY PSRPTEFAFY PGYPGTYQPM ASYLDVSVVQ
gi395532657  TQAATLAAYP TETTAAGEEY SSRPAEFAFY PGYPGPYQPV ASYLDVSVVQ
gi597740267  TQPSALAEKY MDTPVTSEEY PSRAKEFAFY HGYPSPYQSM ASYLDVSVVQ
gi725576032  AQAGTLAAYP AETPTAGEEY PSRPTEFAFY PGYPGTYQPM ASYLDVSVVQ
gi742135928  AQAATLAAYP AETPTSGEEY PSRPTEFAFY PGYPGPYQPM ASYLDVSVVQ
gi348562271  AQAGTLAAYP SDTPAAGEEY PSRPTEFAFY PGYPGAYQPM ASYLDVSVVQ
gi594050844  AQAATLAAYP AETPTSGEEY PSRPTEFAFY PGYPGPYQPM ASYLDVSVVQ
gi533160875  AQAATLAAYP SETPAAGEEY PSRPTEFAFY PGYPGAYQPM ASYLDVSVVQ
gi556738344  AQAATLAAYP AETPASGEEY PSRPTEFAFY PGYPGPYQPM ASYLDVSVVQ
gi803246204  AQAATLAAYP AETPASGEEY PSRPTEFAFY PGYPGPYQPM ASYLDVSVVQ
gi354474760  AQTATLATYP SETPAPGEEY PGRPTEFAFY PGYPGPYQPM ASYLDVSVVQ
gi532051321  AQTATLATYP SETPASGEEY PSRPTEFAFY PGYPGPYQPM ASYLDVSVVQ
gi311267502  AQGATLAAYP AETPTAGEEY PSRPTEFAFY PGYPGPYQPM ASYLDVSVVQ
gi743729506  AQGASLAAYP AETPTSGEEY ASRPTEFAFY PGYPGPYQPM ASYLDMSVVQ
```

gi359754103	TQAATLAAYP	PETTAAGEDY	PSRPAEFAFY	PGYPGPYQPV	ASYLDVSVVQ
gi576886849	TQPSALAEKY	MDTPVTSEEY	PTRAKEFAFY	HGYHGPYQSM	ASYLDVSVVQ
gi586448761	AQGTTLAAYP	PDTPSAGEEY	PSRPTEFAFY	PGYPGPYQPM	ASYLDVSVVQ
gi504159820	AQAPSLTAYP	AETPAAGEEY	PSRPAEFAFY	PGYPGPYQPV	ASYLDVSVVQ
gi524951862	AQTATLATYP	SETPAPGEEY	PSRPTEFAFY	PGYPGPYQPM	ASYLDVSVVQ
gi332692483	TQPSPLAEKY	MDTPVTSEEY	SNRTKEFAFY	HGYPSPYQPV	ASYLDVSVVQ
gi385654474	TQPSPLAEKY	MDTPVTSEEY	SNRTKEFAFY	HGYPSPYQPV	ASYLDVSVVQ
gi478520959	APAATLAAYP	TETPAAGEEY	PSRPTEFAFY	PGYPGPYQPM	ASYLDVSVVQ
gi301128881	TQPAALSSYY	MDSPVPTDDY	PGRPKEFAFY	HGYASAYQPM	PSYLDVSVVQ
gi1011609041	PQSSYPAEKY	VDTPAAGEEF	QPRPTDFAFY	PGYAGPYQPM	ASYLDVSVVQ
gi664747354	AQAATLTAYP	TETPATGEEY	PSRPTEFAFY	PGYPGPYQPM	ASYLDVSVVQ
gi557280818	PQSSYPAEKY	VDTPAAGEEF	QPRPTDFAFY	PGYAGPYQPM	ASYLDVSVVQ
gi359076815	AQAATLAAYP	AETPTSGEEY	PSRPTEFAFY	PGYPGPYQPM	ASYLDVSVVQ
gi632972216	TQPAALGDKY	MDTPVPTDDY	PGRAKEFAFY	HGYAGPYQPM	PSYLDVSVVQ
gi759185571	AQAAALTAYP	AETPTAGEEY	PSRPTEFAFY	PGYPGPYQPM	ASYLDVSVVQ
gi472350967	AQAATLAAYP	AETATAGEEY	PGRPTEFAFY	PGYPGPYQPM	ASYLDVSVVQ
gi586539979	AQAATLTAYP	AETPTAGEEY	PSRPTEFAFY	PGYPGPYQPM	ASYLDVSVVQ
gi157823841	AQTATLATYP	SETPAPGEEY	PSRPTEFAFY	PGYPGPYQPM	ASYLDVSVVQ
gi410980861	AQAATLAAYP	AETAAAGEEY	PSRPTEFAFY	PGYSGPYQPM	ASYLDVSVVQ
gi829772881	AQAATLAAYP	AETAAAAGEY	PGRPTEFAFY	PGYPGTYQPM	ASYLDVSVVQ
gi220898176	AQPSTFAEKY	MDTSVSGEEF	TSRAKEFAFY	QGYAGPYQPV	PSYLDVPVVP
gi1519038	AQTAALATYP	SETPAPGEEY	PSRPTEFAFY	PGYPGPYQPM	ASYLDVSVVQ
gi731207261	AQAAALAAYP	SEPPAAGEEY	PSRPTEFAFY	PGYPGAYQPM	ASYLDVSVVQ
gi20278975	AQPSTLSGYP	ADKTSAGEEF	TSRAKEFAFY	QGYAGPYQPM	PSYLDVPVVP
gi585176637	AQAAPLAAYS	AETATAGEQY	PSRPTEFAFY	PGYPGPYQPM	ASYLDVSVVQ
gi560984071	AQGASLAAYP	AETPASGEEY	ASRPTEFAFY	PGYPGPYQPM	ASYLDMSVVQ
gi73966255	AQAATLAAYP	AETATAGEEY	PSRPTEFAFY	PGYPGPYQPM	ASYLDVSVVQ
gi511890491	AQAATLAAYP	AETAAPGEEY	PSRPTEFAFY	PGYPGPYQPM	ASYLDVSVVQ
gi254212171	AQPSTLSGYP	ADKTSAGEEF	TTRAKEFAFY	QGYAGPYQPM	PSYLDVPVVP
gi558229251	AQPSTFADKY	MDTSVSGEEF	TSRAKELAFY	PGYAGPYQPV	PSYLDMPVVP
gi752394348	AQA--LAAYP	AEPATAGEEY	PSRPTEFAFY	PGYPGPYQPM	ASYLDVSVVQ
gi507952779	AQAATLAAYP	AEAPAAGEEY	PSRPTEFAFY	PGYPGPYQPV	ASYLDVSVVQ
gi507664676	AQAATLAAYP	AETPAPGEEY	PSRATEFAFY	PGYPGPYQPM	ASYLDVSVVP
gi224045310	AQPSTFADKY	MDTSVSGEEF	TSRAKEFAFY	QGYAGPYQPV	PGYLDMPVVP
gi400180333	AQPSAYAEKY	MDTSGSGEEY	TSRAKEFAFY	QGYASPYQPV	PSYIDVPVVP
gi641724374	AQAAALAAYP	AEPPAAAEDY	PGRHAEFAFY	PGYPAPYQPM	ASYLDVSVVQ
gi693302455	AQPSTFADKY	MDTSVAGEEF	GSRAKEFAFY	QGYAAPYQAV	PGYLDMPVVP
gi542179924	TQAAALSSQY	MDTTVSSDEY	PNRAKEFAFY	HGYASPYQSM	ASYLDVSVVQ
gi119370791	TQAAALSSQY	MDTTVNSDEY	SNRAKEFAFY	HSYPSPYQSM	ASYLDVSVVQ
gi678126544	AQSSAYGEKY	MDTSVSGEDF	SSRAKEFAFY	PGYPGPYQPV	PGYLDVPVVS
gi808884419	TQAAALSSQY	MDTTVNSDEY	SNRAKEFAFY	HGYPSPYQSM	ASYLDVSVVQ
gi505802773	APAPTLATYP	TEAPVAGEEY	PSRPTEFAFY	PGYPGPYQPM	ASYLDVSVVQ
gi34484442	TQAAALSSQY	MDTTVNSDDY	SNRAKEFAFY	HSYPSPYQSM	ASYLDVSVVQ
gi519777295	AQPSSFADKY	MDTSSAGEEF	TSRAKEFAFY	QGYAGPYQPV	PGYLDMPMVP
gi657803392	TQAAALSSQY	MDTTVNADEY	SNRAKEFAFY	HGYPSPYQSM	ASYLDVSVVQ
gi554874539	TQAAALSSQY	MDTTVSSDEY	PNRAKEFAFY	HGYASPYQSM	ASYLDVSVVQ
gi499036069	TQAAALSSQY	MDTTVSSDEY	PNRAKEFAFY	HGYASPYQSM	ASYLDVSVVQ
gi301607689	SQPSSFAEKY	MDTSSAGEDF	PSRPKEFAFY	QSYPGPYQPV	PSYLDMPVVS
gi584023332	TQAAALSSQY	MDTTVSSDEY	PNRAKEFAFY	HGYASPYQSM	ASYLDVSVVQ
gi548549273	TQAAALSSQY	MDTTVSSDEY	PNRAKEFAFY	HGYASPYQSM	ASYLDVSVVQ
gi657574876	TQAAALSSQY	MDTTVSSDEY	PNRAKEFAFY	HGYPSPYQSM	ASYLDVSVVQ
gi28268588	SQPSSFADKY	MDTSSAGKDF	PSRPKEFAFY	QSYPGPYQPV	PSYLDMPVVS
gi736251858	TQAAALSSQY	MDTTVNPDDY	PNRAKEFAFY	HGYPSPYQSM	ASYLDVSVVQ
gi530624973	PQSSYSADKY	MDTPAAADDY	QTRPTELAFY	PGYAGPYQPR	ASYLDVSVVQ
gi290760363	AQPSSFADKY	METSVSSEDF	PSRAKEFAFY	QGYAGPYQPM	PGYLDMPVVP
gi551520979	TQAAALSSQY	MDTTVSPDEY	PNRAKEFTFY	HGYANPYQSM	GSYLDVSVVQ
gi658861163	TQAAALSSQY	MDTTVNPDEY	PNRAKEFTFY	HGYANPYQSM	ASYLDVSVVQ
gi617509429	TQAAALSSQY	MDTTVNPDEY	PNRAKEFTFY	HGYANPYQSM	ASYLDVSVVQ
gi47229429	AAPSAYGEKY	MDTSATSDDF	PSRTKEFTFY	PTYPGPYQPV	PSYLDVPVVP
gi831555258	AQPSAYGEKY	MDTAASGEEF	SSRAKEFAFY	PTYPGPYQPV	PSYLDVPVVP
gi591374792	PQSSYSAEKY	VDTPAAADDY	QTRPTELAFY	PGYAGPYQPM	ASYLDVSVVQ
gi432871359	TQAAALSSQY	MDTTVSPDEY	PNHAKEFAFY	HGYPSPYQSM	ASYLDVSVVQ
gi602643660	AQPSSFADKY	METSVAGEDF	PSRAKEFAFY	QGYAGPYQPV	PGYLDMPVVP
gi961915500	AQPSGYGEKY	ADSSVSGE--	---AKEFAFY	QGYSSPYQ--	ASYLDVPVVP
gi961860111	AQPSGYGEKY	ADSSVSGE--	---AKEFAFY	QGYSSPYQT-	-SYLDVPVVP
gi219879168	TQPTSYGEKY	MDTSVSGEEF	PSRAKEFAFY	QGYSGPYQPV	PSYLDVPVVP
gi219879156	TQPTSYGEKY	MDTSVSGEEF	PSRAKEFAFY	QGYSGPYQPV	PSYLDVPVVP
gi219879164	TQPSSYGEKY	MDTSVSGEEF	PSRAKEFAFY	QGYSGPYQPV	ASYLDVPVVP
gi123204435	TQPTSYGEKY	MDTSVSGEEF	SSRAKEFAFY	QGYSGPYQPV	PSYLDVPVVP

gi465985114	AQPASAADKY	MDTAPAAEEF	SSRAKEFAFY	QGYPGPYQPV	PGYLDMPVVP
gi426355784	AAAAAFADKY	MDTAPAAEEF	SSRAKEFAFY	QGYAGPYQPM	PGYLDMPVVP
gi1034052196	AAAAAFADKY	MDTAPAAEEF	SSRAKEFAFY	QGYAGPYQPM	PGYLDMPVVP
gi683901043	AQPSTFADKY	MDTSVSGEEF	TSRAKEFAFY	QGYAGPYQPV	PGYLDMPVVP
gi729725749	PQGPFPTEKY	LDPPAGSEDF	QSRPAEFAFY	PGYGAPYQPV	AGYLDVSVVP
gi663243102	AQGPFPTEKY	LDPSGATEDF	QSRPAEFAFY	PGYGAPYQPV	AGYLDVSVVP
gi339792792	AQPSSFADKY	MDTSSAGDEF	TSRAKEFAFY	QGYAGPYQPV	PGYLDMPMVP
gi768393729	PQGPFPTEKY	LDPPAGSEDF	QSRPAEFAFY	PGYGAPYQPV	AGYLDVSVVP
gi670977729	AQAASLAAYP	AEPATAWEEY	PSRPTEFAFY	PGYPGPYQPM	ASYLDVSVVQ
gi327263844	SYHPAAAEKY	PPEPSPSEEL	SSRAKEFAFY	PSFASSYQAV	PGYLDVSVVP
gi1040268871	AYHPGDKYPE	PSGALPGDDL	SSRAKEFAFY	PSFASSYQAM	PGYLDVSVVP
gi303303637	AYHSGDKYPE	PSGALPGDDL	SSRAKEFAFY	PSFASSYQAM	PGYLDVSVVP
gi724877051	AYHPGDKYPE	PSGALPGDDL	SSRAKEFAFY	PSFASSYQAM	PGYLDVSVVP
gi544470246	AYHPGDKYPE	PSGALPGDDL	SSRAKEFAFY	PSFASSYQAM	PGYLDVSVVP
gi338726307	AYHPGDKYPE	PSGALPGDDL	SSRAKEFAFY	PSFASSYQAM	PGYLDVSVVP
gi297692036	AYHPGDKYPE	PSGALPGDDL	SSRAKEFAFY	PSFASSYQAM	PGYLDVSVVP
gi397521957	AYHPGDKYPE	PSGALPGDDL	SSRAKEFAFY	PSFASSYQAM	PGYLDVSVVP
gi1018974175	AQPSAAADKY	MDTPAAADDF	GSRAKEFAFY	QGYAGPYQPV	PGYLEMPVVP
gi675430530	PHASFPVEKY	MDTSVPANEV	SSRAKEVSFY	QGYTTPYQHV	PGYIDM--VS
gi795174618	PHASFPVEKY	MDSSVPANEV	PARAKEVSFY	QGYTSPYQHV	PGYIDM--VS
gi699633156	PHASFPVEKY	MDTSVPANEV	SSRAKEVSFY	QGYTTPYQHV	PGYIDM--VS
gi374498883	PHASFPVEKY	MDTSVPANEV	SSRAKEVSFY	QGYTTPYQHV	PGYIDM--VS
gi24637241	THNVNLQQKP	CSYQEPAEDL	PSRAKEFAFY	PSFSGSYQTV	PGYLDMSVVP
gi584059997	XXPATLAAYP	AEPPAAGEDY	PSRHAEFAFY	PGYPAPYQPM	ASYLDVSVVQ
gi884764069	AQAPF-GEKY	LEPPAGAEDF	QSRPAEFAFY	PGYGAPYQPV	AGYLDVSVVP
gi66773958	PH-SSLGGFP	VESSVPANEV	PARAKEVSFY	QGYTSPYQHV	PGYIDM--VS
gi224016307	PHASFPVEKY	MDSSVPANEV	PARAKEVSFY	QGYTSPYQHV	PGYIDM--VS
gi224016295	PHASFPVEKY	MDSSVPANEV	PARAKEVSFY	QGYTSPYQHV	PGYIDM--VS
gi224016303	PHASFPVEKY	MDSSVPANEV	PARAKEVSFY	QGYTSPYQHV	PGYIDM--VS
gi224016309	PHASFPVEKY	MDSSVPANEV	PARAKEVSFY	QGYTSPYQHV	PGYIDM--VS
gi374498861	PHASFPVEKY	MDTSVPANEV	SSRAKEVSFY	QGYPSPYQHV	PGYIDM--VS
gi224016313	PHASFPVEKY	MDSSVPANEV	PARAKEVSFY	QGYTSPYQHV	PGYIDM--VS
gi224016305	PHASFPVEKY	MDSSVPANEV	PARAKEVSFY	QGYTSPYQHV	PGYIDM--VS
gi224016311	PHASFPVEKY	MDSSVPANEV	PARAKEVSFY	QGYTSPYQHV	PGYIDM--VS
gi224016299	PHASFPVEKY	MDSSVPANEV	PARAKEVSFY	QGYTSPYQHV	PGYIDM--VS
gi700384804	PHASFPVEKY	MDTSVPANEV	SSRAKEVSFY	QGYTTPYQHV	PGYIDM--VS
gi543378707	PQGPFPNDKY	LEAASGSEDF	QSRPAEFAFY	PGYGAPYQPV	PGYLDVAVVP
gi699659589	PQGPFPGEKY	LDTPAGSEDF	QSRPTEFTFY	PGYGAPYQPV	TGYLDVSVVP
gi310656724	PPHASLGGFA	MESTAPGNEG	ASRAKEVSFY	QGYPNPYQHV	PGYLDM--VS
gi565321354	XXXXXXXXXX	METSVSSEDF	PSRAKEFAFY	QGYAGPYQPM	AGYLDMPVVP
gi290760375	PHAAFPGEKY	MDVPSAGDPV	SSRAKEVSFY	QGYTGPYQHV	PGYLDVVSTF
gi696966138	XXXXXXXXXX	XXTSVPANEV	SSRAKEVSFY	QGYTTPYQHV	PGYIDM--VS
gi677975869	TQGSFPAEKY	LEPPAGSEDF	QSRPAEFAFY	PSYGAPYQPV	AGYLDVSVVP
gi543746989	XXXXXXXXXX	XXTSVPANEV	SSRAKEVSFY	QGYTTPYQHV	PGYIDM--VS
gi701389587	XXXXXXXEKY	MDTSVPANEV	SSRAKEVSFY	QGYTTPYQHV	PGYIDM--VS
gi256014533	AYHPAEKYPE	PSGSLPNEEL	SSRAKEFAFY	PSFASSYQAV	PGYLDVSVVP
gi669281149	XXXXXXXXXX	XXXXXPANEV	SSRAKEVSFY	QGYTTPYQHV	PGYIDM--VS
gi118102888	AQGPFPPEKY	LDPPAGSEDF	QSRPAELAFY	PGYGAPYQPV	AGYLDVSVVP
gi554547429	----------	----------	------FAFY	PGYPAPYQPM	ASYLDVSVVQ
gi694854743	AYHPGEKYPE	AGGPLPGEEL	PTRAQEFAFY	PGFPSSYQAV	PGYLDVSVVP
gi529426764	AYHPGEKYPE	AGGPLPGEEL	PSRAKEFAFY	PGFPSSYQAV	PGYLDVSVVP
gi706150961	XXXXXXXXXX	XXXXAGSEDF	QSRPAEFAFY	PGYGAPYQPV	AGYLDVSVVP
gi719780455	XXXXXXXXXX	AKAPAGGEDS	QSPPAELAFY	PSYGAPYQPV	ASYLDVSVVQ
gi290760370	PHAAFPVEKY	MDSVPSGSDV	SSR----AFY	QGYAGPYQHV	PGYLDVVSTF
gi328682541	AHASVGGEKY	VDDSVTGNDV	SPRTKGLAFY	QGYPGPYPRV	PGYIDM--VP
gi541961056	AYHPGEKYPE	AGGPLPGEEL	PSRAKEFAFY	PGFPSSYQAV	PGYLDVSVVP
gi733928584	XXXXPSEKFT	PNSTLPGEEL	PSRPKEFAFY	PGFASSYQAV	PGYLDVSVVP
gi686600796	AYHPGEKYPE	AGGPLPGEEL	PSRAKEFAFY	PGFPSSYQAV	PGYLDVSVVP
gi704535956	XXXXXXXXXX	XXXXXXSEDF	QSRPAEFAFY	PSYGAPYQPV	AGYLDVSVVP
gi555983692	XXXXXXXXXX	XTAGPAAEEF	SSRAKEFAFY	QGYPGPYQPV	PGYLDMPVVP
gi591300470	XXXXXXXXXX	MARGPAAEEF	SSRAKEFAFY	QGYPGPYQPV	PGYLDMPVVP
gi255683553	AQPSTFADKY	MDTSVSGEEF	TSRAKDWAML	PGPTTRARVF	GYACGAHNWR
gi255683559	AQPSTFADKY	MDTSVSGEEF	TSRAKDWAML	PGPTNRARVF	GYACGAHNWR
gi558170872	XQAATLAAYP	AEPPAAGEDY	PSRPRRSSF-	PGGTAPYQAY	GQVFDVSVVQ
gi255683557	AQPSTFADKY	MDTSVSGEEF	TSRAKDWAML	PGPTTRARVF	GYACGAHNWR
gi874491859	XXGPFPPEKY	LDPPAGSEDF	QSRPAEFAFY	PSYGAPYQPV	AGYLD-----
gi726998761	XXXXXXXXXX	XXXXXGGEDL	PARAKEFAFY	PGFPGSYQAV	PGYLDVSVVP
gi697489159	XXXXXXXXXX	XXXPLPSEEL	PSRAKEFAFY	PGFASSYQTV	PGYLDMSVVP
gi525030647	XXXXXXXXXX	XXXXXXXXXX	XXXXXXXAFY	PGFPGSYQAV	PGYLDVSVVP

```
gi222159495  AQPSPYGDKY MDTSVSGEEF TSRAKEFAFY QGYSGPYQPV SSYLDVPAVP
gi187936703  XXXXXXXXXX XXXXXXXDDY TSAAKDFAFY PSYGSAYHHM PGYLDMPVVP
gi690457118  AYHPGEKYPE AGGPLPGEEL PSRAKEFAFY PGFPSSYQAV PGYLDVSV--
gi559163908  AQPASFADKY MDTSGAGEDF TSRAKEFAFY QGYAGPYQPV PGYLDMPVVP
gi559163610  AQPSSFADKY MDTSVAGEDF TSRAKEFAFY QGYAGPYQPV PGYLDMPVVP
gi559163543  AQPASLADKY MDTSVAGEDF TSRAKEFAFY QGYAGPYQPV PGYLDMPVVP
gi704260001  AYHPGEKYPE AGGPLPGEEL PSRAKEFAFY PGFPSSYQAV PGYLDVSVVP
gi559163971  AQPSSFADKY MDTSVAGEDF TSRAKEFAFY PGYAGPYQPV PGYLDMPVVP
gi559163730  AQPASFADKY MDTSVAGEDF TARAKEFAFY QGYAGPYQPV PGYLDMPVVP
gi269204564  AQPASFADKY MDTSVAGEDF TSRAKEFAFY QGYAGPYQPV PGYLDMPVVP
gi222159485  SQPSSYGEKY MDTSVSGEEF TSRAKEFAFY QGYAGPYQPV PSYLDVPVVP
gi559164035  AQPASFADKY MDTSVAGEDF TSRAKEFAFY QGYAGPYQPV PGYLDMPVVP
gi222708647  AQPSTYAEKY MDTSVSGEEF TSRAKEFAFY QGYAGPYQPV PSYLDVPVVP
gi290760385  PHASLSGEKY MDTSGPANEV SSRAKEVSFY QGYPNPYQHV PGYIDM--VS
gi559163786  AQPSSFADKY METSVSSEDF PSRAKEFAFY QGYAGPYQPM PSYLDMPVVP
gi559163846  SQPSSFADKY MDTSAASEDF PSRAKEFAFY QGYAGPYQPM PGYLDVPVVP
gi318088612  SYHPAEKYAE TSSALPAEEL SSRAKEFAFY PSFASSYQAV PGYLDMSVVP
gi318088602  SYHPAEKYAE TSSALPAEEL SSRAKEFAFY PSFASSYQTV PGYLDMSVVP
gi222708673  AQPSPYGEKY MDTSVSGEEF SSRAKEFAFY QGYSGPYQPV PSYLDVPVVP
gi222708649  AQPASYAEKY MDTSGSGEDF TSRAKEFAFY QGYAGPYQPV PSYLDVPVVP
gi222708671  AQPSSYGEKY MDTSISGEEF PSRAKEYTFY QGYPGPYQPV PSYLDMPVVP
gi222708655  AQPSSYAEKY METSVSGEEF TSRAKEFAFY QGYTSPYQPV PSYLDMPVVP
gi222708681  TQPTSYGEKY MDTSVSGEEF PSRAKEFAFY QGYSGPYQPV PSYLDVNVVP
gi318088600  SYHPAEKYAE TSSALPAEEL SSRAKEFAFY PSFASSYQTV PGYLDMSVVP
gi318088598  SYHPAEKYAE TSSALPAEEL SSRAKEFAFY PSFASSYQAV PGYLDMSVVP
gi222708679  TQPTSYGEKY MDTSVSGEEF PSRAKEFAFY QGYSGPYQPV PSYLDVPVVP
gi318088608  HGSYNPAEKY AETSSPAEEL SSRAKEFAFY PSFASSYQAV PGYLDMSVVP
gi559164131  SYHPAEKYPE PSGSLPSEEL STRAKEFAFY PSFASSYQAV PGYLDVSVVP
gi318088606  SSHPAEKYAE TSSALPAEEL SSRAKQFAFY PSFARSYQAV PGYLDMSVLP
gi318088610  SYHPAEKYAE TSSALPAEEL SGRAKEFAFY PSFASSYQAV PGYLDMSLVP
gi222708677  TQPSSYGEKY MDTSVSGEDF PSRAKEFAFY QGYSGSYQPV PSYLDVPVVP
gi222708657  AQPS-YADKY MDTSVPGEDF PSRPKEFAFY QGYPGPYQPV PSYLDMPVVP
gi700431350  XXXXXXXXXX XXXXXXXXXX XXXXXXXXXX XXXXXXXXXX XXXXXXXXXX
gi387231673  AQAATLTAYP AETPSAGEEY PSRPTEFAFY PGYPGSYQPM ASYLDVSVVQ
gi661918810  PHASFPVEKY MDTSVPANEV SSRAKEVSLY QGYTNPYQHV PGYIDM--VS
gi387231677  AQAATLAAYP TETPAAGEEY PSRPTEFAFY PGYPGPYQPV ASYLDVSVVQ
gi387231659  AQAATLAAYP TETPAAGEEY PSRPTEFAFY PGYPGPYQPV ASYLDVSVVQ
gi325260872  SGAVSFPSFA ATSRHYGDSF PGRRGECG-- PGDPRSY--- PDYM------
gi387231661  AQAATLAAYP TETPAAGEEY PSRPTEFAFY PGYPGPYQPV ASYLDVSVVQ
gi387231655  AQAATLAAYP TETPAAGEEY PSRPTEFAFY PGYPGPYQPV ASYLDVSVVQ
gi387231679  AQAATLAAYP TETPAAGEEY PSRPTEFAFY PGYPGPYQPV ASYLDVSVVQ
gi387231667  AQAATLAAYP AETPGAGEEY PSRHTEFAFY PGYPGPYQPV ASYLDVSVVQ
gi387231227  XXXXXXXXXX XXXXXXXXXX XXXXXXXXXX XXXXXXXXXX XXXXXXXXXX
gi387231653  AQAATLAAYP TETPAAGEEY PSRPTEFAFY PGYPGPYQPV ASYLDVSVVQ
gi699575612  XXXXXXXXXX XXXXXXXXXX XXXXXXXXXX XXXXXXXXXX XXXXXXXXXX
gi676248171  XXXXXXXXXX XXXXXXXXXX XXXXXXXXXX XXXXXXXXXX XXXXXXXXXX
gi723145109  XXXXXXXXXX XXXXXXXXXX XXXXXXXXXX XXXXXXXXXX XXXXXXXXXX
gi697016891  XXXXXXXXXX XXXXXXXXXX XXXXXXXXXX XXXXXXXXXX XXXXXXXXXX
gi677147237  XXXXXXXXXX XXXXXXXXXX XXXXXXXXXX XXXXXXXXXX XXXXXXXXXX
gi256014558  SYHPAEKYPE PSGSLPGEEL SSRAKEFAFY PSFTGSYQAV PGYLDVSVAP
gi679242416  XXXXXXXXXX XXXXXXXXXX XXXXXXXXXX XXXXXXXXXX XXXXXXXXXX
gi683471095  XXXXXXXXXX XXXXXXXXXX XXXXXXXXXX XXXXXXXXXX XXXXXXXXXX
  gi9438781  XXXXXXXXXX XXXXXXXXXX XXXXXXXXXX XXXXXXXXXX XXXXXXXXXX
gi676827072  XXXXXXXXXX XXXXXXXXXX XXXXXXXXXX XXXXXXXXXX XXXXXXXXXX
gi387231651  AQAATLSAYP AETPPAGEEY PSRPTEFAFY PGYPGSYQPM ASYLDVSVVQ
gi677498936  XXXXXXXXXX XXXXXXXXXX XXXXXXXXXX XXXXXXXXXX XXXXXXXXXX
gi683474613  XXXXXXXXXX XXXXXXXXXX XXXXXXXXXX XXXXXXXXXX XXXXXXXXXX
gi387231675  AQAATLAAYP TETPAAGEEY PSRPTEFAFY PGYPGSYQPA TSYLDVSVVQ
gi677248448  XXXXXXXXXX XXXXXXXXXX XXXXXXXXXX XXXXXXXXXX XXXXXXXXXX
gi678138507  XXXXXXXXXX XXXXXXXXXX XXXXXXXXXX XXXXXXXXXX XXXXXXXXXX
gi700349256  XXXXXXXXXX XXXXXXXXXX XXXXXXXXXX XXXXXXXXXX XXXXXXXXXX
gi677225924  XXXXXXXXXX XXXXXXXXXX XXXXXXXXXX XXXXXXXXXX XXXXXXXXXX
gi121308926  PYHASFPYPR TDCRAPNEDH PKRPSSLQFN PSLPSPMERY PSSMSSAAAM
gi194591945  XXXXXXXXXX XXXXXXXXXX XXXXXXXXXX XXXXXXXXXX XXXXXXXXXX
gi195549518  XXXXXXXXXX XXXXXXXXXX XXXXXXXXXX XXXXXXXXXX XXXXXXXXXX
gi194591943  XXXXXXXXXX XXXXXXXXXX XXXXXXXXXX XXXXXXXXXX XXXXXXXXXX
gi387231665  AQAATLTAYP AETP-AGDEY PSRPTEFAFY PGYPGPYQPM ASYLDVSVVQ
gi195549516  XXXXXXXXXX XXXXXXXXXX XXXXXXXXXX XXXXXXXXXX XXXXXXXXXX
 gi34304667  XXXXXXXXXX XXXXXXXXXX XXXXXXXXXX XXXXXXXXXX XXXXXXXXXX
```

```
gi194396133  XXXXXXXXXX XXXXXXXXXX XXXXXXXXXX XXXXXXXXXX XXXXXXXXXX
gi387231669  TQAATLAAYP TETPAAGEEY PSRPTEFAFY PGYPGSYQPV ASYLDVSVVQ
gi429510514  LSAAMLMKSG ETAAYSQARY GSANTSYNFY GGKVGRTGVL PQAFDQFLLE
gi780155006  XXXXXXXXXX XXXXXXXXXX XXXXXXXXXX XXXXXXXXXX XXXXXXXXXX
gi387231671  AQAATLAAYP TETPAAGEEY PSRPTEFAFY PGYPGPYQPV ASYLDVSVVQ
gi387231657  XXXXXXXXYP TETPAAGEEY PSRPTEFAFY PGYPGSYQPA TSYLDVSVVQ
gi387231649  AQAATLAAYP TETLAAGEEY PSRPTEFAFY PGYPGSYQPA TSYLDVSVVQ
gi229298086  XXXXXXXXXX XXXXXXXXXX XXXXXXXXXX XXXXXXXXXX XXXXXXXXXX
gi328682725  XXXXXXXXXX XXXXXXXXXX XXXXXXXXXX XXXXXXXXXX XXXXXXXXXX
gi217035843  XXXXXXXXXX XXXXXXXXXX XXXXXXXXXX XXXXXXXXXX XXXXXXXXXX

             151                                                200
       QUERY TLGAPGEPRH DSLLPVDSYQ SWALAGGWNS QMCCQGEQNP PGPFWKAAFA
gi685597136  TLGAPGEPRH DSLLPVDSYQ SWALAGGWNS QMCCQGEQNP PGPFWKAAFA
 gi60653709  TLGAPGEPRH DSLLPVDSYQ SWALAGGWNS QMCCQGEQNP PGPFWKAAFA
gi795250298  TLGAPGEPRH DSLLPVDSYQ SWALAGGWNS QMCCQGEQNP PGPFWKAAFA
gi635093043  TLGAPGEPRH DSLLPVDSYQ SWALAGGWNS QMCCQGEQNP PGPFWKAAFA
gi109114206  TLGTPGEPRH DSLLPVDSYQ SWALAGGWNS QMCCQGEQNP PGPFWKAAFA
gi795583451  TLGAPGEPRH DSLLPVDSYQ SWALAGGWNS QMCCQGEQNP PGPFWKAAFA
gi559812841  TLGAPGEPRH DSLLPVDSYQ SWALAGGWNS QMCCQGEQNP PGPFWKAAFA
gi332259422  TLGAPGEPRH DSLLPVDSYQ SWALAGGWNS QMCCQGEQNP PGPFWKAAFA
gi795525940  TLGAPGEPRH DSLLPVDSYQ SWALAGGWNS QMCCQGEQNP PGPFWKAAFA
gi426237811  TLGAPGEPRH DSLLPVDSYQ PWALTGGWNS QMCCQGEQNP PGPFWKAAFA
gi744588246  TLGGPGEPRH DSLLPVDSYQ PWALTGGWNS QMCCQGEQNP PGPFWKAAFA
gi617633383  TLGAPGEPRH DSLLPVDTYQ PWALTNGWNS QMCCQGEQNP PGPFWKAAFA
gi213511801  TLGA-GEPRH DTLLPMDSYQ PWALTNGWGG QMYCSKDQSQ AGHLWKSALA
gi444517714  TLGAPGEPRH DSLLPVDGYQ PWALTGGWNS QMCCQGEQNP PGPFWKATFA
gi640808225  TLGAPGEPRH DSLLPVDSYQ SWALAGGWNS QMCCQGEQNP PGPFWKAAFA
gi556958424  TISGAGEPRH ETLLPVDSYQ PWALTNGWNS QMYCSKDQTQ PGHLWKSALA
gi667260003  TLGAPGEPRH DSLLPMDSYQ PWALAGGWNS QMCCQGEQNP PGPFWKAAFA
gi831328207  TLGT-GEPRH DTLLPMDSYQ PWALTNGWGG QMYCSKDQSQ AGHLWKSALA
gi642105600  TLGS-GEPRH DTLLPMDSYQ PWALTNGWGG QMYCSKEQSQ AGHLWKSALA
gi471380475  TLGAPGEPRH DSLLPVDSYQ PWALAGGWNS QMCCQGEQNP PGPFWKAAFA
gi674059771  TLGTPGEPRH DSLLPVDSYQ PWALAGGWNS QMCCQGEQNP PGHFWKAAFA
gi344285419  TLGGPGEPRH DSLLPVDSYQ PWALAGGWNS QMCCQGEQNA PGPFWKAAFA
gi742113362  TLGA-GEPRH DTLLPMDSYQ PWALTNGWGG QMYCSKDQSQ AGQLWKSALA
gi852732630  TLGAPGEPRH DSLLPVDSYQ PWALAGGWNS QMCCQGEQNP PGPFWKAAFS
gi512996173  TLGAPGEPRH DSLLPVDGYQ PWALAGGWNS QMCCQGEQNP PGPFWKAAFA
gi395826626  TLGAPGEPRH DSLLPVDSYQ SWALAGGWNS QMCCQGEQNP PGPFWKAAFA
gi126308251  TLGGPGEPRH DTLLPVDSYQ PWALASGWSG QMCCQGEQNQ PGHFWKTAFA
gi528767552  TLGGPGEPRH DSLLPVDSYQ PWALTGGWNS QMCCQGEQNP PGPFWKAAFA
gi532208489  TLGASGEPRH DSLLPVDSYQ SWALAGGWNS QMCCQGEQNP PGPFWKAAFT
gi507654822  TLGAPGEPRH DSLLPVDSYQ PWALAGGWNS QMCCQGEQNP PGPFWKAAFA
gi488527225  TLGTPGEPRH DTLLPVDSYQ PWALAGGWNS QMCCQGEQNP PGPFWKATFA
gi817274366  TLGAPGEPRH DSLLPVDSYQ SWALAGGWNS QMCCQGEQNP PGPFWKAAFT
gi585671866  TLGAPGEPRH DSLLPVDSYQ PWALAGGWNS QMCCQGDQNP PGPFWKAAFA
gi573901311  TISGPGETRH ETLLPVDSYQ PWALPNGWGS QVYCPKDQSQ AGHLWKSALA
gi532071900  TLGAPGEPRH DSLLPVDSYQ PWALASGWNS QMCCQGEQNP PGPFWKAAFA
gi114149320  TLGT-GEPRH DSLLPMDSYQ PWALANGWGS QMYCSKDQGQ AGHLWKSALA
gi593727116  TLGAPGEPRH DSLLPVDSYQ PWALTGGWNS QMCCQGEQNP PGPFWKAAFA
gi470606860  TLGAPGEPRH DSLLPVDSYQ PWALTGGWNS QMCCQGEQNP PGPFWKAAFA
gi291405863  TLGAPGEPRH DSLLPVDSYQ PWALAGGWNS QMCCQGEQNP PGPFWKAAFA
gi594656397  TLGAPGEPRH DSLLPVDSYQ PWALTGGWNS QMCCQGEQNP PGPFWKAAFA
gi634851243  TLGAPGEPRH DSLLPVDSYQ PWALAGSWNS QMCCQGEQNQ PGPFWKAAFA
gi602709820  TLGAPGEPRH DSLLPVDSYQ PWALTGGWNS QMCCQGEQNP PGPFWKAAFA
gi826271801  TLGAPGEPRH DSLLPVDSYQ SWALAGGWNS QMCCQGEQNP PGPFWKAAFA
gi589917310  TLGAPGEPRH DSLLPVDSYQ PWALAGGWNS QMCCQGEQNP PGPFWKAAFA
gi507543189  TLGTPGEPRH DSLLPVDSYQ PWALAGGWNS QMCCQGEQNP PGPFWKAAFA
gi395532657  TLGGPGEPRH DTLLPVDSYQ PWALASGWSG QMCCQGEQNQ PGHFWKAAFA
gi597740267  TLGT-GEPRH DSLLPMDSYQ PWALANGWGG QMYCSKDQSQ AGHLWKSALA
gi725576032  TLGAPGEPRH DSLLPVDSYQ SWALAGGWNS QMCCQGEQNP PGPFWKAAFT
gi742135928  TLGAPGEPRH DSLLPVDSYQ PWALTGGWNS QMCCQGEQNP PGPFWKAAFA
gi348562271  TLGTPGEPRH DSLLPVDGYQ PWALASGWNS QMCCQGDQNP PGPFWKAAFA
gi594050844  TLGAPGEPRH DSLLPVDSYQ PWALTGGWNS QMCCQGEQNP PGPFWKAAFA
gi533160875  TLGAPGEPRH DPLLPGDSYQ PWALASGWSS QMCCQGEQNP PGPFWKAAFA
gi556738344  TLGAPGEPRH DSLLPVDSYQ PWALTGGWNS QMCCQGEQNP PGPFWKAAFA
gi803246204  TLGAPGEPRH DSLLPVDSYQ PWALTGGWNS QMCCQGEQNP PGPFWKAAFA
gi354474760  TLGAPGEPRH DSLLPVDSYQ PWALASGWNS QMCCQGEQNP PGPFWKAAFA
gi532051321  TLGAPGEPRH DSLLPVDSYQ PWALAGGWNS QMCCQGEQNP PGPFWKATFA
```

gi311267502	TLGTPAEPRH	DSLLPVDSYQ	PWALTGGWNS	QVCCQGEQNP	SGSFWKAAFA
gi743729506	TLGGPGEPRH	DSLLPVDSYQ	PWALTGGWNS	QMCCQGEQNP	PGPFWKAAFA
gi359754103	TLGGPGEPRH	DTLLPVDSYQ	PWALASGWSG	QMCCQGEQNQ	PGHFWKAAFA
gi576886849	TLGT-SEPRH	DSLLPMDSYQ	PWALANGWGG	QMYCSKDQSQ	AGHLWKSALA
gi586448761	TLGAPGEPRH	DSLLPVDSYQ	PWALAGSWNS	QMCCQGEQTP	PGPFWKAAFA
gi504159820	TLGAPGEPRH	DPLLPVDSYQ	PWALAGGWNS	QMCCQGEQNP	PGPFWKTAFA
gi524951862	TLGAPGEPRH	DSLLPVDSYQ	PWALAGGWNS	QMCCQGEQNP	PGPFWKAAFA
gi332692483	TLGT-GEPRH	ETLLPMDSYQ	PWALTNGWGS	QMYCAKDPSQ	SGNLWKSALV
gi385654474	TLGT-GEPRH	ETLLPMDSYQ	PWALTNGWGS	QMYCAKDPSQ	SGNLWKSALV
gi478520959	TLGAPGEPRH	DSLLPVDSYQ	PWALTGGWNS	QMCCQGEQNP	PGPFWKAAFA
gi301128881	TISGAGEPRH	EALLPMEGYQ	PWAL-NGWNS	QMCCSKDQAQ	SGHLWKSTLA
gi1011609041	TMGGPGEARH	ETLLPVDSYQ	PWALTSGWNS	QLCCPKDQSQ	AGHFWKSAFA
gi664747354	TLGAPGEPRH	DSLLPVDSYQ	PWALTGGWNS	QMCCQGEQNP	PGPFWKAAFA
gi557280818	TMGGPGEARH	ETLLPVDSYQ	PWALTSGWNS	QLCCPKDQSQ	AGHFWKSAFA
gi359076815	TLGAPGEPRH	DSLLPVDSYQ	PWALTGGWNS	QMCCQGEQNP	PGPFWKAAFA
gi632972216	TISGAGEPRH	EALLPMDSYQ	PWAL-NGWNS	QMYC-KEQAQ	PPHLWKSTLA
gi759185571	TLGAPGEPRH	EALLPMDSYQ	PWALTSGWNS	QMCCQGEQNP	PGPFWKATCT
gi472350967	TLGAPGEPRH	DSLLPVDSYQ	PWALTGGWNG	QMCCQGEQNP	PGPFWKATFA
gi586539979	TLGAPGEPHH	EALLPMDSYQ	PWALTSGWNS	QMCCQGEQNP	PGAFWKATCT
gi157823841	TLGAPGEPRH	DSLLPVDSYQ	PWALAGGWNS	QMCCQGEQNP	PGPFWKAAFA
gi410980861	TLGAPGEPRH	DSLLPVDSYQ	SWALTGGWNS	QMCCQGEQNP	PGPFWKAAFA
gi829772881	TLGAPGEPRH	DSLLPVDSYQ	SWALAGGWNS	QMCCQGEQNP	PGPFWKAAFA
gi220898176	TIGGPGEPRH	EPLLPMETYQ	PWAITNGWNG	QVYCSKEQAQ	PNHLWKSTLP
gi1519038	TLGAPGEPRH	DSLLPVDSYQ	PWALAGGWNS	QMCCQGEQNP	PGPFWKAAFA
gi731207261	TLGAPGEPRH	DTLLPVDGYQ	PWALAGGWNS	QMCCQGEQNP	PGPFWK-AFA
gi20278975	TISAPGEPRH	DTLLPMESYQ	PWAITNGWNG	QVCCSKEQPQ	ATHLWKSSLP
gi585176637	TLGAPGEPRH	ESLLPVDSYQ	PWALTGGWNG	QMCCQGEQNP	PGPFWKAAFA
gi560984071	TLGGPGEPRH	DSLLPVDSYQ	PWAITGGWNS	QMCCQGEQNP	PGHFWKAAFA
gi73966255	TLGAPGEPRH	DSLLPVDSYQ	PWALTGGWNG	QMCCQGEQNP	PSAFWKAAFA
gi511890491	TLGAPGEPRH	DSLLPVDSYQ	PWSLTGGWNG	QMCCQGEQNP	PGPFWKAAFA
gi254212171	TISAPGEPRH	ETLLPMESYQ	PWAITNGWNG	QVCCSKEQPQ	PTHLWKSSLP
gi558229251	TIGGPGEPRH	EPLLPMESYQ	PWAITSGWNG	QVYCPKEQTQ	PPHLWKSTLP
gi752394348	TLGAPGEPRH	DSLLQVDSYQ	PWALAGGWNG	QMCCQGEQNP	PGPFWKAAFA
gi507952779	TLGAPGEPRH	DSLLPVDSYQ	PWALSSGWNS	QMCCQGEQNP	PGPFWKAAFA
gi507664676	TLGASGEPRH	DALLPVDSYQ	PWPLAGGWNS	QMCCQGEPNP	PGPFWKAPFA
gi224045310	AIGGPGEPRH	DPLLPMDSYQ	PWAITNGWNG	QVYCPKEQSQ	PPHLWKSTLP
gi400180333	TISGPGEPRH	ESLLPVESYQ	PWAITNGWNG	QVYCSKEQTQ	PTHLWKSTIQ
gi641724374	TLGAPGEPRH	DALLPVDSYQ	PWALPGGWNS	QMCCPGDQNP	PGSFWKTAFA
gi693302455	TLGGPGEPRH	ESLLPMESYQ	PWAIPNGWNG	QVYCSKEQTQ	PPHLWKSTLS
gi542179924	TLGT-GEPRH	DTLLPMDSYQ	PWALTNAWGG	QMYCSKDQGQ	AGHLWKSALA
gi119370791	TLGA-GEPRH	DTLLPMDSYQ	PWALTNGWGG	QMYCSKDQGQ	AGHLWKSALA
gi678126544	GMSGPAEPRH	DPLLPMESYQ	PWAITNGWNG	QVYCAKEQSQ	PNHMWKASIQ
gi808884419	TLGT-GEPRH	DTLLPMDSYQ	PWALTNGWGG	QMYCSKDQGQ	AGHLWKSALA
gi505802773	TLGTPGEPRH	DSLLPVDSYQ	PWALPGGWNG	QMCCQGEQTP	PGPFWKAAFA
gi34484442	TLGA-GEPRH	DTLLPMDSYQ	PWALTNGWGG	QMYCSKDQGQ	AGHLWKSALA
gi519777295	TVGGPGEPRH	EPLLPMEPYQ	PWALTNGWNG	QVYCSKEQGQ	PPHLWKSSLP
gi657803392	TLGT-GEPRH	DTLLPMDSYQ	PWALTNGWGG	QMYCSKDQGQ	AGHLWKSALA
gi554874539	TLGT-GEPRH	DTLLPMDSYQ	PWALTNAWGG	QMYCSKDQGQ	AGHLWKSALA
gi499036069	TLGT-GEPRH	DTLLPMDSYQ	PWALTNAWGG	QMYCSKDQGQ	AGHLWKSALA
gi301607689	TIGTAGEPRH	EPLLPMDGYQ	AWPITNGWNG	QVYCAKDQAQ	PTHLWKSSLP
gi584023332	TLGT-GEPRH	DTLLPMDSYQ	PWALTNAWGG	QMYCSKDQGQ	AGHLWKSALA
gi548549273	TLGT-GEPRH	DTLLPMDSYQ	PWALTNAWGG	QMYCSKDQGQ	ASHLWKSALA
gi657574876	TLGT-GEPRH	DTLLPMDSYQ	PWALTNGWGG	QMYCSKDQGQ	AGHLWKSALA
gi28268588	TIGSTGKPRH	EPLLPMDGYQ	AWPITNGWNG	QVYCAKDQAQ	PTHLWKSSLP
gi736251858	TLGS--EPRH	DTLLPMDSYQ	PWALTNGWGG	QMYC-KDQGQ	AGHLWKSALA
gi530624973	TIGGPGEPRH	ETLLPVDSYQ	PWALTGGWNS	QMCCPKDQSQ	AGHFWKSAFA
gi290760363	TIGGRGEPRH	DSMLPVEGYQ	PWAIPNGWNG	QVYCPKEQNQ	PPHLWKSALP
gi551520979	TLGS-GDPRH	ETLLPMDSYQ	PWALTNGWGG	QMYCSKDQTQ	AGHLWKTALA
gi658861163	TLGS-GNPRH	ETLLPMDSYQ	PWALTNGWGG	QMYCSKDQAQ	AGHLWKTALA
gi617509429	TLGS-GDPRH	ETLLPMDSYQ	PWALTNGWGG	QMYCSKDQAQ	AGHLWKTALA
gi47229429	TISAPSEARH	ESLLPVDSYQ	PWTLPNGWNS	QVYCAKEQPQ	PGHMWKSSVA
gi831555258	AISAASEARH	ESLLPMESYQ	PWTLPNGWNG	QVYCAKEQPQ	PGHMWKSSLP
gi591374792	TIGGPGEPRH	ETLLPVDSYQ	PWALTSGWNS	QMCCPKDQNQ	AGHLWKSAFS
gi432871359	TLGA-GEPRH	DSLLPMDGYQ	PWAL-NGWGG	QMYCSKEQAQ	AGHLWKSALA
gi602643660	TLGGRGEPRH	DSMLPVEGYQ	PWAIPNGWNG	QVYCPKEQNQ	PPHLWKSALP
gi961915500	ALSAPPEPR-	ESLLPMEPYQ	PWAITNGWSG	QVYCTKEQSQ	ANSLWKSSLQ
gi961860111	ALSAPPEPR-	ESLLPMEPYQ	PWAITNGWSG	QVYCTKEQSQ	ANSLWKSSLQ
gi219879168	ALSTPAEPRH	ESLLPVETYQ	PWAITNGWSS	PVYCPKDQTQ	SNSLWKSSIQ
gi219879156	ALSTPAEPRH	ESLLPVETYQ	PWAITNGWSS	PVYCPKDQTQ	SNSLWKSSIQ

```
gi219879164  ALSTPAEPRH ESLLPVETYQ PWAITNGWSS PMYCPKDQTQ SNSLWESSIQ
gi123204435  ALSAPAEPRH ESLLPVETYQ PWAITNGWSS PVYCPKDQTQ SNTLWKSSIQ
gi465985114  GLGGPGESRH EPLLPMESYQ PWALPNGWNG QMYCPKEQAQ PSHLWKSTLP
gi426355784  GLGGPGESRH EPLLPMESYQ PWALPNGWNG QMYCPKEQAQ PPHLWKSTLP
gi1034052196 GLGGPGESRH EPLLPMESYQ PWALPNGWNG QMYCPKEQAQ PPHLWKSTLP
gi683901043  AIGGPGEPRH DPLLPMDSYQ PWAITNGWNG QVYCPKEQSQ PPHLWKSTLP
gi729725749  GLGGPGEARH ETLLPVDGYQ PWALGGGWSS QMCCPKEQGQ AGYLLKSAFA
gi663243102  GLGGPGEARH ETLLPVDGYQ PWALGGGWSS QMCCPKEQGQ AGYLLKSAFA
gi339792792  TVGGPGEPRH EPLLPMETYQ PWAITNGWNG QVYCSKEQAQ PPHLWKSALP
gi768393729  GLGGPGEARH ETLLPVDGYQ PWALGGGWSS QMCCPKEQGQ AGYLLKSAFA
gi670977729  TLGAPGEPRH DSLLQADSYQ PWALAGGWTG QMCCQGEQNP PGPFWKAAFA
gi327263844  GISGHPEPRH DALLPMDGYQ HWALSNGWDG QVYCSKEQPQ SGHLWKSPFP
gi1040268871 GISGHPEPRH DALIPVEGYQ HWALSNGWDS QVYCSKEQSQ SAHLWKSPFP
gi303303637  GISGHPEPRH DALIPVEGYQ HWALSNGWDS QVYCSKEQSQ SAHLWKSPFP
gi724877051  GISGHPEPRH DALIPVEGYQ HWALSNGWDS QVYCSKEQSQ SAHLWKSPFP
gi544470246  GISGHPEPRH DALIPVEGYQ HWALSNGWDS QVYCSKEQSQ SAHLWKSPFP
gi338726307  GISGHPEPRH DALIPVEGYQ HWALSNGWDS QVYC-KEQSQ SAHLWKSPFP
gi297692036  GISGHPEPRH DALIPVEGYQ HWALSNGWDS QVYCSKEQSQ SAHLWKSPFP
gi397521957  GISGHPEPRH DALIPVEGYQ HWALSNGWDS QVYCSKEQSQ SAHLWKSPFP
gi1018974175 GLGGPAEPRH EPLLPMESYQ PWALPNGWNG QVYCPKEQPQ PPHLWKSTLP
gi675430530  TFGS-GEPRH ETYISMEGYQ SWTLANGWNS QVYCAKDQTQ SSHFWKSSFP
gi795174618  TFGS-GEPRH EAYISMEGYQ SWTLANGWNS QVYCAKDQPQ GSHFWKSSFP
gi699633156  TFGS-GEPRH ETYISMEGYQ SWTLANGWNS QVYCAKDQTQ SSHFWKSSFP
gi374498883  TFGS-GEPRH ETYISMEGYQ SWTLANGWNS QVYCAKDQTQ SSHFWKSSFP
gi24637241   GISGHPEPRH DALIPMEGYQ HWTLSNGWDG QVYCPKEQGQ AGHLWKSPFP
gi584059997  TLGAPGEPRH DALLPVDSYQ PWALPGGWNS QMCCQGEQNP PGSFWKTAFA
gi884764069  GLGGPGDARH ETLLPVDGYQ PWALGGGWSS QMYCPKEQGQ AGYLLKSAFA
gi66773958   TFGS-GEPRH EAYISMEGYQ SWTLANGWNS QVYCAKDQPQ GSHFWKSSFP
gi224016307  TFGS-GEPRH EAYISMEGYQ SWTLANGWNS QVYCAKDQPQ GSHFWKSSFP
gi224016295  TFGS-GEPRH EAYISMEGYQ SWTLANGWNS QVYCAKDQPQ GSHFWKSSFA
gi224016303  TFGS-GEPRH EAYISMEGYQ SWTLANGWNS QVYCAKDQPQ GSHFWKSSFP
gi224016309  TFGS-GEPRH EAYISMEGYQ SWTLANGWNS QVYCAKDQPQ GSHFWKSSFP
gi374498861  TFGS-GEPRH ETYISMEGYQ SWTLANGWNS QVYCAKDQTQ SSHFWKSSFP
gi224016313  TFGS-GEPRH EAYISMEGYQ SWTLANGWNS QVYCAKDQPQ GSHFWKSSFP
gi224016305  TFGS-GEPRH EAYISMEGYQ SWTLANGWNS QVYCAKDQPQ GSHFWKSSFP
gi224016311  TFGS-GEPRH EAYISMEGYQ SWTLANGWNS QVYCAKDQPQ GSHFWKSSFP
gi224016299  TFGS-GEPRH EAYISMEGYQ SWTLANGWNS QVYCAKDQPQ GSHFWKSSFP
gi700384804  TFGS-GEPRH ETYISMEGYQ SWTLANGWNS QVYCAKDQTQ SSHFWKSSFP
gi543378707  GLG---EPRP EALLPVDGHQ PWALGGGWGG QMCCPKEQGQ PGFLLKSAFA
gi699659589  GLGGPGDARH ETLLPVDSYQ PWALGSSWSS QMCCSKEQGQ AWYHLKSAFA
gi310656724  TFS-PGEPRH DTYISMEGYQ SWTLANGWNS QVYCAKDQAQ TPPFWKSSFP
gi565321354  TIGGRGEPRH DSMLPVEGYQ PWAIPNGWNG QVYCPKEQNQ PPHLWKSALP
gi290760375  GSAAAGEPRH ETYISMEGYQ SWTLANGWNG QVYCAKDQAQ GSHFWKSSFP
gi696966138  TFGS-GEPRH ETYISMEGYQ SWTLANGWNS QVYCAKDQTQ SSHFWKSSFP
gi677975869  GLGAPGEPRH ETLLPVDGYQ PWALGGGWSS QMCCPKEQGQ TGYLLKSAFA
gi543746989  TFGS-GEPRH ETYISMEGYQ SWTLANGWNS QVYCAKDQTQ SSHFWKSSFP
gi701389587  TFGS-GEPRH ETYISMEGYQ SWTLANGWNS QVYCAKDQTQ SSHFWKSSFP
gi256014533  GISGHPEPRH DALLPMEGYQ HWALSNGWDG QVYCAKEQSQ STHLWKSPFP
gi669281149  TFGS-GEPRH ETYISMEGYQ SWTLANGWNS QVYCAKDQTQ SSHFWKSSFP
gi118102888  GLGGPGEPRH EALLPVDGYQ PWALGGSWSG QMCCPKEQGQ AGYLLKSAFA
gi554547429  TLGAPGEPRH DALLPVDSYQ PWALPGGWNS QVCCQGEQNP SGSFWKTAFA
gi694854743  GLGGHPEPRH DALLPMEGYQ HWALSNGWDG QVYCSKEQSQ SAHLWKSPFP
gi529426764  GLGG-----H DALLPMEGYQ HWALSNGWDG QVYCSKEQSQ SAHLWKSPFP
gi706150961  GLGGPGEARH ETLLPVDGYQ PWALGGGWSS QMCCPKEQGQ AGYLLKSAFP
gi719780455  TLGAPGEARH ETLLPVDSYQ PWALSGGWD- QMCCPKEQSQ AGYFLKSAFA
gi290760370  GSAAAGEPRH ETYISMEGYQ SWTLANGWNS QVYCAKDQAQ GSHFWKSSFP
gi328682541  TLGSSVESRH DPFIPVDGYQ PWPPTNSWNS QIYCAKEQTQ GSHFWKSPYT
gi541961056  GLGGHPEPRH DPLLPMEGYQ HWALSNGWDG QVYCSKEQSQ SAHLWKSPFP
gi733928584  GLGAHPEPRH DALLPMEGYQ HWALSNGWDG QVYCSKEQSQ SAHLWKSPFP
gi686600796  GLGGHPEPRH DALPPLEGYQ HWALSNGWDG QVYCSKEQSQ SAHLWKSPFP
gi704535956  GLGGPGEARH ETLLPVDGYQ PWA--HGWSS QMCCPKEQGQ AGYLLKSAFA
gi555983692  GLGGPGESRH EPLLPMESYQ PWALPNGWNG QMYCPKEQAQ PPHLWKSTLP
gi591300470  GLGGPGESRH EPLLPMESYQ PWALPNGWNG QMYCPKEQAQ PPHLWKSTLP
gi255683553  SWRA----RH EPLLPMESYQ PWQLQ--WLN GQCTA--QDQ PAPL--ESLP
gi255683559  SWRA----RH EPLLPMESYQ PWQ--SQWLN GQCTA--QDQ PAPL--ESLP
gi558170872  TLGAPGEPRH DALLPVDSYQ PWALPGGWNS QMCCQGEQNP PGSFWKTAFA
gi255683557  SWRA----RH EPLLPMESYQ PWQ--SQWLN GQCTA--QDQ PAPL--ESLP
gi874491859  GLGGPGEARH ETLLPVDGYQ PWALGGSWSG QMCCPKEQGQ AGYLLKSAFA
gi726998761  ALGAHPEPRH EALLPMEGYQ HWALSNGWDG QVYCSKEQSQ SAHLWKSPFP
```

gi697489159	ALGGHPEPRH	DALLPMEGYQ	HWALSNGWDG	QMYCSKEQSQ	SAHLWKSPFP
gi525030647	ALGAHPEPRH	EPLLPMEGYQ	PWALANGWDG	QVYCPKEQPQ	PAHLWKSPFP
gi222159495	ALSCPTEPRH	ESLLPMEPYQ	PWAIANGWNG	QVYCAKEQPQ	SNALWKSSIQ
gi187936703	SLAPPSEGRH	DPILHMDGYQ	PWALPNGWNG	QMYCAKEQSQ	LSHLWKSPLA
gi690457118	----------	----------	--------DG	QVYCSKEQSQ	SAHLWKSPFP
gi559163908	TLGGRGEPRH	DSVLPMEGYQ	PWAIPNGWNG	QVYCPKEQNQ	PPXXXXXXXX
gi559163610	TIGGPGEPRH	DSVLPVEGYQ	PWAITNGWNG	QVYCPKEQNQ	PPXXXXXXXX
gi559163543	TIGGPGEPRH	DPALPVEGYQ	PWAITNGWNG	QVYCPKEQNQ	PPXXXXXXXX
gi704260001	GLGGHPEPR-	----------	---------G	QVYCSKEQSQ	SAHLWKSPFP
gi559163971	TIGGPGEPRH	DSMLPMEGYQ	PWAITNGWNG	QVYCPKEQNQ	PPXXXXXXXX
gi559163730	ALGGRGEPRH	DSVL--DGYQ	PWAITNGWNG	QVYCPKEQNQ	PPXXXXXXXX
gi269204564	TIGGPGEPRH	DSMLPMESYQ	PWAITNGWNG	QVYCPKEQNQ	PPHXXXXXXX
gi222159485	GMSGPAETRH	EPLLPMESYQ	PWAITNGWNS	QVYCXXXXXX	XXXXXXXXXX
gi559164035	TIGGPAEPRH	ESMLPMEGYQ	PWAIPNGWNG	QVYCPKEQNQ	PPXXXXXXXX
gi222708647	TISAPGEPRH	EPLLPVESYQ	PWAXXXXXXX	XXXXXXXXXX	XXXXXXXXXX
gi290760385	TFGS-GEPRH	ETYISMEGYQ	SWTLANGWNS	QVYCAKDQAQ	GSHFWKSPFP
gi559163786	TIGGRGEPRH	DSMLPVEGYQ	PWSIPNGWNG	QVYCPKEQNQ	PPXXXXXXXX
gi559163846	TLGGRGEPRH	DSMLPMEGYQ	PWAITSGWNG	QVYCPKEQNQ	PPXXXXXXXX
gi318088612	SISAHPEPRH	DALIPMEGYQ	HWALSNGWDG	QVYCSKEQTQ	SSHXXXXXXX
gi318088602	SISAHPEPRH	DALIPMEGYQ	HWALSNGWDG	QVYCSKEQTQ	SSHXXXXXXX
gi222708673	ALSAPAEPRH	ESVLPVETYQ	PWAXXXXXXX	XXXXXXXXXX	XXXXXXXXXX
gi222708649	AISGPGEPRH	EPLLPMESYQ	PWAXXXXXXX	XXXXXXXXXX	XXXXXXXXXX
gi222708671	ALSAPAEPRH	DSLLPVETYQ	PWTXXXXXXX	XXXXXXXXXX	XXXXXXXXXX
gi222708655	TMSGPTEPRH	DSLLPMESYQ	PWAXXXXXXX	XXXXXXXXXX	XXXXXXXXXX
gi222708681	ALSAPAEPRH	ESLLPVETYQ	PWAXXXXXXX	XXXXXXXXXX	XXXXXXXXXX
gi318088600	SISAHPEPRH	DALIPMEGYQ	HWALSNGWDG	QVYCSKEQTQ	SSHXXXXXXX
gi318088598	SISAHPEPRH	DALIPMEGYQ	HWALSNGWDG	QVYCSKEQTQ	SSHXXXXXXX
gi222708679	ALSAPAEPRH	ESLLPVETYQ	PWAXXXXXXX	XXXXXXXXXX	XXXXXXXXXX
gi318088608	SISAHPEPRH	DALIPMEGYQ	HWALSNGWDG	QVYCSKEQTQ	SSHXXXXXXX
gi559164131	GISGHPEPRH	DALLPMEGYQ	HWALSNGWDG	QVYCSKEQSQ	SXXXXXXXXX
gi318088606	SISAHPEPRY	NALIPMEGYQ	HWALSNGWDG	HVYCSKEQKQ	HSHXXXXXXX
gi318088610	SISAHPEPRH	DALIPMEGYH	HWALSNGWDG	QVYCSKEQTQ	SSHXXXXXXX
gi222708677	A-----EPRH	ESLLPVETYQ	PWAXXXXXXX	XXXXXXXXXX	XXXXXXXXXX
gi222708657	ALSGSAEPRH	EPLLPVDSYQ	PWAXXXXXXX	XXXXXXXXXX	XXXXXXXXXX
gi700431350	XXXXXXXXXX	XXXXXXXXXX	XXXXXXXXXX	XMCCPKEQGQ	AGYLLKSAFA
gi387231673	TLGA-GEPRH	EALLPMDSYQ	PWALTGGWNS	QMCCQGEXXX	XXXXXXXXXX
gi661918810	TFGS-GEPRH	ETYISMEGYQ	SWTLANGWNS	QVYCAKDQAQ	SSHFWKSSFP
gi387231677	SLGAPGEPRH	DALLPVDSYQ	PWALTGGWNS	QMCCXXXXXX	XXXXXXXXXX
gi387231659	SLGAPGEPRH	DALLPVDSYQ	PWAFTGGWNS	QMCCQAEXXX	XXXXXXXXXX
gi325260872	-YGSPGDFRD	RTPQIIPSPE	SEALGSSKHK	DDKAELDPNN	PVANWIHA--
gi387231661	SLAAPGEPRH	DALLPVDSYQ	PWALTGGWNS	QMCCQAEXXX	XXXXXXXXXX
gi387231655	SLGAPGEPRH	DALLPVDSYQ	PWAFTGGWNS	QMCCQTEXXX	XXXXXXXXXX
gi387231679	SLGAPGEPRH	DALLPVDSYQ	PWAFTGGWNS	QMCCQAEXXX	XXXXXXXXXX
gi387231667	TLGAPGEPRH	DALMPVESYQ	PWALTGGWNS	QVCCQGEXXX	XXXXXXXXXX
gi387231227	XXXXXXXXXX	XXXXXXXXXX	XXXXXXXXXX	XXXXXXXXXX	XXXXXXXXXX
gi387231653	SLGAPGEPRH	DALLPVDSYQ	PWAFTGGWNS	QMCCQTEXXX	XXXXXXXXXX
gi699575612	XXXXXXXXXX	XXXXXXXXXX	XXXXXXXXXX	XXXXXXXXXX	XXXLLDFSIP
gi676248171	XXXXXXXXXX	XXXXXXXXXX	XXXXXXXXXX	XXXXXXXXXX	XXXXXXXXXX
gi723145109	XXXXXXXXXX	XXXXXXXXXX	XXXXXXXXXX	XXXXXXXXXX	XXXXXXXXXX
gi697016891	XXXXXXXXXX	XXXXXXXXXX	XXXXXXXXXX	XXXXXXXXXX	XXXXXXXXXX
gi677147237	XXXXXXXXXX	XXXXXXXXXX	XXXXXXXXXX	XXXXXXXXXX	XXXXXXXXXX
gi256014558	GISGHSEPRH	DALIPMEGYQ	HWALXXXXXX	XXXXXXXXXX	XXXXXXXXXX
gi679242416	XXXXXXXXXX	XXXXXXXXXX	XXXXXXXXXX	XXXXXXXXXX	XXXXXXXXXX
gi683471095	XXXXXXXXXX	XXXXXXXXXX	XXXXXXXXXX	XXXXXXXXXX	XXXXXXXXXX
gi9438781	XXXXXXXXXX	XXXXXXXXXX	XXXXXXXXXX	XXXXXXXXXX	XXXXXXXXXX
gi676827072	XXXXXXXXXX	XXXXXXXXXX	XXXXXXXXXX	XXXXXXXXXX	XXXXXXXXXX
gi387231651	TLGAPGEPRH	EALLPMDSYQ	PWALTSGWNS	QMCCQGEXXX	XXXXXXXXXX
gi677498936	XXXXXXXXXX	XXXXXXXXXX	XXXXXXXXXX	XXXXXXXXXX	XXXXXXXXXX
gi683474613	XXXXXXXXXX	XXXXXXXXXX	XXXXXXXXXX	XXXXXXXXXX	XXXXXXXXXX
gi387231675	SLGAPGEPRH	DALLPVDSYQ	PWALTGGWNS	QMYCQGEXXX	XXXXXXXXXX
gi677248448	XXXXXXXXXX	XXXXXXXXXX	XXXXXXXXXX	XXXXXXXXXX	XXXXXXXXXX
gi678138507	XXXXXXXXXX	XXXXXXXXXX	XXXXXXXXXX	XXXXXXXXXX	XXXXXXXXXX
gi700349256	XXXXXXXXXX	XXXXXXXXXX	XXXXXXXXXX	XXXXXXXXXX	XXXXXXXXXX
gi677225924	XXXXXXXXXX	XXXXXXXXXX	XXXXXXXXXX	XXXXXXXXXX	XXXXXXXXXX
gi121308926	YSGASSQGQH	H--LPYLNYP	SCATSASY--	-FNMAGDGGP	PG--WMFTFA
gi194591945	XXXXXXXXXX	XXXXXXXXXX	XXXXXXXXXX	XXXXXXXXXX	XXXXXXXXXX
gi195549518	XXXXXXXXXX	XXXXXXXXXX	XXXXXXXXXX	XXXXXXXXXX	XXXXXXXXXX
gi194591943	XXXXXXXXXX	XXXXXXXXXX	XXXXXXXXXX	XXXXXXXXXX	XXXXXXXXXX
gi387231665	TLGAPGEPRH	EALLPMDSYX	XXXXXXXXXX	XXXXXXXXXX	XXXXXXXXXX

```
gi195549516  XXXXXXXXXX XXXXXXXXXX XXXXXXXXXX XXXXXXXXXX XXXXXXXXXX
 gi34304667  XXXXXXXXXX XXXXXXXXXX XXXXXXXXXX XXXXXXXXXX XXXXXXXXXX
gi194396133  XXXXXXXXXX XXXXXXXXXX XXXXXXXXXX XXXXXXXXXX XXXXXXXXXX
gi387231669  SLGAPGEPRH DALXXXXXXX XXXXXXXXXX XXXXXXXXXX XXXXXXXXXX
gi429510514  NSSEGGDEKS EQQRRQQQQQ PKATQSPRDA DAEAAGDAEL SCGGGGGGGG
gi780155006  XXXSPPTSLG HHVLSTHGKH GYSSGSANPH PACGNGDDDP SRP---ESDS
gi387231671  SLGAAGEPRH DALLPVDSXX XXXXXXXXXX XXXXXXXXXX XXXXXXXXXX
gi387231657  SLGAPGEPRH DALLPVDSYQ PWALTGXXXX XXXXXXXXXX XXXXXXXXXX
gi387231649  SLGAPGEPRH DALLPVDSXX XXXXXXXXXX XXXXXXXXXX XXXXXXXXXX
gi229298086  XXXXXXXXXX XXXXXXXXXX XXXXXXXXXX XXXXXXXXXX XXXXXXXXXX
gi328682725  XXXXXXXXXX XXXXXXXXXX XXXXXXXXXX XXXXXXXXXX XXXXXXXXXX
gi217035843  XXXXXXXXXX XXXXXXXXXX XXXXXXXXXX XXXXXXXXXX XXXXXXXXXX

             201                                            250
      QUERY  DSSGQHPPDA CAFRRGRKKR IPYSKGQLRE LEREYAANKF ITKDKRRKIS
gi685597136  DSSGQHPPDA CAFRRGRKKR IPYSKGQLRE LEREYAANKF ITKDKRRKIS
 gi60653709  DSSGQHPPDA CAFRRGRKKR IPYSKGQLRE LEREYAANKF ITKDKRRKIS
gi795250298  DSSGQHPPDA CAFRRGRKKR IPYSKGQLRE LEREYAANKF ITKDKRRKIS
gi635093043  DSSGQHPPDT CAFRRGRKKR IPYSKGQLRE LEREYAANKF ITKDKRRKIS
gi109114206  DSSGQHPPDA CAFRRGRKKR IPYSKGQLRE LEREYAANKF ITKDKRRKIS
gi795583451  DSSGQHPPDA CAFRRGRKKR IPYSKGQLRE LEREYAANKF ITKDKRRKIS
gi559812841  DSSGQHPPDA CAFRRGRKKR IPYSKGQLRE LEREYAANKF ITKDKRRKIS
gi332259422  DSSGQHPPDA CAFRRGRKKR IPYSKGQLRE LEREYAANKF ITKDKRRKIS
gi795525940  DSSGQHPPDA CAFRRGRKKR IPYSKGQLRE LEREYAANKF ITKDKRRKIS
gi426237811  DSSAQHPPDG CAFRRGRKKR IPYSKGQLRE LEREYSANKF ITKDKRRKIS
gi744588246  DSSTQHPPDG CAFRRGRKKR IPYSKGQLRE LEREYSANKF ITKDKRRKIS
gi617633383  DSSAQHLPDG CTYRRGRKKR IPYSKGQLRE LEREYSTNKF ITKDKRRKIS
gi213511801  DVVA-HQHDG NSFRRGRKKR IPYTKVQLKE LEKEYAANKF ITKDKRRKIS
gi444517714  DSSTQHPPDG CSFRRGRKKR IPYSKGQLRE LEREYAANKF ITKDKRRKIS
gi640808225  DSSAQHPPEG CAFRRGRKKR IPYSKGQLRE LEREYAANKF ITKDKRRKIS
gi556958424  DVVA-HQQDG SSFRRGRKKR IPYTKVQLKE LEKEYATNKF ITKDKRRKIS
gi667260003  DASAQHPPDG CAFRRGRKKR IPYSKGQLRE LEREYAANKF ITKDKRRKIS
gi831328207  DVVA-HQHDG SSFRRGRKKR IPYTKVQLKE LEKEYAANKF ITKDKRRKIS
gi642105600  DVVA-HQHDG NSFRRGRKKR IPYTKMQLKE LEKEYAANKF ITKDKRRKIS
gi471380475  DSNAQHPPDG CAFRRGRKKR IPYSKGQLRE LEREYAANKF ITKDKRRKIS
gi674059771  ESSAQHPPEG CAFRRGRKKR IPYSKGQLRE LEREYAANKF ITKDKRRKIS
gi344285419  DSNSQHPPDG CAFRRGRKKR IPYSKGQLRE LEREYAANKF ITKDKRRKIS
gi742113362  DVVA-HQHDG NSFRRGRKKR IPYTKVQLKE LEKEYAANKF ITKDKRRKIS
gi852732630  DSSAQHPSDG CAFRRGRKKR IPYSKGQLRE LEREYAANKF ITKDKRRKIS
gi512996173  DSNAQHPSDG CAFRRGRKKR IPYSKGQLRE LEREYAVNKF ITKDKRRKIA
gi395826626  DSSAQHPPEG CAFRRGRKKR IPYSKGQLRE LEREYAANKF ITKDKRRKIS
gi126308251  DSSAQHPPDG CSYRRGRKKR IPYSKGQLRE LEREYAANKF VTKDKRRKIS
gi528767552  DSSTQHPPDG CAFRRGRKKR IPYSKGQLRE LEREYSANKF ITKDKRRKIS
gi532208489  DSSGQHPPDA CAFRRGRKKR IPYSKGQLRE LEREYAASKF ITKDKRRKIS
gi507654822  DSSAQHPSDG CAFRRGRKKR IPYSKGQLRE LEREYAANKF ITKDKRRKIS
gi488527225  DSSVQHPSEG CAFRRGRKKR IPYSKGQLRE LEREYAANKF ITKDKRRKIA
gi817274366  DSSGQHPPDA CAFRRGRKKR IPYSKGQLRE LEREYAASKF ITKDKRRKIS
gi585671866  DANAQHPPDG CAFRRGRKKR IPYSKGQLRE LEREYAANKF ITKDKRRKIS
gi573901311  DVVA-HQQDG SSFRRGRKKR IPYTKVQLKE LEKEYAANKF ITKDKRRKIS
gi532071900  DSSAQHPSEG CAFRRGRKKR IPYSKGQLRE LEREYAANKF ITKDKRRKIS
gi114149320  DVVA-HQHDG GSFRRGRKKR IPYTKVQLKE LEKEYAANKF ITKDKRRKIS
gi593727116  DSSAQHPPDG CAFRRGRKKR IPYSKGQLRE LEREYSANKF ITKDKRRKIS
gi470606860  DSSAQHPPDG CAFRRGRKKR IPYSKGQLRE LEREYSANKF ITKDKRRKIS
gi291405863  DASAQHPPDG CAFRRGRKKR IPYSKGQLRE LEREYAANKF ITKDKRRKIS
gi594656397  DSSAQHPPDG CAFRRGRKKR IPYSKGQLRE LEREYSANKF ITKDKRRKIS
gi634851243  DSNAQHPPDG CAFRRGRKKR IPYSKGQLRE LEREYAANKF ITKDKRRKIS
gi602709820  DSSAQHPPDG CAFRRGRKKR IPYSKGQLRE LEREYSANKF ITKDKRRKIS
gi826271801  DSSAQHPPEG CAFRRGRKKR IPYSKGQLRE LEREYSANKF ITKDKRRKIS
gi589917310  EPNTQHPPDG CAFRRGRKKR IPYSKGQLRE LEREYAANKF ITKDKRRKIS
gi507543189  ESSVQHPPDG CAFRRGRKKR IPYSKGQLRE LEREYAANKF ITKDKRRRIS
gi395532657  DASAQHPQDG CSYRRGRKKR IPYSKGQLRE LEREYAANKF VTKDKRRKIS
gi597740267  DVVA-HQHEG GSFRRGRKKR IPYTKVQLKE LEKEYAANKF ITKDKRRKIS
gi725576032  DSSGQHLPDA CAFRRGRKKR IPYSKGQLRE LEREYAASKF ITKDKRRKIS
gi742135928  DSSSQHPPEG CTFRRGRKKR IPYSKGQLRE LEREYSANKF ITKDKRRKIS
gi348562271  ESSVQHPSDG CAFRRGRKKR IPYSKGQLRE LEREYAANKF ITKDKRRKIS
gi594050844  DSSSQHPPEG CAFRRGRKKR IPYSKGQLRE LEREYSANKF ITKDKRRKIS
gi533160875  DSSSQHPSDG CAFRRGRKKR IPYSKGQLRE LEREYAANKF ITKDKRRKIS
gi556738344  DSSAQHPPDG CAFRRGRKKR IPYSKGQLRE LEREYSANKF ITKDKRRKIS
gi803246204  DSSAQHPPDG CAFRRGRKKR IPYSKGQLRE LEREYSANKF ITKDKRRKIS
```

```
gi354474760  EPSTQHPPDG CAFRRGRKKR IPYSKGQLRE LEREYATNKF ITKDKRRKIS
gi532051321  EPSTQHPPDG CAFRRGRKKR IPYSKGQLRE LEREYAANKF ITKDKRRKIS
gi311267502  DSSAQHPPEG CAFRRGRKKR IPYSKGQLRE LEREYSANKF ITKDKRRKIS
gi743729506  DSSTQHPPDG CAFRRGRKKR IPYSKGQLRE LEREYSANKF ITKDKRRKIS
gi359754103  DASAQHPPDG CSYRRGRKKR IPYSKGQLRE LEREYAANKF VTKDKRRKIS
gi576886849  DVVA-HQHDG GSFRRGRKKR IPYTKVQLKE LEKEYAANKF ITKDKRRKIS
gi586448761  DSNTQPPSDG CTFRRGRKKR IPYSKGQLRE LEHEYAANKF ITKEKRRKIS
gi504159820  DAGAQHPPDG CAFRRGRKKR IPYSKGQLRE LEREYAANKF ITKDKRRKIS
gi524951862  EPSTQHPPDG CAFRRGRKKR IPYSKGQLRE LEREYAANKF ITKDKRRKIS
gi332692483  DVVA-HQHDG GSFRRGRKKR IPYTKVQLKE LEKEYTANKF ITKDKRRKIS
gi385654474  DVVA-HQHDG GSFRRGRKKR IPYTKVQLKE LEKEYTANKF ITKDKRRKIS
gi478520959  DSSAQHPPDG CAFRRGRKKR IPYSKGQLRE LEREYSANKF ITKDKRRKIS
gi301128881  D-VAVHQQDG SSFRRGRKKR IPYTKVQLKE LEREYATNKF ITKDKRRKIS
gi1011609041 DAVAQHPPDG CSFRRGRKKR IPYSKGQLKE LEKEYASNKF ITKDKRRKIS
gi664747354  DSSAQHPADG CGFRRGRKKR IPYSKGQLRE LEREYSANKF ITKDKRRKIS
gi557280818  DAVAQHPPDG CSFRRGRKKR IPYSKGQLKE LEKEYASNKF ITKDKRRKIS
gi359076815  DSSSQHPPEG CTFRRGRKKR IPYSKGQLRE LEREYSANKF ITKDKRRKIS
gi632972216  DVVG-HQQDG GSFRRGRKKR IPYTKVQLKE LEREYGTNKF ITKDKRRKIS
gi759185571  DSSAQHPPDS CTIHRGRKKR IPYSKGQLRE LEREYSANKF ITKDKRRKIS
gi472350967  ESGAQHLPEG CTFRRGRKKR IPYSKGQLHE LEREYSANKF ITKDKRRKIS
gi586539979  DSSAQHPPDS CTIHRGRKKR IPYSKGQLRE LEREYSANKF ITKDKRRKIS
gi157823841  EPSVQHPPDG CAFRRGRKKR IPYSKGQLRE LEREYAANKF ITKDKRRKIS
gi410980861  DTSAQHPPEG CAFRRGRKKR IPYSKGQLRE LEREYSANKF ITKDKRRKIS
gi829772881  DSSAQHPSEG CAFRRGRKKR IPYSKGQLRE LEREYSANKF ITKDKRRKIS
gi220898176  DVVS-HPSDA NSFRRGRKKR VPYTKVQLKE LEREYATNKF ITKDKRRRIS
gi1519038    EPSVQHPPDG CAFRRGRKKR IPYSKGQLRE LEREYAANKF ITKDKRRKIS
gi731207261  DSSAPHPSDG CAFRRGRKKR IPYSKGQLRE LEREYAANKF ITKDKRRKIS
gi20278975   DVVS-HPSDA SSFRRGRKKR VPYTKVQLKE LEREYATNKF ITKDKRRRIS
gi585176637  ESGTQHPPDG CTFRRGRKKR IPYSKGQLRE LEREYSANKF ITKDKRRKIS
gi560984071  DSSTQHPPDG CAFRRGRKKR IPYSKGQLRE LEREYSANKF ITKDKRRKIS
gi73966255   ESGTQHPPDG CAFRRGRKKR IPYSKGQLRE LEREYSANKF ITKDKRRKIS
gi511890491  ESGVQHPPDG CAFRRGRKKR IPYSKGQLRE LELEYSANKF ITKDKRRKIS
gi254212171  DVVS-HPSDA SSFRRGRKKR VPYTKVQLKE LEREYATNKF ITKDKRRRIS
gi558229251  DVVS-HPSDA SSYRRGRKKR VPYTKVQLKE LEREYATNKF ITKDKRRRIS
gi752394348  ESGAQHPPEG CAFRRGRKKR IPYSKGQLRE LEREYSANKF ITKDKRRKIS
gi507952779  DSSVQHPPDG CAIRRGRKKR VPYSKGQLRE LEREYSANKF ITKDKRRKIS
gi507664676  DSNSQHPPDG CAFRRGRKKR IPYSKGQLRE LEREYSANKF ITKDKRRKIS
gi224045310  DVVS-HPSDA NSYRRGRKKR VPYTKVQLKE LEREYATNKF ITKDKRRRIS
gi400180333  DVVS-HPGDV GSFRRGRKKR VPYTKVQLKE LEREYATNKF ITKDKRRRIS
gi641724374  DSSAQHPPEG CTFHRGRKKR IPYSKGQLRE LEREYSANKF ITKDKRRKIS
gi693302455  DVVS-HPSDA NSYRRGRKKR VPYTKIQLKE LEREYASNKF ITKDKRRRIS
gi542179924  DVVA-HQNDG LSFRRGRKKR IPYTKVQLKE LEKEYAANKF ITKDKRRKIS
gi119370791  DVVA-HQHDG SPFRRGRKKR IPYTKVQLKE LEKEYAANKF ITKDKRRKIS
gi678126544  DAVS-HAGDG GAFRRGRKKR VPYTKVQLKE LEREYATNKF ITKDKRRRIS
gi808884419  DVVA-HQHDG SPFRRGRKKR IPYTKVQLKE LEKEYAANKF ITKDKRRKIS
gi505802773  DPASQHPPD- -SFRRGRKKR IPYSKGQLRE LEREYSVNKF ITKDKRRKIS
gi34484442   DVVA-HQHDG SPFRRGRKKR IPYTKIQLKE LEKEYAANKF ITKDKRRKIS
gi519777295  DVVS-HPSDA NSYRRGRKKR VPYTKVQLKE LEREYATNKF ITKDKRRRIS
gi657803392  DVVA-HQHDG SPFRRGRKKR IPYTKVQLKE LEKEYAANKF ITKDRRRKIS
gi554874539  DVVA-HQNDS LSFRRGRKKR IPYTKVQLKE LEKEYAANKF ITKDKRRKIS
gi499036069  DVVA-HQNDS LSFRRGRKKR IPYTKVQLKE LEKEYAANKF ITKDKRRKIS
gi301607689  DVV--HQSDS SSYRRGRKKR VPYTKVQLKE LEREYATNKF ITKDKRRRIS
gi584023332  DVVA-HQNDS LSFRRGRKKR IPYTKVQLKE LEKEYAANKF ITKDKRRKIS
gi548549273  DVVA-HQNDS LSFRRGRKKR IPYTKVQLKE LEKEYAANKF ITKDKRRKIS
gi657574876  DVVA-HQHDG SPFRRGRKKR IPYTKVQLKE LEKEYAANKF ITKDKRRKIS
gi28268588   DVV--HQSDS SSYRRGRKKR VPYTKVQLKE LEREYGTNKF ITKDKRRRIS
gi736251858  DAVA-HQHDG SHFRRGRKKR IPYTKLQLKE LEKEYAANKF ITKDKRRKIS
gi530624973  D-VGQPPPDA CSFRRGRKKR IPYTKGQLKE LEKEYASSKF ITKDKRRKIA
gi290760363  DVVS-HPSDA NSYRRGRKKR VPYTKVQLKE LEREYATNKF ITKDKRRRIS
gi551520979  DVVA-HQHEG SPFRRGRKKR IPYTKVQLKE LEKEYAANKF ITKDKRRKIS
gi658861163  DVV-VHQHEG SPFRRGRKKR IPYTKVQLKE LEKEYAANKF ITKDKRRKIS
gi617509429  DVVA-HQHEG SPFRRGRKKR IPYTKVQLKE LEKEYAANKF ITKDKRRKIS
gi47229429   DAAS-HTGES GSYRRGRKKR VPYTKVQLKE LEREYAANKF ITKDKRRRIS
gi831555258  DAVSHAAGDV GSFRRGRKKR VPYTKVQLKE LEREYAANKF ITKDKRRRIS
gi591374792  D-AAQHPPDA GSFRRGRKKR VPYSKGQLKE LEKEYASSKF ITKDKRRKIA
gi432871359  DVVA-HQHDG SPFRRGRKKR IPYTKIQLKE LEKEYAANKF ITKDKRRKIS
gi602643660  DVVS-HPSDA NSYRRGRKKR VPYTKVQLKE LEREYATNKF ITKDKRRRIS
gi961915500  DSLSGGMD-- SSVRRGRKKR VPYTKVQLKE LEREYATNKF ITKDKRRRIS
gi961860111  DSLSGGMD-- SSVRRGRKKR VPYTKVQLKE LEREYATNKF ITKDKRRRIS
```

gi219879168	DSVSG--TDG	ASVRRGRKKR	VPYTKVQLKE	LEREYATNKF	ITKDKRRRIS
gi219879156	DSVSG--TDG	ASVRRGRKKR	VPYTKVQLKE	LEREYATNKF	ITKDKRRRIS
gi219879164	DTVSG--GDG	ASVRRGRKKR	VPYTKVQLKE	LEREYATNKF	ITKDKRRRIS
gi123204435	GT------DG	ASVRRGRKKR	VPYTKVQLKE	LEREYATNKF	ITKDKRRRIS
gi465985114	DVVS-HPSDA	SSYRRGRKKR	VPYTKVQLKE	LEREYATNKF	ITKDKRRRIS
gi426355784	DVVS-HPSDA	SSYRRGRKKR	VPYTKVQLKE	LEREYATNKF	ITKDKRRRIS
gi1034052196	DVVS-HPSDA	SSYRRGRKKR	VPYTKVQLKE	LEREYATNKF	ITKDKRRRIS
gi683901043	DVVS-HPSDA	NSYRRGRKKR	VPYTKVQLKE	LEREYATNKF	ITKDKRRRIS
gi729725749	DSAGQLPPDG	CSFRRGRKKR	VPYSKGQLKE	LEKEYASSKF	ITRDKRRKIS
gi663243102	DSAGQLPPDG	CSFRRGRKKR	VPYSKGQLKE	LEKEYASSKF	ITRDKRRKIS
gi339792792	D-VASHPSDA	NAYRRGRKKR	VPYTKVQLKE	LEREYATNKF	ITKDKRRRIS
gi768393729	DSAGQLPTDG	CSFRRGRKKR	VPYSKGQLKE	LEKEYASSKF	ITRDKRRKIS
gi670977729	ESGAQHPPEG	CAFRRGRKKR	IPYSKGQLRE	LEREYSANKF	ITKDKRRKIS
gi327263844	DVVPL-QPEV	SSYRRGRKKR	VPYTKIQLKE	LEKEYAASKF	ITKEKRRRIS
gi1040268871	DVVPL-QPEV	SSYRRGRKKR	VPYTKVQLKE	LEKEYAASKF	ITKEKRRRIS
gi303303637	DVVPL-QPEV	NSYRRGRKKR	VPYTKVQLKE	LEKEYAASKF	ITKEKRRRIS
gi724877051	DVVPL-QPEV	SSYRRGRKKR	VPYTKVQLKE	LEKEYAASKF	ITKEKRRRIS
gi544470246	DVVPL-QPEV	SSYRRGRKKR	VPYTKVQLKE	LEKEYAASKF	ITKEKRRRIS
gi338726307	DVVPL-QPEV	SSYRRGRKKR	VPYTKVQLKE	LEKEYAASKF	ITKEKRRRIS
gi297692036	DVVPL-QPEV	SSYRRGRKKR	VPYTKVQLKE	LEKEYAASKF	ITKEKRRRIS
gi397521957	DVVPL-QPEV	SSYRRGRKKR	VPYTKVQLKE	LEKEYAASKF	ITKEKRRRIS
gi1018974175	DVVS-HPSDA	NSYRRGRKKR	VPYTKVQLKE	LEREYATNKF	ITKDKRRRIS
gi675430530	GDVALNQPDM	CVYRRGRKKR	VPYTKLQLKE	LENEYAINKF	INKDKRRRIS
gi795174618	GDVALNQPDM	CVYRRGRKKR	VPYTKLQLKE	LENEYAINKF	INKDKRRRIS
gi699633156	GDVALNQPDM	CVYRRGRKKR	VPYTKLQLKE	LENEYAINKF	INKDKRRRIS
gi374498883	GDVALNQPDM	CVYRRGRKKR	APYTKLQLKE	LENEYAINKF	INKDKRRRIS
gi24637241	DVVPL-QPEV	SSYRRGRKKR	VPYTKAQLKE	LEKEYAASKF	ITKEKRRRIS
gi584059997	DSSAQHPPEG	CTFHRARKKR	IPYSKGQLRE	LEREYSANKF	ITKDKRRKIS
gi884764069	DSAGQLPPDG	CSFRRGRKKR	VPYSKVQLRE	LEKEYASSKF	ITRDKRRKIS
gi66773958	GDVALNQPDM	CVYRRGRKKR	VPYTKLQLKE	LENEYAINKF	INKDKRRRIS
gi224016307	GDVALNQPDM	CVYRRGRKKR	VPYTKLQLKE	LENEYAINKF	INKDKRRRIS
gi224016295	GDVALNQPDM	CVYRRGRKKR	VPYTKLQLKE	LENEYAINKF	INKDKRRRIS
gi224016303	GDVALNQPDM	CVYRRGRKKR	VPYTKLQLKE	LENEYAINKF	INKDKRRRIS
gi224016309	GDVALNQPDM	CVYRRGRKKR	VPYTKLQLKE	LENEYAINKF	INKDKRRRIS
gi374498861	GDVALNQPDM	CVYRRGRKKR	VPYTKLQLKE	LENEYAINKF	INKDKRRRIS
gi224016313	GDVALNQPDM	CVYRRGRKKR	VPYTKLQLKE	LENEYAINKF	INKDKRRRIS
gi224016305	GDVALNQPDM	CVYRRGRKKR	VPYTKLQLKE	LENEYAINKF	INKDKRRRIS
gi224016311	GDVALNQPDM	CVYRRGRKKR	VPYTKLQLKE	LENEYAINKF	INKDKRRRIS
gi224016299	GDVALNQPDM	CVYRRGRKKR	VPYTKLQLKE	LENEYAINKF	INKDKRRRIS
gi700384804	GDVALNQPDM	CVYRRGRKKR	VPYTKLQLKE	LENEYAINKF	INKDKRRRIS
gi543378707	GKARREPQEG	CALRRGRKKR	VPYSKGQLKE	LEKEFASSKF	ITRDRRRKIS
gi699659589	ASAGQLPPDG	CSFRRGRKKR	VPYSKGQLKE	LEKEYASSKF	ITRDKRRKIS
gi310656724	GEVALNQPDL	CVYRRGRKKR	VPYTKPQLKE	LENEYAINKF	INKDKRRRIS
gi565321354	DVVS-HPSDA	NSYRRGRKKR	VPYTKVQLKE	LEREYATNKF	ITKDKRRRIS
gi290760375	GDVALNQPDM	CVYRRGRKKR	VPYTKLQLKE	LESEYAVNKF	INKDKRRRIS
gi696966138	GDVALNQPDM	CVYRRGRKKR	VPYTKLQLKE	LENEYAINKF	INKDKRRRIS
gi677975869	DSPGQLPPDG	CSFRRGRKKR	VPYSKGQLKE	LEKEYASSKF	ITRDKRRKIS
gi543746989	GDVALNQPDM	CVYRRGRKKR	VPYTKLQLKE	LENEYAINKF	INKDKRRRIS
gi701389587	GDVALNQPDM	CVYRRGRKKR	VPYTKLQLKE	LENEYAINKF	INKDKRRRIS
gi256014533	DVVPL-QPEV	NSYRRGRKKR	VPYTKIQLKE	LEKEYAASKF	ITKEKXXXXX
gi669281149	GDVALNQPDM	CVYRRGRKKR	VPYTKLQLKE	LENEYAINKF	INKDKRRRIS
gi118102888	DAAAPLPPDG	CAFRRGRKKR	VPYSKGQLKE	LEKEYASSKF	ITRDKRRKIS
gi554547429	DSSAQHPPEG	CTFHRGRKKR	IPYSKGQLRE	LEREYSANKF	ITKDKRRKIS
gi694854743	DVVPL-QPEV	SSYRRGRKKR	VPYTKIQLKE	LEKEYAASKF	ITKEKRRRIS
gi529426764	DVVPL-QPEV	SSYRRGRKKR	VPYTKIQLKE	LEKEYAASKF	ITKEKRRRIS
gi706150961	DSAGPLPPEG	CSFRRGRKKR	VPYSKGQLKE	LEKEYASSKF	ITRDKRRKIS
gi719780455	DAAAQLPPDG	CSFRRGRKKR	VPYSKGQLKE	LEKEYASNKF	ITRDKRRKIS
gi290760370	GDVAVNQPDL	CVYRRGRKKR	VPYTKLQLKE	LESEYALNKF	INKDKRRRIS
gi328682541	GDGALSQADV	NSYRRGRKKR	VPYTKLQLKE	LEREYAVNKF	ITKDKRRKIA
gi541961056	DVVPL-QPEV	SSYRRGRKKR	VPYTKIQLKE	LEKEYAASKF	ITKEKRRRIS
gi733928584	DVVPL-QPEV	SSYRRGRKKR	VPYTKIQLKE	LEKEYAASKF	ITKEKRRRIS
gi686600796	DVVPL-QPEV	SSYRRGRKKR	VPYTKIQLKE	LEKEYAASKF	ITKEKRRRIS
gi704535956	DSTAQLPPDG	CSFRRGRKKR	VPYSKGQLKE	LEQEYASSKF	ITRDKRRKIS
gi555983692	DVVS-HPSDA	SSYRRGRKKR	VPYTKVQLKE	LEREYATNKF	ITKDKRRRIS
gi591300470	DVVS-HPSDA	SSYRRGRKKR	VPYTKVQLKE	LEREYATNKF	ITKDKRRRIS
gi255683553	DVVS-HPSDA	SSYRRGRKKR	VPYTKVQLKE	LEREYATNKF	ITKDKRRRIN
gi255683559	DVVS-HPSDA	SSYRRGRKKR	VPYTKVQLKE	LEREYATNKF	ITKDKRRRIN
gi558170872	DSSTQHPLEG	CTFHRGRKKR	IPYSKGQLRE	LEREYSANKF	ITKDKRRKIS
gi255683557	DVVS-HPSDA	SSYRRGRKKR	VPYTKVQLKE	LEREYATNKF	ITKDKRRRIN

gi874491859	DATTQLPSDG	CSFRRGRKKR	VPYSKGQLKE	LEKEYASSKF	ITRDKRRKIA
gi726998761	DVVPL-QPEV	SSYRRGRKKR	VPYTKIQLKE	LEKEYAASKF	ITKEKRRRIS
gi697489159	DVVPL-QPEV	SSYRRGRKKR	VPYTKIQLKE	LEKEYAASKF	ITKEKRRRIS
gi525030647	DVVPL-QPEG	SSFRRGRKKR	VPYSKVQLKE	LEKEFAASKF	ITKEKRRRIS
gi222159495	XXXXXXXXXX	XXXXXXXXXX	XXXXXXXXXX	XXXXXXXXXX	XXXXXXXXXX
gi187936703	D-GSQPPHEM	SLFRRGRKKR	VPYTKVQLKE	LEREYAANKF	ITKDKRRKIS
gi690457118	DVVPL-QPEV	SSYRRGRKKR	VPYTKIQLKE	LEKEYAASKF	ITKEKRRRIS
gi559163908	XXXXXXXXXX	XXXXXXXXXX	XXXXXXXXXX	XXXXXXXXXX	XXXXXXXXXX
gi559163610	XXXXXXXXXX	XXXXXXXXXX	XXXXXXXXXX	XXXXXXXXXX	XXXXXXXXXX
gi559163543	XXXXXXXXXX	XXXXXXXXXX	XXXXXXXXXX	XXXXXXXXXX	XXXXXXXXXX
gi704260001	DVVPL-QPEV	SSYRRGRKKR	VPYTKIQLKE	LEKEYAASKF	ITKEKRRRIS
gi559163971	XXXXXXXXXX	XXXXXXXXXX	XXXXXXXXXX	XXXXXXXXXX	XXXXXXXXXX
gi559163730	XXXXXXXXXX	XXXXXXXXXX	XXXXXXXXXX	XXXXXXXXXX	XXXXXXXXXX
gi269204564	XXXXXXXXXX	XXXXXXXXXX	XXXXXXXXXX	XXXXXXXXXX	XXXXXXXXXX
gi222159485	XXXXXXXXXX	XXXXXXXXXX	XXXXXXXXXX	XXXXXXXXXX	XXXXXXXXXX
gi559164035	XXXXXXXXXX	XXXXXXXXXX	XXXXXXXXXX	XXXXXXXXXX	XXXXXXXXXX
gi222708647	XXXXXXXXXX	XXXXXXXXXX	XXXXXXXXXX	XXXXXXXXXX	XXXXXXXXXX
gi290760385	GDVALNQPDM	CVYRRGRKKR	VPYTKLQLKE	LENEYTINKF	INKDKRRXXX
gi559163786	XXXXXXXXXX	XXXXXXXXXX	XXXXXXXXXX	XXXXXXXXXX	XXXXXXXXXX
gi559163846	XXXXXXXXXX	XXXXXXXXXX	XXXXXXXXXX	XXXXXXXXXX	XXXXXXXXXX
gi318088612	XXXXXXXXXX	XXXXXXXXXX	XXXXXXXXXX	XXXXXXXXXX	XXXXXXXXXX
gi318088602	XXXXXXXXXX	XXXXXXXXXX	XXXXXXXXXX	XXXXXXXXXX	XXXXXXXXXX
gi222708673	XXXXXXXXXX	XXXXXXXXXX	XXXXXXXXXX	XXXXXXXXXX	XXXXXXXXXX
gi222708649	XXXXXXXXXX	XXXXXXXXXX	XXXXXXXXXX	XXXXXXXXXX	XXXXXXXXXX
gi222708671	XXXXXXXXXX	XXXXXXXXXX	XXXXXXXXXX	XXXXXXXXXX	XXXXXXXXXX
gi222708655	XXXXXXXXXX	XXXXXXXXXX	XXXXXXXXXX	XXXXXXXXXX	XXXXXXXXXX
gi222708681	XXXXXXXXXX	XXXXXXXXXX	XXXXXXXXXX	XXXXXXXXXX	XXXXXXXXXX
gi318088600	XXXXXXXXXX	XXXXXXXXXX	XXXXXXXXXX	XXXXXXXXXX	XXXXXXXXXX
gi318088598	XXXXXXXXXX	XXXXXXXXXX	XXXXXXXXXX	XXXXXXXXXX	XXXXXXXXXX
gi222708679	XXXXXXXXXX	XXXXXXXXXX	XXXXXXXXXX	XXXXXXXXXX	XXXXXXXXXX
gi318088608	XXXXXXXXXX	XXXXXXXXXX	XXXXXXXXXX	XXXXXXXXXX	XXXXXXXXXX
gi559164131	XXXXXXXXXX	XXXXXXXXXX	XXXXXXXXXX	XXXXXXXXXX	XXXXXXXXXX
gi318088606	XXXXXXXXXX	XXXXXXXXXX	XXXXXXXXXX	XXXXXXXXXX	XXXXXXXXXX
gi318088610	XXXXXXXXXX	XXXXXXXXXX	XXXXXXXXXX	XXXXXXXXXX	XXXXXXXXXX
gi222708677	XXXXXXXXXX	XXXXXXXXXX	XXXXXXXXXX	XXXXXXXXXX	XXXXXXXXXX
gi222708657	XXXXXXXXXX	XXXXXXXXXX	XXXXXXXXXX	XXXXXXXXXX	XXXXXXXXXX
gi700431350	DSSGQLPPDG	CSFRRGRKKR	VPYSKGQLKE	LEKEYASSKF	ITRDKRRKIS
gi387231673	XXXXXXXXXX	XXXXXXXXXX	XXXXXXXXXX	XXXXXXXXXX	XXXXXXXXXX
gi661918810	GDVALNQPDM	CVYRRXXXXX	XXXXXXXXXX	XXXXXXXXXX	XXXXXXXXXX
gi387231677	XXXXXXXXXX	XXXXXXXXXX	XXXXXXXXXX	XXXXXXXXXX	XXXXXXXXXX
gi387231659	XXXXXXXXXX	XXXXXXXXXX	XXXXXXXXXX	XXXXXXXXXX	XXXXXXXXXX
gi325260872	----------	---RSTRKKR	CPYTKYQTLE	LEKEFLFNMY	LTRDRRYEVA
gi387231661	XXXXXXXXXX	XXXXXXXXXX	XXXXXXXXXX	XXXXXXXXXX	XXXXXXXXXX
gi387231655	XXXXXXXXXX	XXXXXXXXXX	XXXXXXXXXX	XXXXXXXXXX	XXXXXXXXXX
gi387231679	XXXXXXXXXX	XXXXXXXXXX	XXXXXXXXXX	XXXXXXXXXX	XXXXXXXXXX
gi387231667	XXXXXXXXXX	XXXXXXXXXX	XXXXXXXXXX	XXXXXXXXXX	XXXXXXXXXX
gi387231227	XXVVSHPSDS	SSYRRGRKKR	VPYTKVQLKE	LEREYATNKF	ITKDKRRRIS
gi387231653	XXXXXXXXXX	XXXXXXXXXX	XXXXXXXXXX	XXXXXXXXXX	XXXXXXXXXX
gi699575612	DVVS-HPPDA	NSYRRGRKKR	VPYTKVQLKE	LEREYATNKF	ITKDKRRRIS
gi676248171	XDVVSHPSDA	NSYRRGRKKR	VPYTKVQLKE	LEREYATNKF	ITKDKRRRIS
gi723145109	GDVALNQPDM	CVYRRGRKKR	VPYTKLQLKE	LENEYAINKF	INKDKRRRIS
gi697016891	GDVALNQPDM	CVYRRGRKKR	VPYTKLQLKE	LENEYAINKF	INKDKRRRIS
gi677147237	GDVALNQPDM	CVYRRGRKKR	VPYTKLQLKE	LENEYAINKF	INKDKRRRIS
gi256014558	XXXXXXXXXX	XXXXXXXXXX	XXXXXXXXXX	XXXXXXXXXX	XXXXXXXXXX
gi679242416	XDVVSHPSDA	NSYRRGRKKR	VPYTKVQLKE	LEREYATNKF	ITKDKRRRIS
gi683471095	XXXXXXXPDG	CSFRRGRKKR	VPYSKGQLKE	LEKEYASSKF	ITRDKRRKIS
gi9438781	XDVALNQPDM	CVYRRGRKKR	VPYTKLQLKE	LENEYAINKF	INKDKRRTIS
gi676827072	XXXXXXXPEG	CSFRRGRKKR	VPYSKGQLKE	LEREYASSKF	ITRDKRRKIS
gi387231651	XXXXXXXXXX	XXXXXXXXXX	XXXXXXXXXX	XXXXXXXXXX	XXXXXXXXXX
gi677498936	XXXXXXXXXX	XXYRRGRKKR	VPYTKVQLKE	LEREYATNKF	ITKDKRRRIS
gi683474613	GDVALNQPDM	CVYRRGRKKR	VPYTKLQLKE	LENEYAINKF	INKDKRR--S
gi387231675	XXXXXXXXXX	XXXXXXXXXX	XXXXXXXXXX	XXXXXXXXXX	XXXXXXXXXX
gi677248448	XDVVPLQPEV	SSYRRGRKKR	VPYTKIQLKE	LEKEYAASKF	ITKEKRRRIS
gi678138507	XXXXXXXXXX	XXXXXXXXXX	XXYSKGQLKE	LEKEYASSKF	ITRDKRRKIS
gi700349256	XXVVPLQPEV	SSYRRGRKKR	VPYTKIQLKE	LEKEYAASKF	ITKEKRRRIS
gi677225924	XXXXXXXXEV	SSYRRGRKKR	VPYTKIQLKE	LEKEYAASKF	ITKEKRRRIS
gi121308926	----------	-TFPR-RTKR	RPYSKVQIFE	LEKEFQLHQY	LTRDRRARLS
gi194591945	XXXXXXXXXX	XXXXXXRKKR	VPYTKVQLKE	LEREYATNKF	ITKDKRRRIS
gi195549518	XXXXXXXXXX	XXXXXXRKKS	VPYTKVQLKE	LEREYATNKF	ITKDKRRRIS

```
gi194591943   XXXXXXXXXX XXXXXXRKKR VPYTKVQLKE LERECATNKF ITKDKRRRIS
gi387231665   XXXXXXXXXX XXXXXXXXXX XXXXXXXXXX XXXXXXXXXX XXXXXXXXXX
gi195549516   XXXXXXXXXX XXXXXXRKKR VPYTKVQLKE LEREYATNKF ITKDKRRRIS
 gi34304667   XXXXXXXXXX XXXXXXRKKR QPYSKQQTRQ LEQEYAQNKF ITRQKREQIS
gi194396133   XXXXXXXXXX XXXXXXRKKS VPYTKVQLKD LEREYATNKF ITKDKRRRIS
gi387231669   XXXXXXXXXX XXXXXXXXXX XXXXXXXXXX XXXXXXXXXX XXXXXXXXXX
gi429510514   GTEKPGGSSG AAVQRSRKKR CPYTKFQIRE LEREFFFNVY INKEKRLQLS
gi780155006   DHSVSHQAVS ASYTRTRKKR KPYTKFQTFE LEKEFLYNMY LTRDRRSHIS
gi387231671   XXXXXXXXXX XXXXXXXXXX XXXXXXXXXX XXXXXXXXXX XXXXXXXXXX
gi387231657   XXXXXXXXXX XXXXXXXXXX XXXXXXXXXX XXXXXXXXXX XXXXXXXXXX
gi387231649   XXXXXXXXXX XXXXXXXXXX XXXXXXXXXX XXXXXXXXXX XXXXXXXXXX
gi229298086   XXXXXXXXXX XXXXRTRKKR RPYSKPQLAL LEDEYASQKF LTKEKRKEIS
gi328682725   XXXXXXXXXX XXXXXXXXXX XXYSKGQLKE LEKEYASNKF ITKDKRRKIS
gi217035843   XXXXXXXXXX XXXXXXXXXX XXYSKPQLAL LEDEYASQKF LTKEKRKEIS

              251                                              300
      QUERY   AATSLSERQI TIWFQNRRVK EKKVLAKVKN SATP
gi685597136   AATSLSERQI TIWFQNRRVK EKKVLAKXXX XXXX
 gi60653709   AATSLSERQI TIWFQNRRVK EKKVLAKVKN SATP
gi795250298   AATSLSERQI TIWFQNRRVK EKKVLAKVKN SATP
gi635093043   AATSLSERQI TIWFQNRRVK EKKVLAKVKN SATP
gi109114206   AATSLSERQI TIWFQNRRVK EKKVLAKVKN SATP
gi795583451   AATSLSERQI TIWFQNRRVK EKKVLAKVKN SATP
gi559812841   AATSLSERQI TIWFQNRRVK KKKVLAKVKN SATP
gi332259422   AATSLSERQI TIWFQNRRVK EKKVLAKVKN SATP
gi795525940   AATSLSERQI TIWFQNRRVK EKKVLAKVXX XXXX
gi426237811   AATSLSERQI TIWFQNRRVK EKKVLAKVKT SATP
gi744588246   VATSLSERQI TIWFQNRRVK EKKVLAKVKT SATP
gi617633383   AATSLSERQI TIWFQNRRVK EKKVLAKVKT SSTX
gi213511801   AGTNLSERQI TIWFQNRRVK EKKFVAKVKT SAXX
gi444517714   AATSLSERQI TIWFQNRRVK EKKVLAKVKT SATX
gi640808225   VATSLSERQI TIWFQNRRVK EKKVLAKVKN SATP
gi556958424   AATNLSERQI TIWFQNRRVK EKKVVAKIKP TTXX
gi667260003   AATSLSERQI TIWFQNRRVK EKKVLAKVKT SSTP
gi831328207   AATNLSERQI TIWFQNRRVK EKKFVAKVKT SAXX
gi642105600   TGTNLSERQI TIWFQNRRVK EKKFVAKVKT SAXX
gi471380475   AATSLSERQI TIWFQNRRVK EKKVLAKVKT MATP
gi674059771   AATSLSERQI TIWFQNRRVK EKKVLAKVKT SATX
gi344285419   AATSLSERQI TIWFQNRRVK EKKVLAKVKT ITTP
gi742113362   AVTNLSERQI TIWFQNRRVK EKKFVAKVKT SAXX
gi852732630   ATTSLSERQI TIWFQNRRVK EKKVLAKVKT SATP
gi512996173   AATSLSERQI TIWFQNRRVK EKKVLAKVKT SATP
gi395826626   AATSLSERQI TIWFQNRRVK EKKVLAKVKT SATP
gi126308251   AATSLSERQI TIWFQNRRVK EKKVLAKIKT SATP
gi528767552   VATSLSERQI TIWFQNRRVK EKKVLAKVKT SATP
gi532208489   AATSLSERQI TIWFQNRRVK EKKVLAKVKN STTP
gi507654822   AATSLSERQI TIWFQNRRVK EKKVLAKVKT SATP
gi488527225   AATSLSERQI TIWFQNRRVK EKKVLAKVKT SATP
gi817274366   AATSLSERQI TIWFQNRRVK EKKVLAKVKN SATP
gi585671866   AATSLSERQI TIWFQNRRVK EKKVLAKVKT SATP
gi573901311   AATNLSERQI TIWFQNRRVK EKKFVAKVKS STXX
gi532071900   AATSLSERQI TIWFQNRRVK EKKVLAKVKT SATP
gi114149320   AVTNLSERQI TIWFQNRRVK EKKFIAKVKN TAXX
gi593727116   AATSLSERQI TIWFQNRRVK EKKVLAKVKT SAIP
gi470606860   AATSLSERQI TIWFQNRRVK EKKVLAKVKT SAIP
gi291405863   AATSLSERQI TIWFQNRRVK EKKVLAKVKT SATP
gi594656397   AATSLSERQI TIWFQNRRVK EKKVLAKVKT SAIP
gi634851243   AATSLSERQI TIWFQNRRVK EKKVLAKVKT SATX
gi602709820   AATSLSERQI TIWFQNRRVK EKKVLAKVKT SGIP
gi826271801   AATSLSERQI TIWFQNRRVK EKKVLAKVKT SAIP
gi589917310   AATSLSERQI TIWFQNRRVK EKKVLAKVKT SATP
gi507543189   AATSLSERQI TIWFQNRRVK EKKVLAKVKS SATP
gi395532657   AATSLSERQI TIWFQNRRVK EKKVLAKIKT SATP
gi597740267   AVTNLSERQI TIWFQNRRVK EKKFIAKVKT SAXX
gi725576032   AATSLSERQI TIWFQNRRVK EKKVLAKVKN SATP
gi742135928   AATSLSERQI TIWFQNRRVK EKKVLAKVKT SATP
gi348562271   AATSLSERQI TIWFQNRRVK EKKVLAKVKT SATP
gi594050844   AATSLSERQI TIWFQNRRVK EKKVLAKVKT STTX
gi533160875   AATSLSERQI TIWFQNRRVK EKKVLAKVKT SATP
```

gi556738344	AATSLSERQI	TIWFQNRRVK	EKKVLAKVKT	SATP
gi803246204	AATSLSERQI	TIWFQNRRVK	EKKVLAKVKT	SATP
gi354474760	AATSLSERQI	TIWFQNRRVK	EKKVLAKVKT	SATP
gi532051321	AATSLSERQI	TIWFQNRRVK	EKKVLAKVKT	GATP
gi311267502	AATSLSERQI	TIWFQNRRVK	EKKVLAKVKT	SATP
gi743729506	VATSLSERQI	TIWFQNRRVK	EKKVLAKVKT	SATP
gi359754103	AATSLSERQI	TIWFQNRRVK	EKKVLAKIKT	NATP
gi576886849	AVTNLSERQI	TIWFQNRRVK	EKKFVAKVKS	SAXX
gi586448761	AATSLSERQI	TIWFQNRRVK	EKKVLAKVKT	SMTP
gi504159820	AATSLSERQI	TIWFQNRRVK	EKKVLAKVKT	SATP
gi524951862	AATSLSERQI	TIWFQNRRVK	EKKVLAKVKT	SATP
gi332692483	AVTNLSERQI	TIWFQNRRVK	EKKLSAKVKN	IAXX
gi385654474	AVTNLSERQI	TIWFQNRRVK	EKKLSAKVKN	VAXX
gi478520959	AATSLSERQI	TIWFQNRRVK	EKKVLAKVKT	STTP
gi301128881	AATNLSERQI	TIWFQNRRVK	EKKVIAKVKT	NTXX
gi1011609041	AVTNLSERQI	TIWFQNRRVK	EKKVIAKIKS	SSTP
gi664747354	AATSLSERQI	TIWFQNRRVK	EKKVLAKVKT	SATP
gi557280818	AVTNLSERQI	TIWFQNRRVK	EKKVIAKIKS	SSTP
gi359076815	AATSLSERQI	TIWFQNRRVK	EKKVLAKVKT	SAPP
gi632972216	AATNLSERQI	TIWFQNRRVK	EKKIVAKVKS	NTTX
gi759185571	AVTSLSERQI	TIWFQNRRVK	EKKVLTKVKT	SATX
gi472350967	AATSLSERQI	TIWFQNRRVK	EKKVLAKVKT	STTP
gi586539979	AVTSLSERQI	TIWFQNRRVK	EKKVLAKVKT	SATP
gi157823841	AATSLSERQI	TIWFQNRRVK	EKKVLAKVKT	STTP
gi410980861	AATSLSERQI	TIWFQNRRVK	EKKVLAKVKT	STTP
gi829772881	AATSLSERQI	TIWFQNRRVK	EKKVLAKVKT	SATP
gi220898176	ATTNLSERQV	TIWFQNRRVK	EKKVINKLKT	TSXX
gi1519038	AATSLSERQI	TIWFQNRRVK	EKKVLAKVKT	STTP
gi731207261	AATSLSERQI	TIWFQNRRVK	EKKVLAKVKT	SATP
gi20278975	ASTNLSERQV	TIWFQNRRVK	EKKVLTKLKS	NSXX
gi585176637	AATSLSERQI	TIWFQNRRVK	EKKVLAKVKT	STTP
gi560984071	AATSLSERQI	TIWFQNRRVK	EKKVLAKVKT	SATX
gi73966255	AATSLSERQI	TIWFQNRRVK	EKKVLAKVKT	STTX
gi511890491	AATSLSERQI	TIWFQNRRVK	EKKVLAKVKT	TTNP
gi254212171	ASTNLSERQV	TIWFQNRRVK	EKKVLTKLKS	NSXX
gi558229251	ATTNLSERQV	TIWFQNRRVK	EKKVINKLKT	TSXX
gi752394348	AATSLSERQI	TIWFQNRRVK	EKKVLAKVKT	TTTX
gi507952779	AATSLSERQI	TIWFQNRRVK	EKKVLAKVKT	SSTP
gi507664676	AATSLSERQI	TIWFQNRRVK	EKKVLSKVKT	SATP
gi224045310	ATTNLSERQV	TIWFQNRRVK	EKKVINKLKT	TSXX
gi400180333	AQTNLSERQV	TIWFQNRRVK	EKKVVHKTKS	TSXX
gi641724374	VATSLSERQI	TIWFQNRRVK	EKKVLAKVKT	NTTX
gi693302455	ATTNLSERQV	TIWFQNRRVK	EKKVINKLKS	TSXX
gi542179924	AVTNLSERQI	TIWFQNRRVK	EKKFVAKVKS	STXX
gi119370791	AATNLSERQI	TIWFQNRRVK	EKKFVAKVKS	NAXX
gi678126544	AQTNLSERQV	TIWFQNRRVK	EKKVVNKLKT	ASXX
gi808884419	AATNLSERQI	TIWFQNRRVK	EKKFVAKVKT	SAXX
gi505802773	AATSLSERQI	TIWFQNRRVK	EKKVLSKVKP	SATP
gi34484442	AATNLSERQI	TIWFQNRRVK	EKKFGAKVKS	SAXX
gi519777295	ATTNLSERQV	TIWFQNRRVK	EKKVINKLKT	TSXX
gi657803392	AMTNLSERQI	TIWFQNRRVK	EKKFVAKVKP	SAXX
gi554874539	AVTNLSERQI	TIWFQNRRVK	EKKFVAKVKS	STXX
gi499036069	AVTNLSERQI	TIWFQNRRVK	EKKFVAKVKS	STXX
gi301607689	ATTNLSERQV	TIWFQNRRVK	EKKVINKLKS	TSXX
gi584023332	AVTNLSERQI	TIWFQNRRVK	EKKFVAKVKS	STXX
gi548549273	AVTNLSERQI	TIWFQNRRVK	EKKFVAKVKS	STXX
gi657574876	AVTNLSERQI	TIWFQNRRVK	EKKFVAKVKS	SVXX
gi28268588	ATTNLSERQV	TIWFQNRRVK	EKKVINKLKS	TSXX
gi736251858	AGTNLSERQI	TIWFQNRRVK	EKKFVAKVKT	PTXX
gi530624973	AATNLTERQI	TIWFQNRRVK	EKKVVAKVKT	SSIP
gi290760363	AATNLSERQV	TIWFQNRRVK	EKKVINKLKT	SSXX
gi551520979	AVTNLSERQI	TIWFQNRRVK	EKKFVAKVKS	SAXX
gi658861163	AVTNLSERQI	TIWFQNRRVK	EKKFVAKVKS	SAXX
gi617509429	AVTNLSERQI	TIWFQNRRVK	EKKFVAKVKS	SAXX
gi47229429	AQTNLSERQV	TIWFQNRRVK	EKKVVNKLKT	ISXX
gi831555258	AQTNLSERQV	TIWFQNRRVK	EKKIVNKLKT	ISXX
gi591374792	AATSLTERQI	TIWFQNRRVK	EKKVVAKVKT	SSTP
gi432871359	AVTNLSERQI	TIWFQNRRVK	EKKFVAKVKS	SAXX
gi602643660	AATNLSERQV	TIWFQNRRVK	EKKVINKLKT	SSXX

gi961915500	AQTNLTERQV	TIWFQNRRVK	EKKVVNKFKS	SSXX
gi961860111	AQTNLTERQV	TIWFQNRRVK	EKKVVNKFKS	SSXX
gi219879168	AHTNLTERQV	TIWFXXXXXX	XXXXXXXXXX	XXXX
gi219879156	AHTNLTERQV	TIWFXXXXXX	XXXXXXXXXX	XXXX
gi219879164	AHTNLTERQV	TIWFXXXXXX	XXXXXXXXXX	XXXX
gi123204435	AHTNLTERQV	TIWFXXXXXX	XXXXXXXXXX	XXXX
gi465985114	ATTNLSERQV	TIWFQNRRVK	EKKVINKLKT	TSXX
gi426355784	ATTNLSERQV	TIWFQNRRVK	EKKVINKLKT	TSXX
gi1034052196	ATTNLSERQV	TIWFQNRRVK	EKKVINKLKT	TSXX
gi683901043	ATTNLSERQV	TIWFQNRRVK	EKKVINKLKT	TSXX
gi729725749	AATNLTERQI	TIWFQNRRVK	EKKVVAKIKS	SSSX
gi663243102	AATNLTERQI	TIWFQNRRVK	EKKVVAKIKT	SSSX
gi339792792	ATTNLSERQV	XXXXXXXXXX	XXXXXXXXXX	XXXX
gi768393729	AATNLTERQI	TIWFQNRRVK	EKKVVAKIKS	SSSX
gi670977729	AATSLSERQI	TIWFQNRRVK	EKKVLAKVKT	TTTP
gi327263844	ATTNLSERQV	TIWFQNRRXX	XXXXXXXXXX	XXXX
gi1040268871	ATTNLSERQV	TIWFQNRRXX	XXXXXXXXXX	XXXX
gi303303637	ATTNLSERQV	TIWFQNRRXX	XXXXXXXXXX	XXXX
gi724877051	ATTNLSERQV	TIWFQNRRXX	XXXXXXXXXX	XXXX
gi544470246	ATTNLSERQV	TIWFQNRRXX	XXXXXXXXXX	XXXX
gi338726307	ATTNLSERQV	TIWFQNRRXX	XXXXXXXXXX	XXXX
gi297692036	ATTNLSERQV	TIWFQNRRXX	XXXXXXXXXX	XXXX
gi397521957	ATTNLSERQV	TIWFQNRRXX	XXXXXXXXXX	XXXX
gi1018974175	ATTNLSERQV	TIWFQNRRVK	EKKVINKLKT	TSXX
gi675430530	AATNLSERQV	TIWFQNRRVK	DKKIVSKLKD	NVSX
gi795174618	AATNLSERQV	TIWFQNRRVK	DKKIVSKLKD	TVSX
gi699633156	AATNLSERQV	TIWFQNRRVK	DKKIVSKLKD	NVSX
gi374498883	AATNLSERQV	TIWFQNRRVK	DKKIVSKLKD	NVXX
gi24637241	AATGLSERQV	TIWFQNRRXX	XXXXXXXXXX	XXXX
gi584059997	VATSLSERQI	TIWFQNRRVK	EKKVLAKVKT	NATX
gi884764069	AATNLTERQI	TIWFQNRRVK	EXXXXXXXXX	XXXX
gi66773958	AATNLSERQV	TIWFQNRRVK	DKKIVSKLKD	TVSX
gi224016307	AATNLSERQV	TIWFQNRRVK	DKKIVSKLKX	XXXX
gi224016295	AATNLSERQV	TIWFQNRRVK	DKKIISKLKX	XXXX
gi224016303	AATNLSERQV	TIWFQNRRVK	DKKIVSKLKX	XXXX
gi224016309	AATNLSERQV	TIWFQNRRVK	DKKIVSKLKX	XXXX
gi374498861	AATNLSERQV	TIWFQNRRVK	DKKIVSXXXX	XXXX
gi224016313	AATNLSERQV	TIWFQNRRVK	DKKIVSKLKX	XXXX
gi224016305	AATNLSERQV	TIWFQNRRVK	DKKIVSKLKX	XXXX
gi224016311	AATNLSERQV	TIWFQNRRVK	DKKIVSKLKX	XXXX
gi224016299	AATNLSERQV	TIWFQNRRVK	DKKIVSKLKX	XXXX
gi700384804	AATNLSERQV	TIWFQNRRVK	DKKIVSKLKD	NVSX
gi543378707	AATNLSERQI	TIWFQNRRVK	EKKVVAKIKN	SAXX
gi699659589	AATNLTERQI	TIWFQNRRVK	EKKVVAKIKX	XXXX
gi310656724	ATTNLSERQV	TIWFQNRRVK	DKKLVSKLKE	TVXX
gi565321354	AATNLSERQV	TIWFQNRRVK	EKKVINKLKT	SSXX
gi290760375	AATNLSERQV	TIWFQNRRVK	DKKIVSKLKD	TVSX
gi696966138	AATNLSERQV	TIWFQNRRVK	DKKIVSKLKD	SVSX
gi677975869	AATNLTERQI	TIWFQNRRVK	EKKVVAKIKS	STSX
gi543746989	AATNLSERQV	TIWFQNRRVK	DKKIVSKLKD	NVSX
gi701389587	AATNLSERQV	TIWFQNRRVK	DKKIVSKLKD	NVSX
gi256014533	XXXXXXXXXX	XXXXXXXXXX	XXXXXXXXXX	XXXX
gi669281149	AATNLSERQV	TIWFQNRRVK	DKKIVSKLKD	NVSX
gi118102888	AATNLTERQI	TIWFQNRRVK	EKKVVAKIKS	SSXX
gi554547429	VATSLSERQI	TIWFQNRRVK	EKKVLAKVKT	NATX
gi694854743	ATTNLSERQV	TIWFQNRRXX	XXXXXXXXXX	XXXX
gi529426764	ATTNLSERQV	TIWFQNRRXX	XXXXXXXXXX	XXXX
gi706150961	AATNLTERQI	TIWFQNRRVK	EKKVVAKIKG	SSSX
gi719780455	AATNLTERQI	TIWFQNRRVK	EKKVVAKIKS	SGTP
gi290760370	AATNLSERQV	TIWFQNRRVK	DKKIVSKLKD	SVSX
gi328682541	AATSLSERQV	TIXXXXXXXX	XXXXXXXXXX	XXXX
gi541961056	ATTNLSERQV	TIWFQNRRXX	XXXXXXXXXX	XXXX
gi733928584	ATTNLSERQV	TIWFQNRRXX	XXXXXXXXXX	XXXX
gi686600796	ATTNLSERQV	TIWFQNRRXX	XXXXXXXXXX	XXXX
gi704535956	AVTNLSERQI	TIWFQNRRVK	EKKVVAKIKS	STSX
gi555983692	ATTNLSERQV	TIWFQNRRVK	EKKVINKLKT	TSXX
gi591300470	ATTNLSERQV	TIWFQNRRVK	EKKVINKLKT	TSXX
gi255683553	XXXXXXXXXX	XXXXXXXXXX	XXXXXXXXXX	XXXX
gi255683559	XXXXXXXXXX	XXXXXXXXXX	XXXXXXXXXX	XXXX

```
gi558170872  VATSLSERQI TIWFQNRRVK EKKVLAKVKT NATX
gi255683557  XXXXXXXXXX XXXXXXXXXX XXXXXXXXXX XXXX
gi874491859  AATNLTERQI TIWFQNRRVK EKKVVAKIKS SSTP
gi726998761  ATTNLSERQV TIWFQNRRXX XXXXXXXXXX XXXX
gi697489159  ATTNLSERQV TIWFQNRRXX XXXXXXXXXX XXXX
gi525030647  ASTSLSERQV TIWFQNRRVK EKKALSKAKX XXXX
gi222159495  XXXXXXXXXX XXXXXXXXXX XXXXXXXXXX XXXX
gi187936703  AAISLSERQV TIWFQNRRVK EKKVVSKMKT TXXX
gi690457118  ATTNLSERQV TIWFQNRRVK EKKVVSKTKT AXXX
gi559163908  XXXXXXXXXX XXXXXXXXXX XXXXXXXXXX XXXX
gi559163610  XXXXXXXXXX XXXXXXXXXX XXXXXXXXXX XXXX
gi559163543  XXXXXXXXXX XXXXXXXXXX XXXXXXXXXX XXXX
gi704260001  ATTNLSERQV TIWFQNRRXX XXXXXXXXXX XXXX
gi559163971  XXXXXXXXXX XXXXXXXXXX XXXXXXXXXX XXXX
gi559163730  XXXXXXXXXX XXXXXXXXXX XXXXXXXXXX XXXX
gi269204564  XXXXXXXXXX XXXXXXXXXX XXXXXXXXXX XXXX
gi222159485  XXXXXXXXXX XXXXXXXXXX XXXXXXXXXX XXXX
gi559164035  XXXXXXXXXX XXXXXXXXXX XXXXXXXXXX XXXX
gi222708647  XXXXXXXXXX XXXXXXXXXX XXXXXXXXXX XXXX
gi290760385  XXXXXXXXXX XXXXXXXXXX XXXXXXXXXX XXXX
gi559163786  XXXXXXXXXX XXXXXXXXXX XXXXXXXXXX XXXX
gi559163846  XXXXXXXXXX XXXXXXXXXX XXXXXXXXXX XXXX
gi318088612  XXXXXXXXXX XXXXXXXXXX XXXXXXXXXX XXXX
gi318088602  XXXXXXXXXX XXXXXXXXXX XXXXXXXXXX XXXX
gi222708673  XXXXXXXXXX XXXXXXXXXX XXXXXXXXXX XXXX
gi222708649  XXXXXXXXXX XXXXXXXXXX XXXXXXXXXX XXXX
gi222708671  XXXXXXXXXX XXXXXXXXXX XXXXXXXXXX XXXX
gi222708655  XXXXXXXXXX XXXXXXXXXX XXXXXXXXXX XXXX
gi222708681  XXXXXXXXXX XXXXXXXXXX XXXXXXXXXX XXXX
gi318088600  XXXXXXXXXX XXXXXXXXXX XXXXXXXXXX XXXX
gi318088598  XXXXXXXXXX XXXXXXXXXX XXXXXXXXXX XXXX
gi222708679  XXXXXXXXXX XXXXXXXXXX XXXXXXXXXX XXXX
gi318088608  XXXXXXXXXX XXXXXXXXXX XXXXXXXXXX XXXX
gi559164131  XXXXXXXXXX XXXXXXXXXX XXXXXXXXXX XXXX
gi318088606  XXXXXXXXXX XXXXXXXXXX XXXXXXXXXX XXXX
gi318088610  XXXXXXXXXX XXXXXXXXXX XXXXXXXXXX XXXX
gi222708677  XXXXXXXXXX XXXXXXXXXX XXXXXXXXXX XXXX
gi222708657  XXXXXXXXXX XXXXXXXXXX XXXXXXXXXX XXXX
gi700431350  AATNLTERQI TIWFQNRRVK EKKVVAKIKS SSSX
gi387231673  XXXXXXXXXX XXXXXXXXXX XXXXXXXXXX XXXX
gi661918810  XXXXXXXXXX XXXXXXXXXX XXXXXXXXXX XXXX
gi387231677  XXXXXXXXXX XXXXXXXXXX XXXXXXXXXX XXXX
gi387231659  XXXXXXXXXX XXXXXXXXXX XXXXXXXXXX XXXX
gi325260872  RVLNLTERQV KIWFQXXXXX XXXXXXXXXX XXXX
gi387231661  XXXXXXXXXX XXXXXXXXXX XXXXXXXXXX XXXX
gi387231655  XXXXXXXXXX XXXXXXXXXX XXXXXXXXXX XXXX
gi387231679  XXXXXXXXXX XXXXXXXXXX XXXXXXXXXX XXXX
gi387231667  XXXXXXXXXX XXXXXXXXXX XXXXXXXXXX XXXX
gi387231227  ATTNLSERQV TIWFQNRRVK EKKVINKLKT TSXX
gi387231653  XXXXXXXXXX XXXXXXXXXX XXXXXXXXXX XXXX
gi699575612  ATTNLSERQV TIWFQNRRVK EKKVINKLKT TSXX
gi676248171  ATTNLSERQV TIWFQNRRVK EKKVINKLKX XXXX
gi723145109  AATNLSERQV TIWFQNRRVK DKKIVSKLKD NVSX
gi697016891  AATNLSERQV TIWFQNRRVK DKKIVSKLKD NVSX
gi677147237  AATNLSERQV TIWFQNRRVK DKKIVSKLKD NVSX
gi256014558  XXXXXXXXXX XXXXXXXXXX XXXXXXXXXX XXXX
gi679242416  ATTNLSERQV TIWFQNRRVK EKKVINKXXX XXXX
gi683471095  AATNLTERQI TIWFQNRRVK EKKVVAKIKS SNSX
  gi9438781  RATNLSERQV TIWFQNRRVK DKKIVSKLKD TVSX
gi676827072  AATNLTERQI TIWFQNRRVK EKKVVAKIKS SSSX
gi387231651  XXXXXXXXXX XXXXXXXXXX XXXXXXXXXX XXXX
gi677498936  ATTNLSERQV TIWFQNRRVK EKKVINKLKX XXXX
gi683474613  PATNLSERQV TIWFQNRRVK DKKIVSKLKD NVSX
gi387231675  XXXXXXXXXX XXXXXXXXXX XXXXXXXXXX XXXX
gi677248448  ATTNLSERQV TIWFQNRRXX XXXXXXXXXX XXXX
gi678138507  AATNLTERQI TIWFQNRRVK EKKVVAKIKS SSSX
gi700349256  ATTNLSERQV TIWFQNRRXX XXXXXXXXXX XXXX
gi677225924  ATTNLSERQV TIWFQNRRXX XXXXXXXXXX XXXX
gi121308926  QSLTLSERQV KIWFQNRRMK QKKLNEKEKX XXXX
```

```
gi194591945  AHTNLTERQV TIWFQNRSMX XXXXXXXXXX XXXX
gi195549518  ATTNLSERQV TIWFQNRSMX XXXXXXXXXX XXXX
gi194591943  AHTNLTERQV TIWFQNSRMX XXXXXXXXXX XXXX
gi387231665  XXXXXXXXXX XXXXXXXXXX XXXXXXXXXX XXXX
gi195549516  ATTNLSERQV TIWFQXXXXX XXXXXXXXXX XXXX
 gi34304667  RDLSLTDRQV KIWFQNRRVK EKKXXXXXXX XXXX
gi194396133  AHTNLTERQV TIWFQNSSMX XXXXXXXXXX XXXX
gi387231669  XXXXXXXXXX XXXXXXXXXX XXXXXXXXXX XXXX
gi429510514  RLLNLTDRQV KIWFQNRRMK EKKLNXXXXX XXXX
gi780155006  RALSLTERQV KIWFQNRRMK LKKMRAREEN XXXX
gi387231671  XXXXXXXXXX XXXXXXXXXX XXXXXXXXXX XXXX
gi387231657  XXXXXXXXXX XXXXXXXXXX XXXXXXXXXX XXXX
gi387231649  XXXXXXXXXX XXXXXXXXXX XXXXXXXXXX XXXX
gi229298086  ESSSLSERQV MIWFQNRRMK EKKLXXXXXX XXXX
gi328682725  AVTNLSERQI TIXXXXXXXX XXXXXXXXXX XXXX
gi217035843  ESSSLSERQV MIWFQNRRMK EKKLXXXXXX XXXX
```
